# Epstein-Barr virus-encoded microRNA-BART17-5p suppresses cell proliferation and metastasis by targeting CERK

**DOI:** 10.1371/journal.ppat.1014519

**Published:** 2026-09-15

**Authors:** Peilin Wu, Xiazhen Xu, Qiong Wu, Lu Zhang, Fan Xin, Xiaowei Zheng, Xu Lin, Wannan Chen

**Affiliations:** 1 Key Laboratory of Gastrointestinal Cancer (Fujian Medical University), Ministry of Education, School of Basic Medical Sciences, Fujian Medical University, Fuzhou, China; 2 Department of paediatrics, the First Affiliated Hospital of Fujian Medical University, Fuzhou, China; 3 Fujian Key Laboratory of Tumor Microbiology, Department of Medical Microbiology, School of Basic Medical Sciences, Fujian Medical University, Fuzhou, China; Wistar Institute, UNITED STATES OF AMERICA

## Abstract

Epstein-Barr virus (EBV) is an oncogenic DNA virus that is etiologically associated with multiple malignancies, including nasopharyngeal carcinoma, gastric carcinoma, and lymphomas. Although EBV encodes numerous viral microRNAs (miRNAs), the pathogenic functions of individual miRNAs remain incompletely characterized. In this study, we identify EBV-encoded miR-BART17-5p as a tumor-suppressive miRNA that directly targets ceramide kinase (CERK). CERK expression is significantly downregulated in EBV-associated gastric cancer (EBVaGC) tissues and EBV-positive AGS-EBV cells and inversely correlates with miR-BART17-5p levels. Mechanistic analyses further demonstrate that miR-BART17-5p directly targets the 3′ untranslated region (3′UTR) of *CERK*, thereby regulating its expression. Overexpression of miR-BART17-5p mimics or knockdown of *CERK* in AGS cells significantly suppresses tumor -associated phenotypes, including cell proliferation, migration, and invasion. Conversely, inhibition of miR-BART17-5p in AGS-EBV cells rescues these phenotypes. Similar results were obtained in SNU719 cells. Mechanistically, miR-BART17-5p inhibits the PI3K/AKT signaling by targeting *CERK*. Restoration of CERK expression reactivates PI3K/AKT signaling and reverses the inhibitory effects on cell proliferation, migration, and invasion in AGS cells. To evaluate the therapeutic potential of miR-BART17-5p, systemic administration of engineered cholesterol-conjugated miR-BART17-5p agomirs significantly suppresses tumor growth in xenograft models. Collectively, our findings reveal a previously unrecognized EBV-host interaction whereby miR-BART17-5p constrains cancer progression through modulation of CERK/PI3K/AKT signaling activation, highlighting its potential as a preclinical therapeutic strategy for cancer.

## Author summary

Epstein-Barr virus (EBV) is a common virus that infects most people worldwide and is linked to several types of cancer, including cancers of the stomach, nose, and blood system. Although this virus produces many small regulatory molecules, microRNAs, the roles of many of these molecules in cancer development remain unclear. In this study, we investigated the function of one viral microRNA, known as miR-BART17-5p, and its impact on cancer progression. We found that miR-BART17-5p acts as a natural brake on cancer growth by reducing the activity of a host protein, ceramide kinase (CERK), which normally promotes signals that support cell growth and spread. When levels of miR-BART17-5p were increased, cells grew more slowly and were less able to move and invade surrounding tissues. In contrast, blocking this microRNA restored aggressive cancer behaviors. We also showed that restoring miR-BART17-5p in animal models using a chemically modified mimic significantly reduced tumor growth. Our findings reveal an unexpected way in which a virus can restrain cancer development by regulating host cell signaling. This work improves our understanding of virus-host interactions and suggests that viral microRNAs may be developed as candidate molecules for future therapeutic development in cancers associated with EBV.

## Introduction

Epstein-Barr virus (EBV), also known as human herpesvirus 4 (HHV-4), is a double-stranded DNA virus with a linear genome of approximately 170 kb. EBV was the first human virus identified as oncogenic and is etiologically associated with multiple malignancies, with more than 90% of adults worldwide harboring lifelong infection [1–3]. Following entry into host cells, EBV establishes either lytic or latent infection programs. EBV was also among the first oncoviruses shown to encode viral microRNAs (miRNAs), which interact with both viral and host targets and contribute to virus-host regulatory networks [4,5].

MiRNAs are 21–23 nucleotide noncoding RNAs that mediate post-transcriptional gene silencing by guiding the RNA-induced silencing complex (RISC) to partially or fully complementary sequences within the 3′-untranslated regions (3′-UTRs) of target mRNAs, thereby repressing translation or inducing mRNA degradation [6]. EBV was the first human virus discovered to encode miRNAs [5,7] and expresses 44 mature miRNAs derived from two genomic clusters [8], including 4 from the BamHI fragment H rightward open reading frame 1 (BHRF1) region and 40 from the BamHI-A rightward transcripts (BARTs) region. BHRF1 miRNAs are mainly expressed in lymphocytes but are rarely detected in epithelial cells. In contrast, BART miRNAs are highly expressed in most EBV-positive epithelial malignancies, including EBV-associated gastric cancer (EBVaGC), and can also be detected, albeit at lower levels, in EBV-transformed lymphocytes and lymphoblastoid cell lines [9].

Ceramide kinase (CERK), a key regulator of sphingolipid metabolism, phosphorylates ceramide to generate ceramide-1-phosphate (C1P) and has emerged as an oncogenic driver in several malignancies, including ovarian, breast, and lung cancers [10–18]. CERK has been implicated in promoting tumor cell proliferation, migration, and resistance to therapy through activation of the PI3K/AKT and Ras/ERK signaling pathways across multiple cancer types [11–13,15,17]. Notably, recent studies have demonstrated that miRNAs can regulate CERK-mediated oncogenic signaling. For instance, miR-34a suppresses *CERK* expression to modulate AKT and IRS1 phosphorylation [19]. In addition, accumulating evidence indicates that EBV-encoded miRNAs contribute to EBV-associated tumorigenesis by targeting host genes involved in cancer-related pathways [7,20–23].

Our previous bioinformatics analyses predicted that the six mature miRNAs encoded by EBV could target nine host genes, including *CERK*, *PPARα*, *USP2*, *TRIB3*, *HSD17B12*, *SACS*, *MPP6*, *NT5DC3* and *KHDRBS3* [23]. Among these, the function and mechanism of miR-BART20-3p in targeting the host gene *PPARα* have been elucidated [23]. In this study, we investigated the functional relationship and underlying mechanisms between miR-BART17-5p and *CERK*. We demonstrated that miR-BART17-5p directly suppresses *CERK* through a putative 3’-UTR binding sequence, thereby attenuating PI3K/AKT signaling and inhibiting cell proliferation, migration, and invasion. Given the inherent biostability of viral miRNAs, we further developed cholesterol-conjugated miR-BART17-5p agomirs, which induced tumor regression in gastric cancer models. This work highlights the potential therapeutic repurposing of a viral regulatory molecule into a targeted agent against gastric cancer.

## Materials and methods

### Ethics statement

This study was conducted in accordance with ethical standards. The use of clinical gastric cancer tissues was approved by The Ethics Committee of Fujian Medical University (Approval No. FJMU-EC-2025–261). All animal experiments were approved by The Institutional Animal Care and Use Committee of the same institution (Protocol No. IACUC FJMU 2024–0401). Formalin-fixed, paraffin-embedded (FFPE) tissue blocks were obtained from the First Affiliated Hospital of Fujian Medical University.

### Kaplan-Meier survival analysis

Kaplan-Meier survival analysis of *CERK* expression in gastric cancer was performed using the Kaplan-Meier Plotter database (https://kmplot.com/analysis), which integrates gene expression and clinical outcome data from GEO, EGA, and TCGA in January 2024. The cohort contained 875 patients. Patients were stratified into high- and low-*CERK* expression groups using the platform’s auto-selected best cutoff. Overall survival was analyzed using the Kaplan-Meier method with log-rank testing. Hazard ratios (HRs) and 95% confidence intervals (CIs) were obtained from the platform. The exact parameters used included Affy ID 218421_at (*CERK*), with all clinicopathological filters set to “all”. Risk table numbers: at baseline, low n = 622, high n = 253; at 50 months: 231 vs. 67; at 100 months: 41 vs. 7; at 150 months: 1 vs. 0.

### Gene expression analysis of public dataset

Gene expression data for gastric cancer were obtained from the Gene Expression Omnibus (GEO) database under accession number GSE62254 (https://www.ncbi.nlm.nih.gov/geo/query/acc.cgi?acc=GSE62254). This dataset comprises 300 primary gastric adenocarcinoma samples and was generated on the Affymetrix Human Genome U133 Plus 2.0 Array platform (Affymetrix, Santa Clara, CA, USA). The raw CEL files were processed and normalized using the Robust Multi-array Average (RMA) method as previously described. Clinical annotation, including EBV status, was obtained from the Asian Cancer Research Group (ACRG) classification, in which samples were categorized as EBVaGC or EBVnGC based on consensus clustering of gene expression profiles. *CERK* expression levels (Affymetrix probe ID 218421_at) were extracted from the normalized expression matrix. Statistical comparison between EBVaGC (n = 39) and EBVnGC (n = 235) groups was performed using the Mann-Whitney U test, with statistical significance set at *P* < 0.05. All analyses were conducted using R software (version 4.0.3, R Foundation for Statistical Computing, Vienna, Austria).

### Cell culture and reagents

The human gastric adenocarcinoma cell line AGS (EBV-negative) was obtained from the American Type Culture Collection (Catalog No.CRL-1739, ATCC, Manassas, VA, USA). SNU719 is a gastric cancer cell line naturally infected with EBV was purchased from FuHeng biology (Shanghai, China). The isogenic AGS-EBV cell line was established in-house by infection with recombinant EBV derived from Akata cells [23], which was a kind gift from Professor Musheng Zeng (Affiliated Cancer Hospital of Sun Yat-sen University, China). All cell lines were routinely assessed and confirmed to be free of mycoplasma contamination. AGS and AGS-EBV cells were maintained in Dulbecco's Modified Eagle Medium/Nutrient Mixture F-12 (DMEM/F12; Catalog No. 11320033, Gibco, Waltham, MA, USA) whereas SNU719 cells were cultured in RPMI‑1640 medium (Catalog No. 11875093, Gibco, Waltham, MA, USA), both supplemented with 10% fetal bovine serum (FBS; Catalog No. P30-1302, PAN-Biotech, Aidenbach, Germany) at 37℃ in a 5% CO_2_ humidified incubator. For the AGS-EBV cell line, 400 μg/mL G418 (Catalog No. 11811031, Gibco) was added to the culture medium to sustain EBV episomes. No G418 was added for SNU719 cells. The PI3K inhibitor LY294002 (Catalog No. S1105) and the CERK inhibitor NVP231 (Catalog No. S6501) were purchased from Selleck Chemicals (Houston, TX, USA) and were dissolved in dimethyl sulfoxide (DMSO; Catalog No. D2650, Sigma-Aldrich, St. Louis, MO, USA) to prepare stock solutions. For functional assays, cells were treated with LY294002 at a final concentration of 40 μM or with NVP231 at 800 nM for 24 h. Nucleic acid transfection (for both plasmids and oligonucleotides) was performed using Lipofectamine 3000 reagent (Catalog No. L3000015, Invitrogen, Waltham, MA, USA) according to the manufacturer’s protocol.

### Immunohistochemistry (IHC)

IHC staining was performed on 3–4 μm sections from the above FFPE blocks. Following deparaffinization, antigen retrieval was conducted using pH 9.0 EDTA buffer (Catalog No. ZLI-9071, ZSGB-BIO, Beijing, China) under high-pressure steam for 3 min. After blocking, sections were incubated overnight at 4℃ with a primary antibody against CERK (Catalog No. ab155061, Abcam, Cambridge, UK). Subsequently, sections were incubated with an HRP-conjugated secondary antibody (Catalog No. ZB-2306, ZSGB-BIO) for 20 min at room temperature. Signal visualization was performed using a DAB substrate kit (Catalog No. ZLI-9018, ZSGB-BIO), followed by hematoxylin counterstaining, dehydration, and mounting. Stained sections were observed and analyzed under a light microscope.

### EBER in situ hybridization (ISH)

EBER in situ hybridization was performed on 4 μm FFPE sections from confirmed EBVaGC cases using a commercial kit (Catalog No. ISH-7001, ZSGB-BIO) with protocol optimization. Briefly, sections were baked (60℃, 2 h), deparaffinized, and digested with pepsin (37℃, 30 min). Following hybridization with a DIG-labeled EBER probe at 37℃ overnight, signals were detected with an HRP-conjugated anti-DIG antibody and visualized using DAB substrate. Sections were then counterstained with hematoxylin. Positive nuclear signals were assessed by a qualified pathologist.

### Target prediction

The 3′-UTR sequence of human *CERK* (NCBI Reference Sequence: NM_022766.6) was analyzed for potential binding sites of EBV-encoded miRNAs. To enhance prediction reliability and minimize potential false positives and false negatives, three widely used miRNA target prediction tools were employed simultaneously, including RNAhybrid (http://bibiserv.cebitec.uni-bielefeld.de/rnahybrid), RNA22 v2 (https://cm.jefferson.edu/rna22v2), and miRanda (https://bioweb.pasteur.fr/packages/pack@miRanda@3.3a). Only the candidate binding sites consistently predicted by all three algorithms were selected for subsequent experimental validation.

### Plasmids and oligonucleotides

Plasmids and oligonucleotides used in this study are summarized as follows (detailed sequences and sources are provided in S1 Table). The *CERK* expression plasmid phouge-CERK (abbreviated as pCERK) was synthesized from Tsingke Biotechnology by inserting the full-length human *CERK* sequences into the empty vector pCDH-EF1-MCS-T2A-Puro (abbreviated as phouge) (Catalog No. CD527A-1, System Biosciences, Palo Alto, CA, USA). Wild-type and mutant *CERK* 3′-UTR recombinant reporter plasmids, pmirGLO-CERK 3′-UTR-WT (abbreviated as CERK WT) and pmirGLO-CERK 3′-UTR-MUT (abbreviated as CERK MUT), were custom-synthesized by GenePharma (China) using the pmirGLO dual-luciferase reporter vector (Catalog No. E1330, Promega, Madison, WI, USA). This vector contains both Firefly luciferase and Renilla luciferase genes on a single plasmid, allowing normalization of transfection efficiency within the same construct and minimizing potential variability caused by co-transfection of separate plasmids. For *in vitro* studies, the miR-BART17-5p mimics and inhibitor, corresponding negative controls, and siRNAs (si-CERK#1, #2 and si-NC) were from GenePharma. For *in vivo* studies, miR-BART17-5p agomir and antagomir and controls were from RiboBio Technologies (China). The negative control agomir sequence corresponds to C. elegans cel-miR-67-5p, a widely used non-targeting control in mammalian studies [24,25].

### Luciferase reporter assay

For AGS cells, co-transfection was performed with 20 nM of the miR-BART17-5p mimics (abbreviated as B17-5p mimics) along with either the pmirGLO, CERK WT, or CERK MUT reporter plasmid. For AGS-EBV cells or SNU719 cells, co-transfection was performed with 20 nM of the miR-BART17-5p inhibitor (abbreviated as B17-5p inhibitor) along with either the pmirGLO, CERK WT, or CERK MUT reporter plasmid. At 48 h post-transfection, luciferase activity was measured using the Dual-Glo luciferase reporter assay system (Catalog No. E2940, Promega). Firefly luciferase activity was normalized to Renilla luciferase activity to control for transfection efficiency. Subsequently, the normalized Firefly/Renilla ratio for each sample was further expressed relative to the mean ratio of the corresponding negative control group, consisting of pmirGLO with NC-mimics or NC-inhibitor, from three independent experiments. Data are presented as mean ± SD of three independent experiments, and statistical comparisons were performed using these normalized ratios.

### Reverse transcription quantitative PCR (RT-qPCR)

Total RNA was extracted with TRIzol (Catalog No. 15596026, Invitrogen). cDNA was synthesized using separate systems: the Evo M-MLV RT Premix Kit (Catalog No. AG11728, Agbio, Hunan, China) with random hexamers for mRNA, and the Evo M-MLV RT Kit (Catalog No. AG11707, Agbio) with gene-specific stem-loop primers for miRNA. qPCR was performed using the GreenPro Taq HS Premix Kit (Catalog No. AG11701, Agbio) on a Gentier 96E system (TIANLONG, Xi'an, China). For mRNA (e.g., *CERK*), expression levels were normalized to GAPDH and calculated using the 2^-ΔΔCt method. For miR‑BART17-5p, expression was normalized to U6 and quantified using the 2^-ΔCt method. ΔCt = Ct_miR-BART17-5p_-Ct_U6_. Primer sequences are in S2 Table.

### Western blotting

Cells were lysed, and proteins (50 μg per lane) were separated by SDS-PAGE (10% or 12% gels) and transferred to PVDF membranes. After blocking with 5% bovine serum albumin (BSA), membranes were incubated with primary antibodies (see S3 Table for list and dilutions) overnight at 4℃, followed by HRP-conjugated secondary antibodies (1:2000). Signals were detected with ECL reagent (Catalog No. RPN2232, Amersham, Buckinghamshire, UK) and captured using either an Image Quant LAS 4000 mini system (Cytiva, Marlborough, MA, USA) or X-ray films (Fujifilm, Tokyo, Japan) in a darkroom. All chemiluminescent signals quantified with ImageJ software (National Institutes of Health, Bethesda, MD, USA).

### Cell counting kit-8 (CCK-8) assay

The CCK-8 assay was conducted using the CCK-8 reagent (Catalog No. CK04, Dojindo, Tokyo, Japan) in accordance with the manufacturer's instructions. Briefly, 24 h post-transfection, cells were harvested and seeded into 96-well plates at a density of 1,000 cells per well. Starting from the day of seeding, designated as Day 1, the culture medium was carefully removed at the same time point for four consecutive days. Each well was then replenished with 100 µL of fresh culture medium containing 10 µL of CCK-8 reagent. The plates were incubated in a dark environment at 37°C for 2 h. Following incubation, the absorbance of each well was measured at a wavelength of 450 nm using a multimode plate reader (PerkinElmer, Shelton, CT, USA). The assay was performed daily for a total of five days to generate a growth curve.

### EdU cell proliferation assay

The 5-ethynyl-2'-deoxyuridine (EdU) incorporation assay was employed in this study as one of the methods to evaluate cell proliferation. Briefly, cells were labeled using a commercial EdU imaging kit (Catalog No. KTA2031, Abbkine, Wuhan, China) following the manufacturer’s instructions. After fixation and permeabilization, EdU staining and nuclear counterstaining were performed sequentially. Fluorescence images were captured using a Zeiss fluorescence microscope (Zeiss Axio Observer 7, Zeiss, Oberkochen, Germany). Proliferating cells were identified by red fluorescence (Ex/Em: 546/565 nm) from EdU incorporation, while all nuclei were stained blue fluorescence (Ex/Em: 358/461 nm). In the merged images, nuclei positive for EdU appeared pink. The ratio of EdU-positive cells to total nuclei was quantified using ImageJ software.

### Wound-healing assay

Cells were seeded into 35-mm culture plates and transfected with 20 nM of B17-5p mimics, inhibitor, or their respective controls. After 6 h of transfection, a straight scratch was generated in the confluent cell monolayer using a sterile 200 µL pipette tip. The detached cells and debris were removed by gently washing with phosphate-buffered saline (PBS). Subsequently, the cells were maintained under standard culture conditions. Wound images were captured immediately (0 h) and 24 h after scratching using a Zeiss Axio Observer 7 inverted microscope, and the wound widths were quantified using ZEN software (version 3.4, Zeiss). Cell migration was evaluated by comparing wound closure across different transfection groups.

### Transwell migration and invasion assay

The migratory and invasive capabilities of cells were evaluated using a Transwell chamber system (24-well format, Corning, USA). For the migration assay, standard cell culture inserts with 8.0 μm pore transparent polyethylene terephthalate (PET) membranes were utilized (Catalog No. 353097, Corning Inc., Corning, NY, USA). The invasion assay was conducted using Matrigel-coated invasion chambers featuring 8.0 μm pore PET membranes (Catalog No. 354480, Corning Inc.). For AGS and AGS‑EBV cells, 24 h after transfection with either 20 nM B17-5p mimics or inhibitor, AGS or AGS-EBV cells were collected and resuspended in serum-free medium. For SNU719 cells, 24 h after transfection with 20 nM B17‑5p inhibitor or its negative control (NC inhibitor), cells were collected and resuspended in serum‑free medium. Subsequently, approximately 5 × 10⁴ AGS or AGS-EBV or 5 × 10^5^ SNU719 cells in 500 μL of serum-free suspension were seeded into the upper compartment of each chamber. The lower compartment was filled with 800 μL of complete medium containing 10% fetal bovine serum (FBS), which served as a chemoattractant. Following a 20 h incubation period under standard culture conditions, non-migrated or non-invaded cells remaining on the upper surface of the membrane were gently removed with a cotton-tipped swab. Cells that had migrated or invaded to the lower surface were fixed with 4% paraformaldehyde for 15 min and then stained with 0.1% crystal violet for 20 min. The stained membranes were imaged using a Zeiss Axio Observer 7 inverted microscope, and the number of migrated/invaded cells was quantified using ImageJ software in five randomly selected fields per membrane.

### Animal studies

To investigate the exogenous tumor-suppressive function of miR-BART17-5p, six-week-old female NSG mice (NM-NSG-001, Model Organisms, Shanghai, China) were subcutaneously inoculated with parental AGS cells (5 × 10⁶ cells per mouse), which lack endogenous expression of this miRNA. When tumors reached approximately 5 mm in diameter, mice were randomized into two groups (n = 5 per group) for intratumoral treatment. Mice received injections of either a miR-BART17-5p agomir or a negative control agomir (NC-agomir) (10 nmol per mouse, twice weekly, RiboBio Technologies). The negative control agomir corresponds to C. elegans cel-miR-67-5p, a widely used non-targeting control in mammalian studies with no known endogenous targets in human or mouse.

To assess the oncogenic consequence of inhibiting endogenous miR-BART17-5p, a separate cohort of NSG mice was subcutaneously inoculated with AGS-EBV cells (5 × 10^6^ cells per mouse), which express this miRNA endogenously. Once tumors were established (~5 mm in diameter), mice were randomized into two groups (n = 5 per group) to receive intratumoral injections of either a miR-BART17-5p antagomir or its corresponding negative control (NC-antagomir) (10 nmol per mouse, twice weekly, RiboBio Technologies). For both studies, tumor growth was monitored by measuring the longest diameter (A) and the perpendicular diameter (B) every 3–4 days; volume was calculated as (A × B^2^) / 2. Mice were euthanized after 3–4 weeks of treatment or if the longest tumor diameter approached 15 mm, whichever came first. Subcutaneous tumors were excised, weighed, and processed. A minimum of n = 3 tumors per group were allocated for subsequent Western blotting and IHC analyses.

### Statistical analysis

Quantitative data are presented as mean ± SD from at least three independent experiments. Comparisons between two groups were performed using the Student’s t-test (for normally distributed data) or the Mann-Whitney U test (for non-normally distributed data), as specified in the figure legends; if not otherwise stated, the Student’s t-test was applied. Comparisons among multiple groups were analyzed by one-way ANOVA with appropriate post-hoc tests. Tumor growth curves in vivo were compared using two-way ANOVA. Survival analysis was conducted using the Kaplan-Meier method with the log‑rank test, based on a referenced gastric cancer cohort [26]. Unless noted otherwise, exact P‑values are reported in the figures. In the animal experiments presenting tumor volume changes, statistical significance is denoted directly on the figures as *P < 0.05, **P < 0.01, ***P < 0.001, or ns (not significant) due to space constraints. All statistical analyses were performed using IBM SPSS Statistics 26 (IBM Corp., Armonk, NY, USA).

## Results

### EBV infection downregulates CERK expression

**Fig 1:**
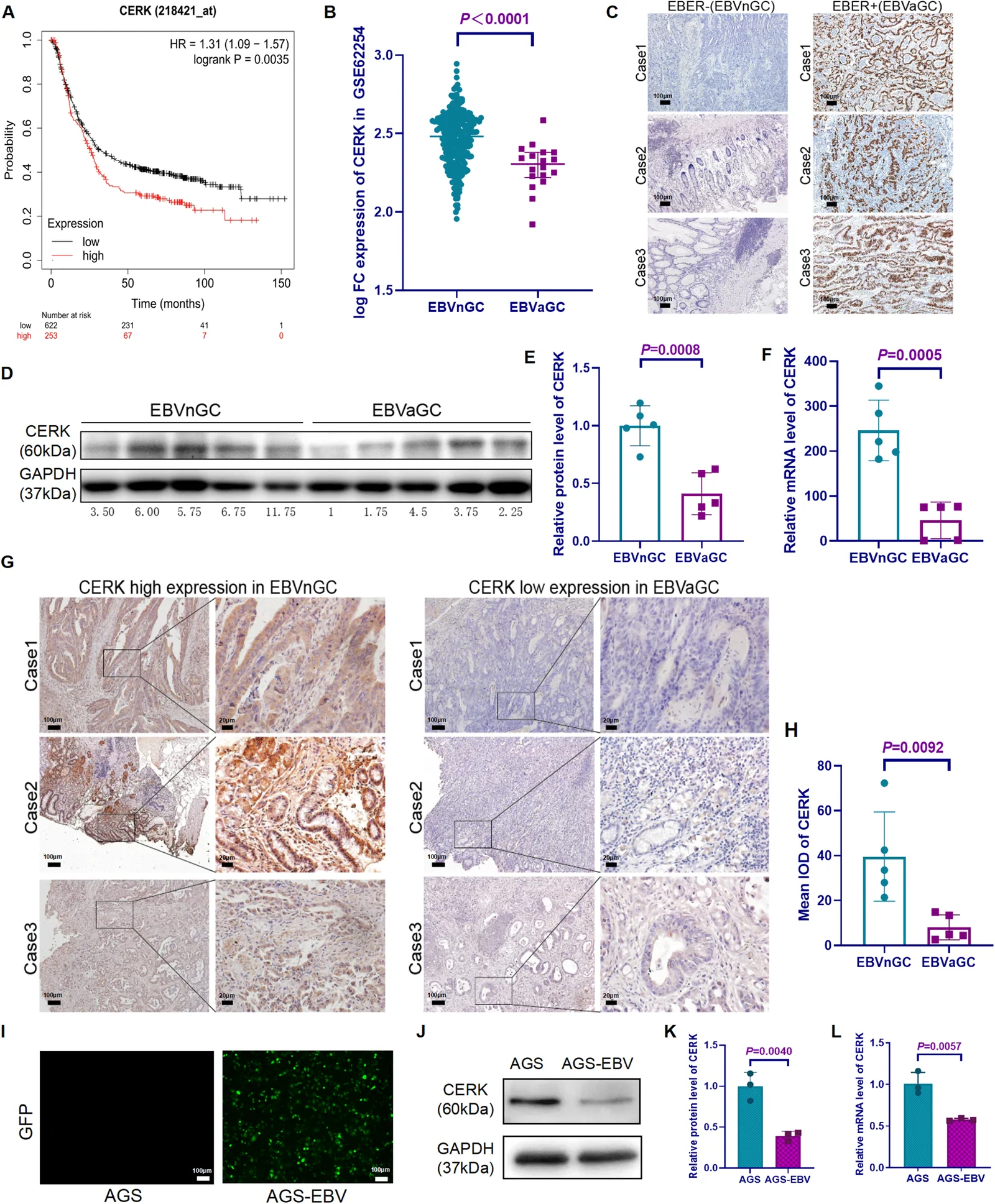
EBV infection downregulates CERK expression. (A) Kaplan-Meier analysis of overall survival in 875 gastric cancer patients from the Kaplan-Meier Plotter database, stratified by high versus low *CERK* expression using the auto-selected best cutoff (HR = 1.31, 95% CI = 1.09**–**1.57, log‑rank P = 0.0035). (B) Analysis of *CERK* mRNA expression in the GSE62254 dataset shows significant downregulation in EBVaGC compared to EBVnGC tissues (n = 274; Mann-Whitney U test, *P* < 0.0001). (C) Representative images of EBER in situ hybridization (EBER-ISH) confirming EBV status in clinical gastric cancer samples (scale bar: 100 μm). (D, E) Western blotting analysis of CERK protein in clinical samples. Representative blots (D) and quantitative analysis normalized to GAPDH (E) demonstrate reduced CERK levels in EBVaGC (n = 10). (F) RT-qPCR analysis of *CERK* mRNA levels in EBVnGC and EBVaGC tissues, normalized to U6 (n = 10). (G) IHC staining of CERK in EBVnGC and EBVaGC samples (scale bars: 100 μm and 20 μm). (H) Quantification of CERK IHC staining intensity (Integrated Optical Density, IOD) in tumor epithelium (n = 10). (I) Fluorescence images of isogenic AGS (EBV-negative) and AGS-EBV (EBV-positive) cell lines. GFP signals indicate EBV (scale bar: 100 μm). (J, K) Western blotting analysis of CERK in AGS and AGS-EBV cells. Representative blots (J) and quantification normalized to GAPDH (K) confirm lower CERK expression upon EBV infection (n = 3). (L) RT-qPCR analysis of *CERK* mRNA levels in AGS and AGS-EBV cells (n = 3). For all quantitative panels (E, F, H, K, L), individual data points are shown as dots, and bars represent mean ± SD.

CERK has been implicated in poor clinical prognosis across multiple cancer types. In gastric cancer (GC), analysis of a large patient cohort (n = 875) from the Kaplan‑Meier Plotter database demonstrated that high *CERK* expression (n = 253) correlates with poor overall survival compared with low *CERK* expression (n = 622; HR = 1.31, 95% CI = 1.09**–**1.57, log‑rank P = 0.0035; Fig 1A), highlighting its broader role in tumor biology. In our prior bioinformatic screening, *CERK* expression was significantly downregulated in EBVaGC compared to EBVnGC (EBV-negative gastric cancer) [23]. This observation was independently validated using a public transcriptomic dataset (GSE62254, Fig 1B). To further investigate the regulation of CERK in a viral-associated context, we validated this relationship in clinical GC samples, in which EBV status was confirmed by EBER-ISH (Fig 1C). Characteristics of EBVnGC patients and EBVaGC patients are summarized in Table 1. Consistent with our bioinformatic predictions, CERK expression was significantly reduced in EBVaGC at both the protein level (as assessed by Western blotting, Fig 1D-1E) and at mRNA level (as determined by RT-qPCR, Fig 1F), and this reduction was also confirmed by immunohistochemistry (Fig 1G-1H). The suppressive effect of EBV infection on CERK expression was further confirmed *in vitro* using isogenic cell lines pairs (AGS and AGS-EBV). Successful EBV infection was verified by GFP reporter expression (Fig 1I), and lower CERK expression was observed in EBV-positive cells by Western blotting (Fig 1J-1K) and RT-qPCR (Fig 1L). Together, these consistent results across datasets, clinical samples, and cellular models demonstrate that EBV infection significantly downregulates CERK expression. To identify the viral factor responsible for this effect, we focused on miR-BART17-5p, a candidate miRNA predicted to target *CERK* in our previous analysis [23].

**Table 1:** Characteristics of EBVnGC patients and EBVaGC patients.

Characteristics	EBVnGC	EBVaGC
	n = 5	n = 5
**Age (years)**		
<60	0	2
≧60	5	3
**Gender**		
Male	3	4
Female	2	1
**Size**		
<5 cm	1	2
≧ 5 cm	4	3
**Differentiation**		
Well/Moderate	2	2
Poor	3	3
**Lauren classification**		
Intestinal	4	4
Diffuse	1	1
**T stage**		
T1 + T2	1	0
T3 + T4	4	5
**Lymph node status**		
Negative	4	3
Positive	1	2
**Distant metastasis**		
Negative	5	5
Positive	0	0

### MiR-BART17-5p suppresses CERK expression via direct 3′-UTR binding

**Fig 2:**
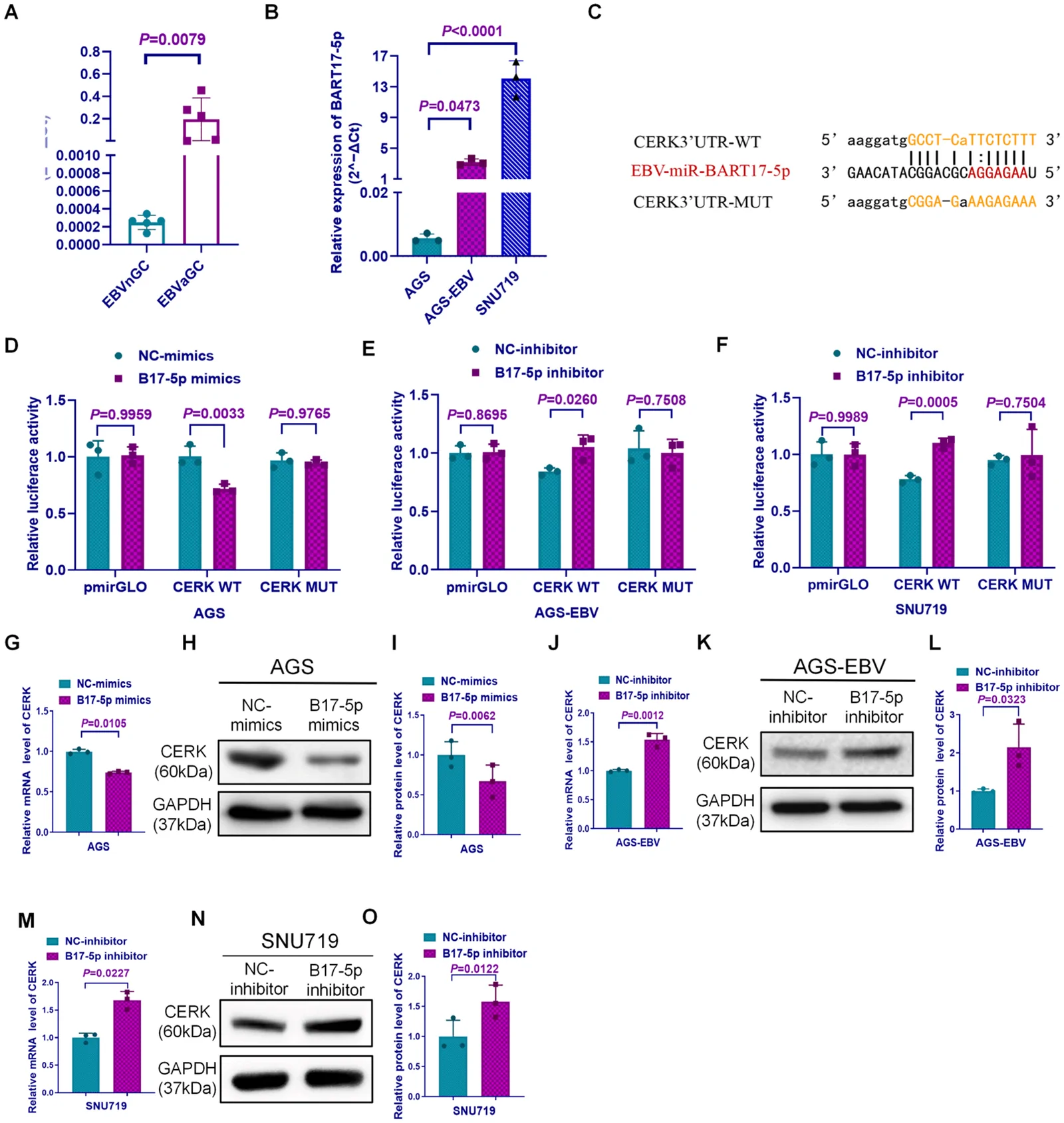
miR-BART17-5p directly targets CERK. (A) Expression levels of miR-BART17-5p in clinical EBVaGC (n = 5) and EBVnGC (n = 5) tissues. Data are presented as relative expression calculated using the 2^-ΔCt method (normalized to U6), ΔCt = Ct_miR‑BART17‑5p_-Ct_U6_. (B) Expression levels of miR‑BART17‑5p in AGS, AGS‑EBV, and SNU719 cells (n = 3 per group). Data are shown as relative expression calculated using the 2^-ΔCt method (normalized to U6), ΔCt = Ct_miR‑BART17‑5p_-Ct_U6_. (C) Predicted binding of miR-BART17-5p to the 3′-UTR of *CERK* mRNA. Complementary base pairs are indicated by yellow uppercase letters and solid lines, mismatches are shown in lowercase with dashed lines. Schematic of the wild-type (WT) and mutant (MUT) *CERK* reporter constructs. (D) Luciferase reporter assay in AGS cells co‑transfected with B17-5p mimics or NC-mimics together with WT or MUT *CERK* 3’ UTR reporter (n = 3). Firefly luciferase activities were normalized to Renilla and measured 48 h post‑transfection. (E) Luciferase reporter assay in AGS‑EBV cells co‑transfected with B17-5p inhibitor or NC-inhibitor together with WT or MUT *CERK* 3’ UTR reporter (n = 3). (F) Luciferase reporter assay in SNU719 cells co-transfected with B17‑5p inhibitor or NC-inhibitor together with WT or MUT reporter (n = 3). (G) RT-qPCR analysis of *CERK* mRNA expression in AGS cells transfected with B17-5p mimics or NC-mimics (n = 3). (H, I) Western blotting analysis of CERK protein expression in AGS cells transfected with B17-5p mimics or NC-mimics. Representative blots (H) and quantitative analysis normalized to GAPDH (I) are shown (n = 3). (J) RT-qPCR analysis of *CERK* mRNA expression in AGS‑EBV cells transfected with B17-5p inhibitor or NC-inhibitor (n = 3). (K, L) Western blotting analysis of CERK protein expression in AGS-EBV cells transfected with B17-5p inhibitor or NC-inhibitor. Representative blots (K) and quantitative analysis normalized to GAPDH (L) are shown (n = 3). (M) RT-qPCR analysis of *CERK* mRNA expression in SNU719 cells transfected with miR‑BART17-5p inhibitor or NC-inhibitor (n = 3). (N, O) Western blotting analysis of CERK protein expression in SNU719 cells transfected with B17-5p inhibitor or NC-inhibitor. Representative blots (N) and quantitative analysis (O) (n = 3). For all quantitative panels (A, B, D, E, F, G, I, J, L, M, O), individual data points are shown as dots, and bars represent mean ± SD.

To validate the relationship between miR-BART17-5p and CERK, we first examined the expression levels of miR-BART17-5p in tissues and cell lines. Compared with EBVnGC tissues, miR-BART17-5p was significantly detected in EBVaGC tissues (Fig 2A), and miR-BART17-5p was specifically expressed in AGS-EBV and SNU719 cells (with levels in SNU719 even exceeding those in AGS-EBV), whereas it was undetectable in parental AGS cells (Fig 2B). RNAhybrid, RNA22 v2, and miRanda co-analysis consistently predicted a putative miR-BART17-5p binding site within the 3′-UTR of *CERK* mRNA (Fig 2C). To experimentally validate this interaction, we performed dual-luciferase reporter assays using constructs containing either the wild-type (WT) *CERK* 3′-UTR or a mutant (MUT) version with a disrupted seed-matching sequence. In AGS cells, transfection with miR-BART17-5p mimics significantly reduced the luciferase activities driven by the WT reporter but had no effect on the MUT construct (Fig 2D). Conversely, in AGS-EBV cells, inhibition of endogenous miR-BART17-5p significantly increased the activities of the WT reporter, while the MUT construct remained unaffected (Fig 2E). Consistently, using SNU719 cells, which naturally harbor EBV, we also observed that inhibition of endogenous miR‑BART17‑5p significantly enhanced the activity of the WT *CERK* 3′‑UTR reporter, whereas the MUT reporter showed no change (Fig 2F). Overexpression of miR-BART17-5p in AGS cells markedly reduced CERK expression at both the mRNA (Fig 2G) and protein levels (Fig 2H-2I). Conversely, inhibition of endogenous miR-BART17-5p in AGS-EBV cells significantly increased CERK expression (Fig 2J-2L). Furthermore, inhibition of endogenous miR‑BART17‑5p in SNU719 cells also led to a significant increase in CERK expression at the mRNA (Fig 2M) and protein levels (Fig 2N-2O).

### MiR-BART17-5p inhibits GC cell proliferation, migration, and invasion via targeting *CERK*

**Fig 3:**
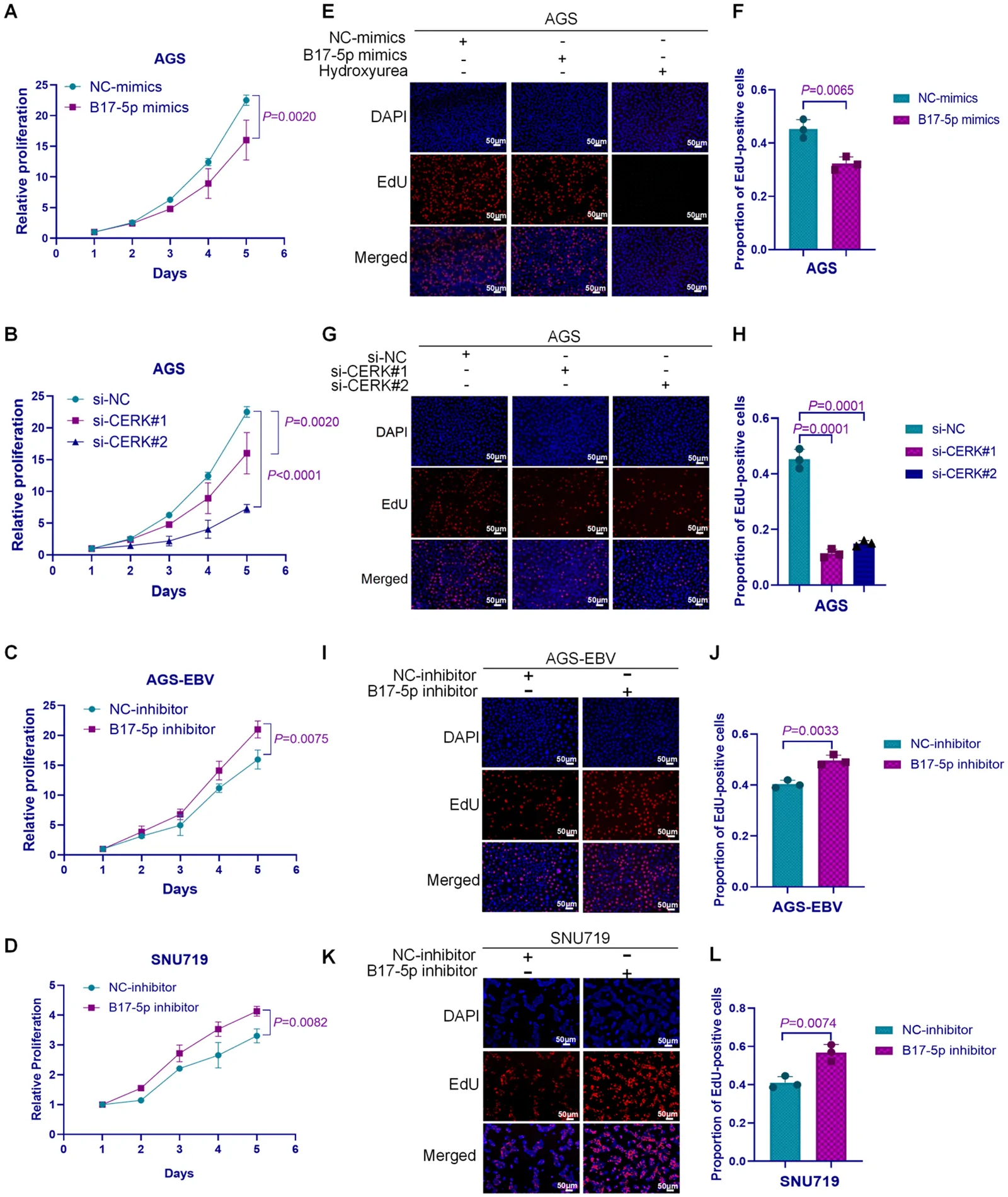
CERK downregulation by miR-BART17-5p inhibits GC cell proliferation. CCK-8 assays measuring proliferation of AGS at 1, 2-, 3-, 4-, and 5-day post-transfection, data normalized to Day 1 with baseline correction. (A) CCK-8 proliferation curves of AGS cells transfected with B17-5p mimics or NC-mimics (n = 3). (B) CCK-8 proliferation curves of AGS cells transfected with *CERK* siRNA (si-CERK#1 and #2) or si-NC (n = 3). (C) CCK-8 proliferation curves of AGS-EBV cells treated with B17-5p inhibitor or NC-inhibitor (n = 3). (D) CCK-8 proliferation curves of SNU719 cells treated with B17-5p inhibitor or NC-inhibitor (n = 3). EdU-positive cells (EdU^+^ cells, red) and total cells (nuclei, blue) were counted from 5 random fields per group using ImageJ software. Cell proliferation was quantified as the proportion of EdU-positive cells relative to total cells (EdU^+^ cells / total cells). Scale bar: 50μm. (E, F) Representative images (E) and quantification (F) of EdU^+^ cells in AGS cells transfected with B17-5p mimics (n = 3). (G, H) Representative images (G) and quantification (H) of EdU^+^ cells in AGS cells after *CERK* siRNA transfection (n = 3). (I, J) Representative images (I) and quantification (J) of EdU^+^ cells in AGS-EBV cells treated with B17-5p inhibitor (n = 3). (K, L) Representative images (K) and quantification (L) of EdU^+^ cells in SNU719 cells treated with B17-5p inhibitor (n = 3).

We next investigated whether miR-BART17-5p-mediated downregulation of CERK contributes to malignant phenotypes. In EBV-negative AGS cells, overexpression of miR-BART17-5p, which suppresses *CERK* expression, as well as siRNA-mediated knockdown of *CERK*, significantly impaired cell proliferation (Fig 3A-3B). Conversely, in EBV-positive AGS-EBV cells, inhibition of endogenous miR-BART17-5p restored *CERK* expression and promoted cell proliferation (Fig 3C). Consistently, in SNU719 cells, inhibition of endogenous miR‑BART17‑5p also enhanced cell proliferation (Fig 3D). These findings were corroborated by EdU incorporation assays, in which suppression of *CERK*, either by B17-5p mimics or *CERK*-specific siRNA, decreased the proportion of EdU-positive cells in AGS cells (Fig 3E-3H), whereas inhibition of miR-BART17-5p increased EdU incorporation in AGS-EBV cells (Fig 3I-3J). Furthermore, in SNU719 cells, inhibition of endogenous miR-BART17-5p significantly increased the percentage of EdU-positive cells, as shown by representative images (Fig 3K) and quantitative analysis (Fig 3L).

**Fig 4:**
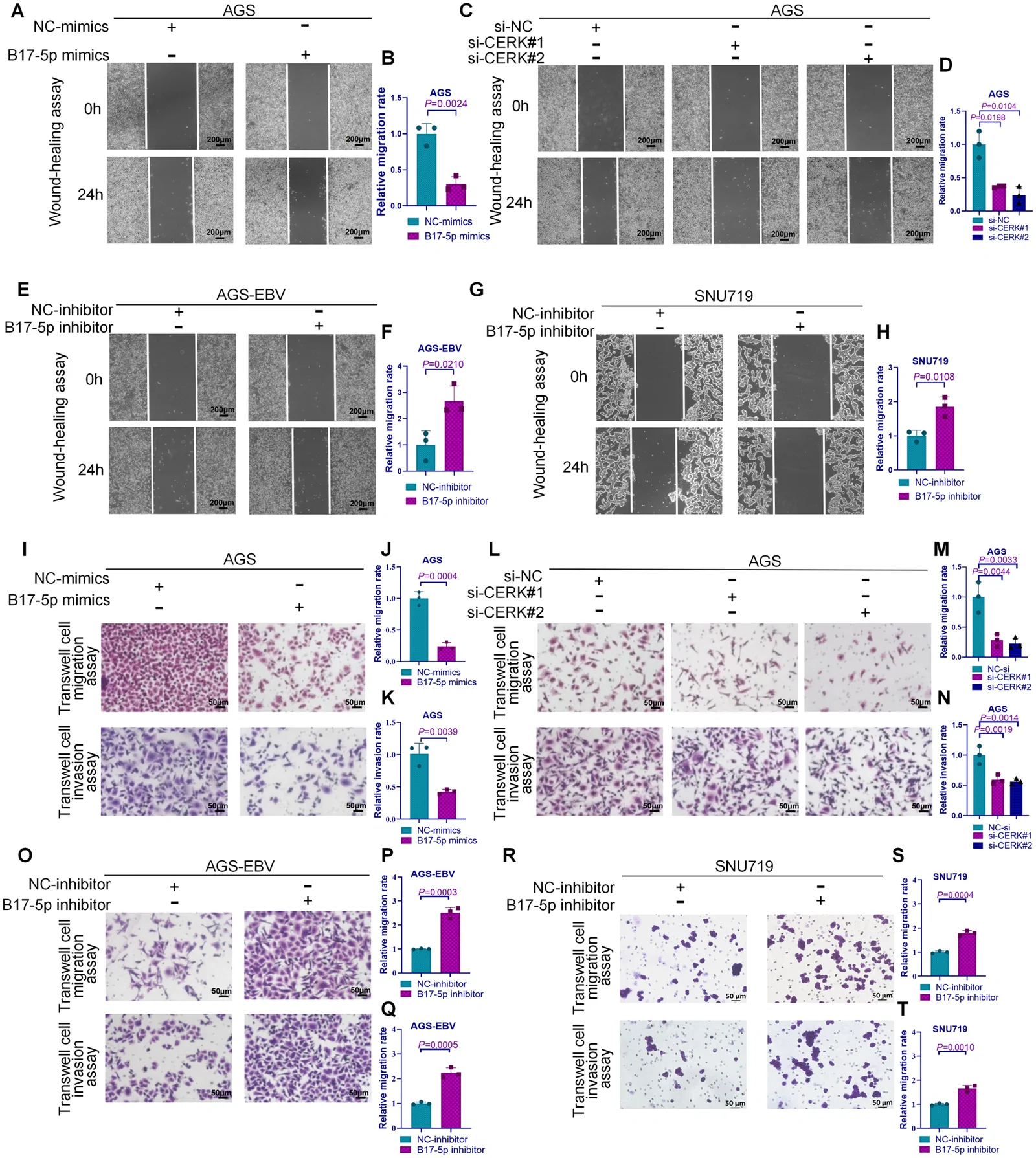
MiR-BART17-5p inhibits GC cell migration and invasion. (A, B) AGS cells were transfected with B17-5p mimics or NC-mimics, wound-healing test in AGS with (A) representative images at 0 h and 24 h (scale bar: 200 μm), (B) quantified by ImageJ (n = 3). (C, D) AGS cells were transfected with *CERK* siRNA (si-CERK#1 and #2) or si-NC, wound-healing assays in AGS with (C) representative images at 0 h and 24 h (scale bar: 200 μm), (D) quantified by ImageJ (n = 3). (E, F) AGS-EBV cells were transfected with B17-5p inhibitor or NC-inhibitor, wound-healing assays in AGS-EBV with (E) representative images at 0 h and 24 h (scale bar: 200 μm), (F) quantified by ImageJ (n = 3). (G, H) SNU719 cells were transfected with B17-5p inhibitor or NC-inhibitor. Wound-healing assay in SNU719 with (G) representative images at 0 h and 24 h (scale bar: 200 μm), (H) quantification by ImageJ (n = 3). (I) Representative images of Transwell migration assays and Tanswell invasion assays (Matrigel-coated) of AGS cells treated with B17-5p mimics or NC-mimics (scale bar: 50 μm). (J, K) Statistical analysis of migrated (J) and invaded (K) cells for AGS cells treated as in (I) (n = 3). (L) Representative images of Transwell migration assays and Transwell invasion assays (Matrigel-coated) of AGS cells treated with *CERK* siRNA (si-CERK#1 and #2) or si-NC (scale bar: 50 μm). (M, N) Statistical analysis of migrated (M) and invaded (N) cells for AGS cells treated as in (L) (n = 3). (O) Representative images of Transwell migration assays and Transwell invasion assays (Matrigel-coated) of AGS-EBV cells treated with B17-5p inhibitor or NC-inhibitor (scale bar: 50 μm). (P, Q) Statistical analysis of migrated (P) and invaded (Q) cells for AGS‑EBV cells treated as in (O) (n = 3). (R) Representative images of Transwell migration and invasion assays of SNU719 cells treated with B17‑5p inhibitor or NC‑inhibitor (scale bar: 50 μm). (S, T) Statistical analysis of migrated (S) and invaded (T) cells for SNU719 cells treated as in (R) (n = 3).

We further examined the effects of the miR-BART17-5p-mediated regulation of *CERK* on cell motility. Wound-healing assays revealed that miR-BART17-5p overexpression or *CERK* knockdown significantly impeded wound closure in AGS cells (Fig 4A-4D). In contrast, inhibition of miR-BART17-5p accelerated wound closure in AGS-EBV and SNU719 cells (Fig 4E-4H). Consistently, Transwell assays demonstrated that suppression of CERK inhibited both migration and invasion of AGS cells (Fig 4I-4N), while inhibition of miR-BART17-5p enhanced migratory and invasive capacities in AGS-EBV and SNU719 cells (Fig 4O-4T).

**Fig 5:**
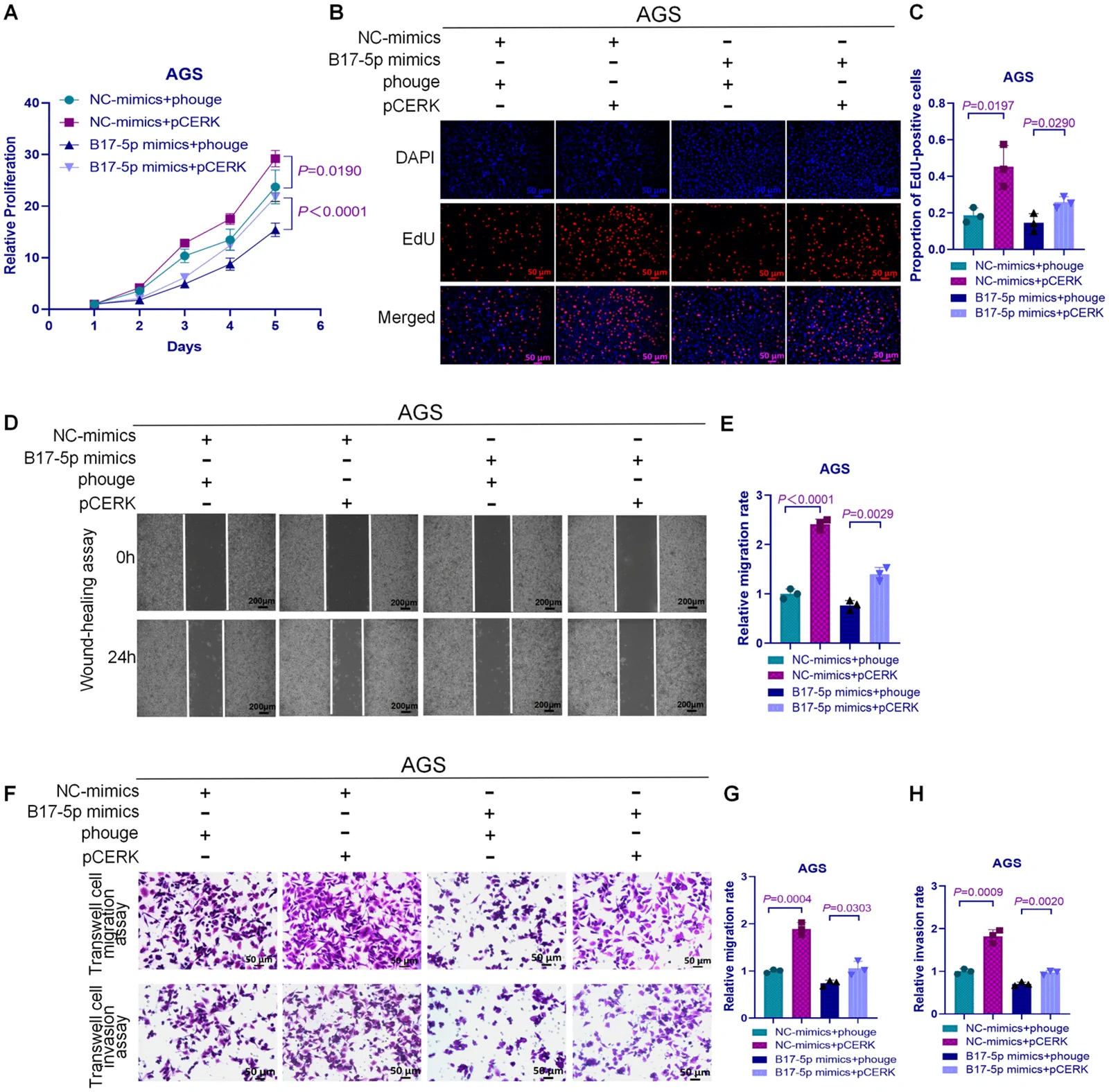
CERK overexpression rescues the tumor-suppressive effects of miR-BART17-5p in GC cells. AGS cells were co‑transfected with either NC‑mimics or B17‑5p mimics together with either empty vector (phouge) or a CERK‑overexpressing vector (pCERK), generating four groups: NC-mimics + phouge, NC-mimics + pCERK, B17-5p mimics + phouge, and B17-5p mimics + pCERK. (A) Cell proliferation assessed by CCK-8 assay at indicated time points (Days 1–5). Absorbance values were normalized to Day 1 (n = 3). (B, C) Cell proliferation assessed by EdU assay. (B) Representative fluorescence images of EdU-positive cells (EdU^+^ cells, red) and total cells (nuclei, blue). Scale bar: 50 μm. (C) Quantification of EdU^+^ cell proportion (EdU^+^cells / total cells), from five random fields per group (n = 3). (D, E) Cell migration assessed by wound-healing assay. (D) Representative images at 0 and 24 h (scale bar: 200 μm). (E) Quantitative analysis of wound closure (n = 3). (F-H) Cell migration and invasion assessed by Transwell assay. (F) Representative images of migrated (uncoated) and invaded (Matrigel-coated) cells. Quantitative analysis of (G) migrated and (H) invaded cells (scale bar: 50 μm, n = 3).

Importantly, the specificity of the miR‑BART17-5p-CERK regulatory relationship was further confirmed by rescue experiments. In AGS cells, four experimental groups were established, including NC-mimics with empty vector (phouge, as described in Methods), NC-mimics with CERK-overexpressing vector (pCERK), B17-5p mimics with phouge, and B17-5p mimics with pCERK. CCK-8 assays demonstrated that CERK overexpression alone significantly promoted cell proliferation compared to the group of NC-mimics with phouge, and this enhancement was also observed when pCERK was co‑transfected with B17‑5p mimics, where the proliferation rate was substantially increased compared to the group of B17-5p mimics with phouge (Fig 5A). EdU incorporation assays confirmed that CERK overexpression increased the percentage of EdU-positive cells, and co-expression of CERK restored EdU incorporation reduced by B17‑5p mimics (representative images in Fig 5B; quantification in Fig 5C). Similarly, wound-healing assays showed that CERK overexpression enhanced cell migration, and this migratory capacity was restored upon co-transfection of pCERK with mimics (representative images in Fig 5D; quantification in Fig 5E). Transwell migration and invasion assays further supported these observations: CERK overexpression increased both migratory and invasive abilities, and co-expression of CERK effectively reversed the suppressive effects of miR‑BART17‑5p mimics (representative images in Fig 5F; migration quantification in Fig 5G; invasion quantification in Fig 5H). Collectively, these rescue experiments confirm that miR‑BART17‑5p exerts its tumor‑suppressive functions primarily through targeting *CERK*.

### MiR-BART17-5p suppresses GC progression partially via the CERK/PI3K/AKT pathway

**Fig 6:**
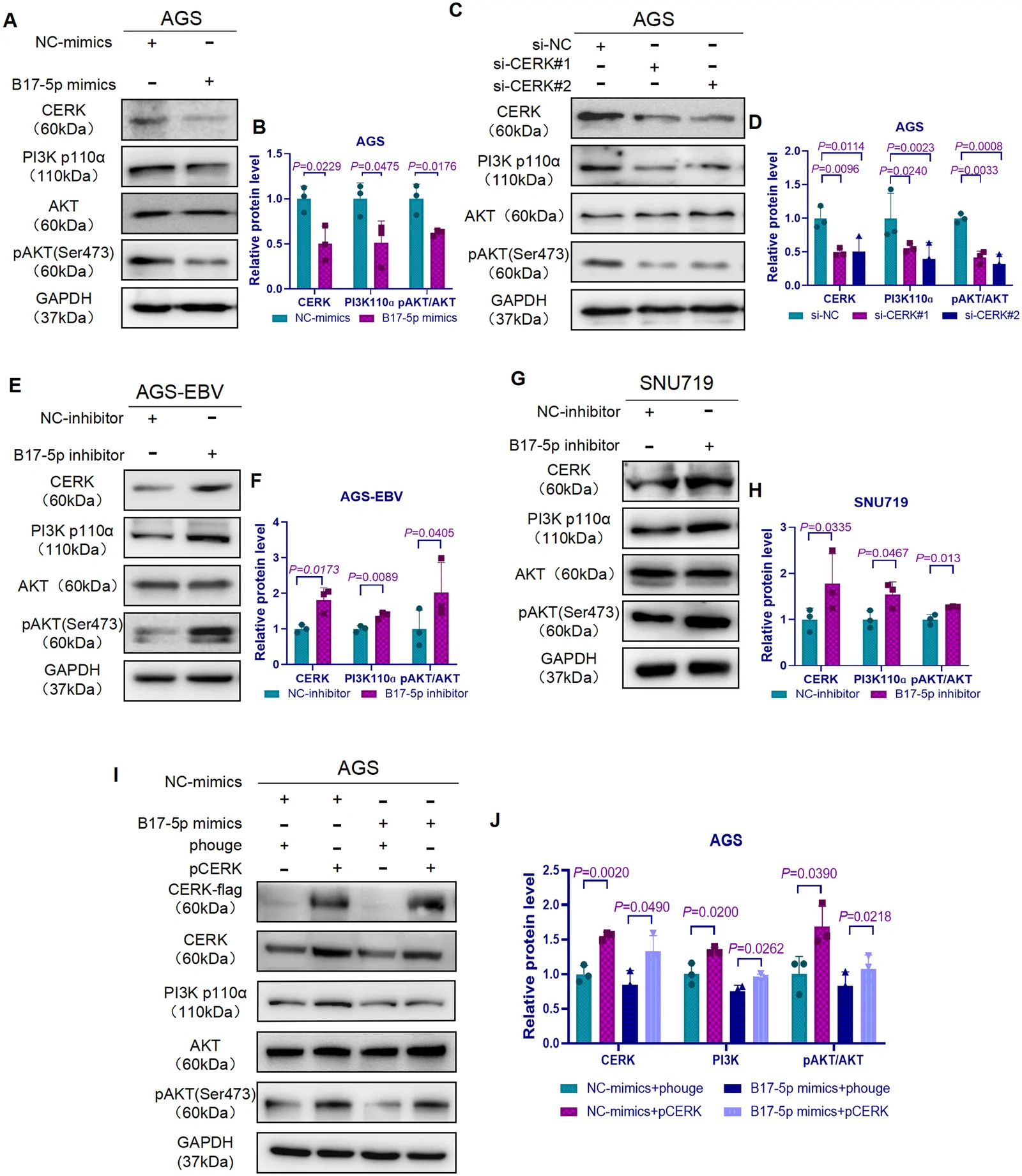
MiR-BART17-5p suppresses the PI3K/AKT signaling pathway by targeting *CERK.* (A, B) Western blotting analysis of CERK, PI3K p110α, phosphorylated AKT (p-AKT), and total AKT in AGS cells transfected with B17-5p mimics or NC-mimics. Representative blots (A) and quantification normalized to GAPDH (B) are shown (n = 3). (C, D) Western blotting analysis of the indicated proteins in AGS cells transfected with *CERK* siRNA (si-CERK#1 and #2) or si-NC. Representative blots (C) and quantification normalized to GAPDH (D) are shown (n = 3). (E, F) Western blotting analysis of the indicated proteins in AGS-EBV cells transfected with B17-5p inhibitor or NC-inhibitor. Representative blots (E) and quantification normalized to GAPDH (F) are shown (n = 3). (G, H) Western blotting analysis of the indicated proteins in SNU719 cells transfected with B17-5p inhibitor or NC-inhibitor. Representative blots (G) and quantification normalized to GAPDH (H) are shown (n = 3). (I, J) Rescue of PI3K/AKT signaling by CERK overexpression. Western blotting analysis of the indicated proteins in AGS cells co‑transfected with either NC‑mimics or B17‑5p mimics together with either empty vector (phouge) or CERK‑overexpressing vector (pCERK), resulting in four groups as indicated. Representative blots (I) and quantification normalized to GAPDH (J) are shown (n = 3).

To elucidate the downstream mechanism by which miR-BART17-5p inhibits GC progression, we examined its effects on the PI3K/AKT signaling pathway, a key pathway downstream of CERK. Overexpression of miR-BART17-5p in AGS cells markedly reduced CERK protein levels, accompanied by decreased expression of PI3K p110α and reduced AKT phosphorylation (Fig 6A-6B). A similar attenuation of PI3K/AKT signaling was observed following siRNA-mediated knockdown of CERK (Fig 6C-6D). Conversely, inhibition of endogenous miR-BART17-5p in AGS-EBV or SNU719 cells restored CERK expression and concomitantly enhanced PI3K/AKT signaling activity (Fig 6E-6H). Furthermore, overexpression of CERK alone increased PI3K p110α and p‑AKT levels, whereas co‑expression of CERK with miR‑BART17‑5p mimics rescued the suppressed PI3K/AKT activity (Fig 6I‑6J), confirming CERK as the central mediator of miR-BART17-5p-dependent regulation of PI3K/AKT signaling.

**Fig 7:**
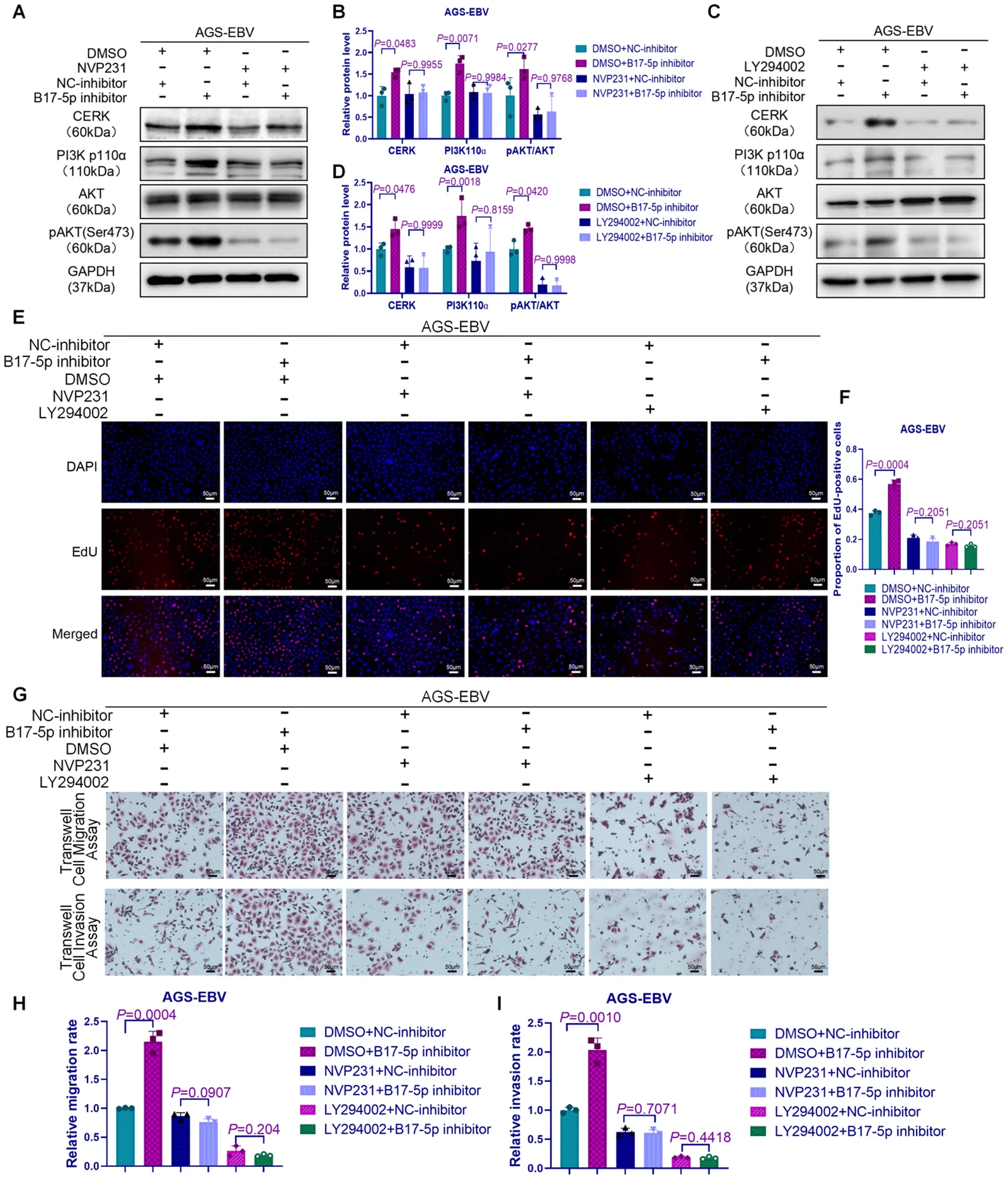
Pharmacological inhibition of CERK or PI3K partially reverses the tumor-suppressive effects of miR-BART17-5p. (A-D) Inhibition of the PI3K/AKT pathway by specific inhibitors. Western blotting analysis of phosphorylated AKT (p-AKT) and total AKT in AGS cells transfected with B17-5p mimics and treated with either the CERK inhibitor NVP231 (800 nM) (A, B) or the PI3K inhibitor LY294002 (40 μM) (C, D). Representative blots (A, C) and quantification normalized to GAPDH (B, D) are shown (n = 3). (E, F) Rescue of cell proliferation. Proliferation of AGS cells treated as indicated was assessed by CCK-8 assay (E) and EdU assay (F) (n = 3). (G-I) Rescue of cell migration and invasion. (G) Representative images of migrated (uncoated) and invaded (Matrigel-coated) cells (scale bar: 50 μm, n = 3). (H, I) Quantitative analysis of migrated and invaded cells.

To further establish the functional relevance of PI3K/AKT signaling in this context, we used pharmacological inhibitors. Treatment with the CERK inhibitor NVP231 (800 nM) or the PI3K inhibitor LY294002 (40 μM) effectively inhibited PI3K/AKT signaling (Fig 7A-7D). Importantly, both inhibitors significantly reversed the anti-proliferative (Fig 7E-7F) and anti-migratory effects of miR-BART17-5p (Fig 7G-7I), suggesting that suppression of GC cell growth and motility by miR-BART17-5p occurs at least in part through CERK-dependent PI3K/AKT signaling.

### MiR-BART17-5p inhibits the growth of GC xenografts *in vivo* by targeting the CERK/AKT signaling pathway

**Fig 8:**
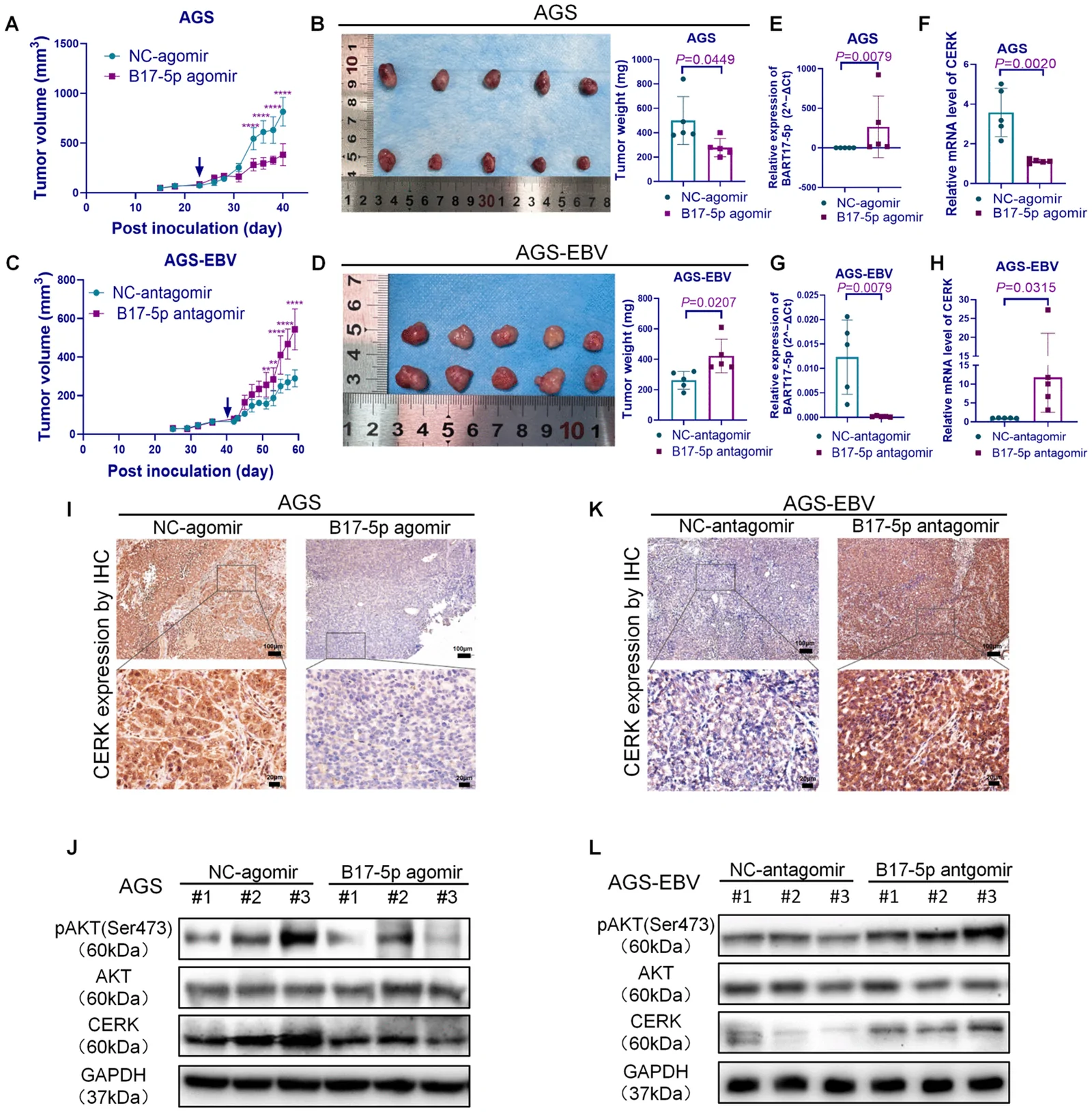
MiR-BART17-5p inhibits the growth of GC xenografts *in vivo* by targeting the CERK/AKT signaling pathway. (A-D) Therapeutic effects of agomir and antagomir in xenograft models. (A, B) Growth curves (A) and final tumor weights (B) of AGS xenografts following intratumoral injection of cholesterol-conjugated miR-BART17-5p agomir (abbreviated as B17-5p agomir) or NC-agomir (n = 5). (C, D) Growth curves (C) and final tumor weights (D) of AGS-EBV xenografts treated with miR-BART17-5p antagomir (abbreviated as B17-5p antagomir) or NC-antagomir (n = 5). (E-H) *In vivo* validation of on-target effects at the mRNA level. RT-qPCR analysis of miR-BART17-5p and *CERK* mRNA levels in (E, F) AGS tumors from the agomir-treated group and (G, H) AGS-EBV tumors from the antagomir-treated group (n = 5). (I-L) *In vivo* validation at the protein and signaling level. (I, J) Representative Western blotting (I) and immunohistochemistry (IHC) images (J) showing reduced levels of CERK and phosphorylated AKT (p-AKT, Ser473) in agomir-treated AGS tumors. (K, L) Representative Western blotting (K) and IHC images (L) showing restored levels of CERK and p-AKT in antagomir-treated AGS-EBV tumors. For IHC, scale bars: 100 μm and 20 μm.

To validate our *in vitro* findings in an *in vivo* context, we evaluated the effects of miR-BART17-5p in xenograft mouse models using cholesterol-conjugated agomir and antagomir. AGS cells were subcutaneously injected into NSG mice to establish tumors of similar size, followed by intratumoral injection of miR-BART17-5p agomir (5 nmol). Compared with the negative control, agomir treatment markedly suppressed the growth of AGS xenografts (Fig 8A-8B). In contrast, intratumoral injection of miR-BART17-5p antagomir significantly promoted the growth of AGS-EBV xenografts (Fig 8C-8D).

*In vivo* molecular analyses further validated the regulatory role of miR-BART17-5p on CERK. The RT-qPCR results confirmed efficient delivery and on-target activity of the agomir, as evidenced by elevated miR-BART17-5p levels accompanied by reduced *CERK* mRNA expression in agomir-treated AGS tumors (Fig 8E-8F). Conversely, antagomir-treated AGS-EBV tumors exhibited decreased miR-BART17-5p expression and concomitant upregulation of *CERK* mRNA (Fig 8G-8H). Consistent changes were observed at the protein level. Agomir treatment reduced CERK protein expression and AKT phosphorylation at Ser473, as shown by Western blotting and IHC analyses (Fig 8I-8J), whereas antagomir treatment restored CERK expression and increased AKT phosphorylation (Fig 8K-8L). Together, these data indicate that modulation of miR-BART17-5p levels *in vivo* directly regulates CERK-mediated AKT activation and significantly impacts tumor growth.

## Discussion

CERK is a lipid kinase that phosphorylates ceramide to generate C1P, a bioactive sphingolipid involved in the regulation of diverse cellular processes, including cell growth, survival, and migration [18]. CERK has been reported as an oncogenic driver in multiple cancer types, contributing to tumor progression, therapeutic resistance, and poor prognosis, often through activation of the PI3K/AKT signaling pathway [12,14,17,27]. However, the clinical relevance of CERK in GC remains unexplored. Here, analysis of the Kaplan-Meier database revealed that elevated *CERK* mRNA expression is significantly associated with poor prognosis in GC patients. Functionally, we demonstrated that CERK knockdown inhibited the malignant phenotypes in AGS cells, including proliferation, migration, and invasion, through inhibition of the PI3K/AKT pathway. These findings are consistent with the established oncogenic role of CERK in other malignancies. Furthermore, through integrated bioinformatics analyses and experimental validation in GC tissues and cell models, we identified EBV infection as a negative regulator of CERK expression, uncovering a previously unrecognized layer of viral modulation of this oncogenic kinase.

EBV is a ubiquitous human oncovirus that is etiologically associated with a wide spectrum of diseases, including multiple malignancies and autoimmune disorders [28–31]. Its pathogenic potential is mediated by a complex repertoire of viral gene products, among which EBV-encoded miRNAs have emerged as critical regulators of the host cellular environment. These miRNAs exhibit remarkable functional diversity, exerting both oncogenic and tumor-suppressive effects depending on the host targets they regulate. For instance, miR-BART1-3p promotes gastric tumor growth by enhancing exosome-mediated oncogenic signaling, with USP37 and MACC1 identified as downstream targets involved in cell proliferation [32]. In contrast, miR-BART20-3p suppresses proliferation and migration in EBV-positive gastric cancer by inhibiting *PPARα*(23). Similarly, miR-BART16 negatively regulates cell proliferation by modulating the expression of the viral oncoprotein LMP1 [33,34], while miR-BART15-3p promotes cell apoptosis by targeting the anti-apoptotic protein BRUCE [33,34]. Beyond their cell-autonomous effects, EBV miRNAs also modulate host immune surveillance and the viral life cycle. For example, miR-BART2 facilitates immune evasion by targeting the stress-induced ligand MICB [35] and auto-regulates the viral life cycle by inhibiting viral DNA polymerase [36]. This functional diversity underscores the complexity and non-monolithic nature of the EBV-encoded miRNA network.

We acknowledge that a recent study reported that miR‑BART17‑5p promotes gastric cancer cell migration by targeting *KLF2* [37], and a pro‑tumor role has also been described in nasopharyngeal carcinoma [38]. Several factors may explain this apparent discrepancy. First, like many miRNAs, miR-BART17-5p has multiple targets, and its net effect likely depends on which target is functionally dominant in a given cellular context. In our setting, *CERK* is a key target, leading to PI3K/AKT inhibition and tumor suppression, whereas *KLF2* or *ARID1A* may predominate in other studies. Importantly, miRNA-mediated regulation typically results in partial, rather than complete, suppression of individual targets, consistent with a fine-tuning mode of action within complex regulatory networks. Therefore, the moderate downregulation of CERK observed in our study is biologically expected and does not diminish its functional significance. Second, differences in experimental conditions (e.g., cell passage, transfection reagents, or mimic/inhibitor design) could influence phenotypic outcomes. Importantly, our conclusions are supported by multiple functional assays, rescue experiments, and in vivo models, consistently demonstrating a tumor‑suppressive role of miR‑BART17‑5p in our system. In this study, we identify miR-BART17-5p as a viral factor that directly targets the host gene *CERK*, thereby inhibiting the PI3K/AKT pathway and tumor progression. This finding is consistent with the established paradigm that viral miRNAs regulate host signaling pathways, while providing a specific mechanistic example of how EBV fine-tunes oncogenic signaling through host lipid kinase regulation.

However, in the CERK rescue experiments, re-expression of CERK partially restored PI3K/AKT signaling and cellular phenotypes in the presence of miR-BART17-5p, but did not fully recapitulate control levels. This difference can be explained by the reduced effective CERK dosage under miR-BART17-5p expression, where endogenous CERK is suppressed and signaling relies primarily on exogenous CERK, whereas both endogenous and exogenous CERK contribute to total protein abundance in the control condition. Consequently, exogenous CERK alone only partially restores downstream PI3K/AKT activity and cellular phenotypes. This suggests that CERK is a key functional mediator of miR-BART17-5p, while additional miR-BART17-5p targets may also contribute to the observed phenotypic effects.

The divergent phenotypic outcomes of EBV-encoded miRNAs, which range from oncogenic to tumor-suppressive, are attributable to the distinct host gene networks they regulate. Our findings elucidate a mechanism by which miR-BART17-5p targets *CERK,* demonstrating how a single EBV miRNA can exert a defined regulatory effect within the complex landscape of virus-host interactions. The presence of tumor-suppressive viral miRNAs may reflect an evolutionary strategy that enables long-term viral persistence within the host. By fine-tuning the expression of specific host oncogenes, EBV may modulate the tumor microenvironment to avoid excessive cellular proliferation that could trigger immune surveillance or compromise viral latency, thereby fostering a cellular state conducive to sustained infection.

It is necessary to point out that while EBVaGC cell lines such as SNU719 and AGS-EBV are widely used models for mechanistic studies of EBVaGC biology, viral gene and miRNA expression levels in these *in vitro* systems do not fully reflect those observed in primary tumors. In contrast, *in vivo* xenograft tumors show markedly reduced miR-BART17-5p levels compared with cultured cells and are more similar to those observed in primary EBVaGC tissues, indicating that the tumor microenvironment restores a more physiologically relevant expression context. Notably, even under this lower expression level, modulation of miR-BART17-5p still significantly affects tumor growth, supporting the biological relevance of the miR-BART17-5p-CERK/PI3K-AKT regulatory axis in both in *vitro* and in *vivo* settings.

Targeting *CERK* with the viral miRNA miR-BART17-5p represents a potential therapeutic strategy in preclinical models. Our *in vivo* studies provide direct evidence supporting the feasibility of this approach. In xenograft models, administration of a cholesterol-conjugated miR-BART17-5p agomir effectively suppressed tumor growth, with molecular analyses confirming the intended downregulation of both CERK and phospho-AKT in the treated tumors. The identification of *CERK* as a direct target of the tumor-suppressive miR-BART17-5p opens a new avenue for therapeutic intervention. This miRNA-based strategy marks a significant shift in strategy from conventional small-molecule CERK inhibitors, such as NVP231 [39,40]. Unlike kinase inhibitors, a synthetic miR-BART17-5p agomir suppresses *CERK* through highly sequence-specific RNA interference, potentially minimizing the off-target effects. Furthermore, by acting at the mRNA level, this approach complements strategies that target the enzymatic activity of the already synthesized proteins. Crucially, this strategy leverages a naturally evolved viral regulatory circuit as its therapeutic blueprint. By enhancing this endogenous pathway through agomir delivery, it aims to restore a physiological brake on oncogenic signaling, offering superior biological compatibility compared with introducing entirely foreign chemical inhibitors. Notably, the agomir formulation used in this study has been granted a Chinese invention patent (Patent No. ZL202510014577.6).

The feasibility of employing exogenous miRNAs for cancer therapy is supported by an expanding body of preclinical evidence [41–45]. Exploratory studies have investigated the therapeutic application of non-human miRNAs. For example, the plant-derived miRNA osa-miR164d, delivered via ginger-derived exosome-like nanoparticles, was shown to target the TAB1 gene in macrophages, thereby alleviating intestinal inflammation [46]. This finding demonstrates the feasibility of cross-kingdom gene regulation as a therapeutic strategy. In parallel, the feasibility of delivering synthetic miRNAs to gastric tumors has been experimentally demonstrated. For example, a PAMAM/miR-144 nanocarrier system effectively inhibited gastric cancer cell migration by targeting the mTOR pathway, while lipid nanoparticle (LNP) -delivered siRNAs achieved efficient gene silencing in gastric cancer models [47]. Collectively, these studies underscore the therapeutic potential of nucleic acid-based interventions and establish critical technical precedents for delivery optimization.

To translate the miR-BART17-5p-based strategy toward clinical application, future studies must prioritize the optimization of agomir delivery. Advanced delivery platforms, such as LNPs, will be essential for improving molecular stability and enabling precise tissue targeting [48–54]. In the context of GC therapy, further refinement of delivery systems is required beyond conventional nanoparticle platforms. Future strategies may incorporate active targeting ligands to enhance selective delivery to GC cells. For instance, the AS1411 aptamer leverages the differential expression of surface nucleolin, which is abundant on GC cells but minimally expressed on normal cells, thereby enabling selective tumor accumulation of therapeutic agents [55,56]. Additionally, engineering carriers with mucoadhesive or mucus-penetrating properties are crucial for overcoming the gastric mucosal barrier and ensuring effective local delivery [57]. Moreover, the selection or design of highly biocompatible vectors, such as biomimetic bacterial membrane vesicles, is vital for minimizing potential immunogenicity concerns, particularly when non-human miRNA sequences are employed [58]. Robust protection of the nucleic acid payload against the degradative gastric environment is also necessary, particularly for oral administration strategies [59]. Finally, optimized delivery systems must undergo rigorous evaluations of efficacy and safety in patient-derived organoids and immunocompetent animal models. Future studies can build upon these considerations to refine miR-BART17-5p delivery, thereby enhancing therapeutic efficacy and further validating *in vivo* safety. Given that miR-BART17-5p has also been reported to exhibit functional roles in other EBV-associated malignancies, including nasopharyngeal carcinoma, it is plausible that similar regulatory effects on CERK-dependent signaling may also occur in additional tumor contexts. However, this requires further investigation, as the functional outcome of miR-BART17-5p is likely to be context-dependent.

In summary, this study reveals a previously unrecognized EBV-host regulatory mechanism in which EBV-encoded miR-BART17-5p directly targets *CERK*, leading to inhibition of the PI3K/AKT signaling and consequent suppression of tumor cell proliferation, migration, and invasion. Notably, significant tumor regression was achieved *in vivo* through the administration of the miR-BART17-5p agomir, confirming the therapeutic potential of this viral miRNA. Taken together, these findings not only expand the functional spectrum of EBV-encoded miRNAs but also establish a novel therapeutic strategy that harnesses pathogen-derived regulatory molecules to target critical oncogenic pathways.

## Supporting information

S1 Table Sequences of plasmids and oligonucleotides. (XLSX)

S2 Table Primer sequences for RT-qPCR assay. (XLSX)

S3 Table Antibody conditions used in experiments. (XLSX)

S4 Table Minimal data set. (XLSX)

S1 File Raw gel. (PDF)
