# Supplementary material for: Epstein-Barr virus-encoded microRNA-BART17-5p suppresses cell proliferation and metastasis by targeting CERK
Source: PLoS Pathog. 2026 Sep 15;22(9):e1014519. doi: 10.1371/journal.ppat.1014519 (PMC13577566; doi:10.1371/journal.ppat.1014519)

These images correspond to Figure 1D

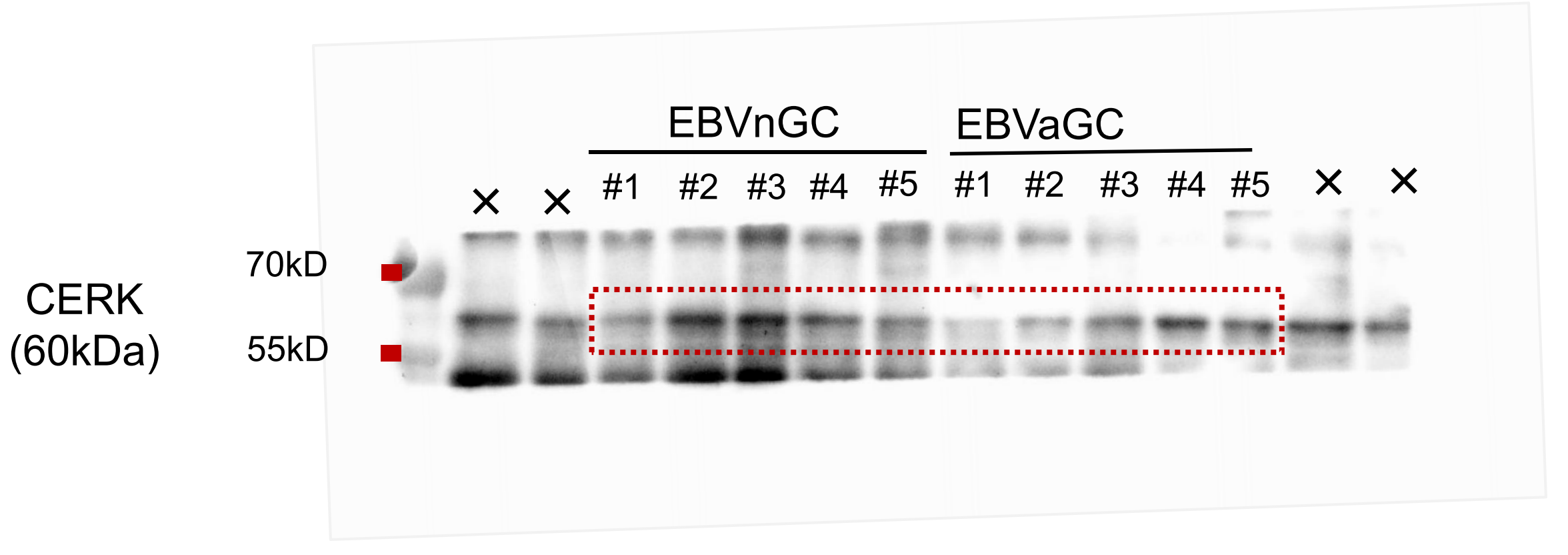

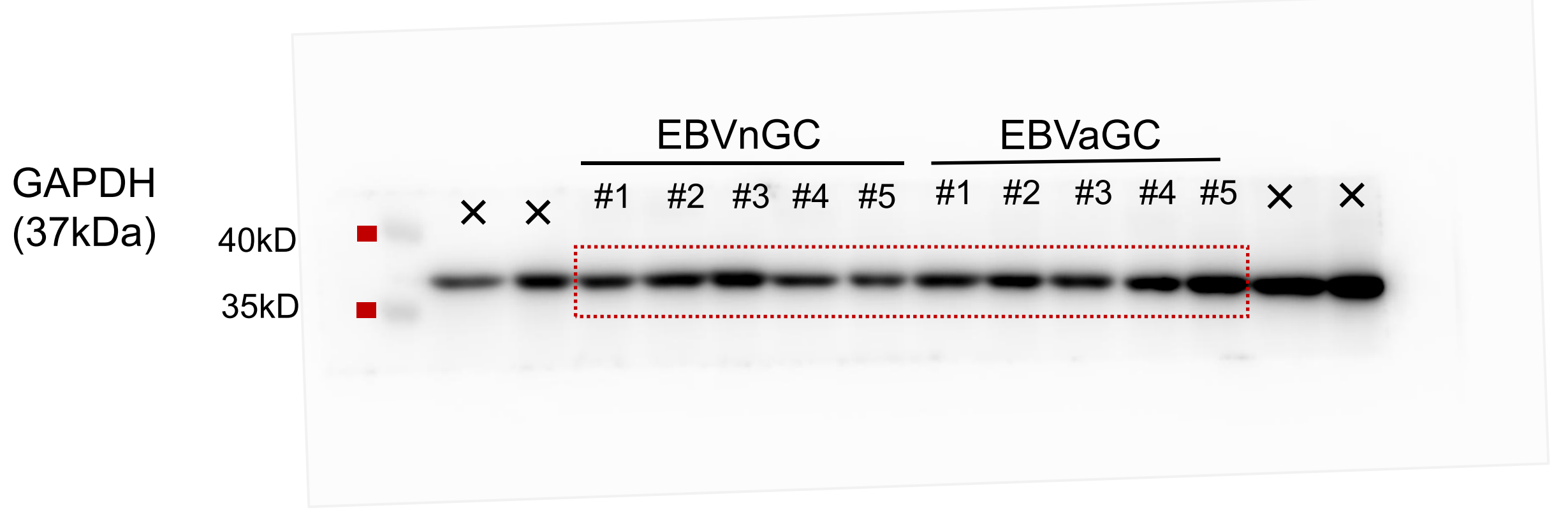

This image corresponds to Figure 1D

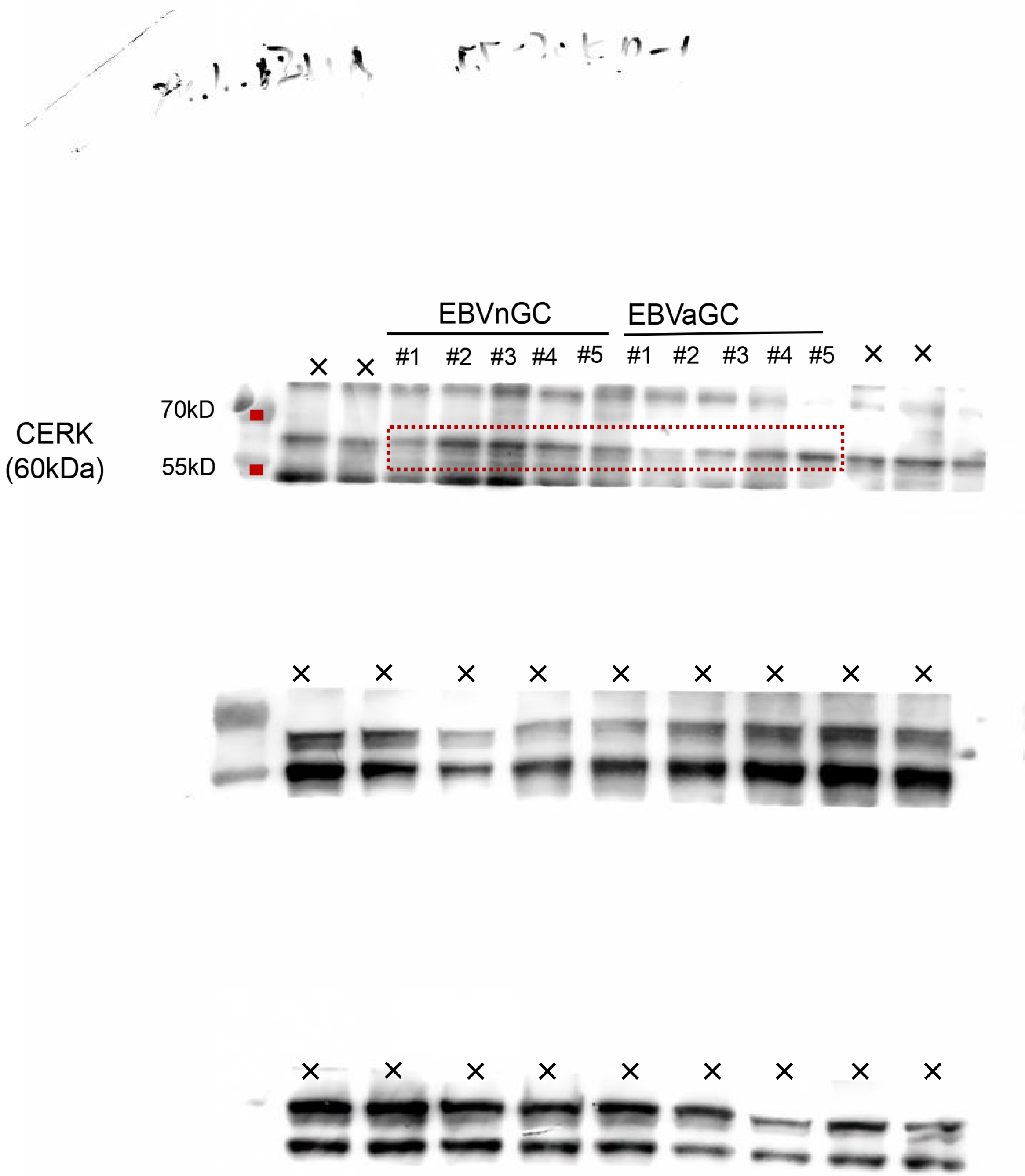

This image corresponds to Figure 1D

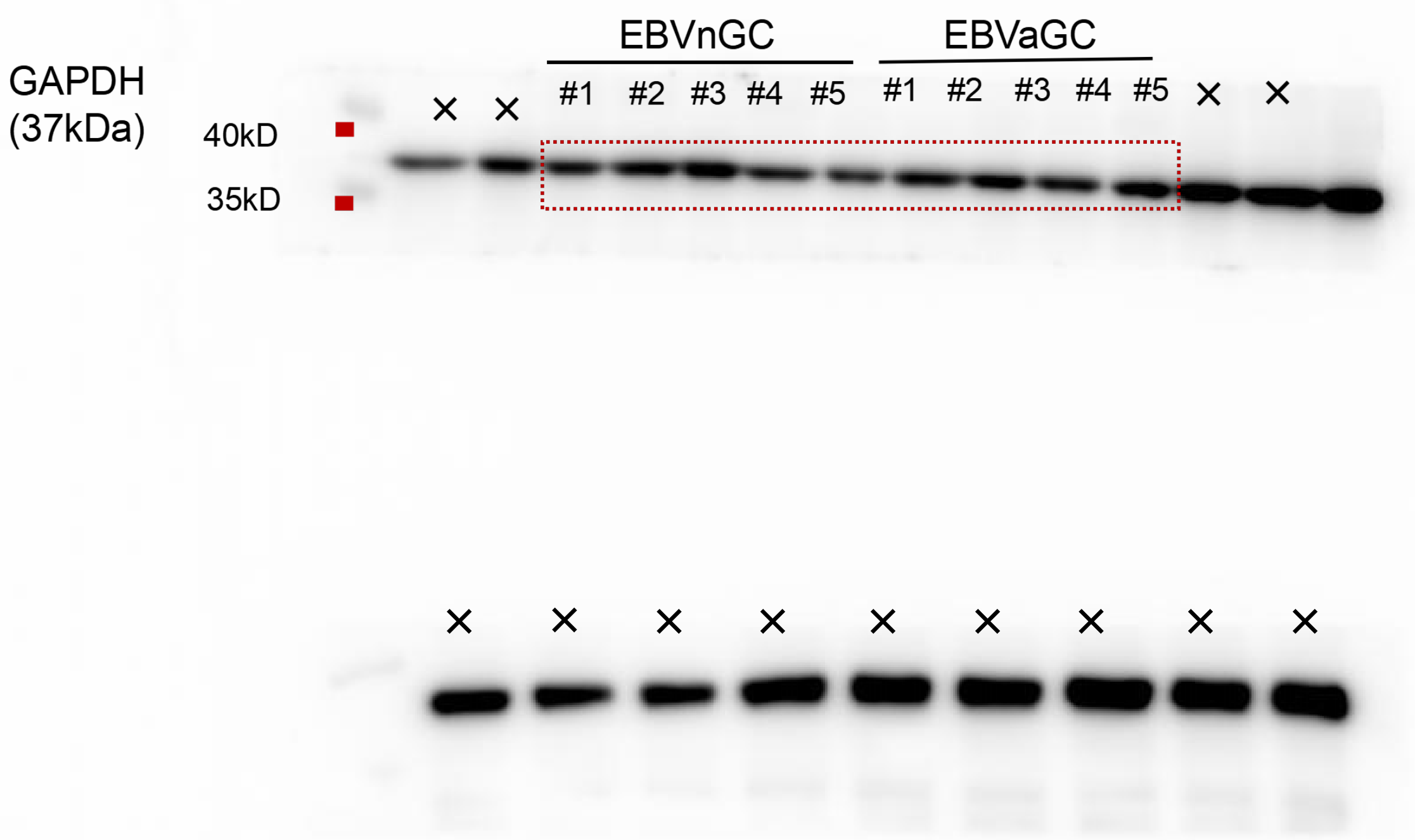

These images correspond to Figure 1J

Biological Replicate #1

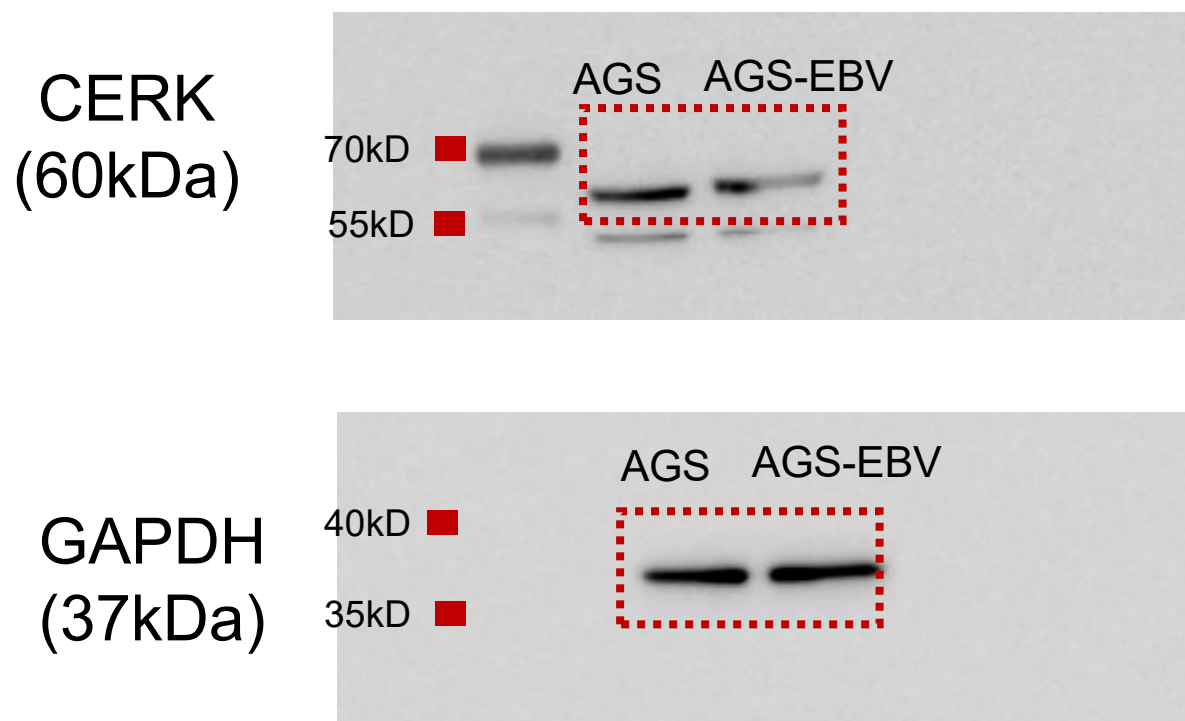

Biological Replicate #2

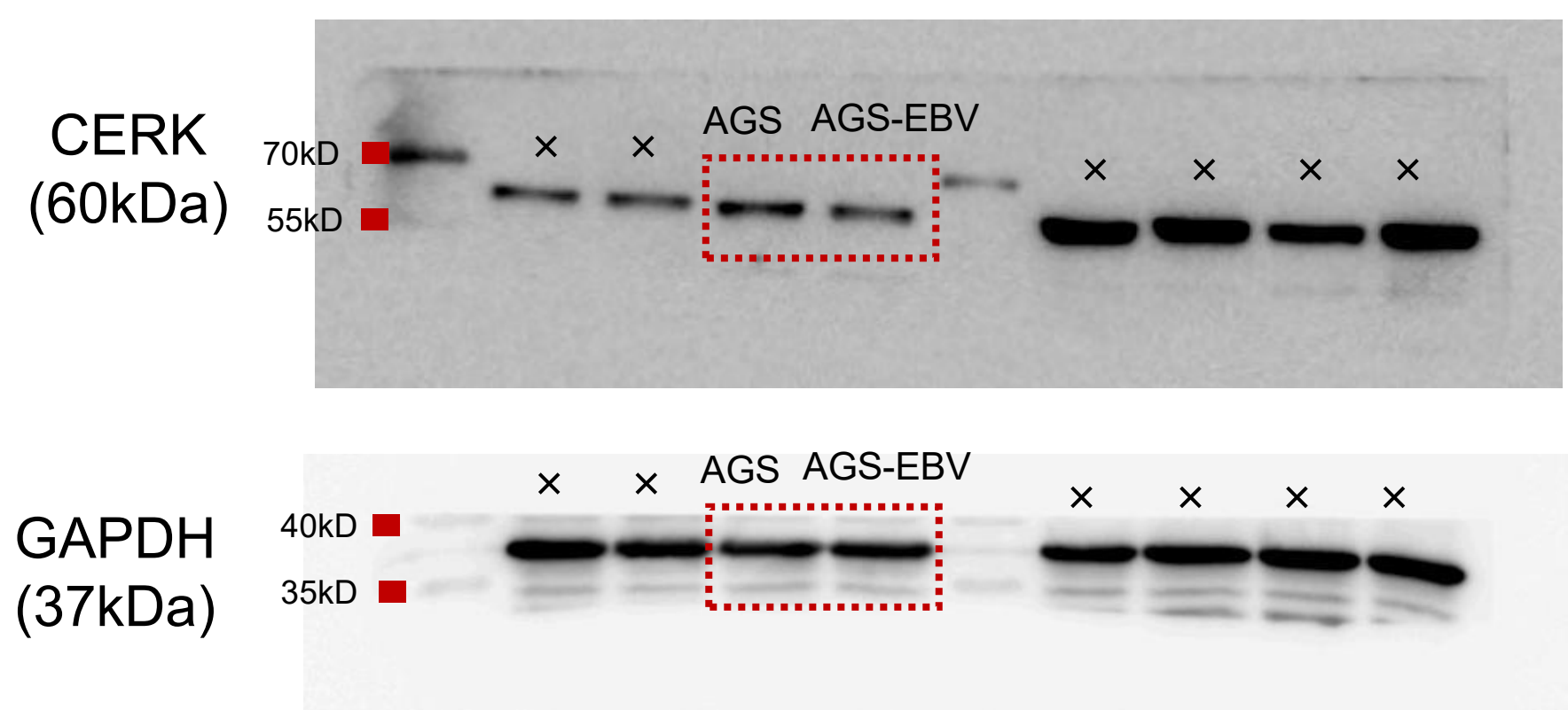

Biological Replicate #3

▲ These images was selected as the representative result in Figure 1J.

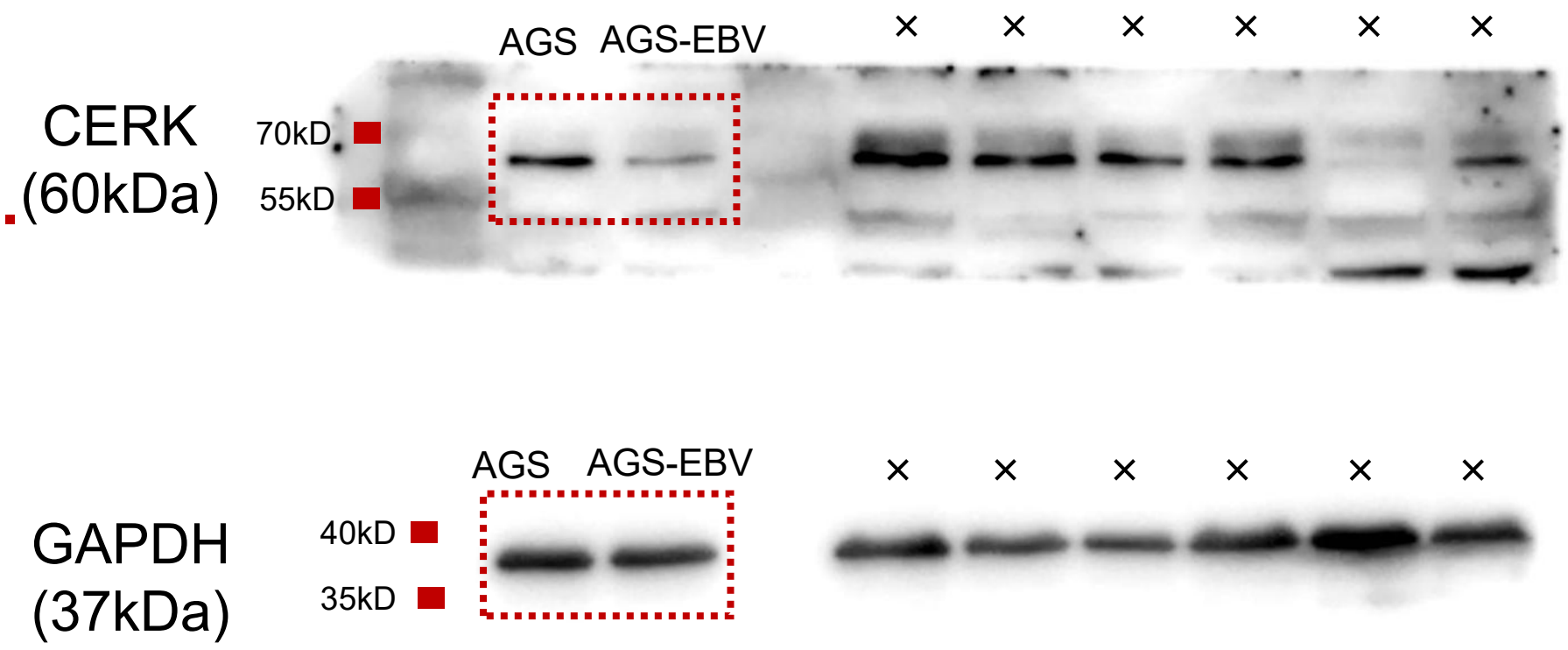

This image corresponds to Figure 1J

BiologicalReplicate#1

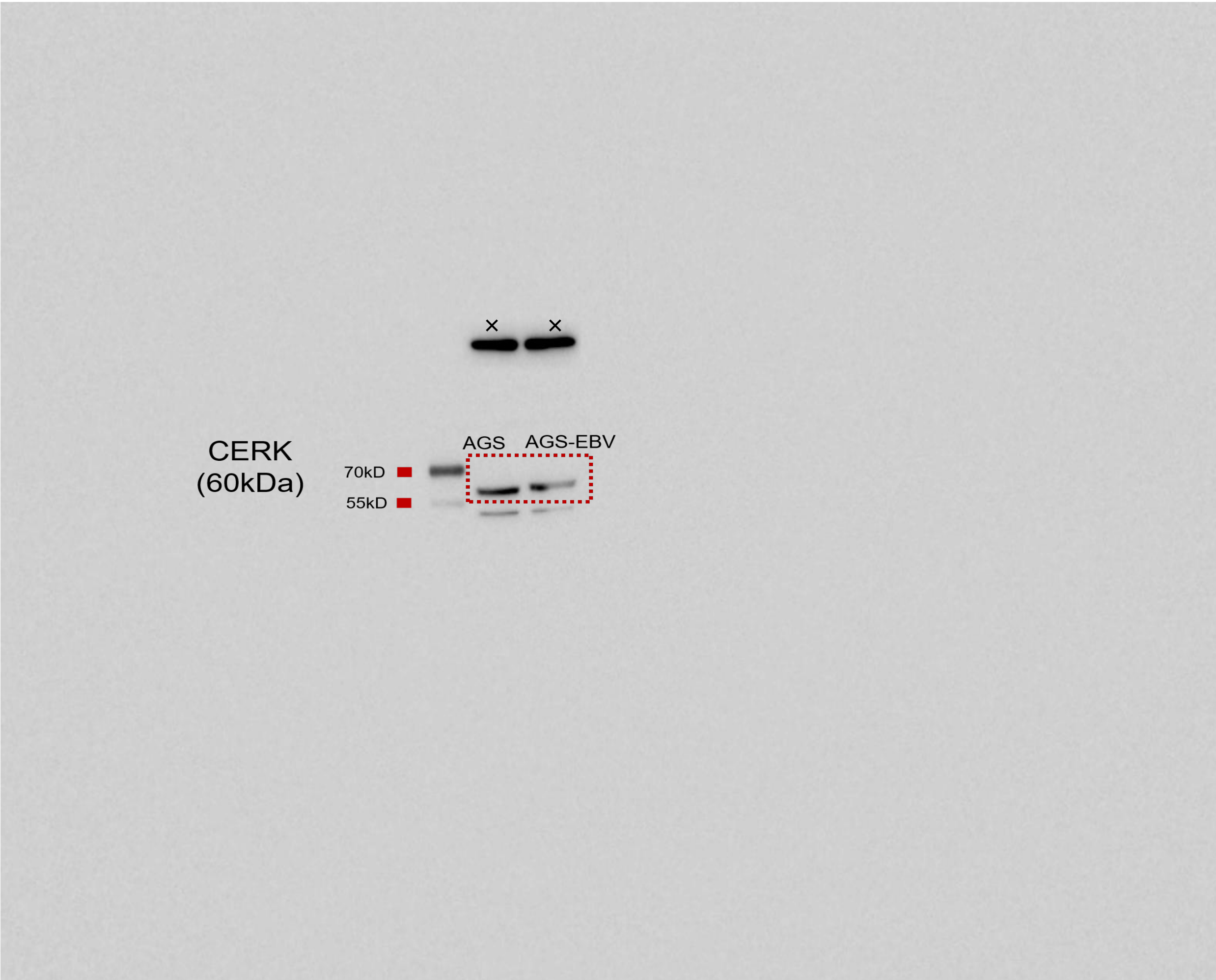

This image corresponds to Figure 1J

BiologicalReplicate#1

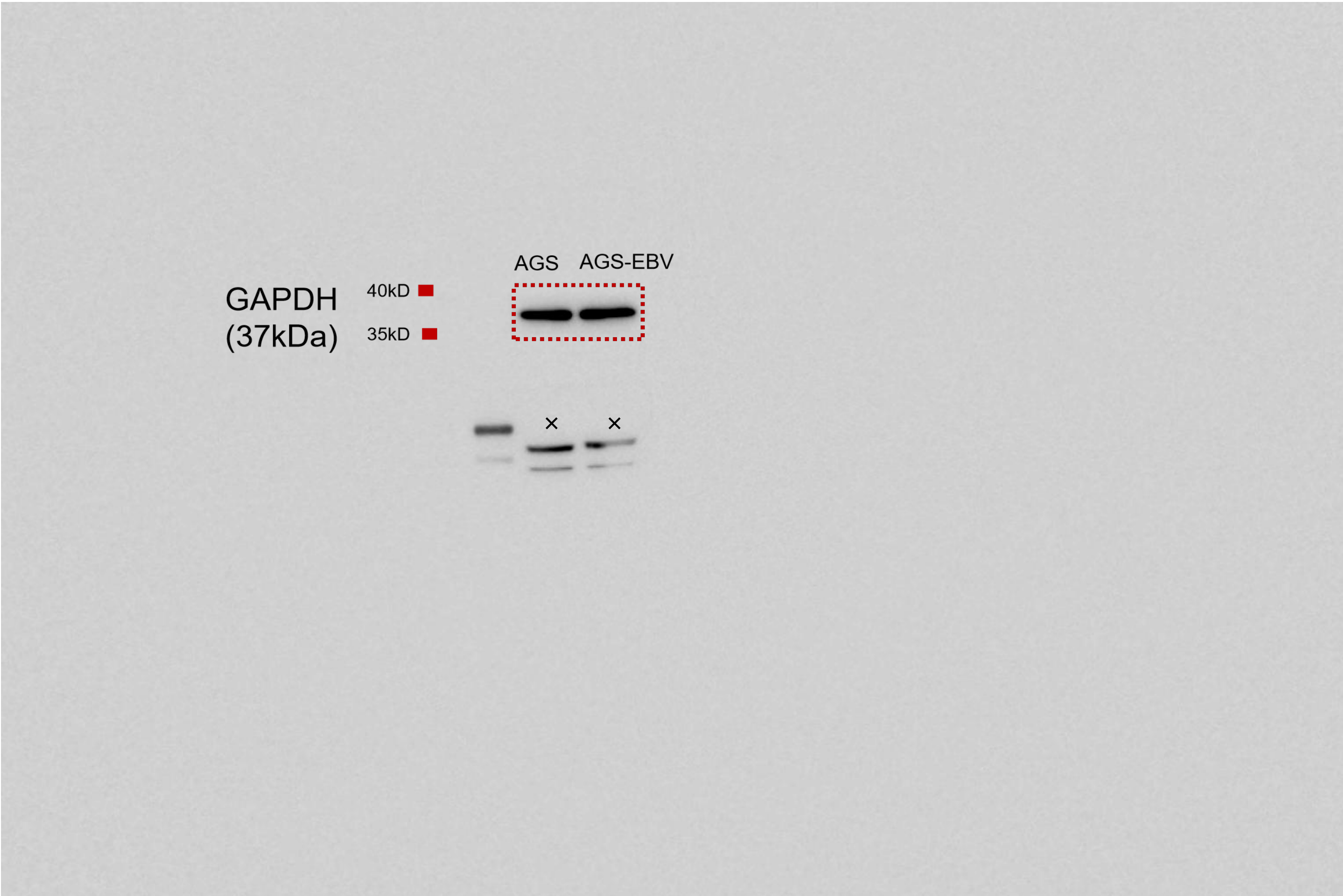

This image corresponds to Figure 1J

Biological Replicate #2

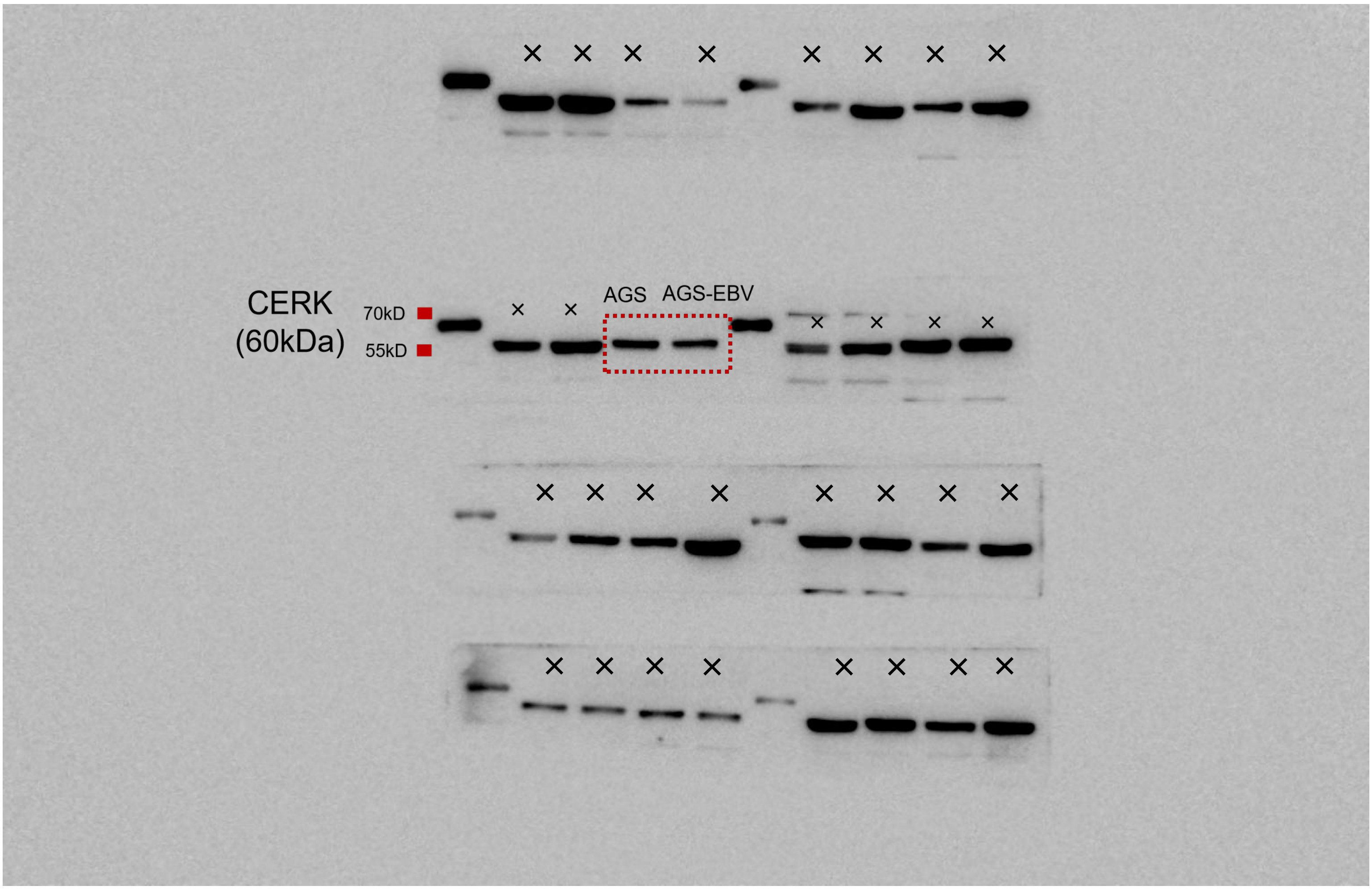

This image corresponds to Figure 1J

Biological Replicate #2

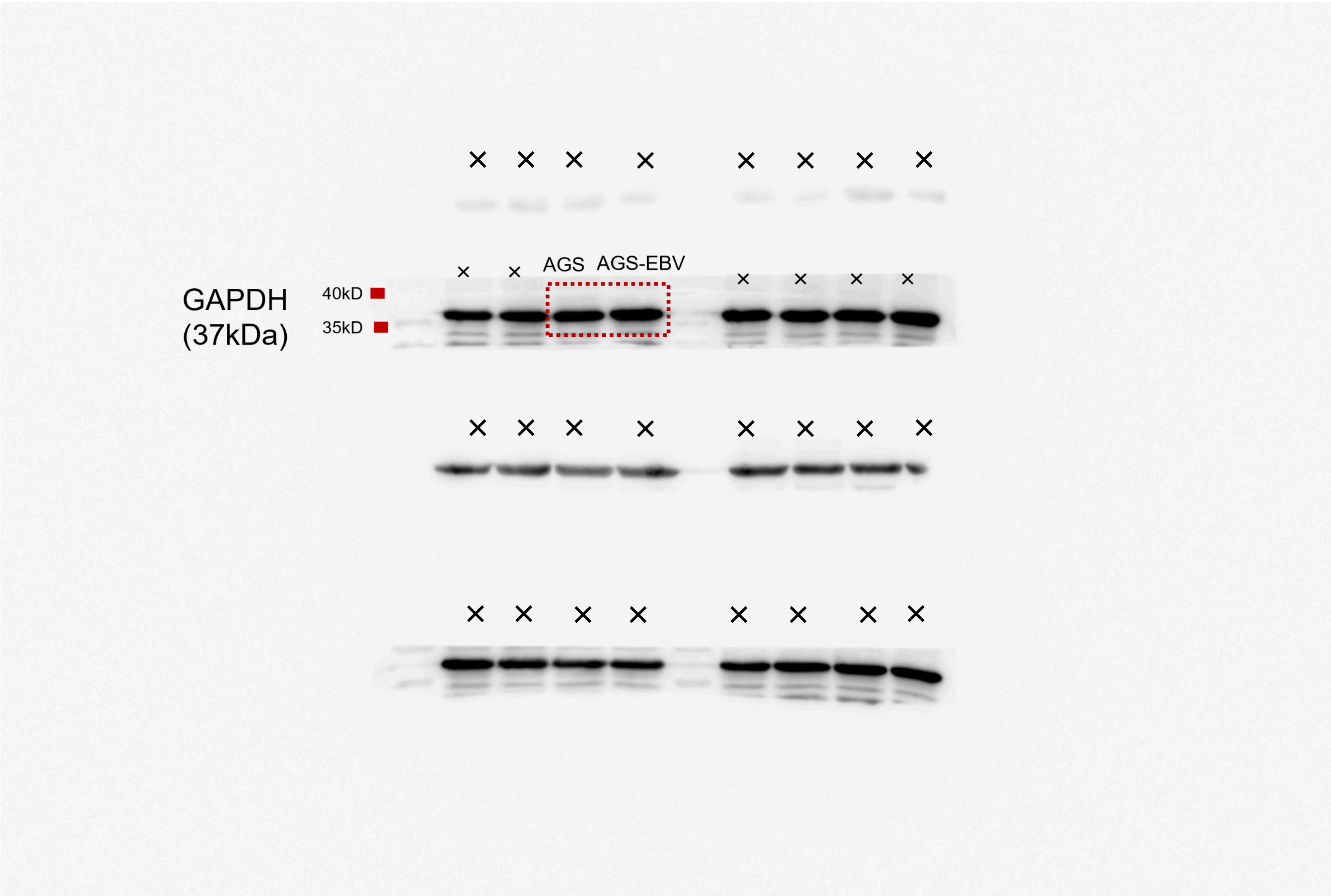

This image corresponds to Figure 1J

BiologicalReplicate#3

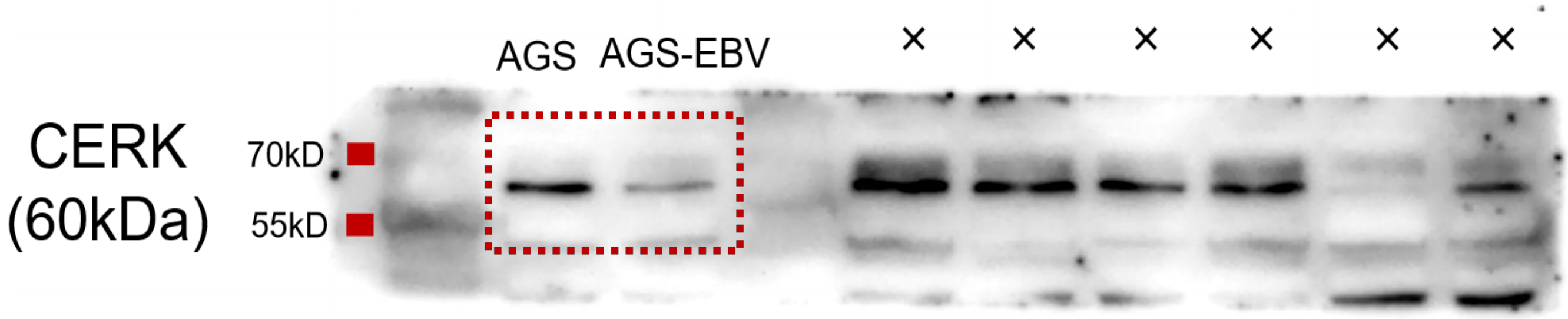

This image corresponds to Figure 1J

BiologicalReplicate#3

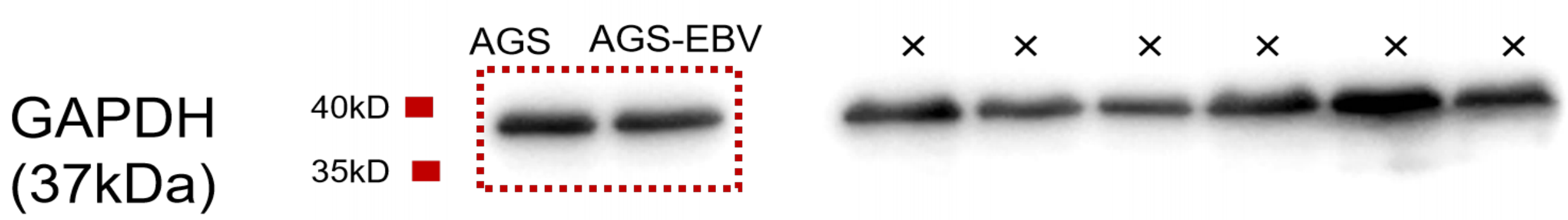

These images correspond to Figure 2 H

Biological Replicate #1

▲ These images was selected as the representative result in Figure 2H.

CERK (60kDa)

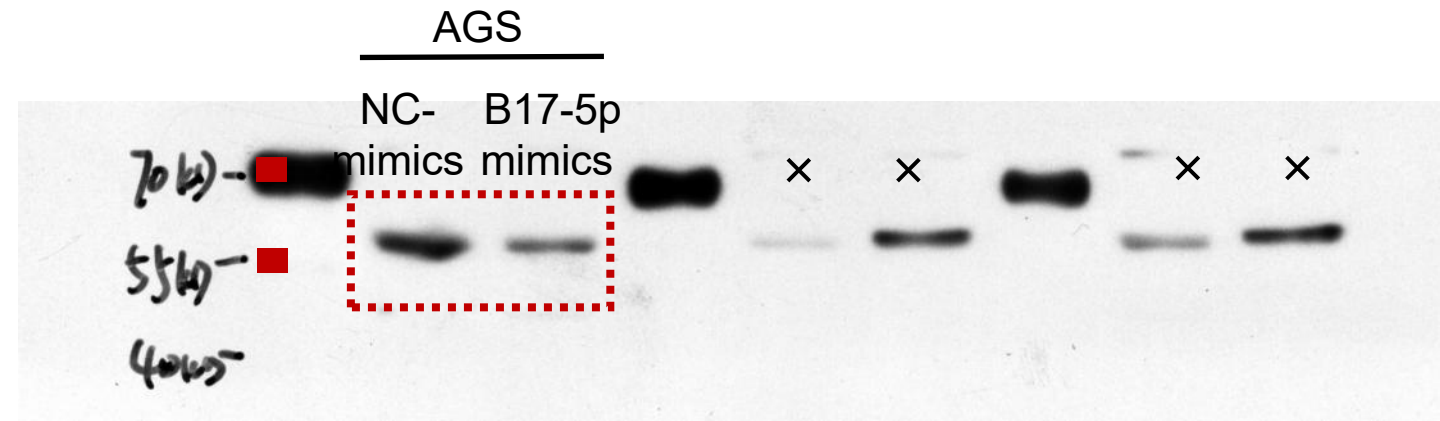

GAPDH (37kDa)

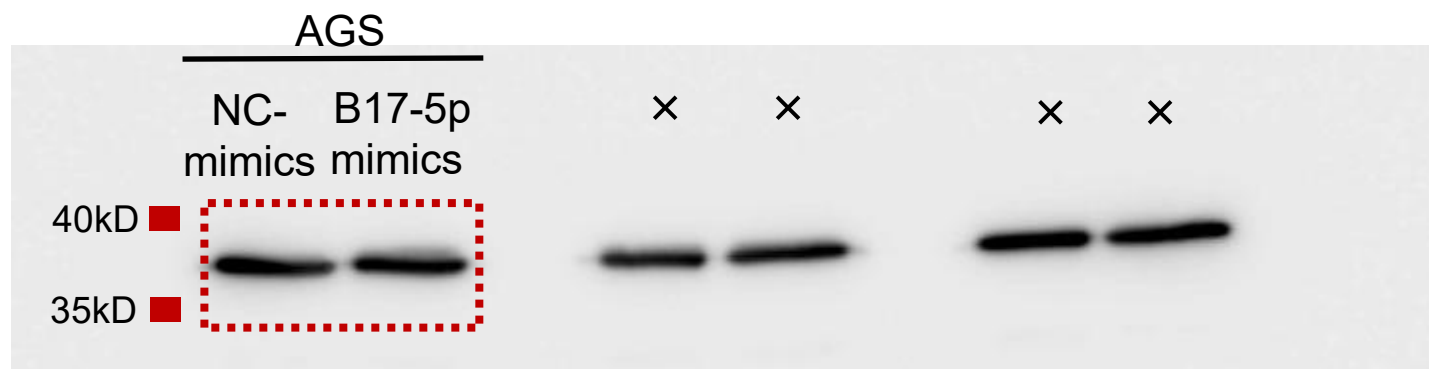

Biological Replicate #2

CERK (60kDa)

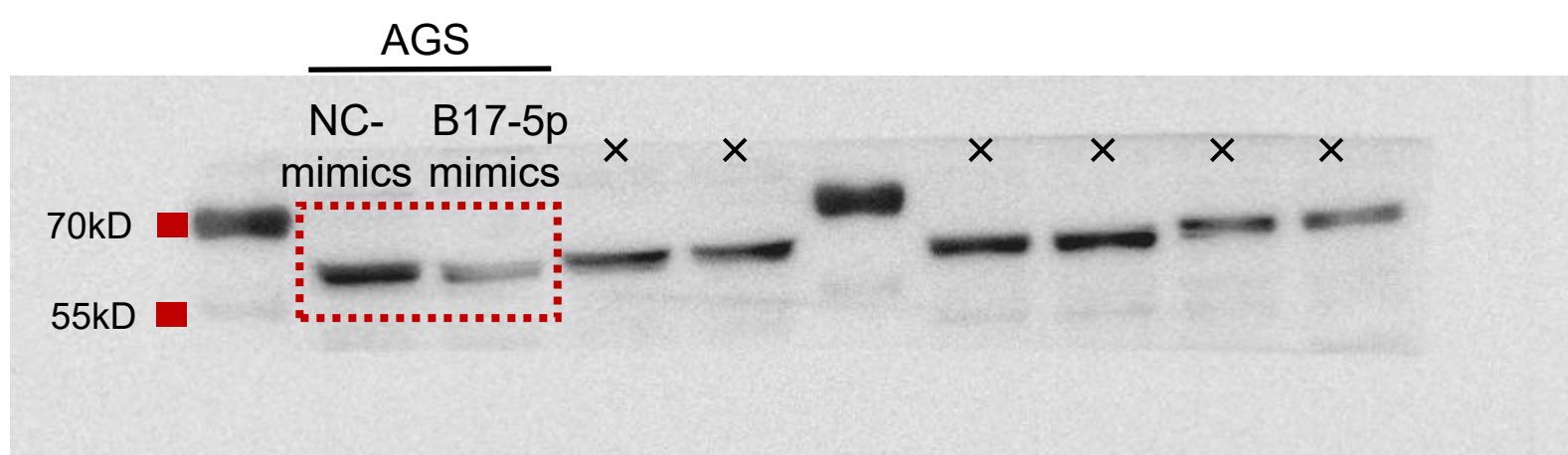

GAPDH (37kDa)

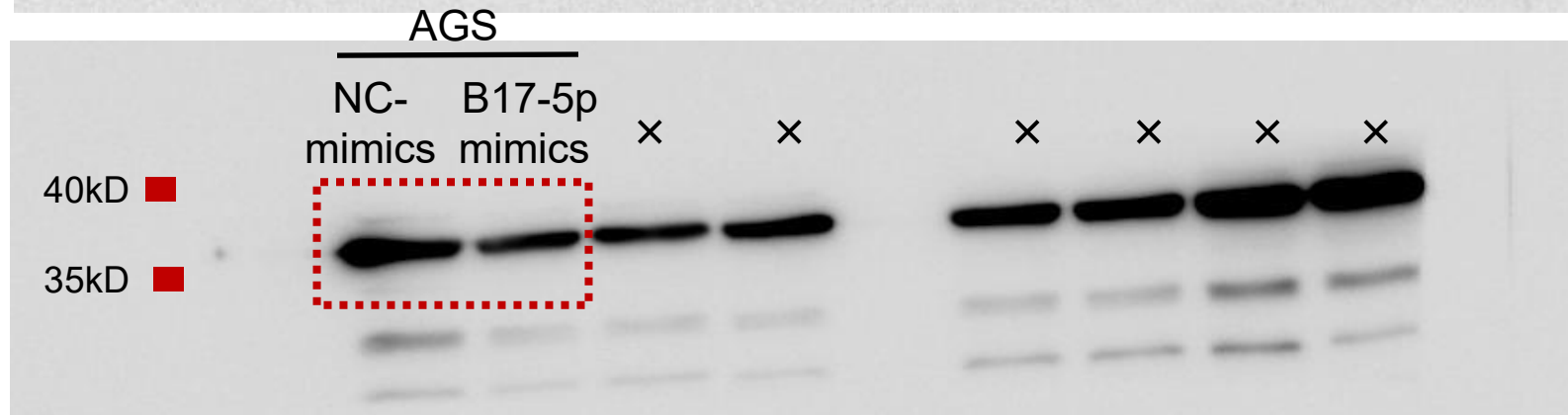

Biological Replicate #3

CERK (60kDa)

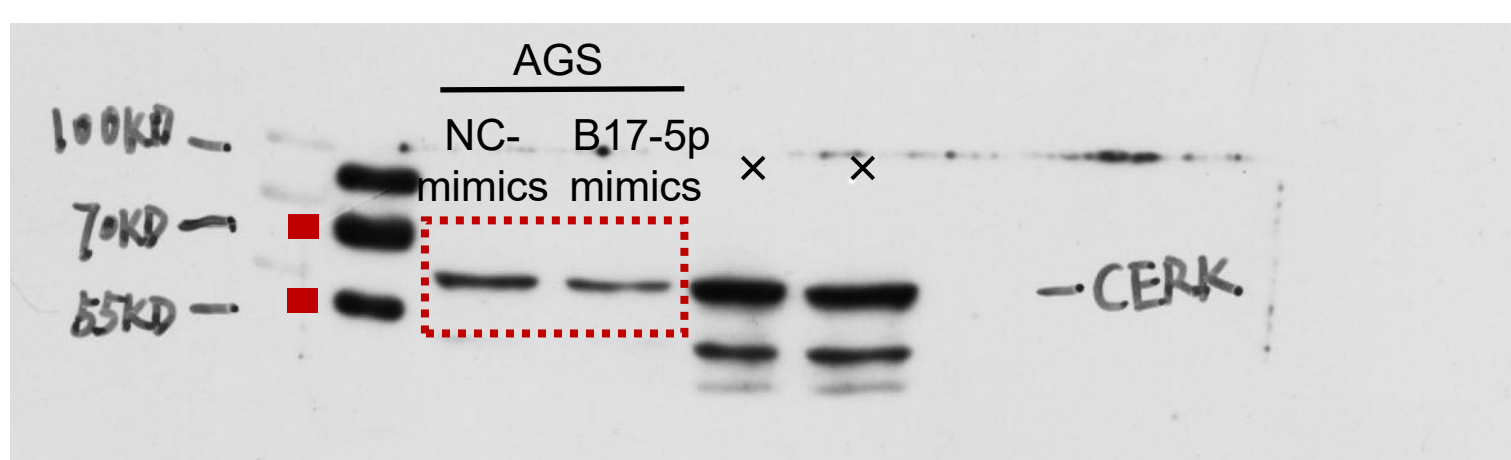

GAPDH (37kDa)

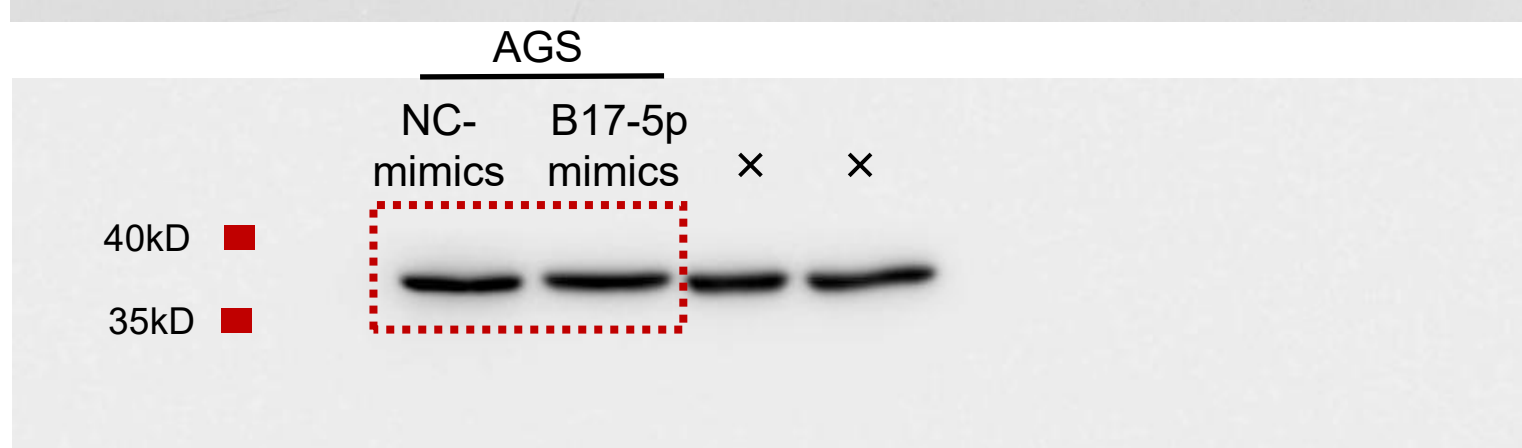

This image corresponds to Figure 2 H

Biological Replicate #1

CERK
(60kDa)

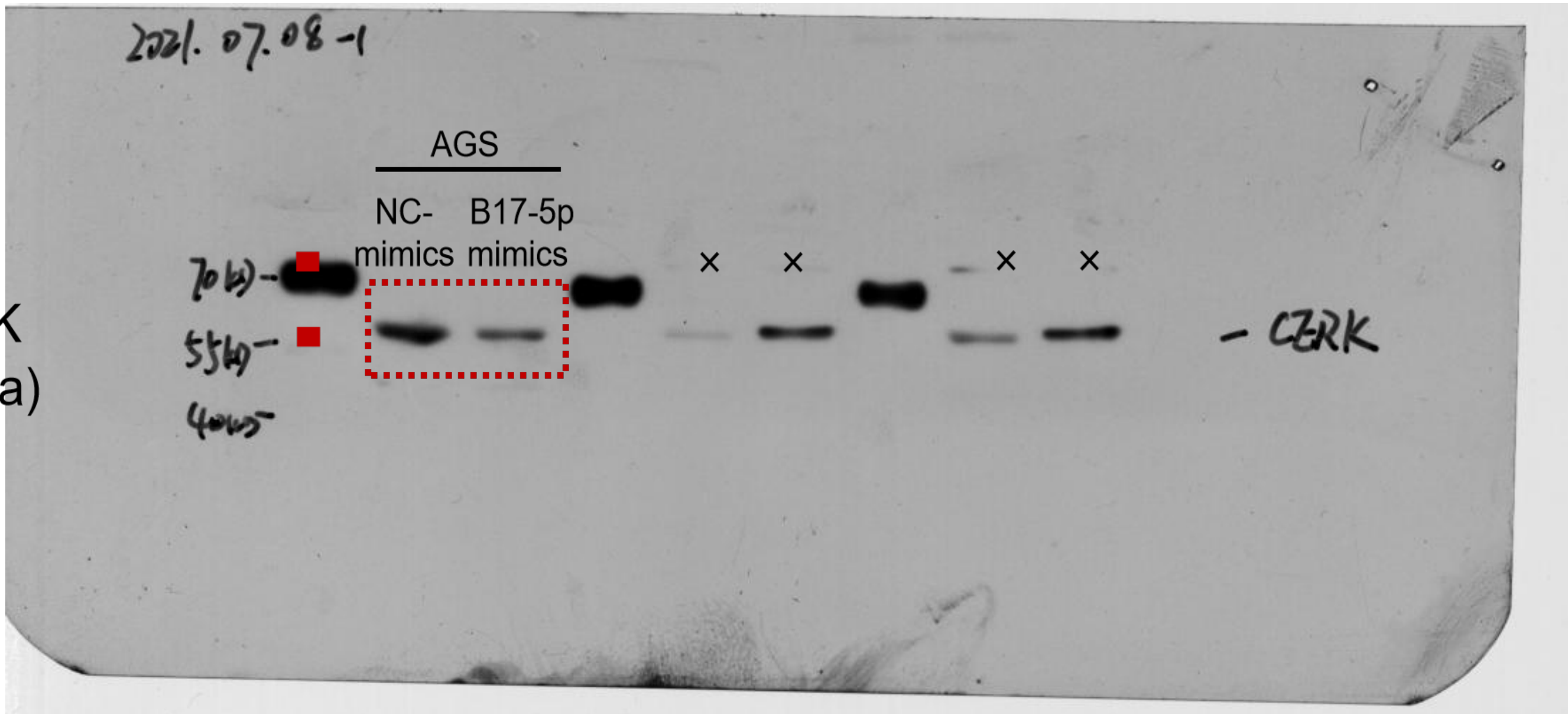

This image corresponds to Figure 2 H

Biological Replicate #1

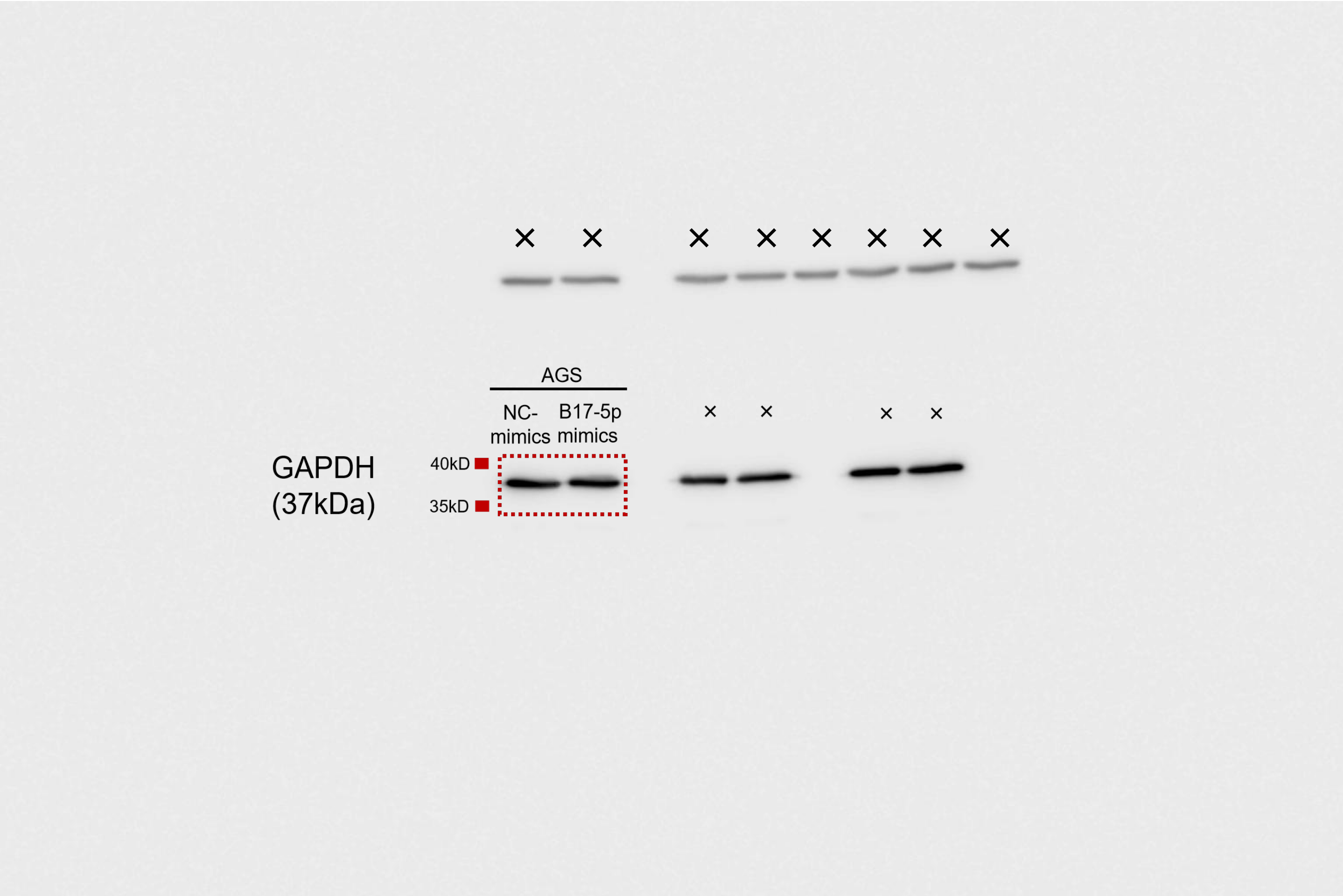

This image corresponds to Figure 2 H

Biological Replicate #2

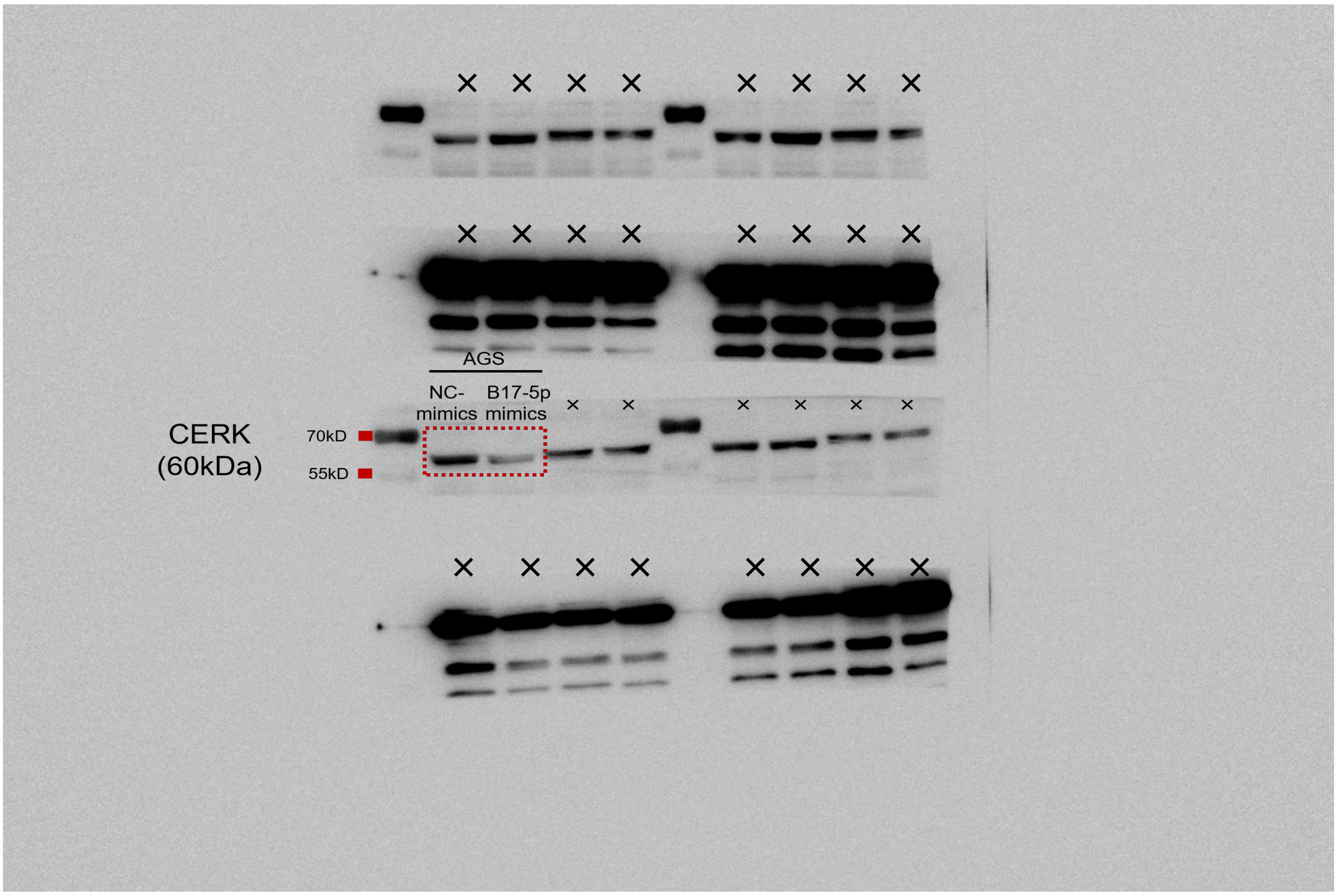

This image corresponds to Figure 2 H

Biological Replicate #2

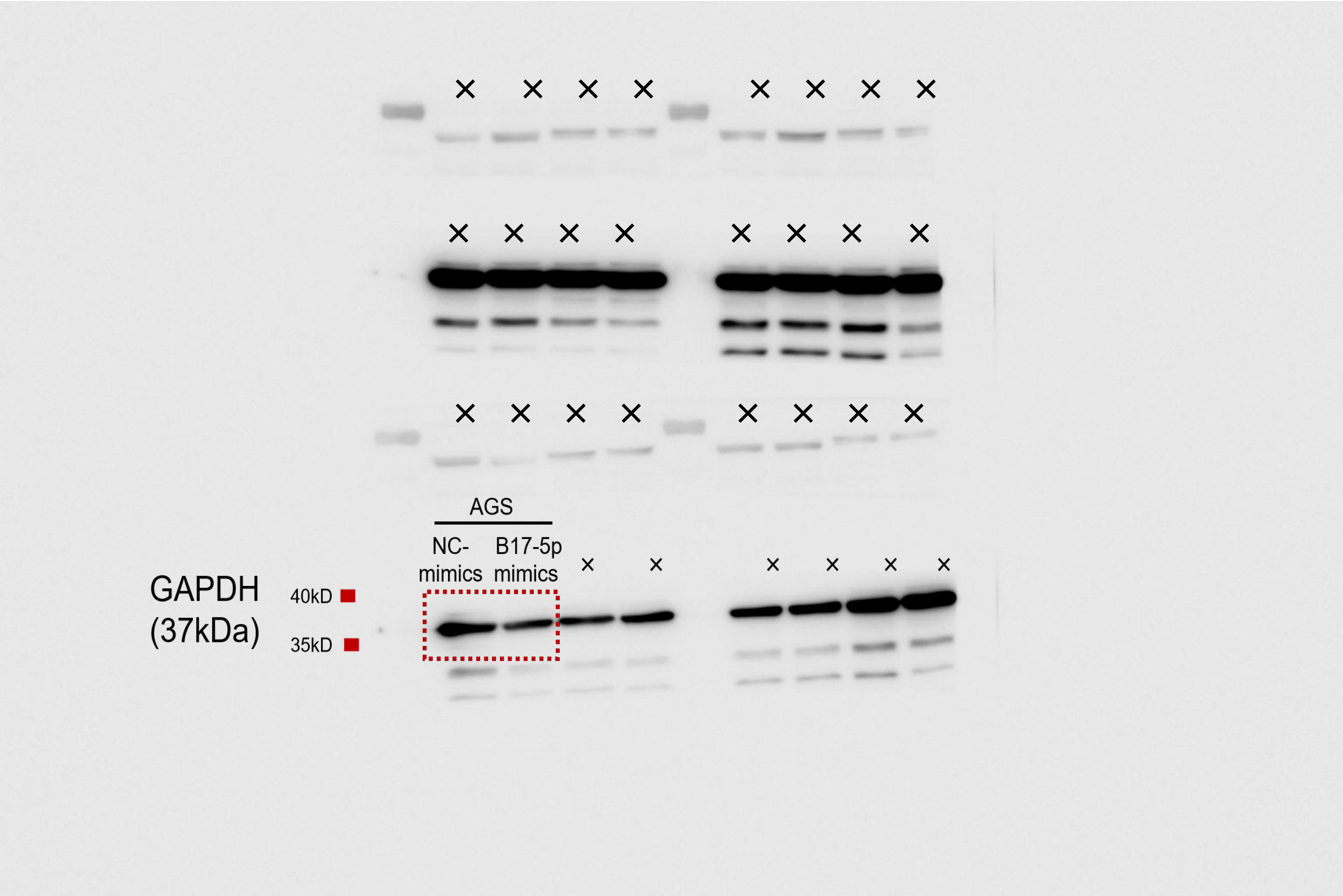

This image corresponds to Figure 2 H

Biological Replicate #3

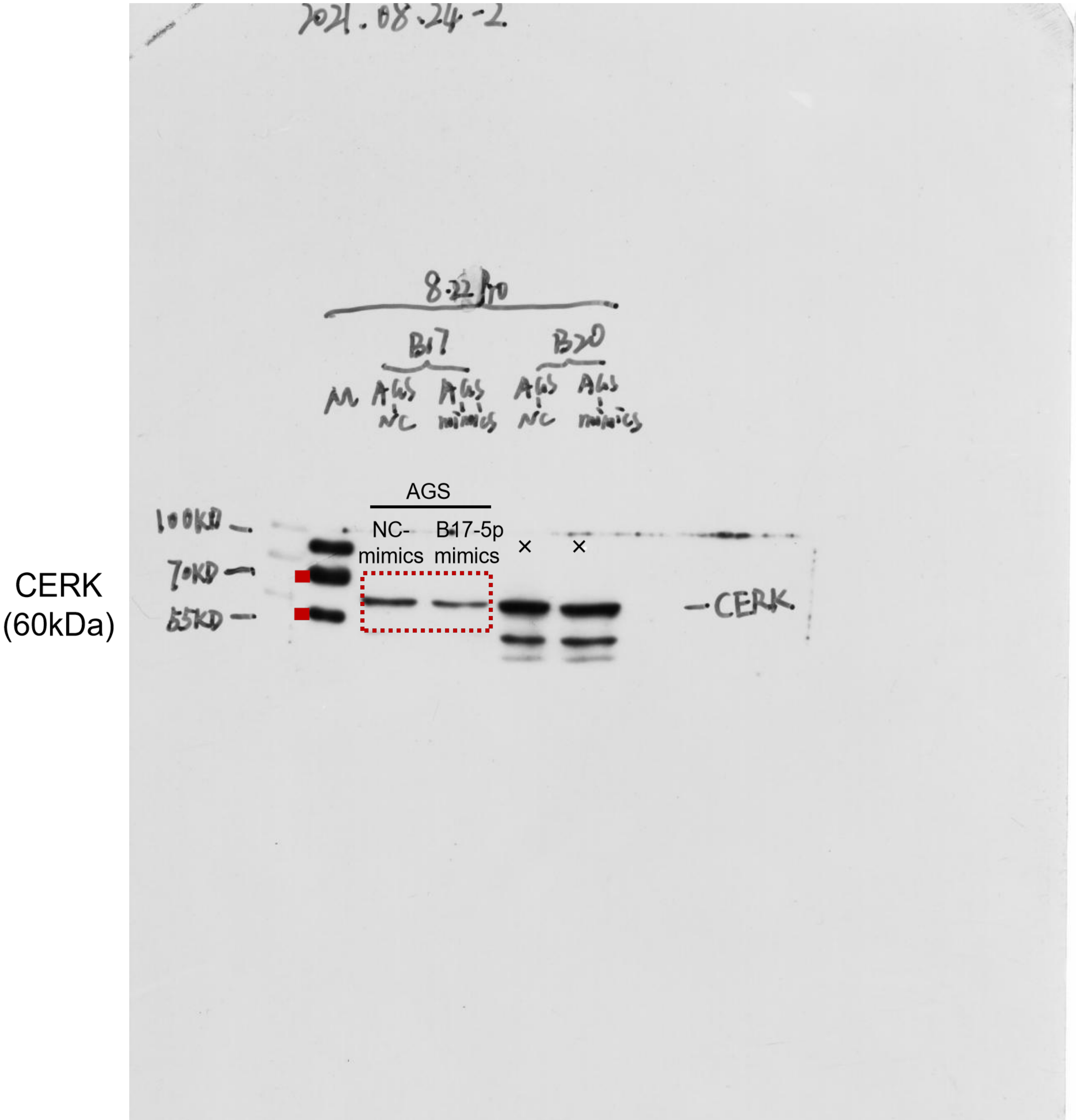

This image corresponds to Figure 2 H

Biological Replicate #3

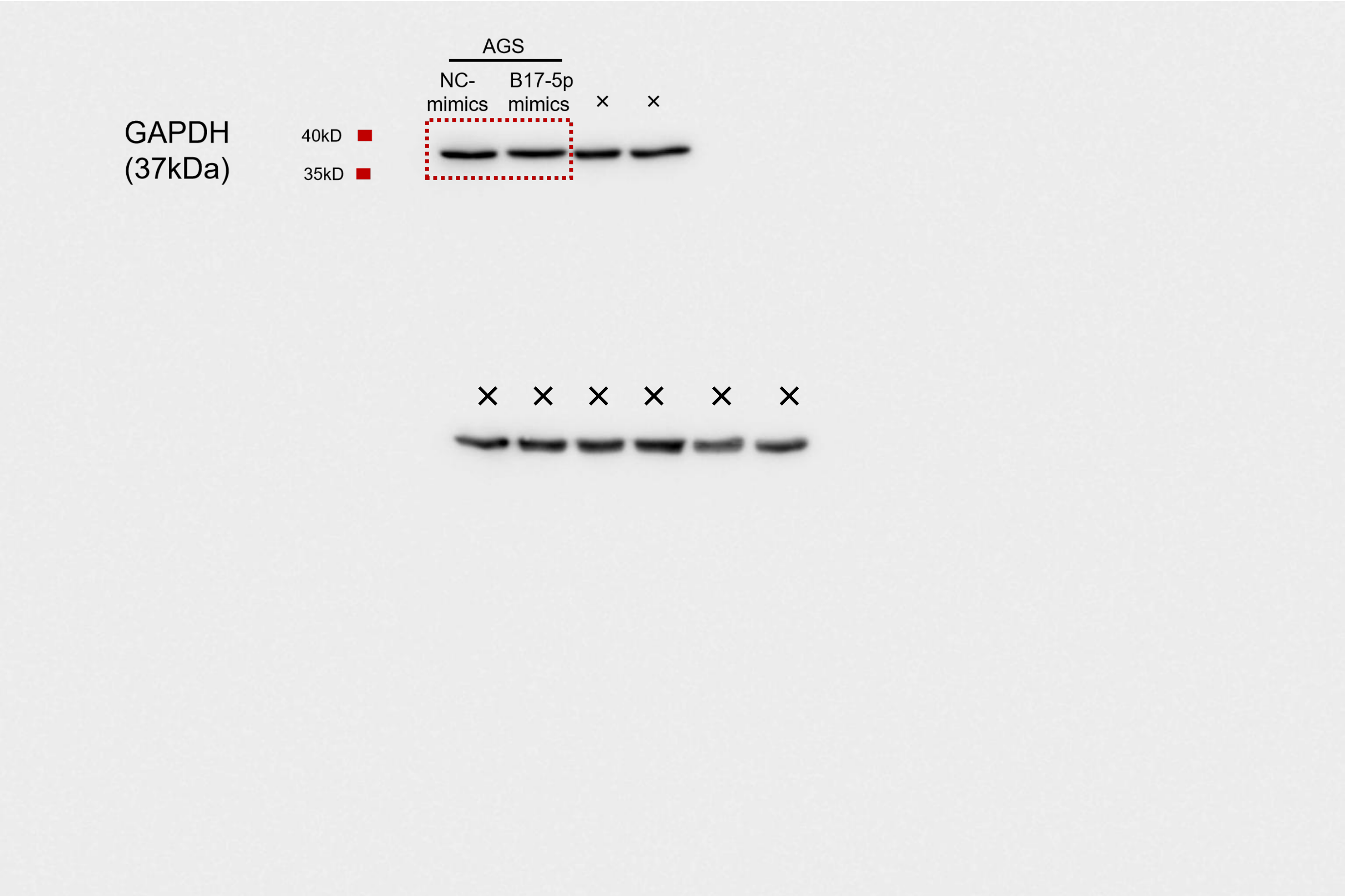

These images correspond to Figure 2K

Biological Replicate #1

▲ These images was selected as the representative result in Figure 2K.

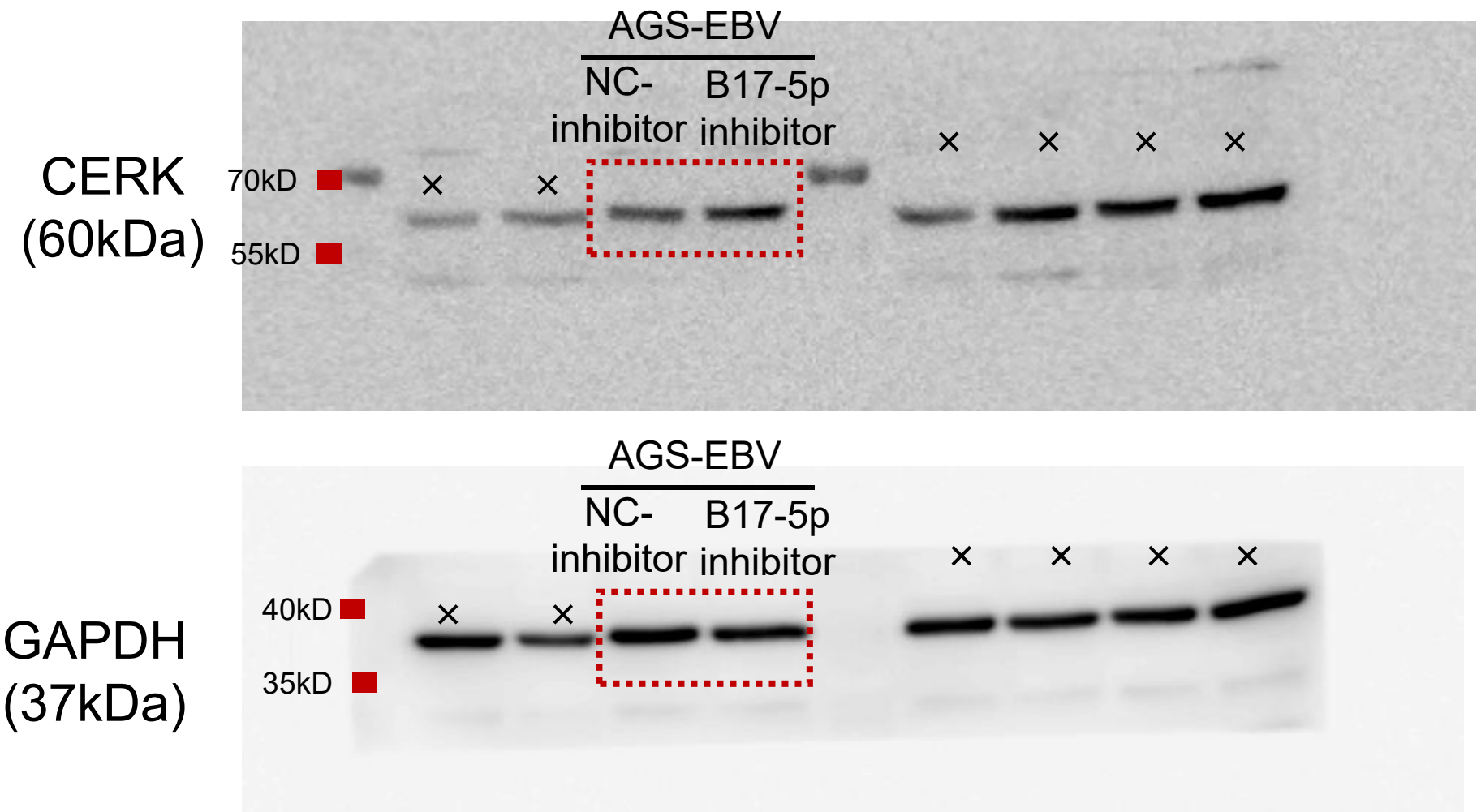

Biological Replicate #2

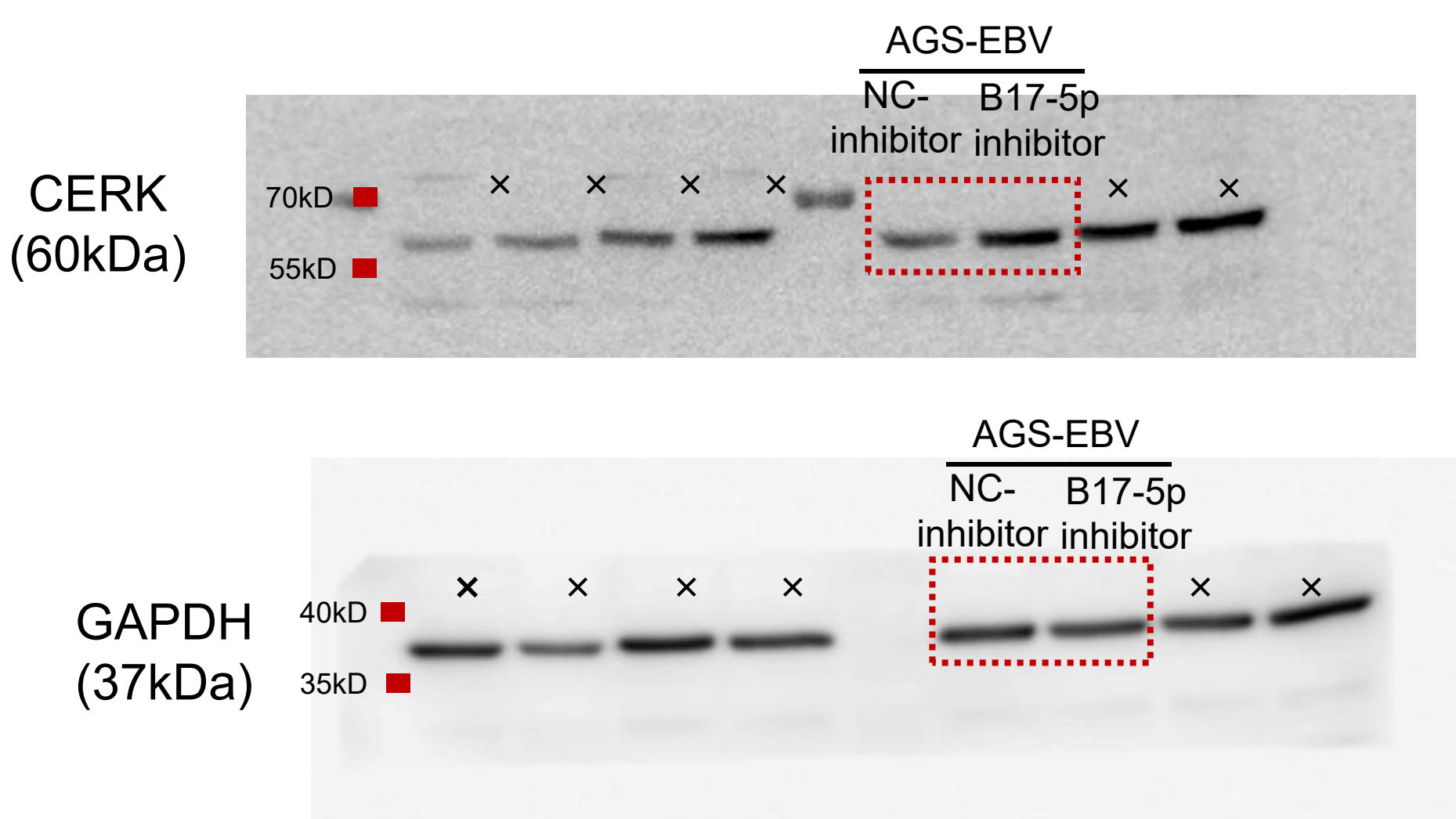

Biological Replicate #3

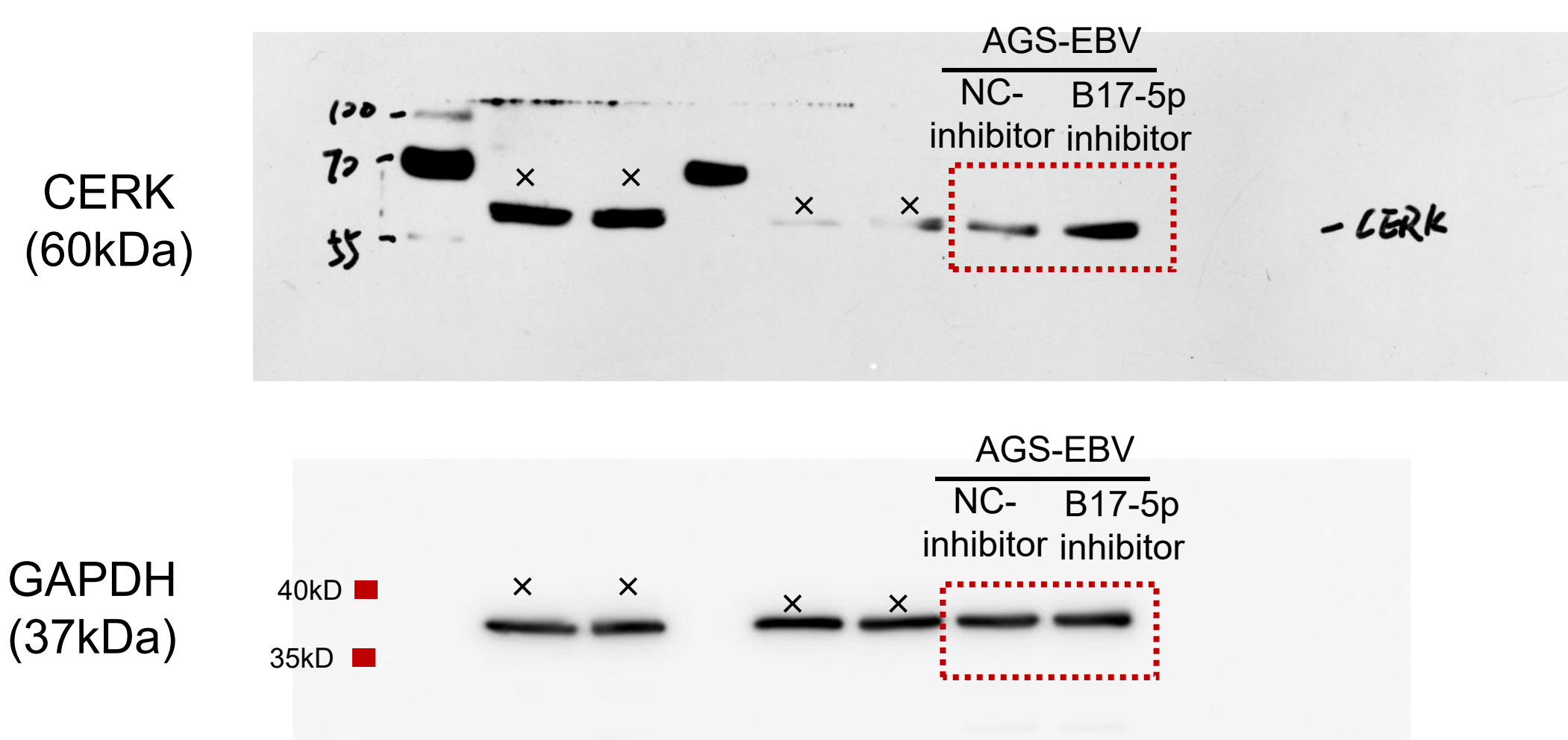

This image corresponds to Figure 2K

Biological Replicate #1

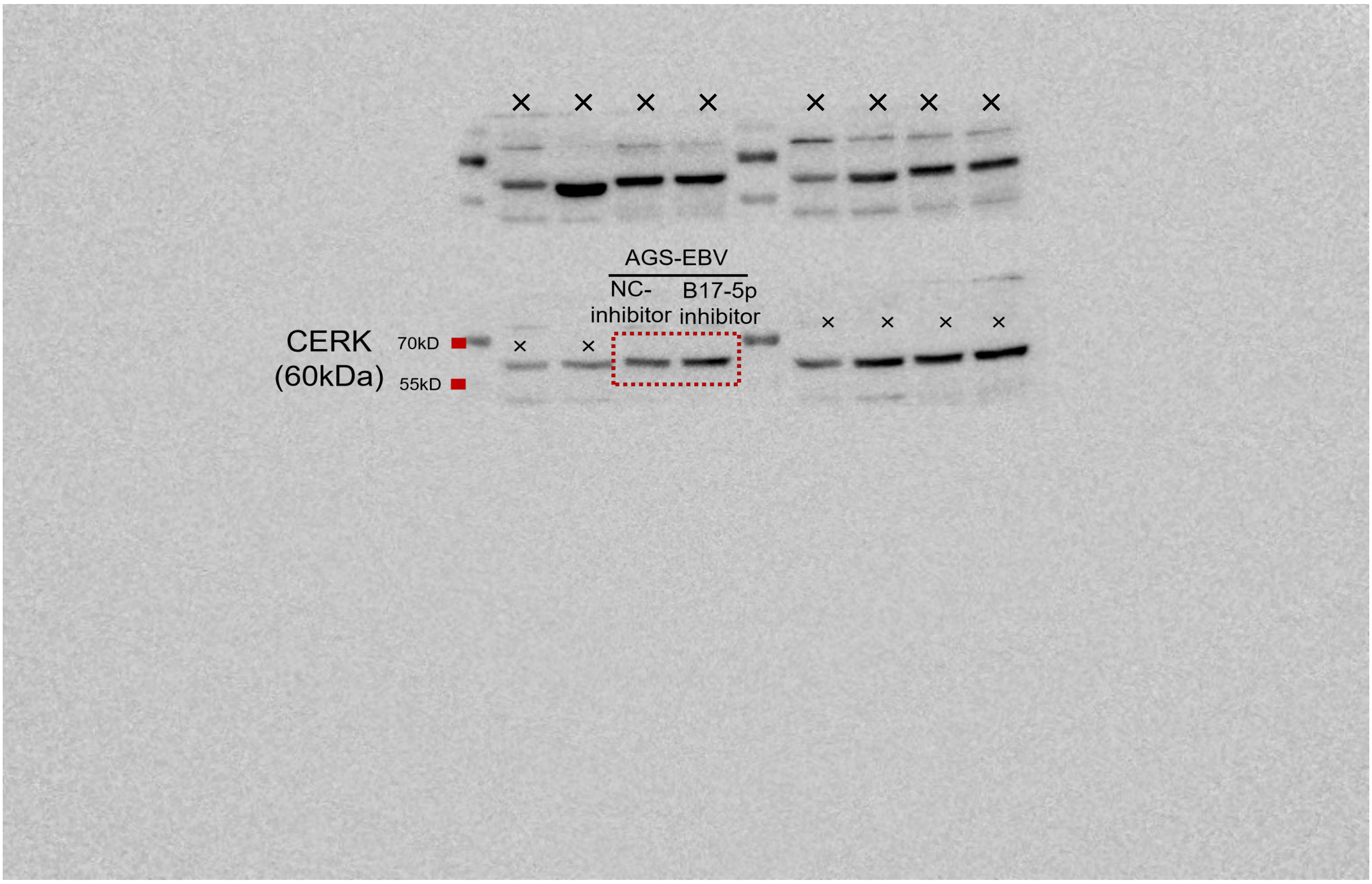

This image corresponds to Figure 2K

Biological Replicate #1

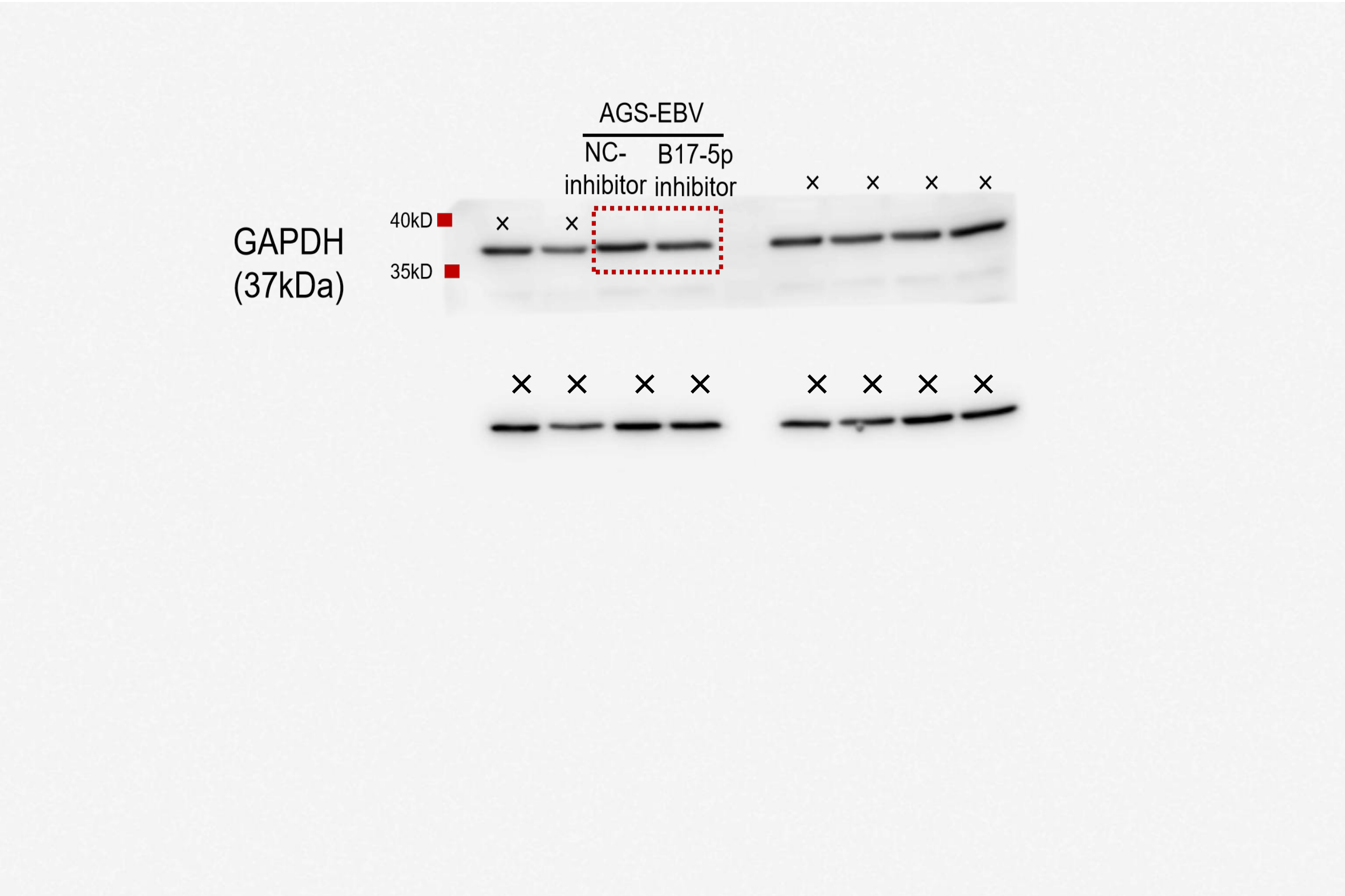

This image corresponds to Figure 2K

Biological Replicate #2

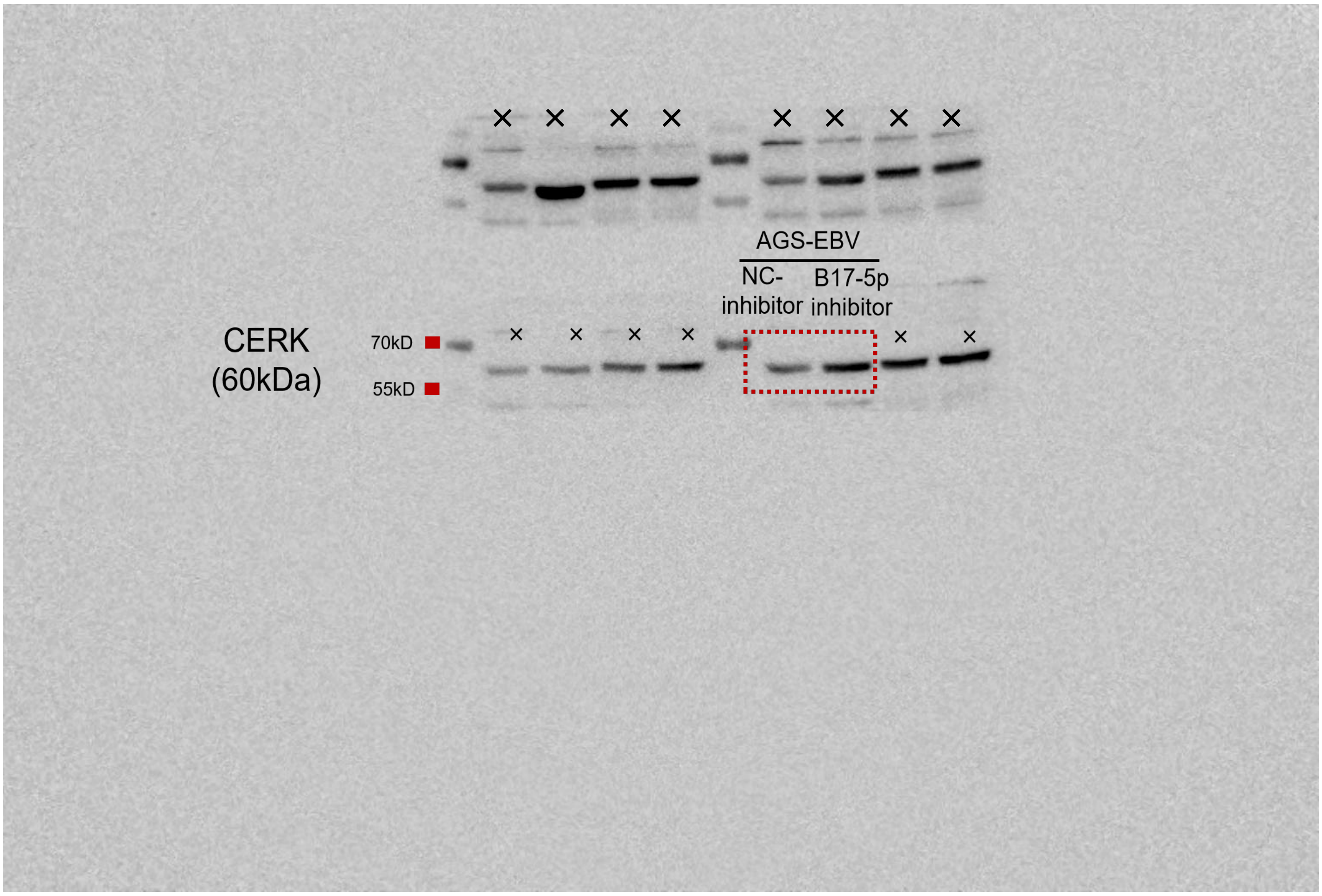

This image corresponds to Figure 2K

Biological Replicate #2

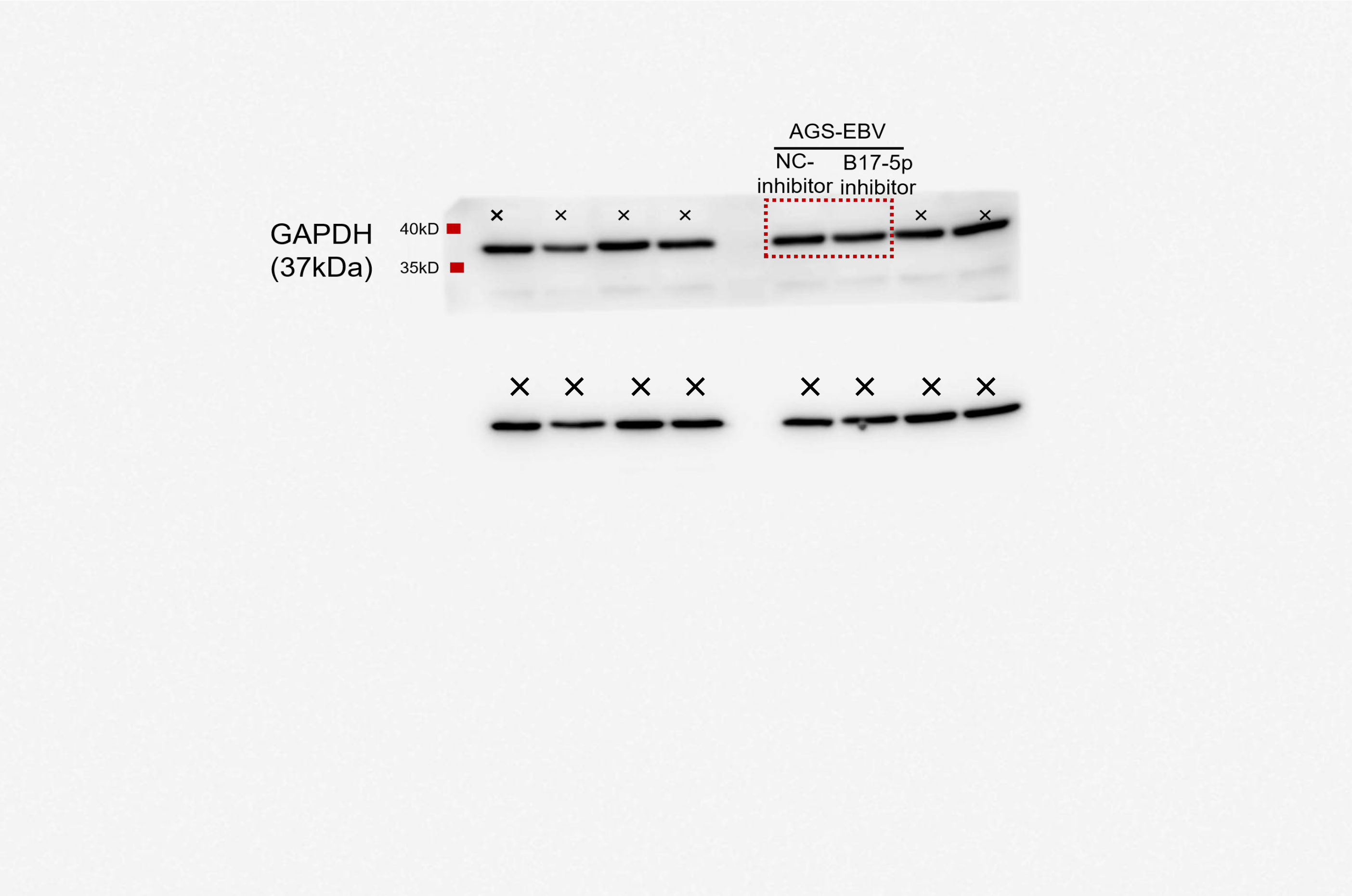

This image corresponds to Figure 2K

Biological Replicate #3

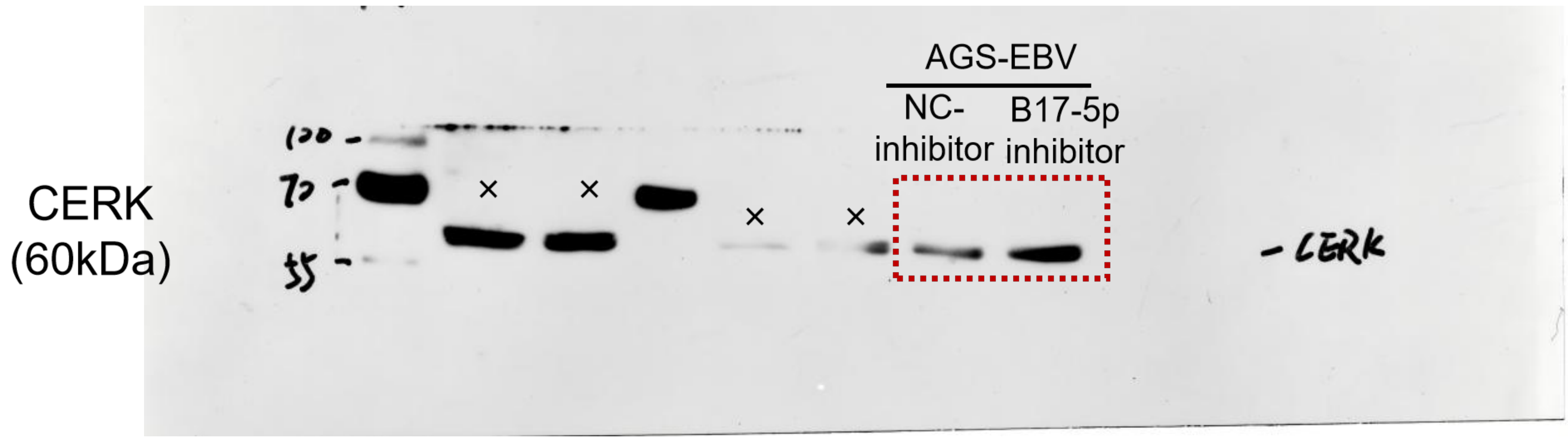

This image corresponds to Figure 2K

Biological Replicate #3

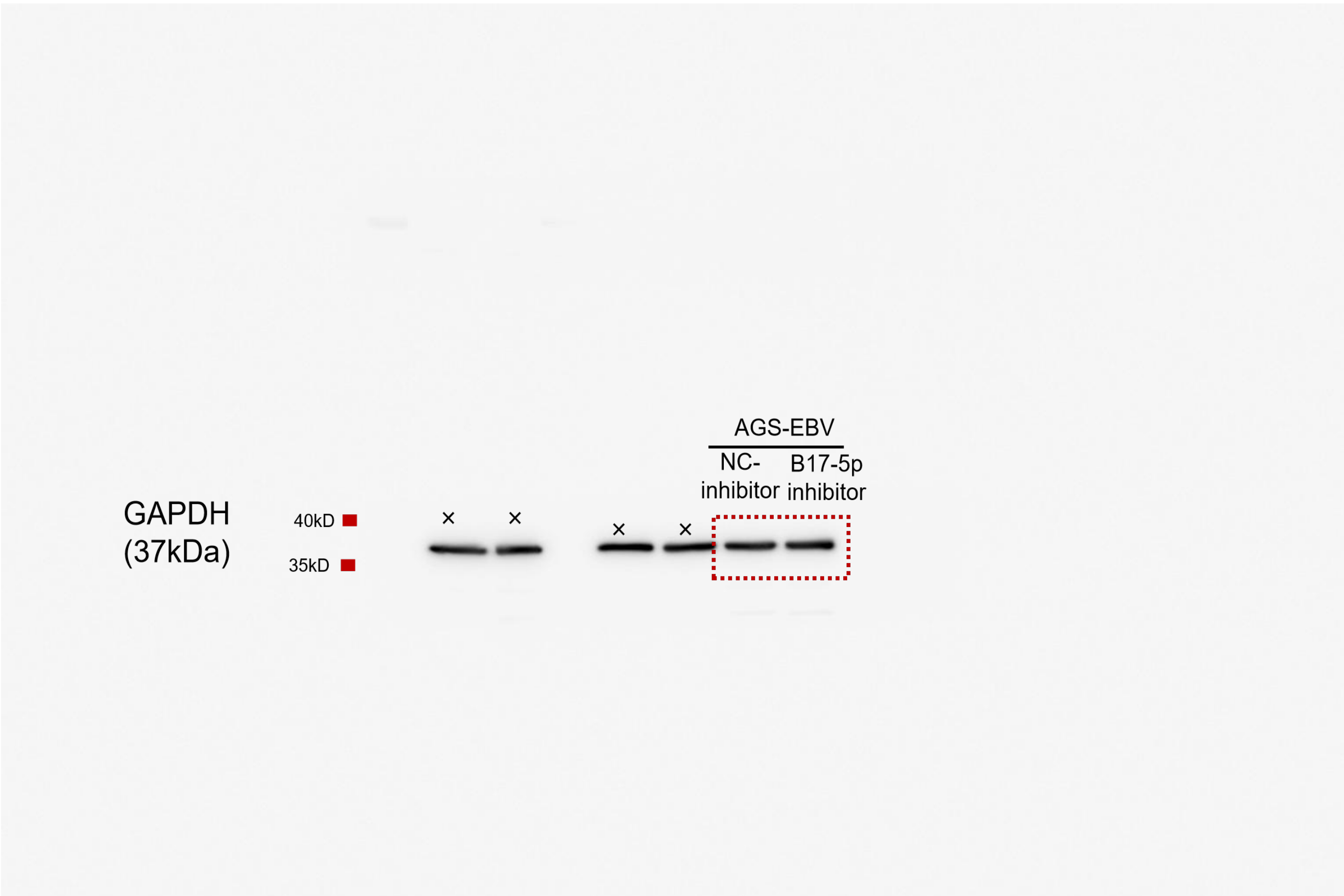

These images correspond to Figure 2N

Biological Replicate #1

▲ These images was selected as the representative result in Figure 2N.

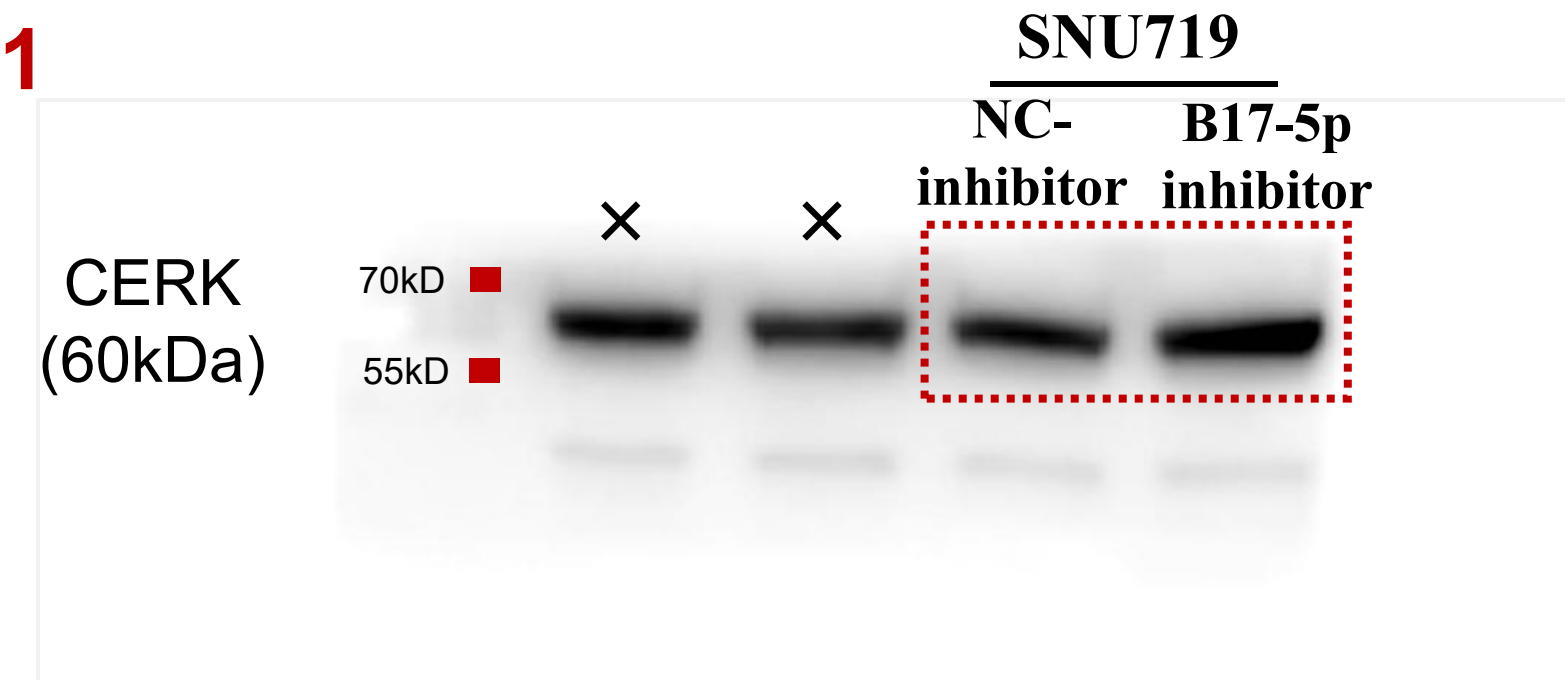

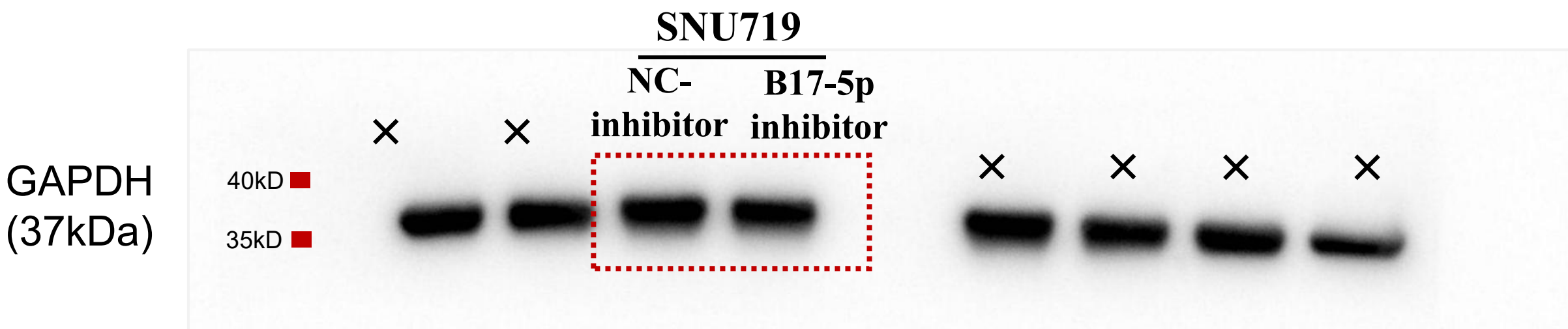

Biological Replicate #2

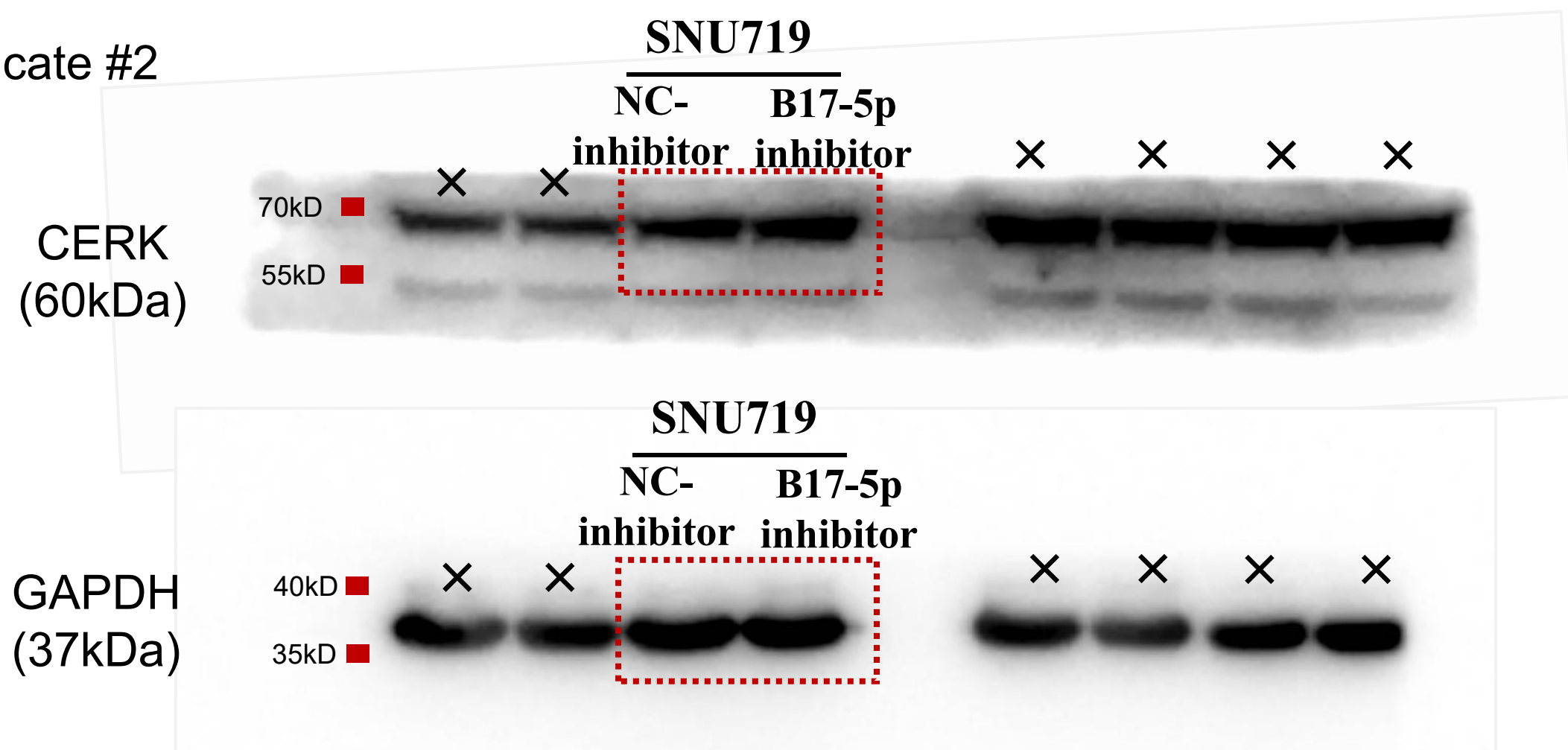

Biological Replicate #3

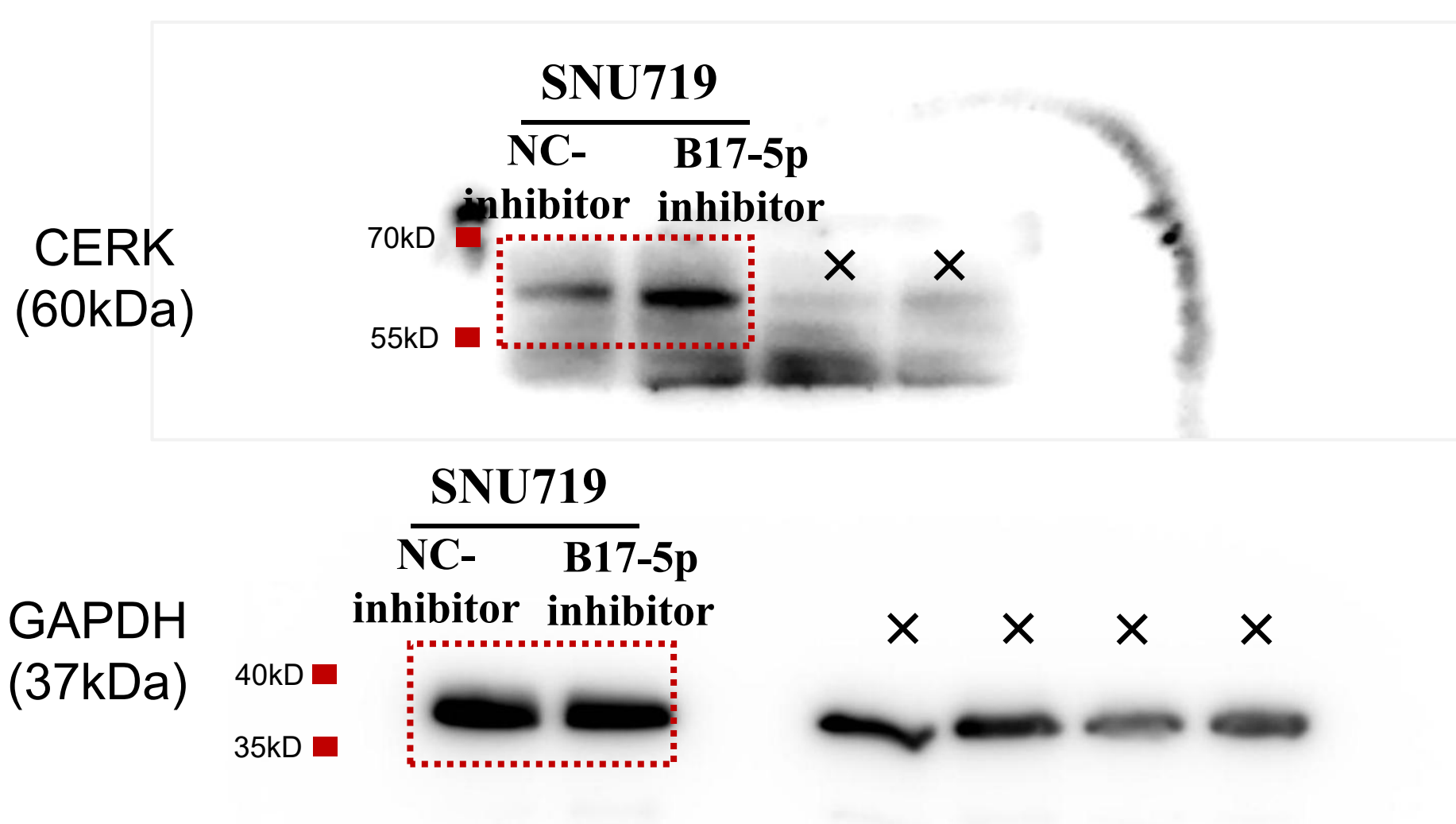

This image corresponds to Figure 2N

Biological Replicate #1

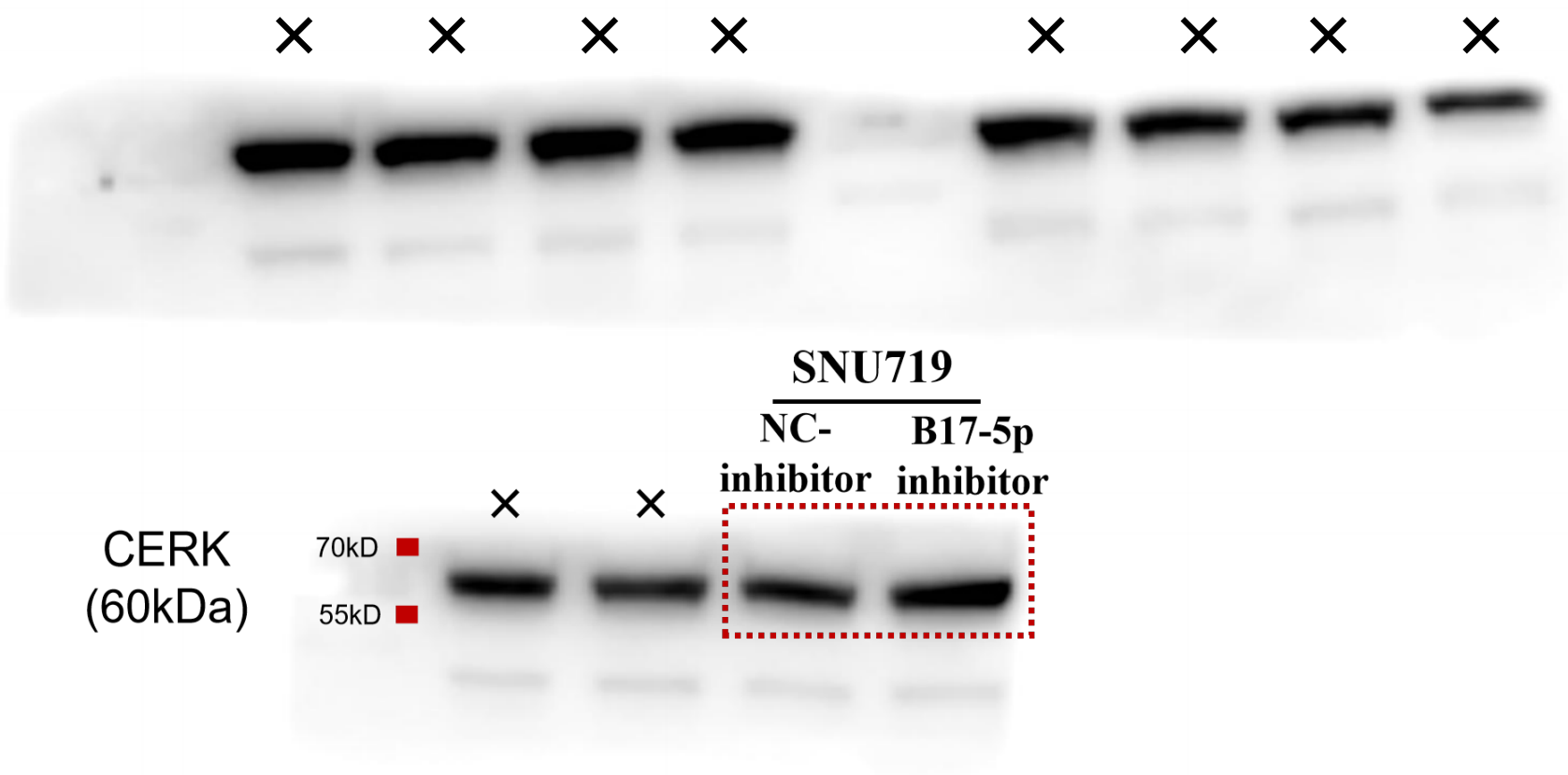

This image corresponds to Figure 2N

Biological Replicate #1

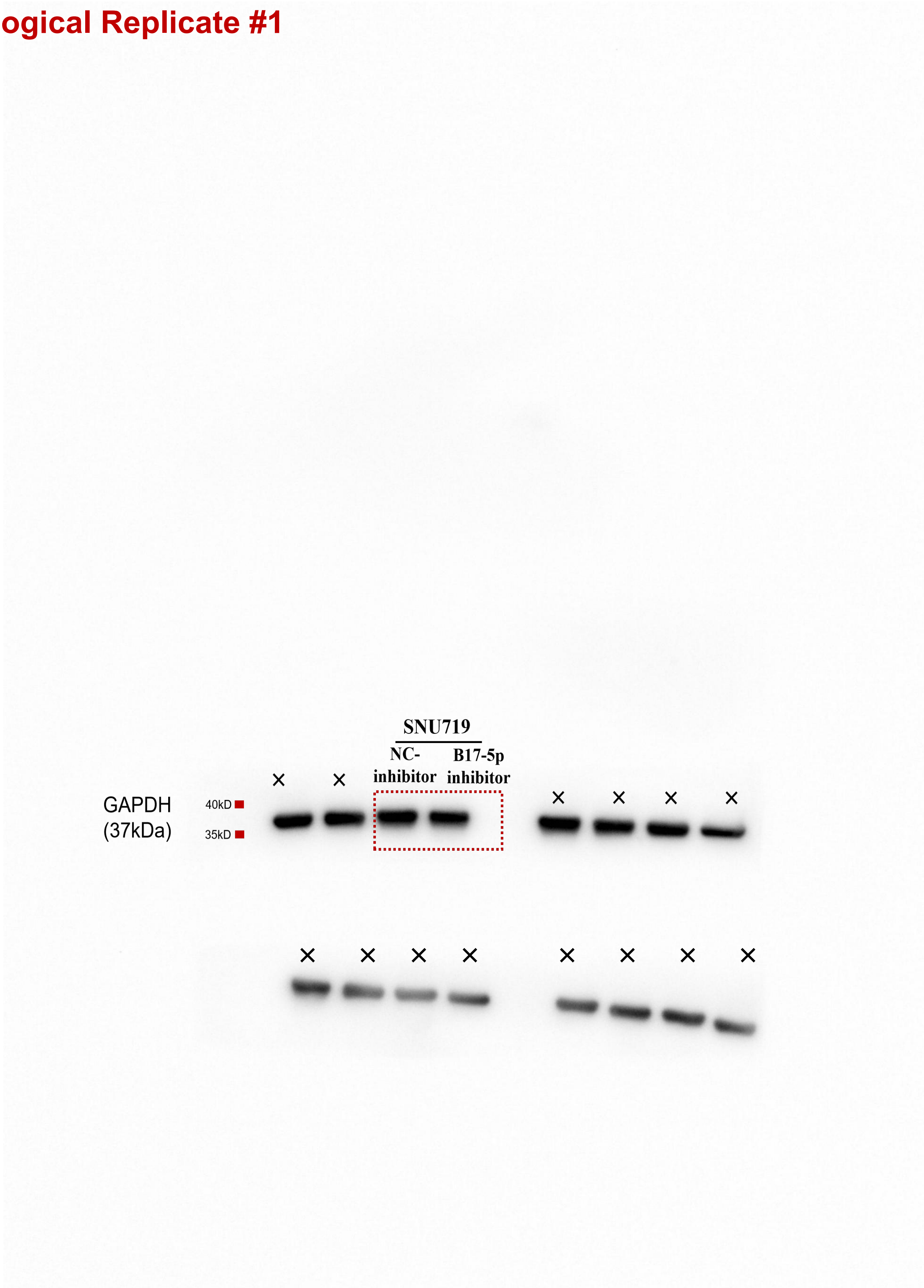

This image corresponds to Figure 2N

Biological Replicate #2

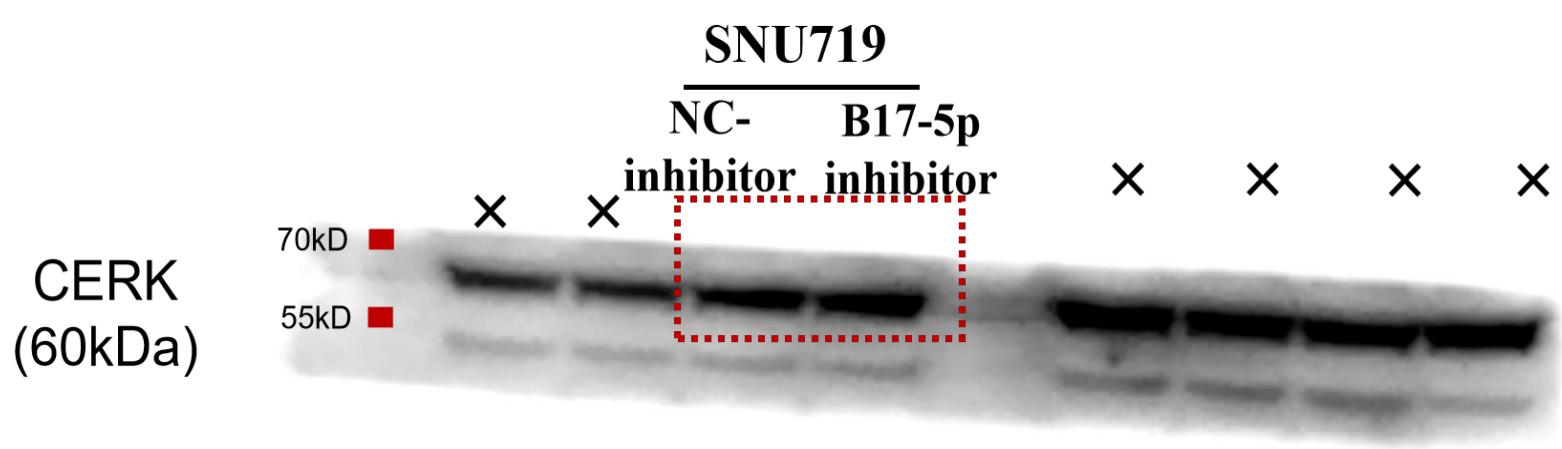

This image corresponds to Figure 2N

Biological Replicate #2

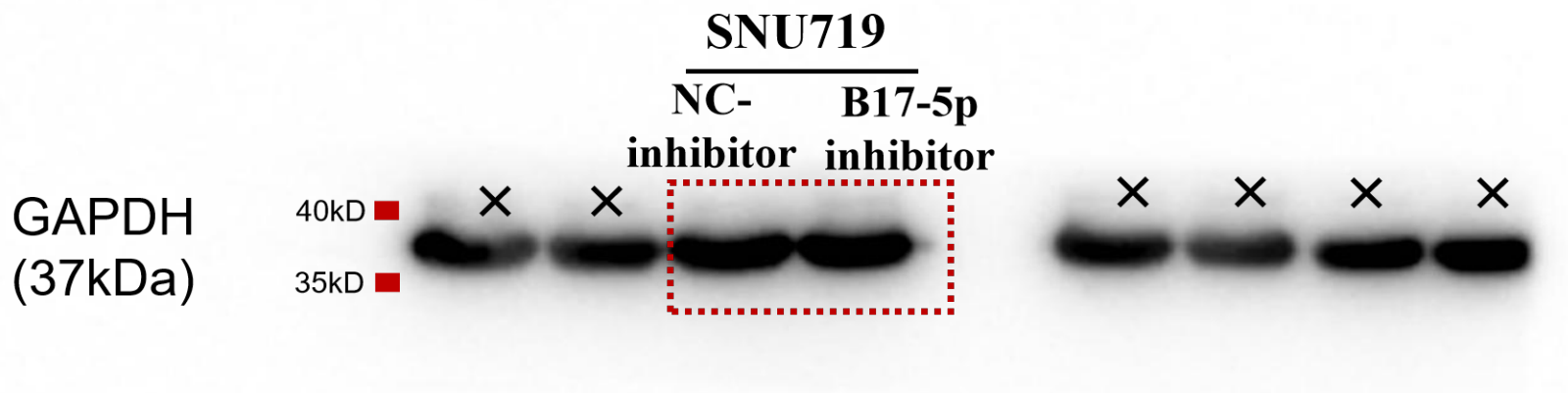

This image corresponds to Figure 2N

Biological Replicate #3

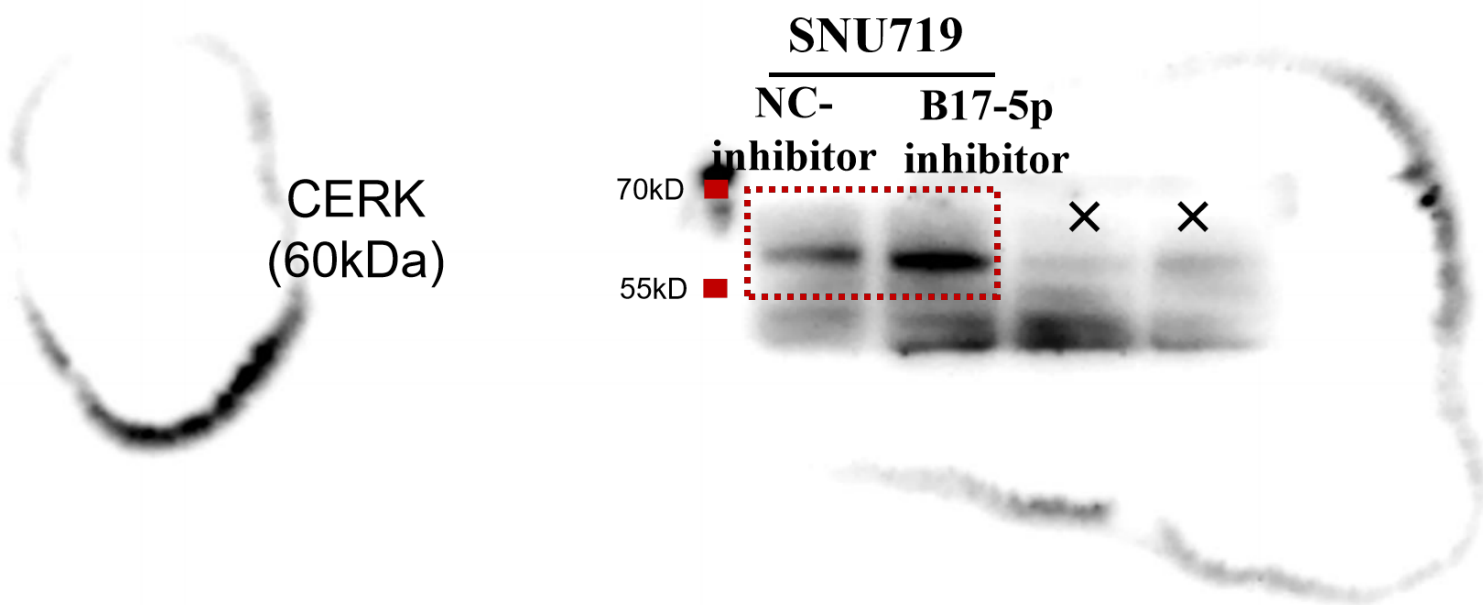

This image corresponds to Figure 2N

Biological Replicate #3

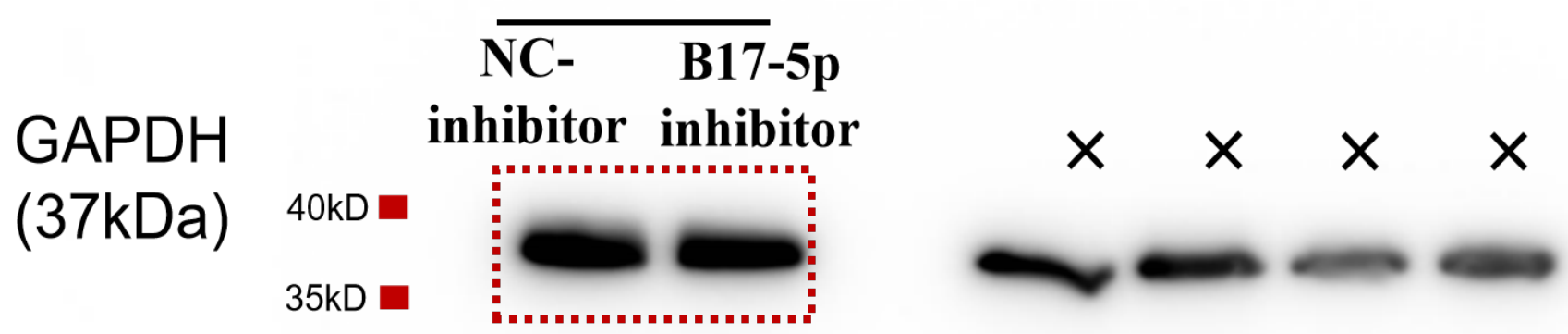

These images correspond to Figure 6A

Biological Replicate #1

▲ These images was selected as the representative result in Figure 6A.

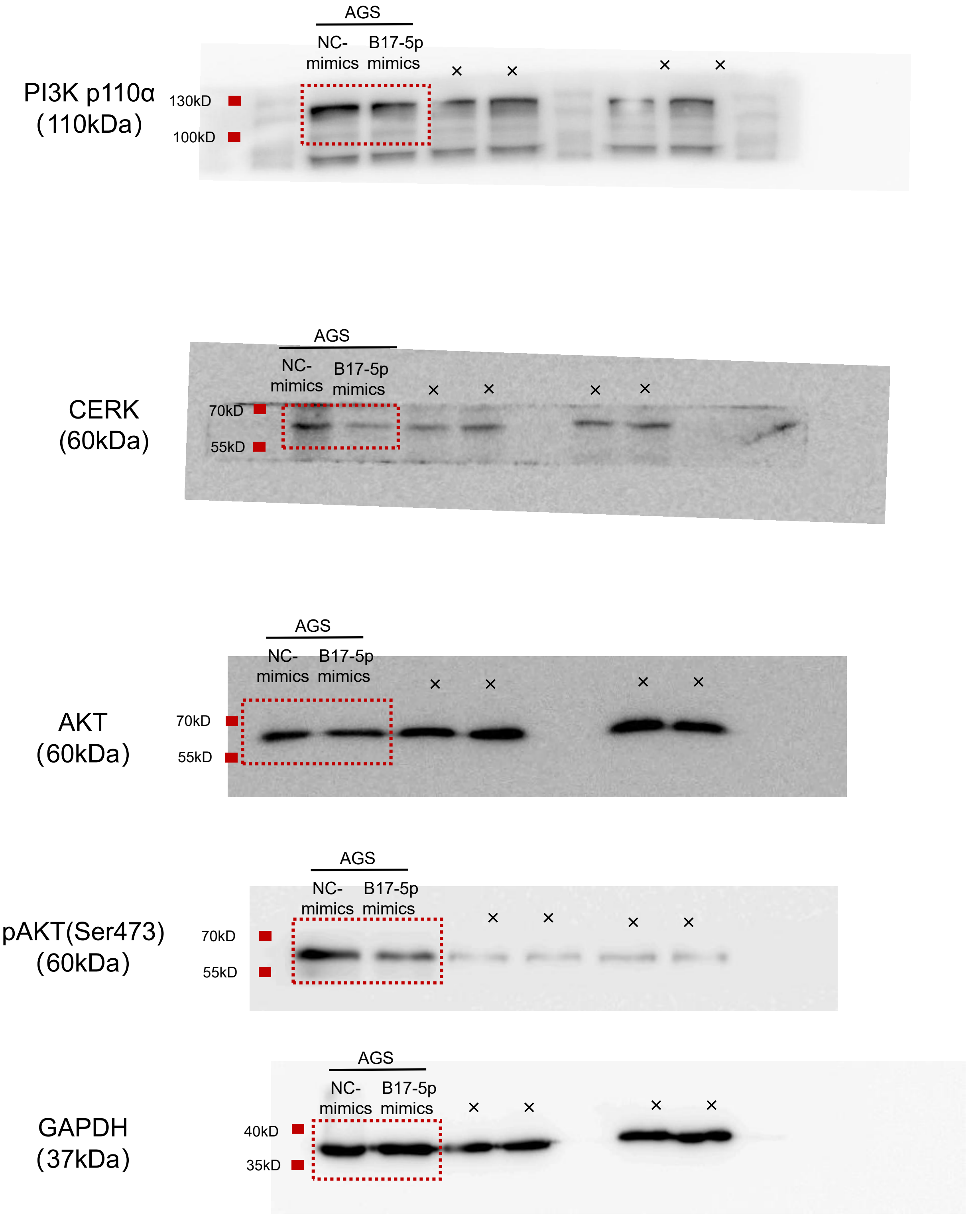

This image corresponds to Figure 6A

Biological Replicate #1

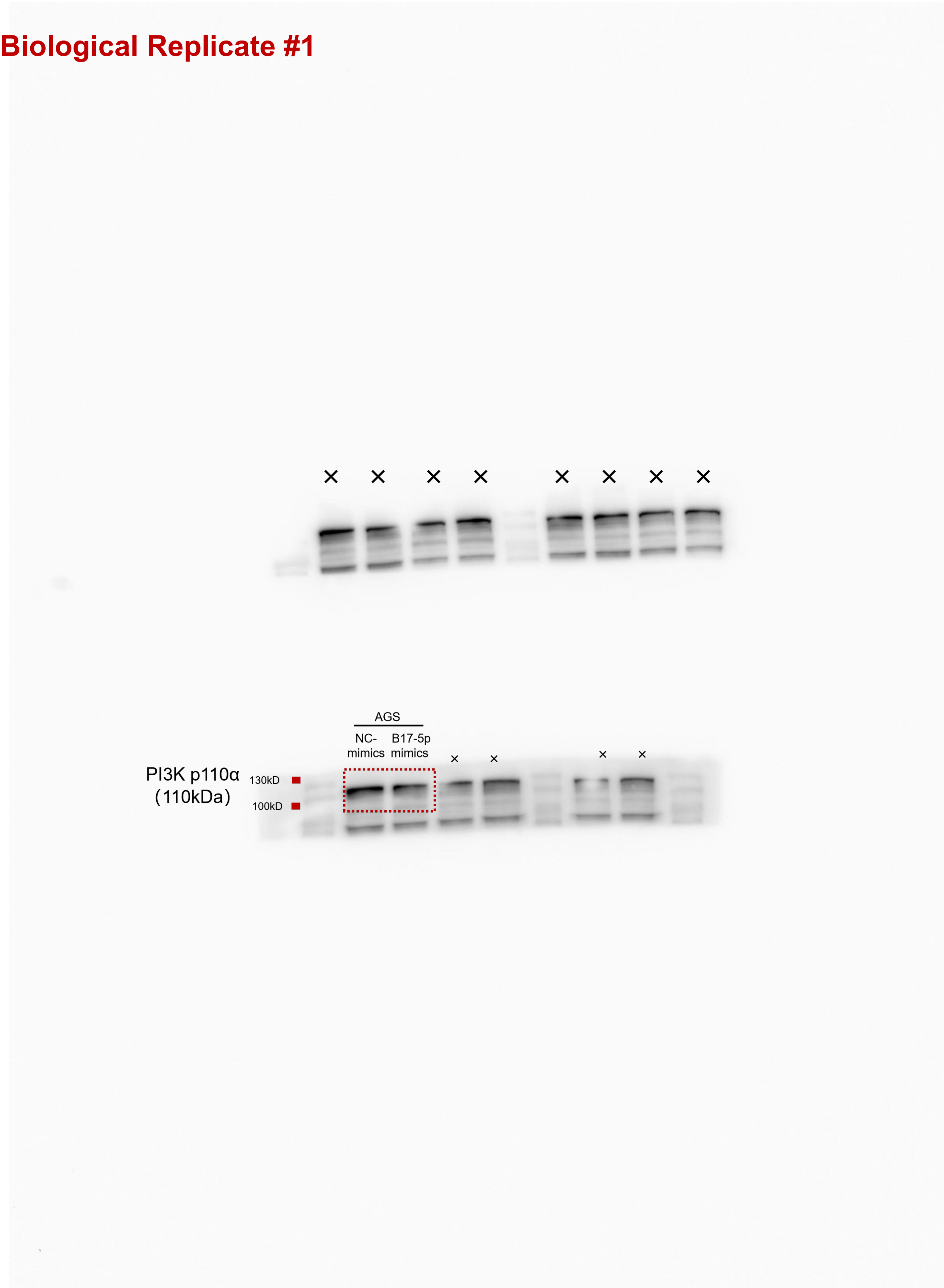

This image corresponds to Figure 6A

Biological Replicate #1

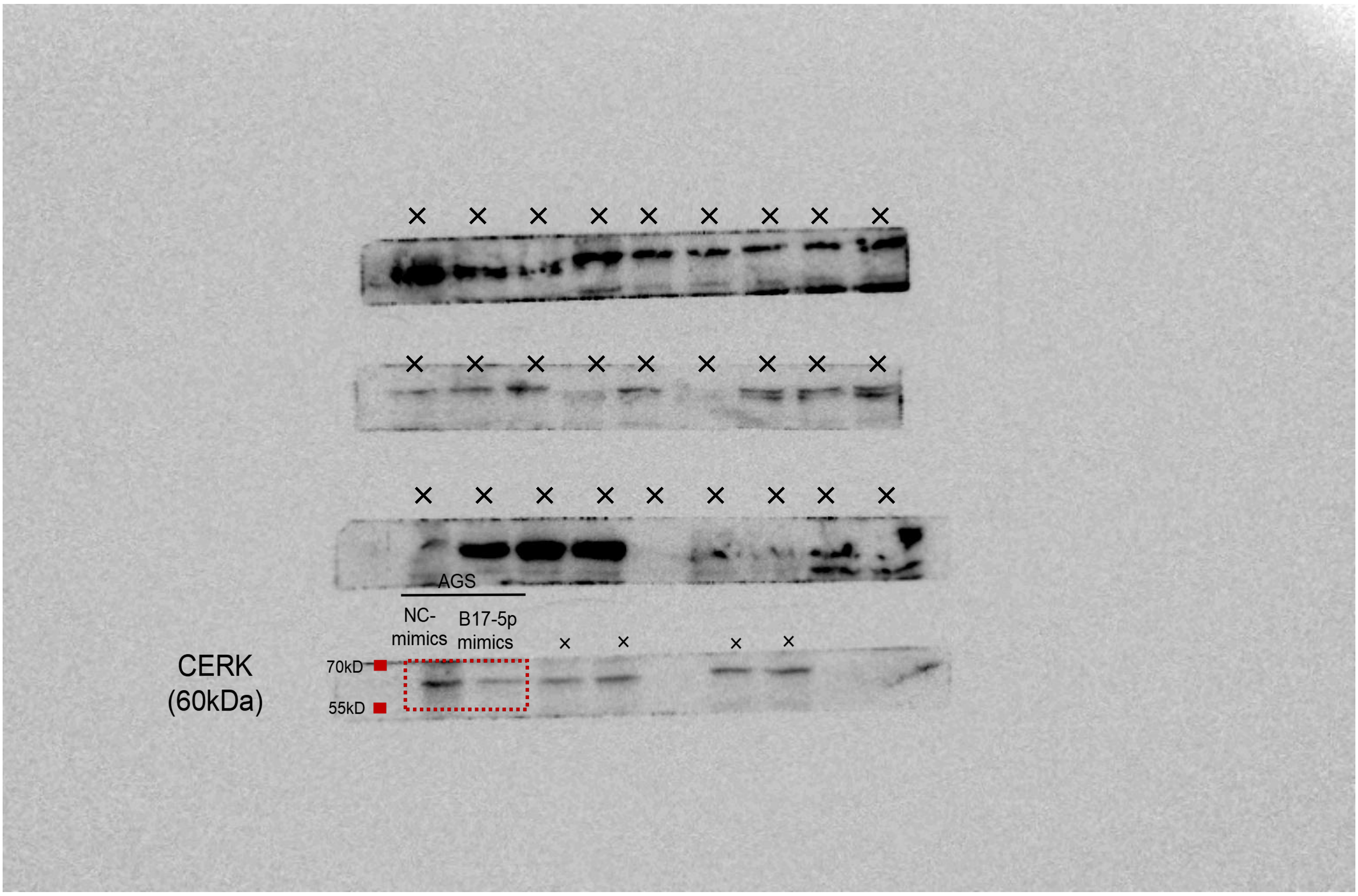

This image corresponds to Figure 6A

Biological Replicate #1

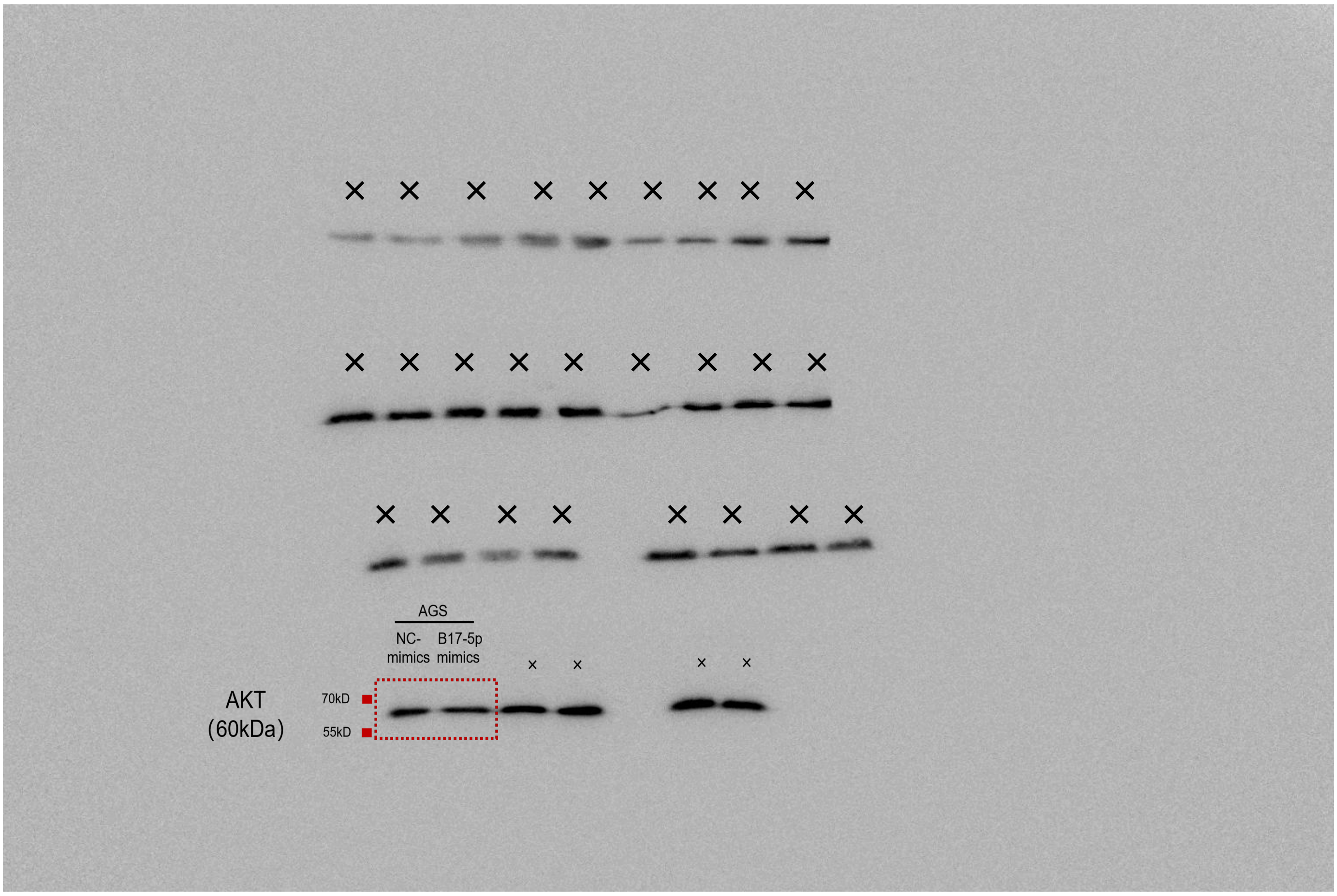

This image corresponds to Figure 6A

Biological Replicate #1

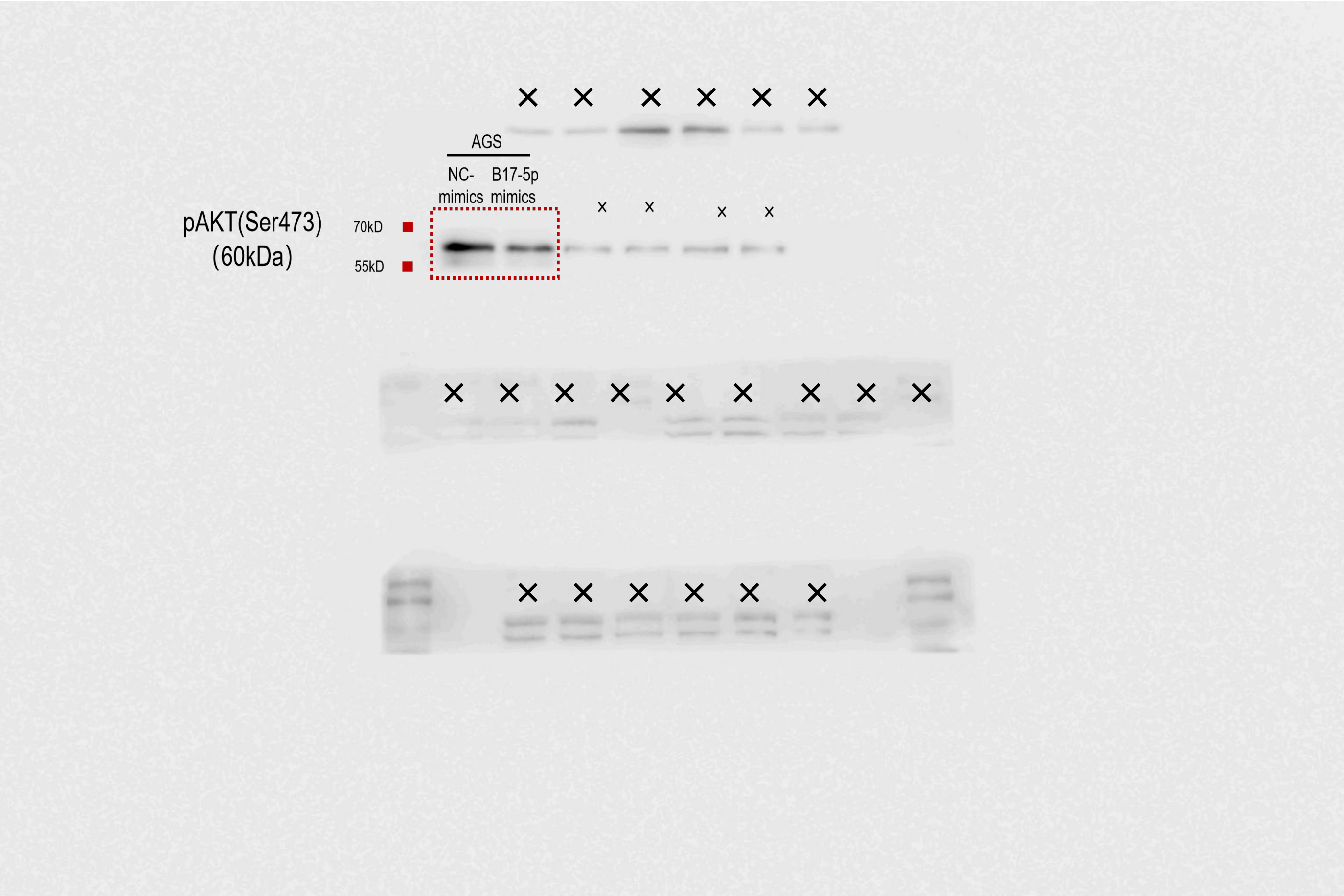

This image corresponds to Figure 6A

Biological Replicate #1

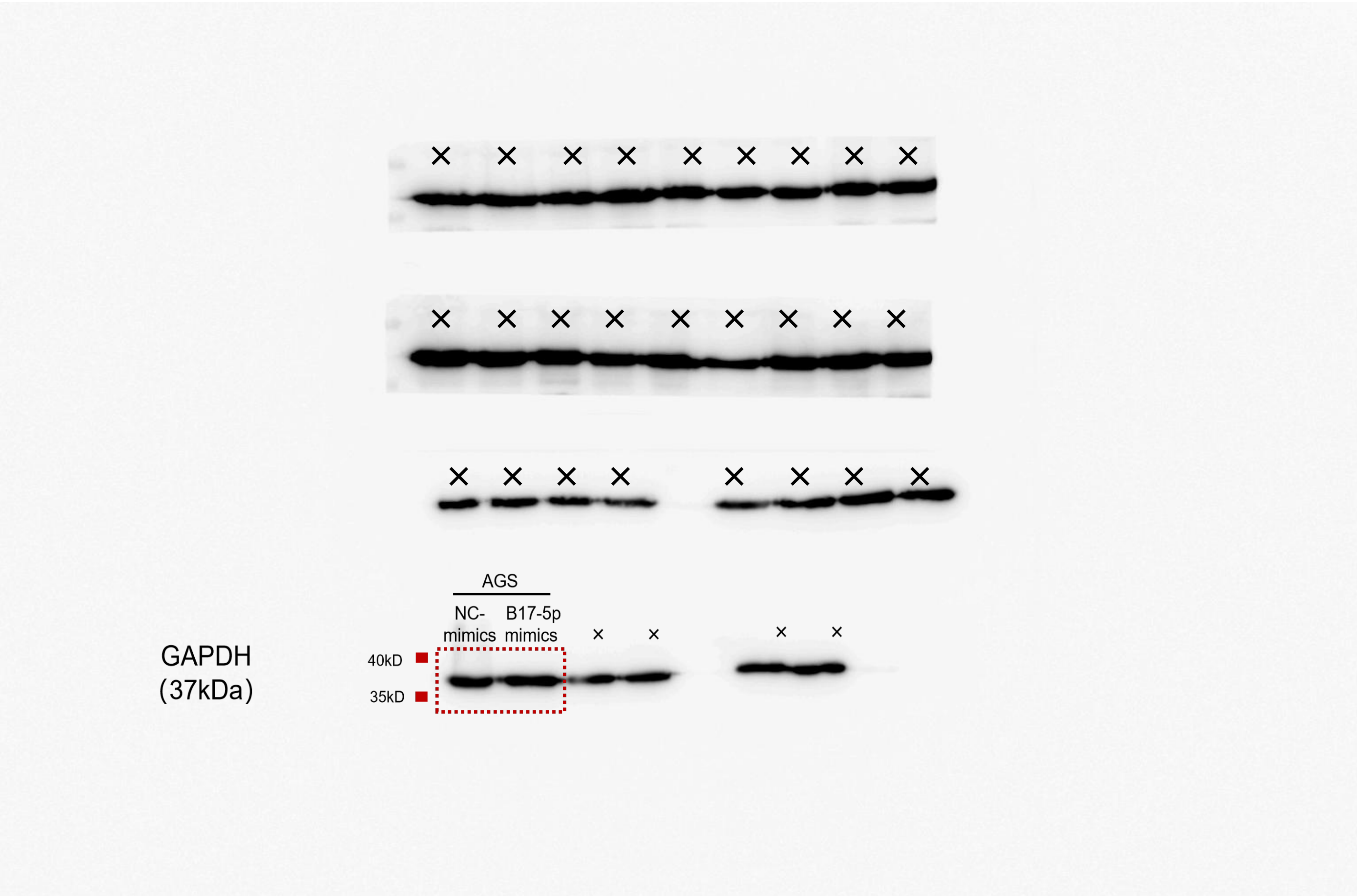

These images correspond to Figure 6A

Biological Replicate #2

PI3K p110α
(110kDa)

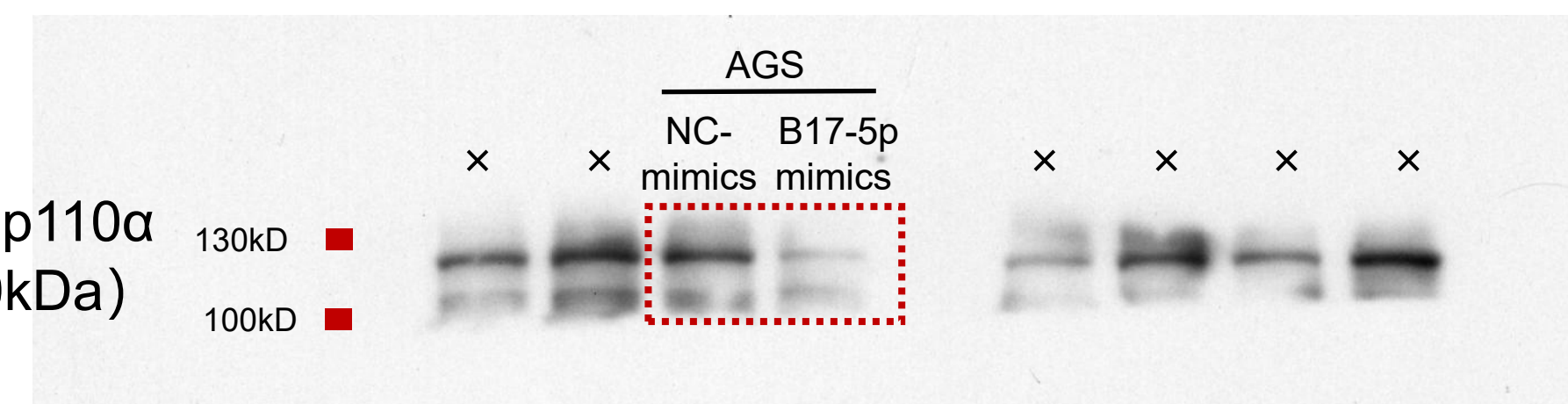

CERK
(60kDa)

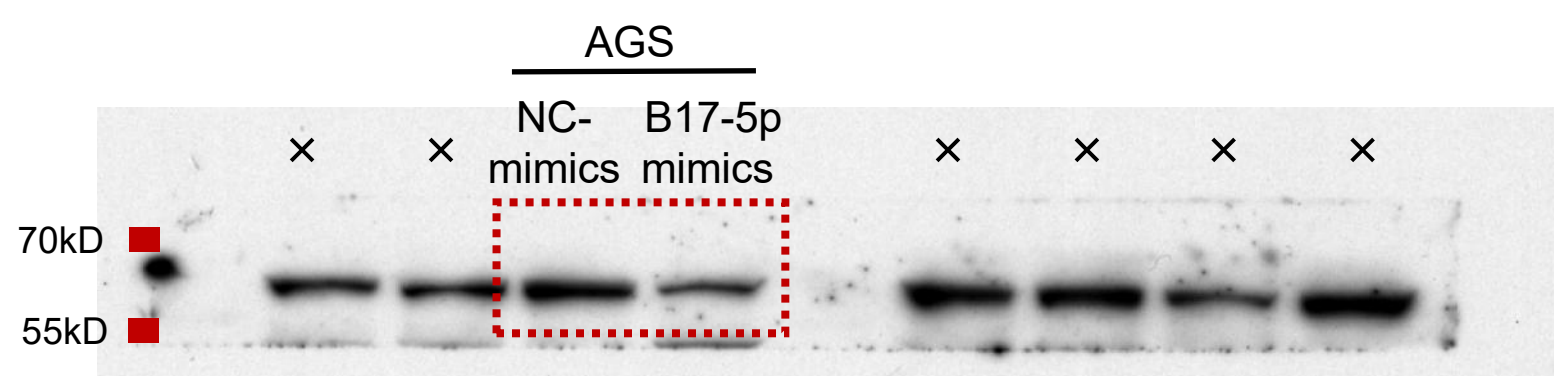

AKT
(60kDa)

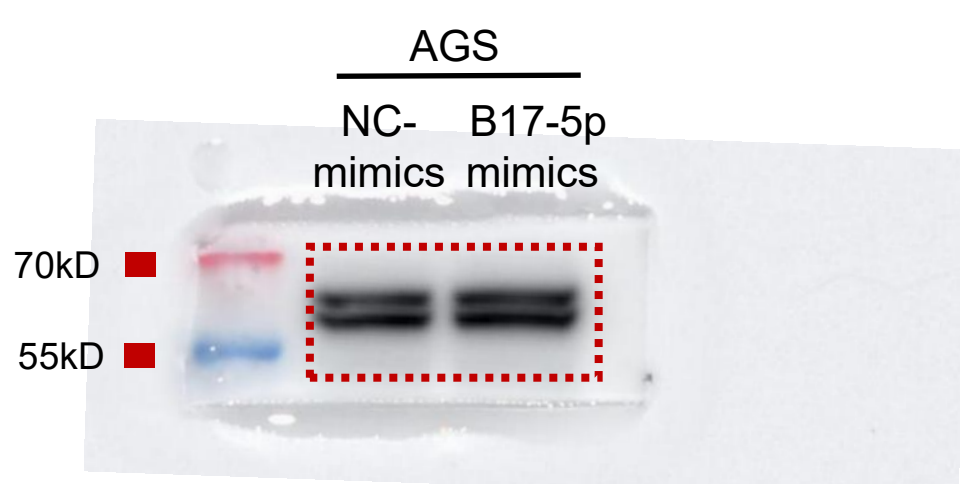

pAKT(Ser473)
(60kDa)

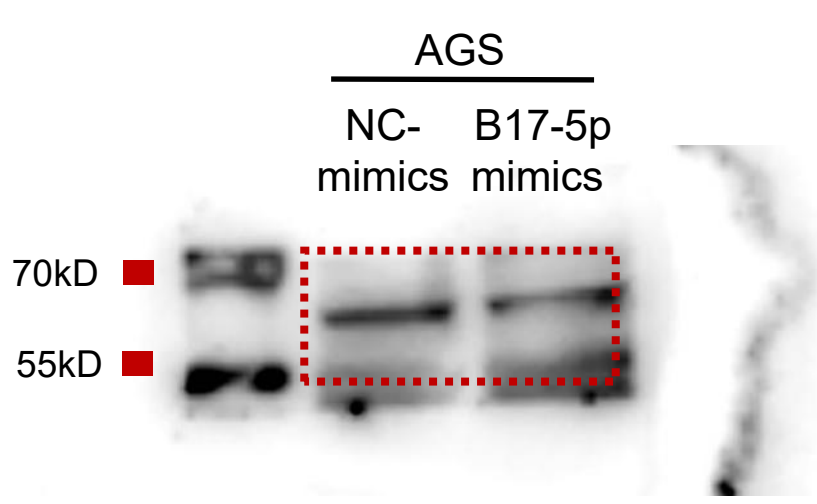

GAPDH
(37kDa)

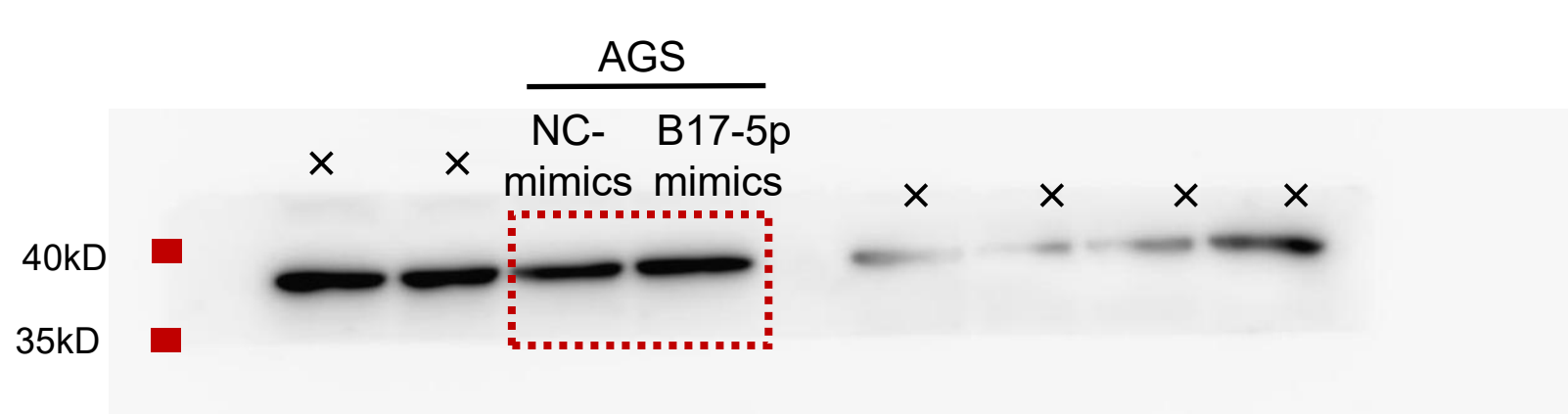

This image corresponds to Figure 6A

Biological Replicate #2

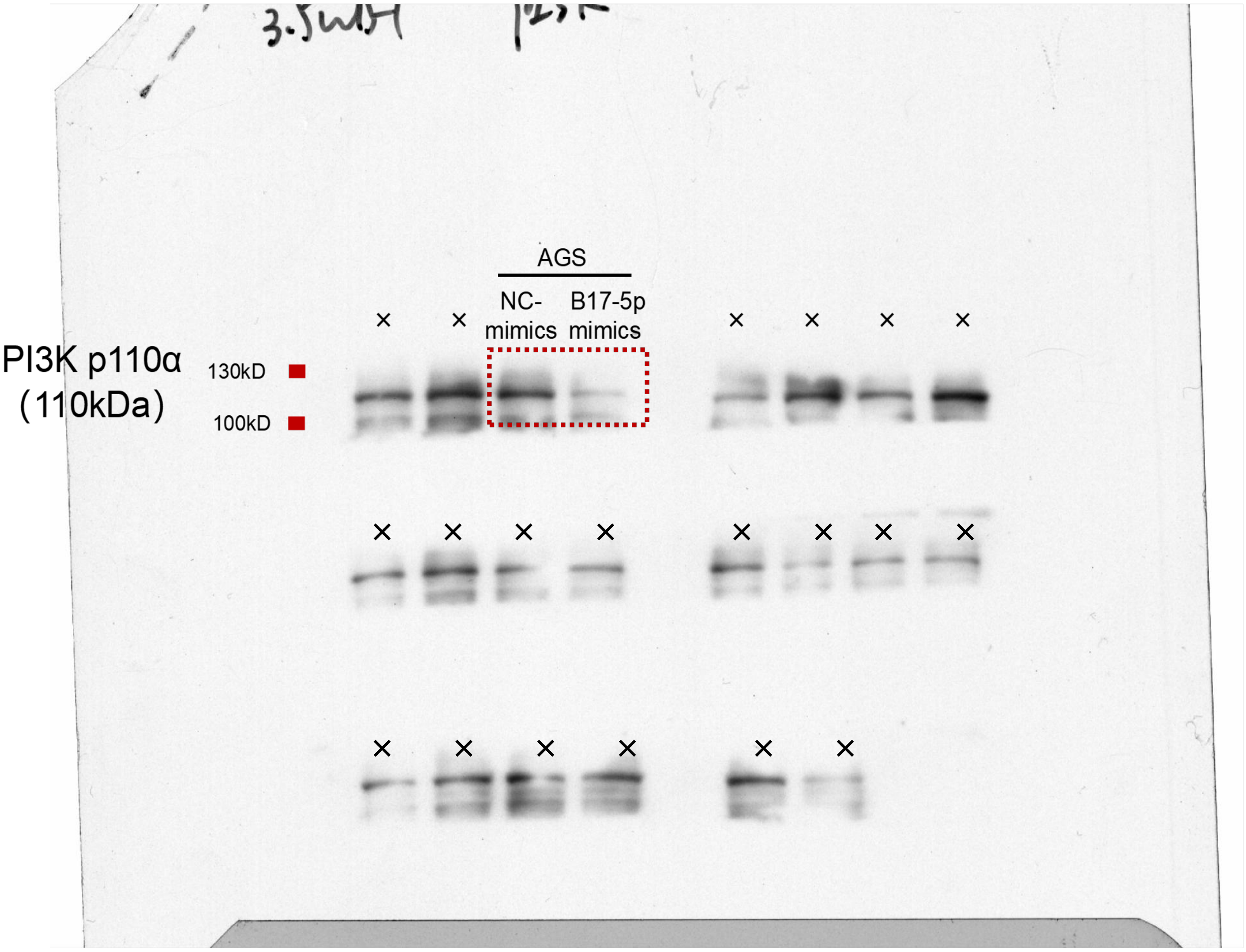

This image corresponds to Figure 6A

Biological Replicate #2

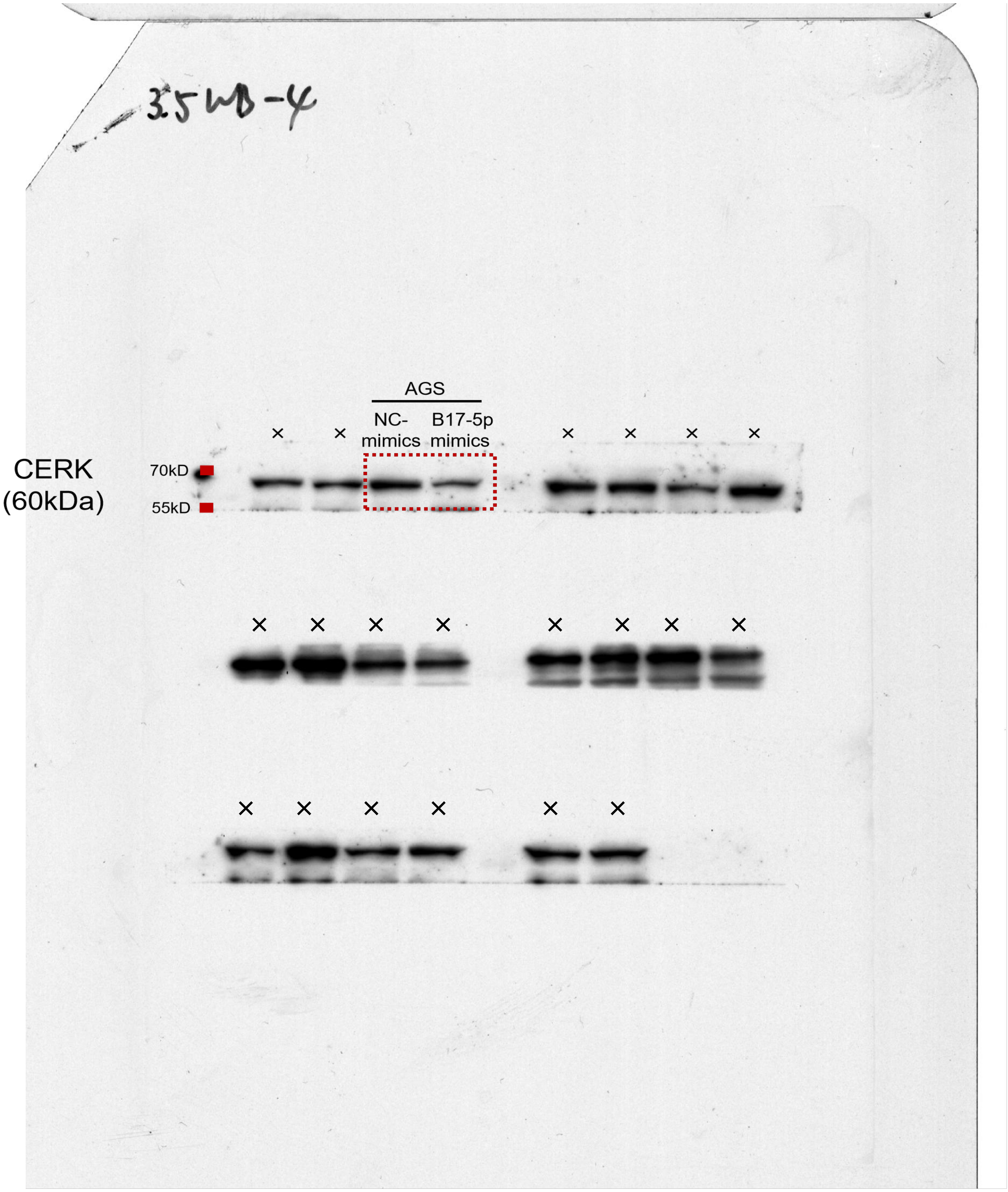

This image corresponds to Figure 6A

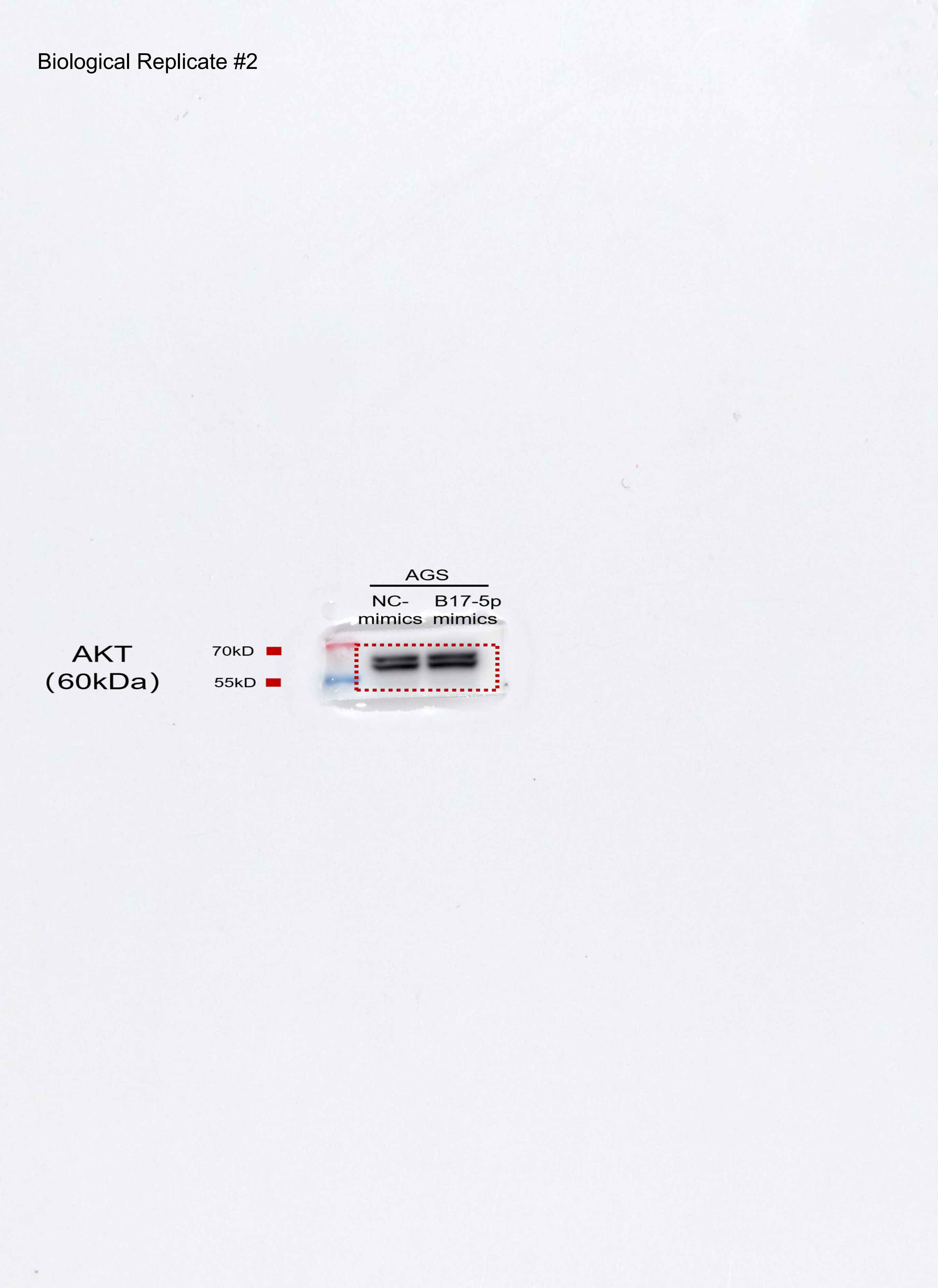

This image corresponds to Figure 6A

Biological Replicate #2

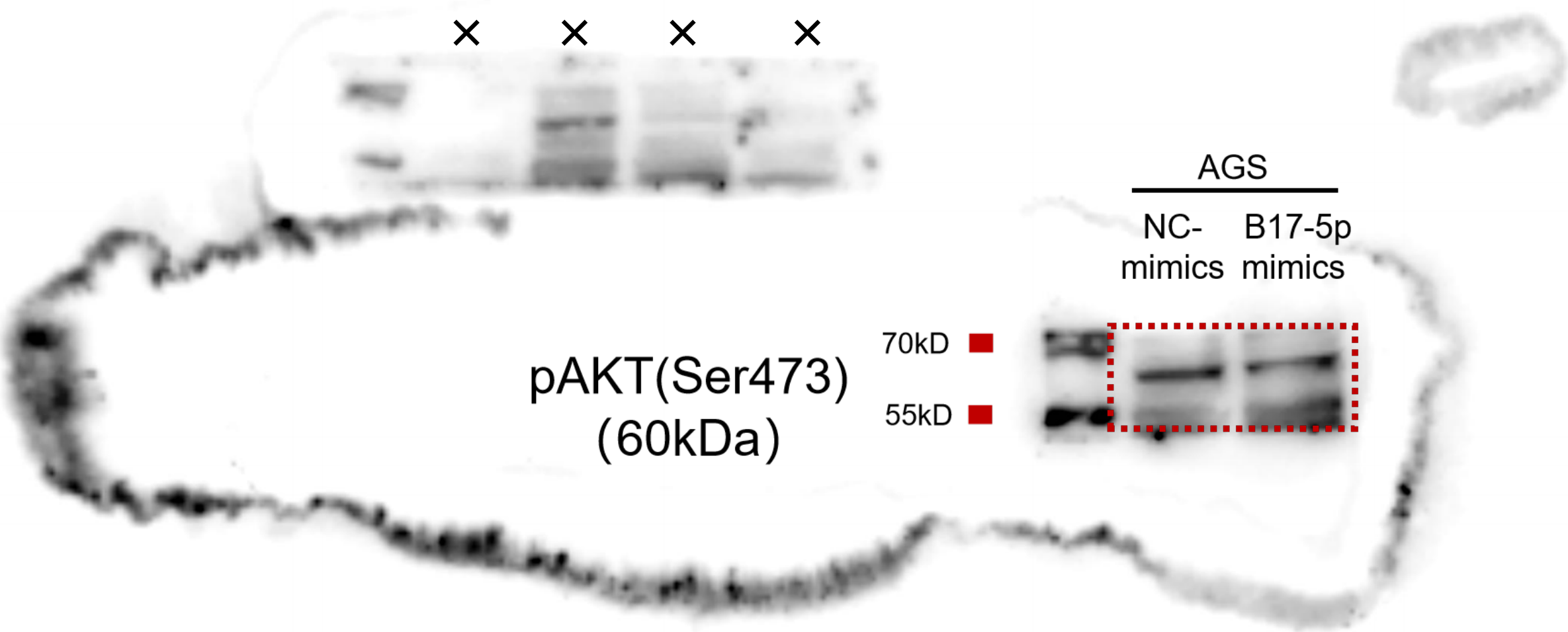

This image corresponds to Figure 6A

Biological Replicate #2

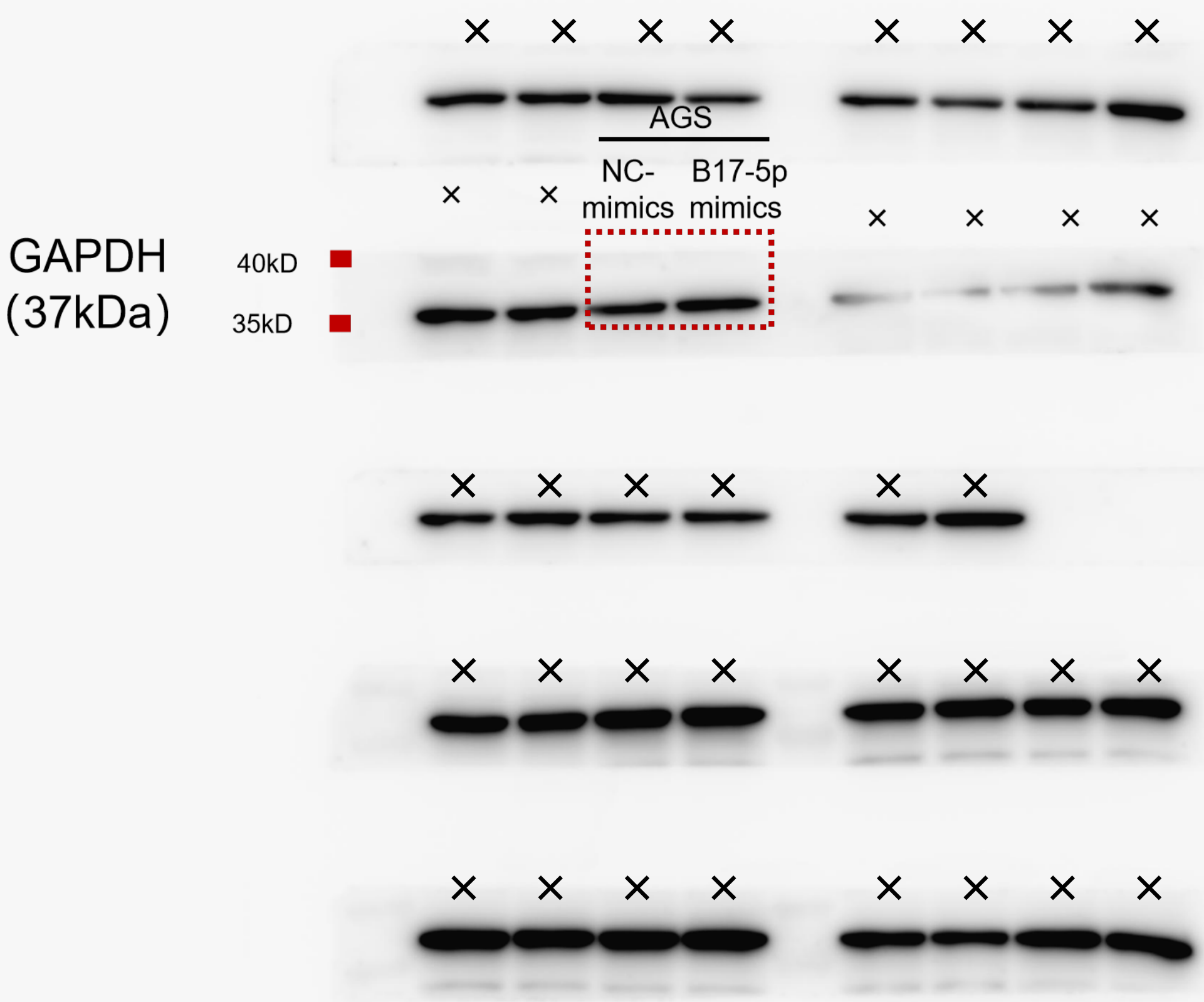

This image corresponds to Figure 6A

Biological Replicate #3

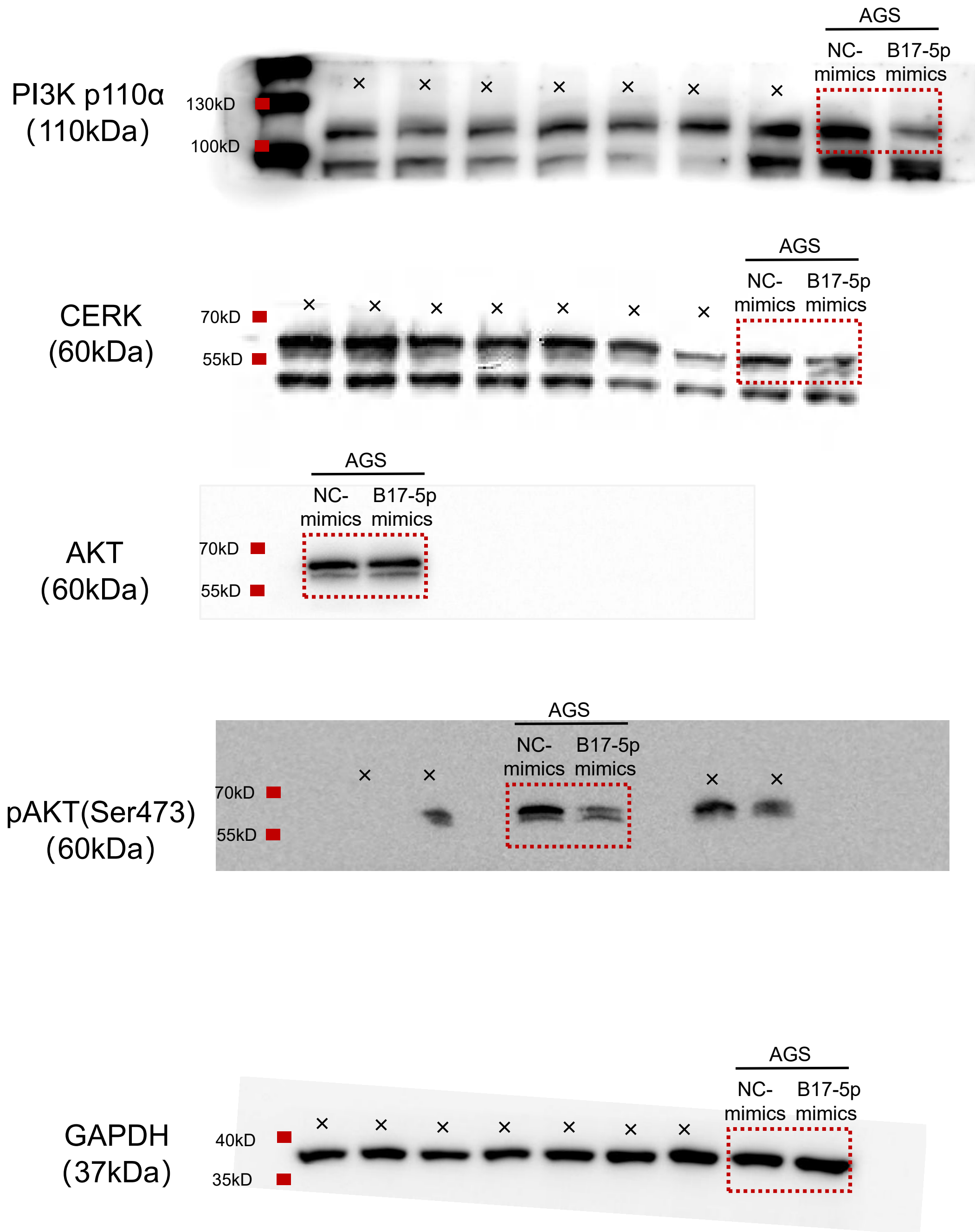

This image corresponds to Figure 6A

Biological Replicate #3

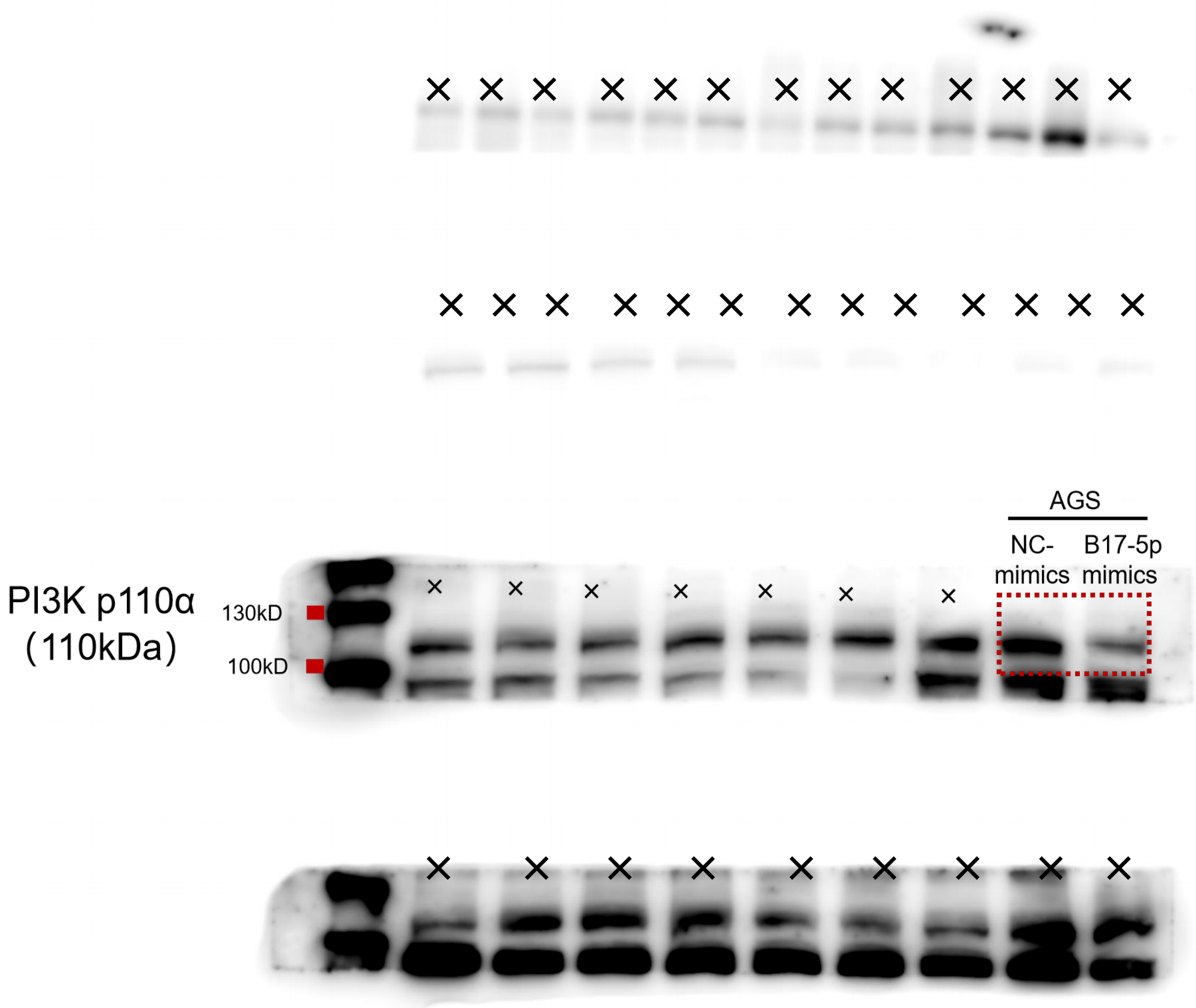

This image corresponds to Figure 6A

Biological Replicate #3

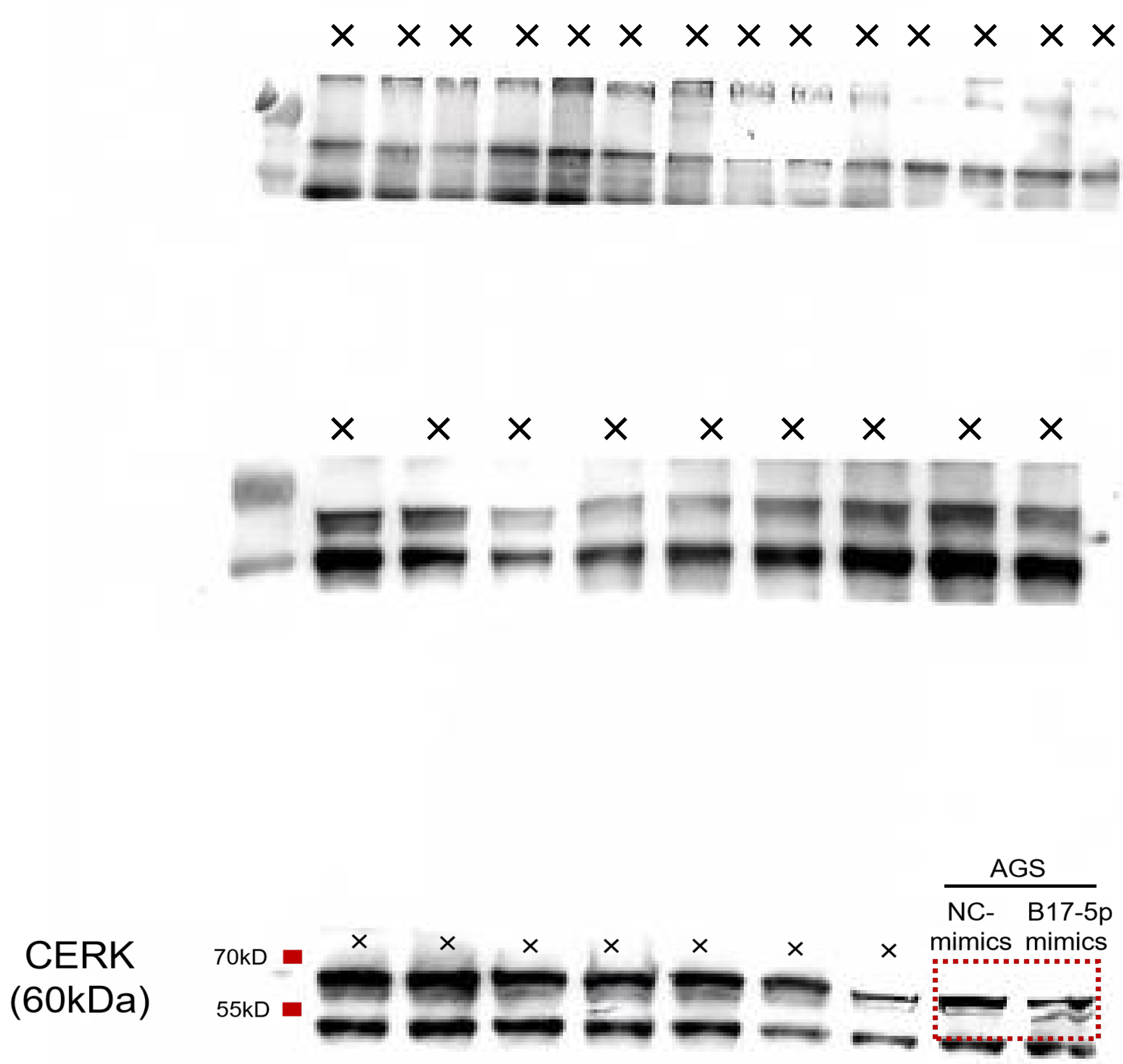

This image corresponds to Figure 6A

Biological Replicate #3

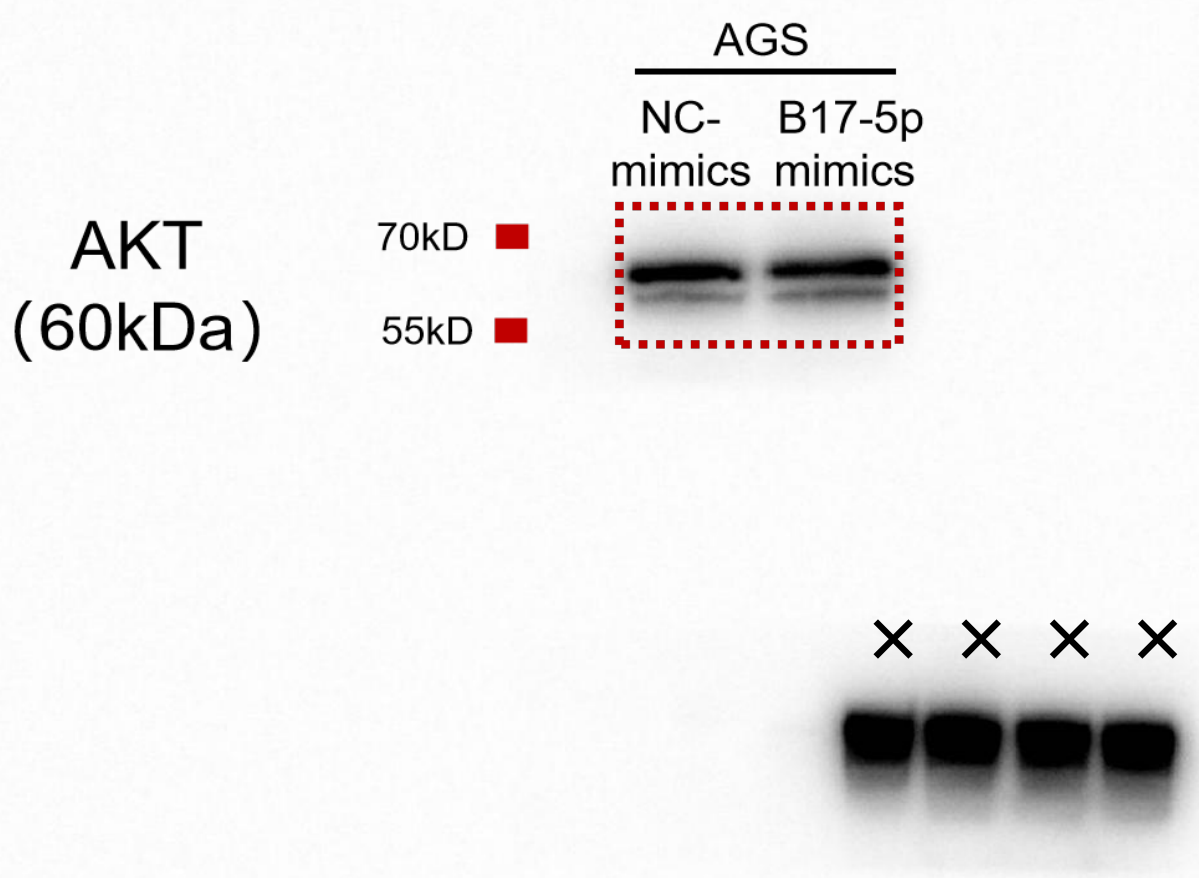

This image correspond to Figure 6A

Biological Replicate #3

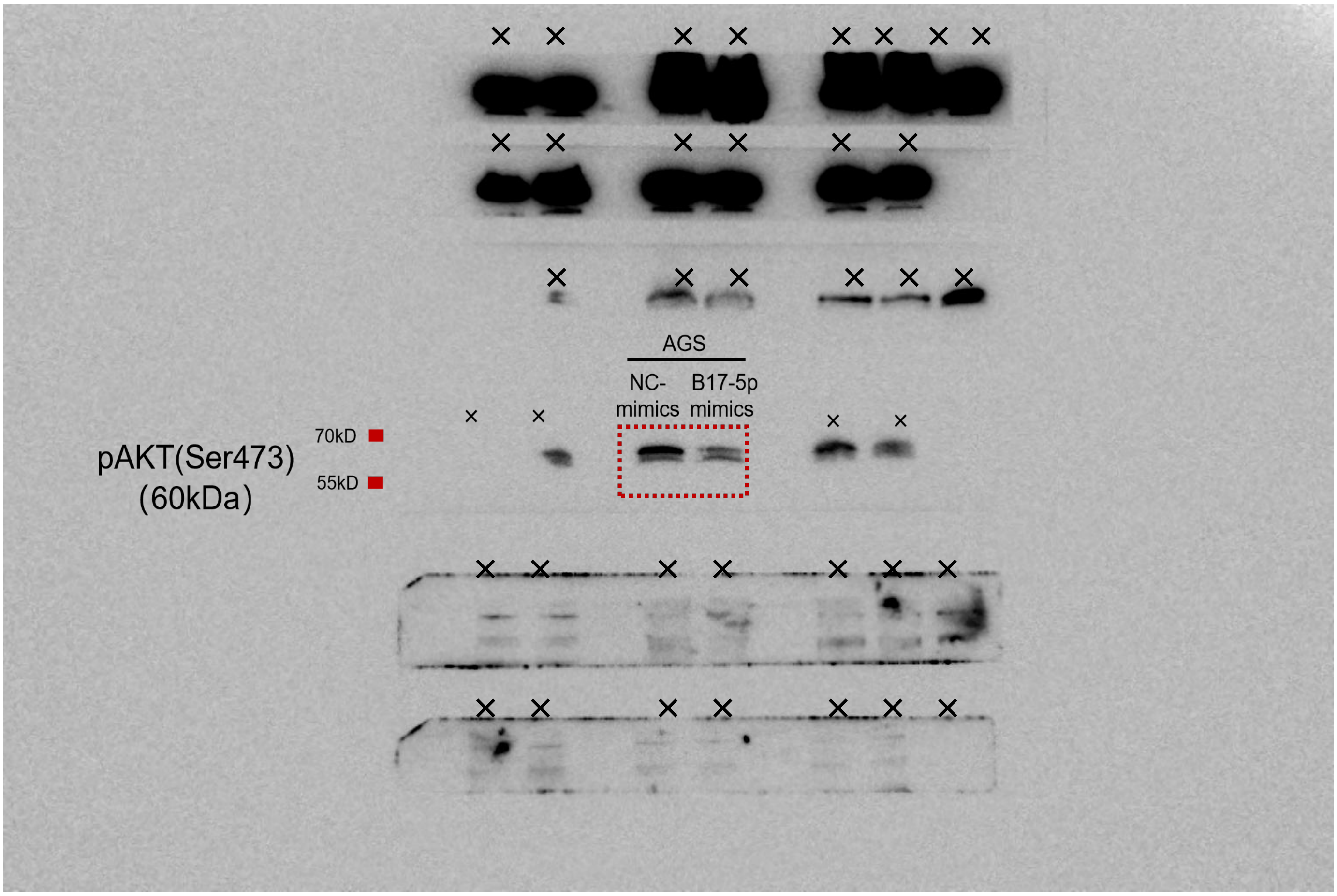

This image correspond to Figure 6A

Biological Replicate #3

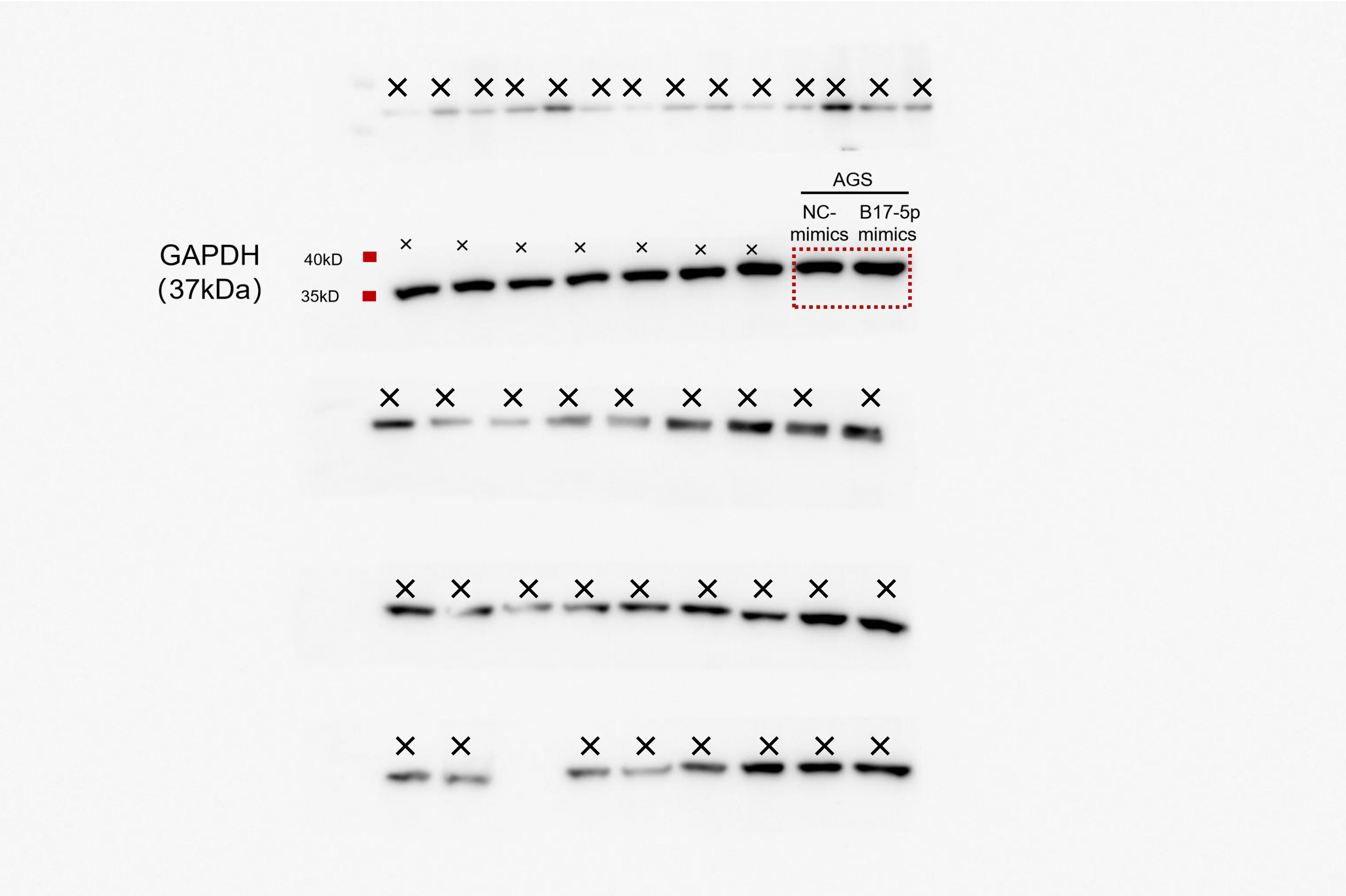

These images correspond to Figure 6C

Biological Replicate #1

▲ These images was selected as the representative result in Figure 6C.

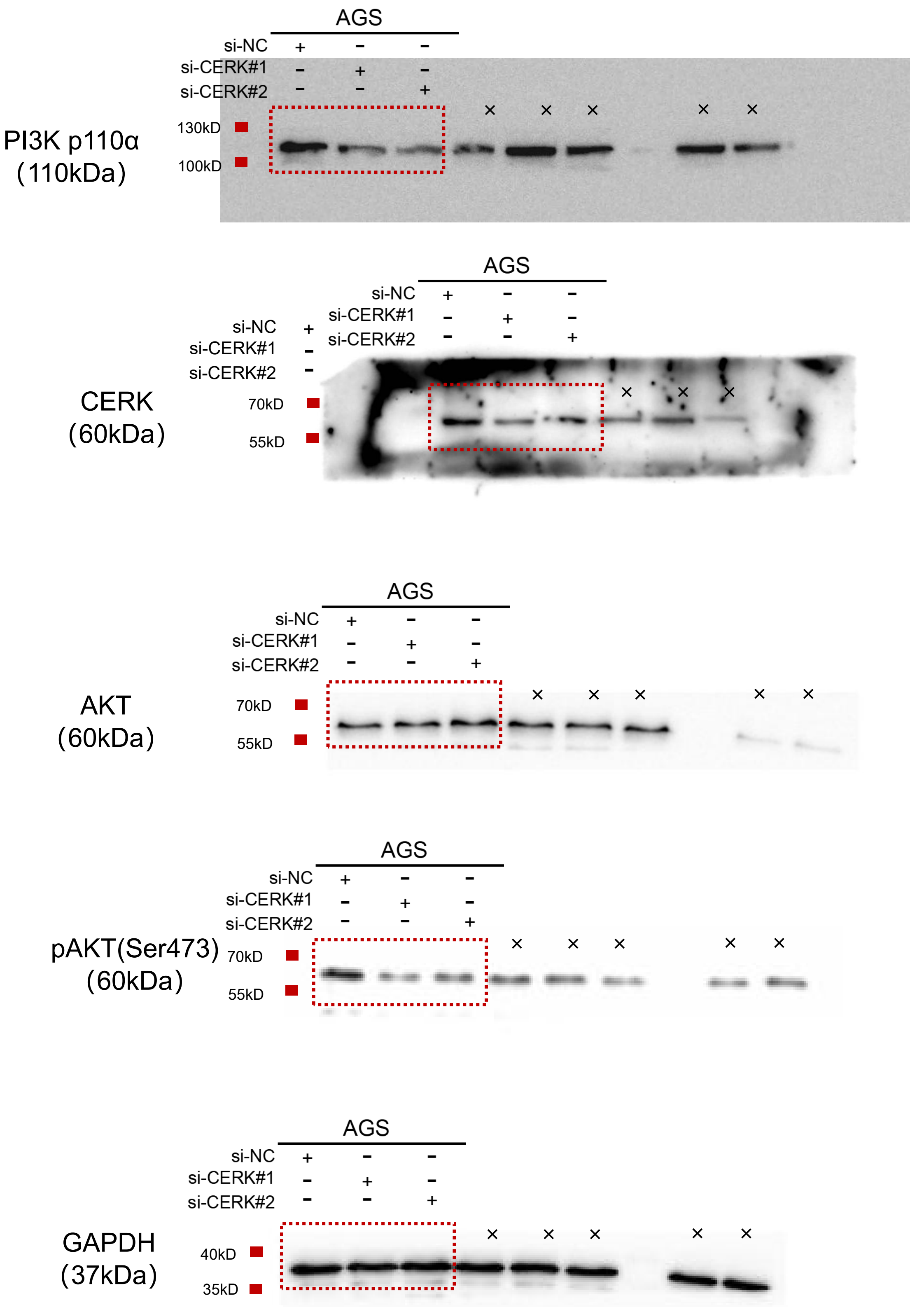

This image correspond s to Figure 6C

Biological Replicate #1

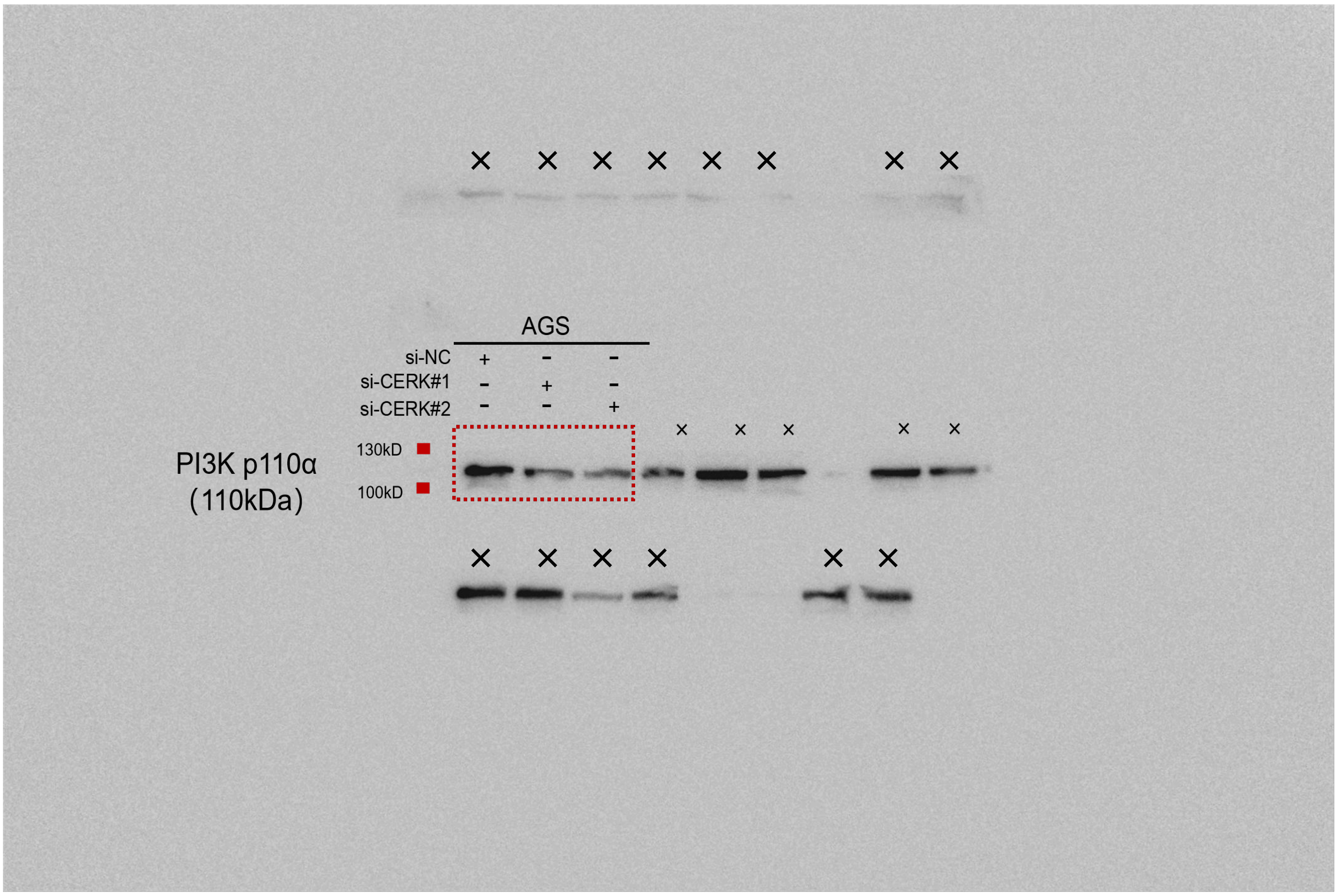

This image correspond s to Figure 6C

Biological Replicate #1

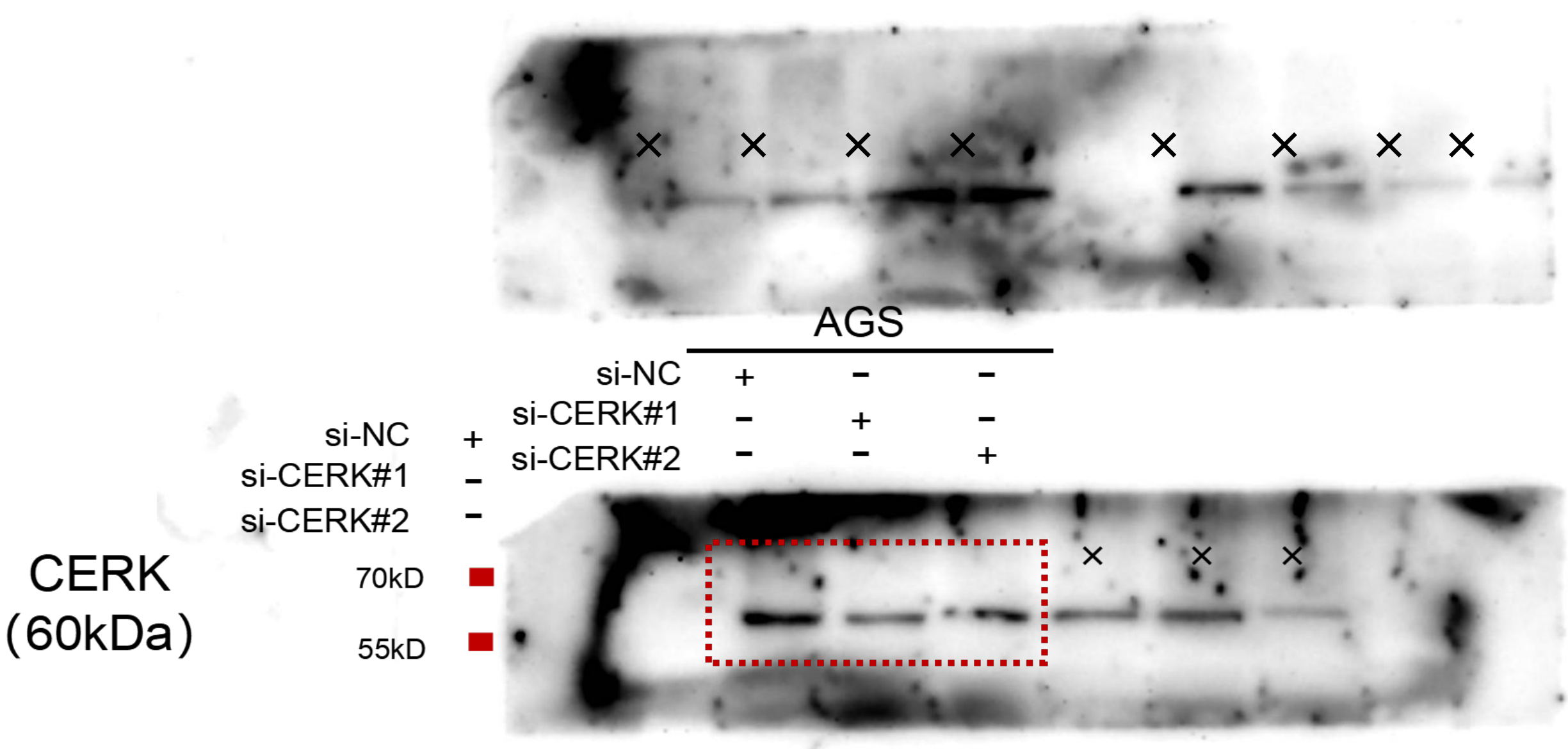

This image correspond s to Figure 6C

Biological Replicate #1

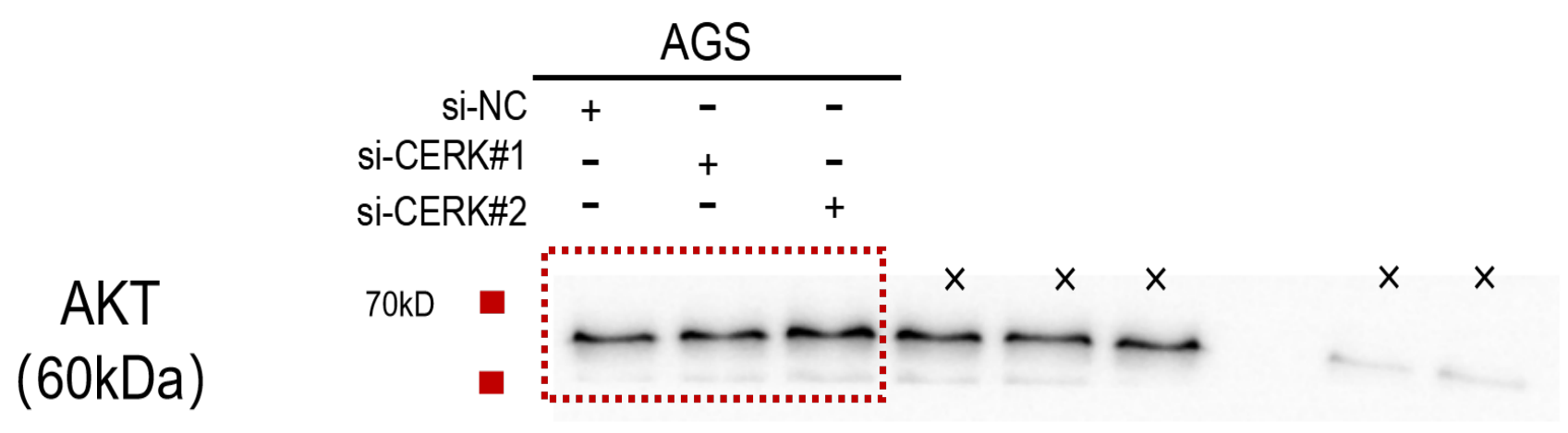

This image correspond s to Figure 6C

Biological Replicate #1

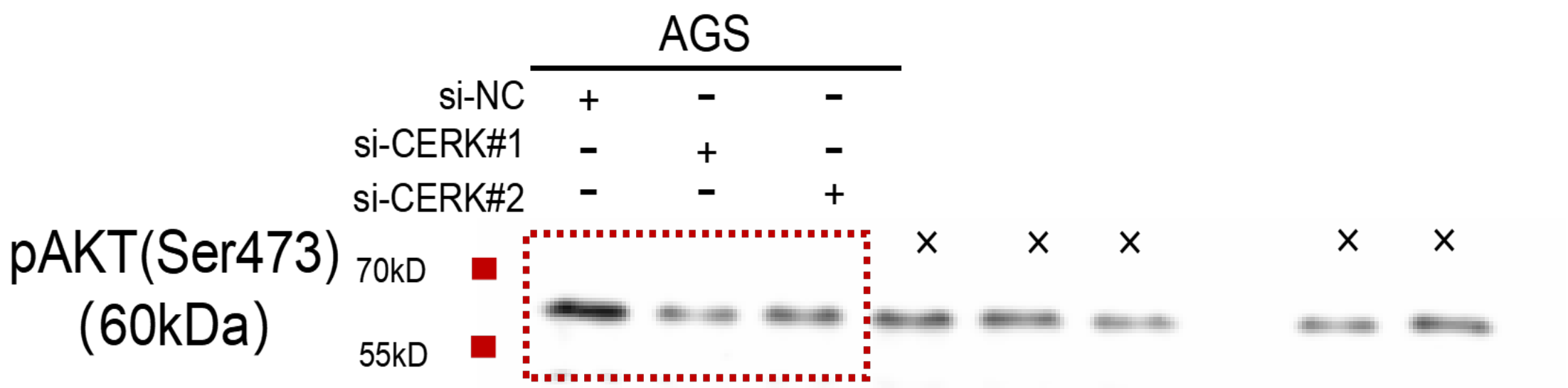

This image correspond s to Figure 6C

Biological Replicate #1

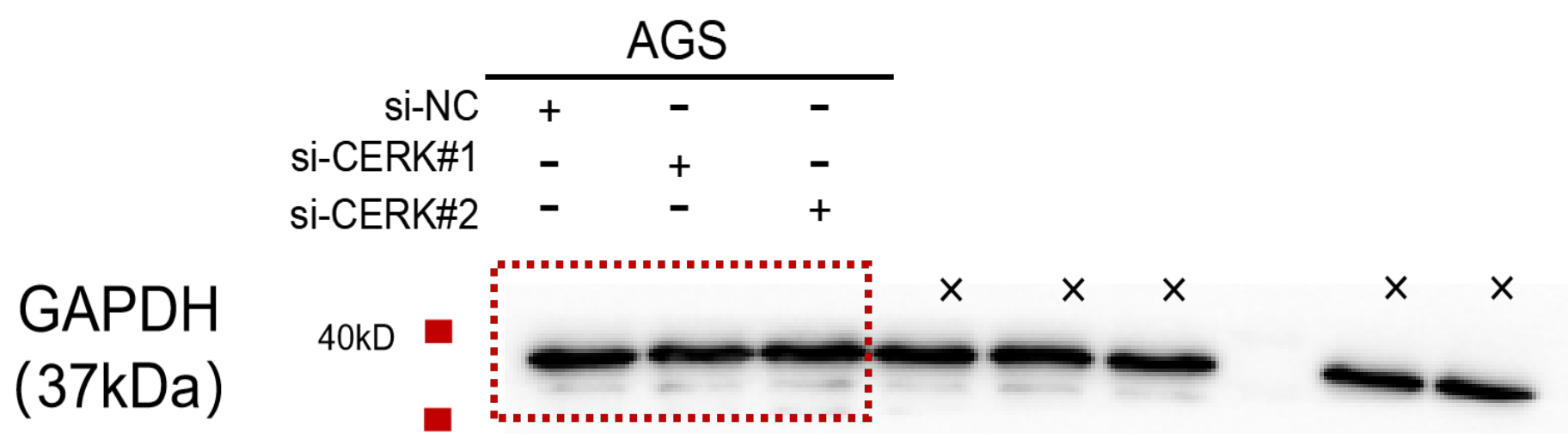

These images correspond to Figure 6C

Biological Replicate #2

PI3K p110α (110kDa)

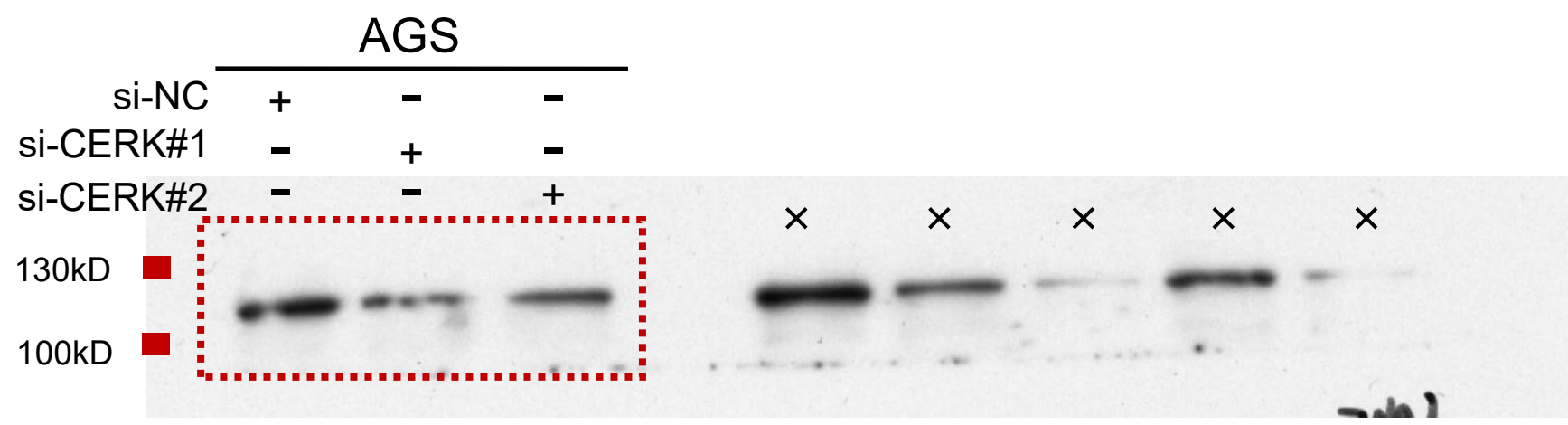

CERK (60kDa)

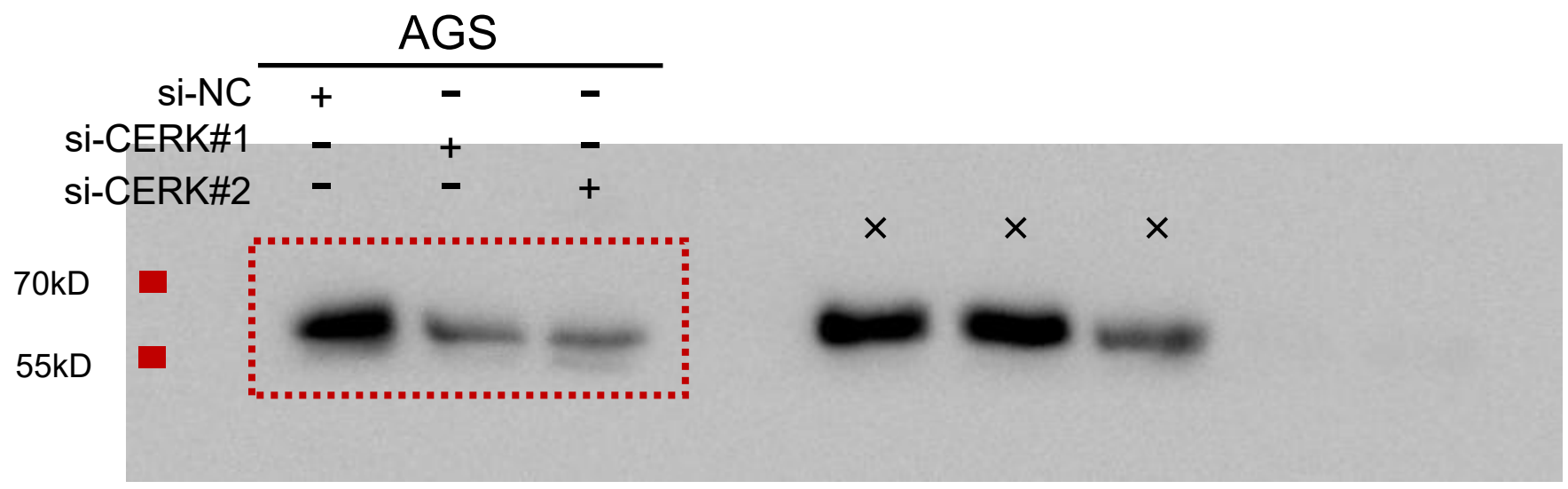

AKT (60kDa)

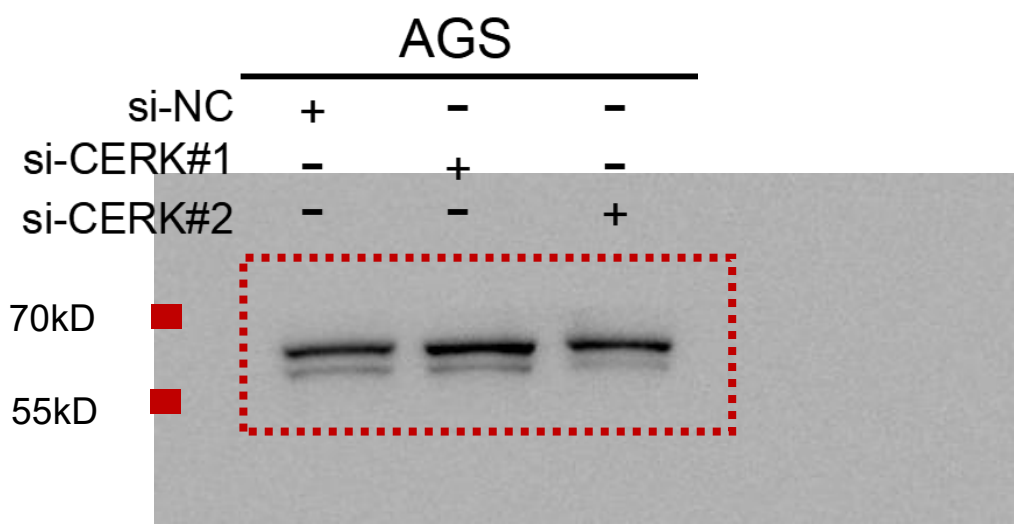

pAKT(Ser473) (60kDa)

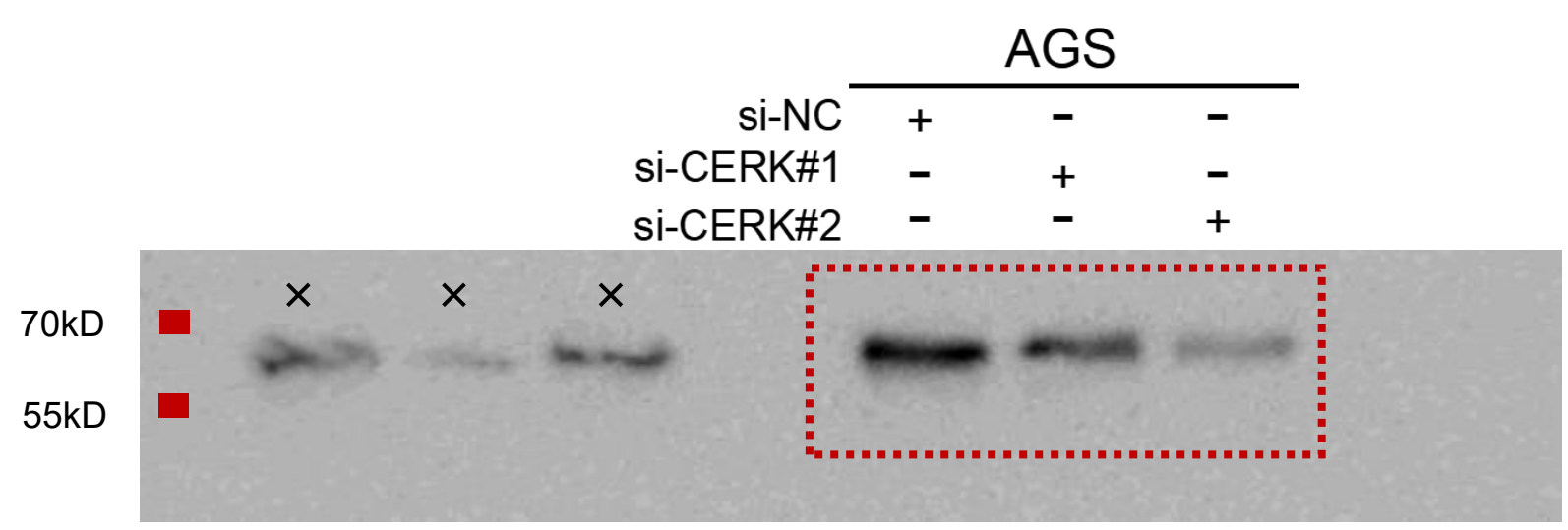

GAPDH (37kDa)

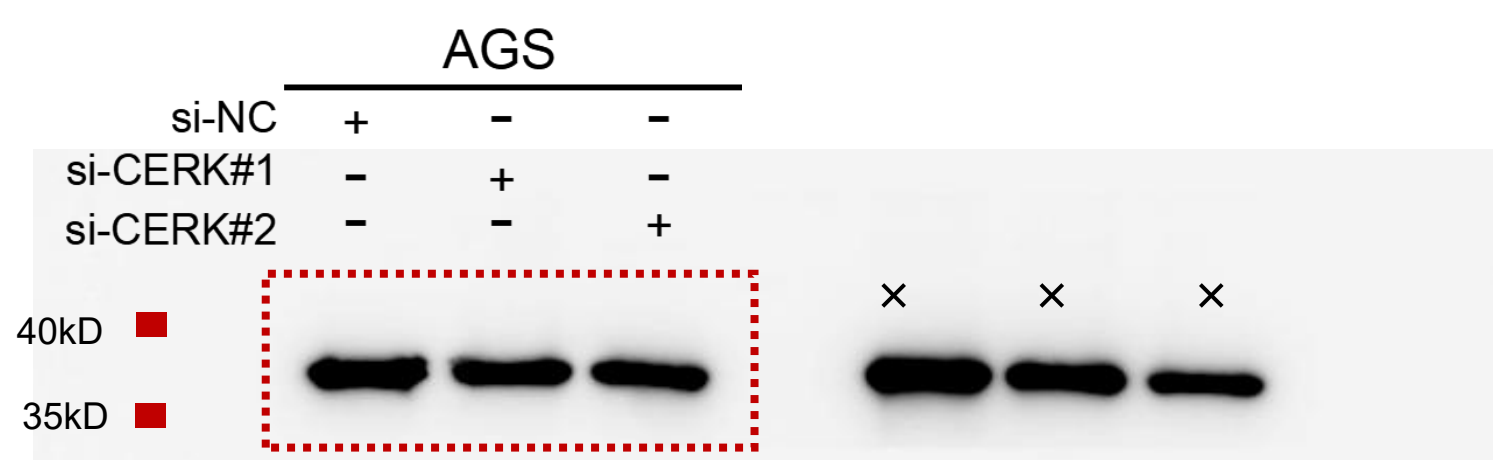

This image corresponds to Figure 6C

Biological Replicate #2

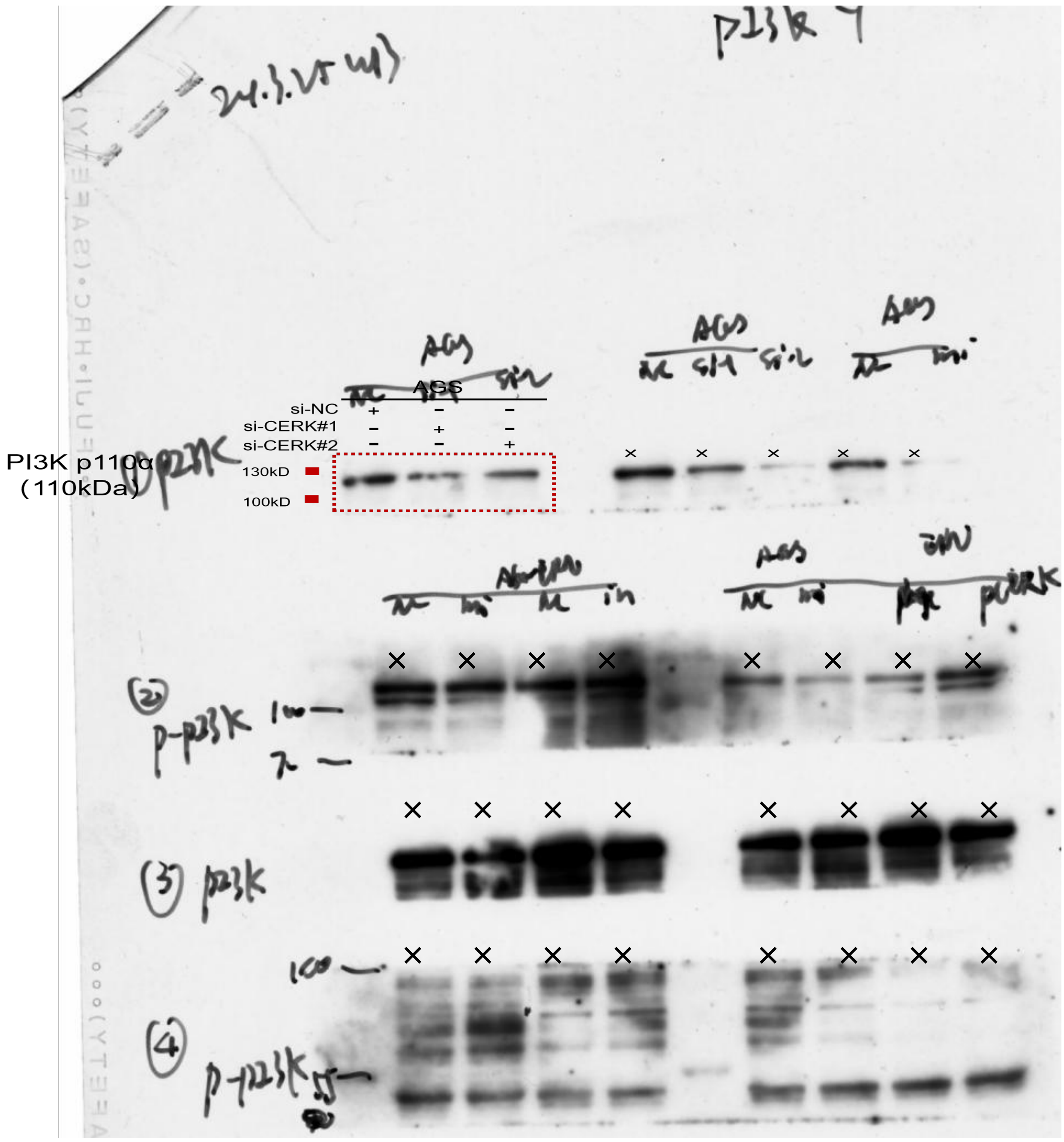

This image corresponds to Figure 6C

Biological Replicate #2

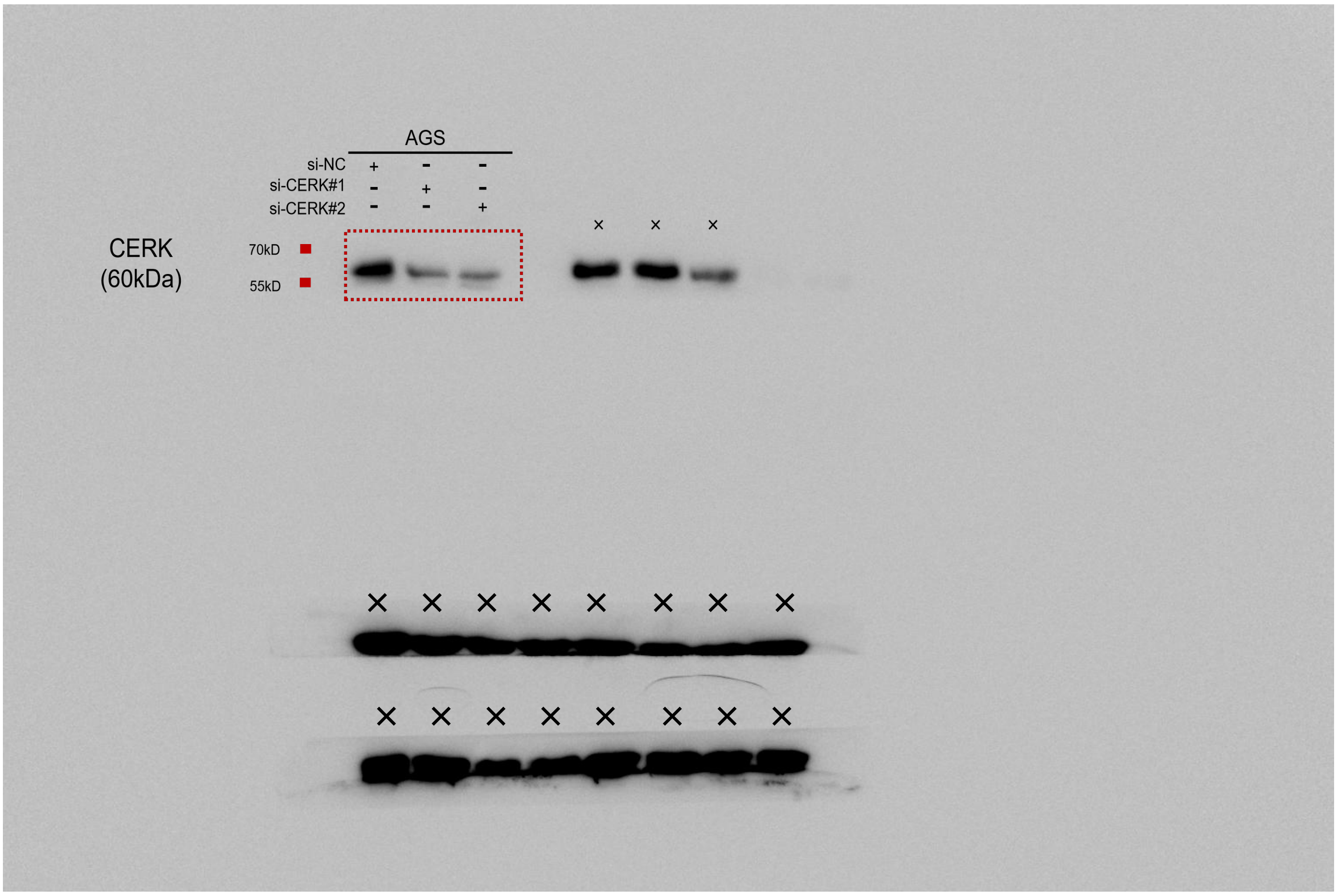

This image corresponds to Figure 6C

Biological Replicate #2

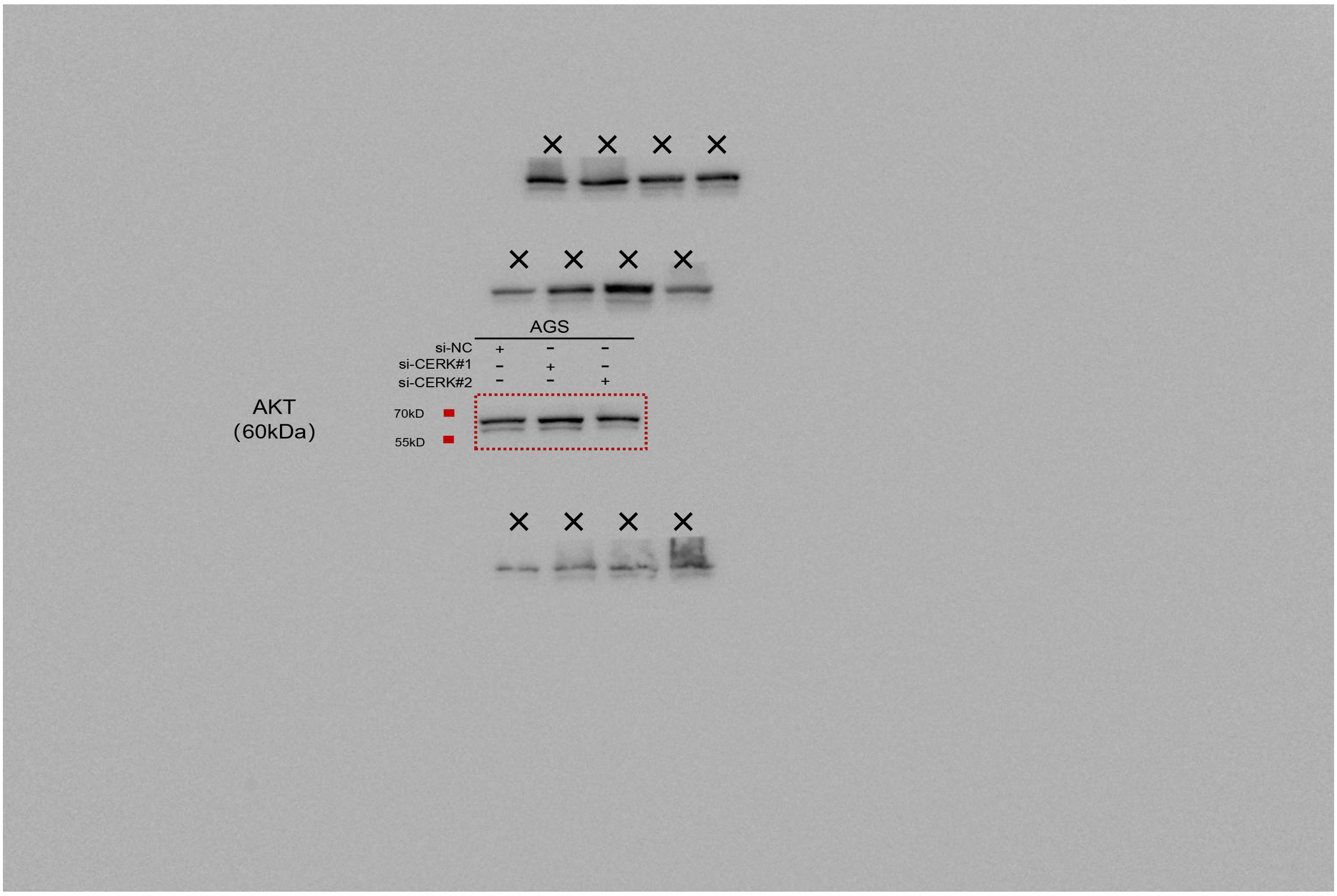

This image corresponds to Figure 6C

Biological Replicate #2

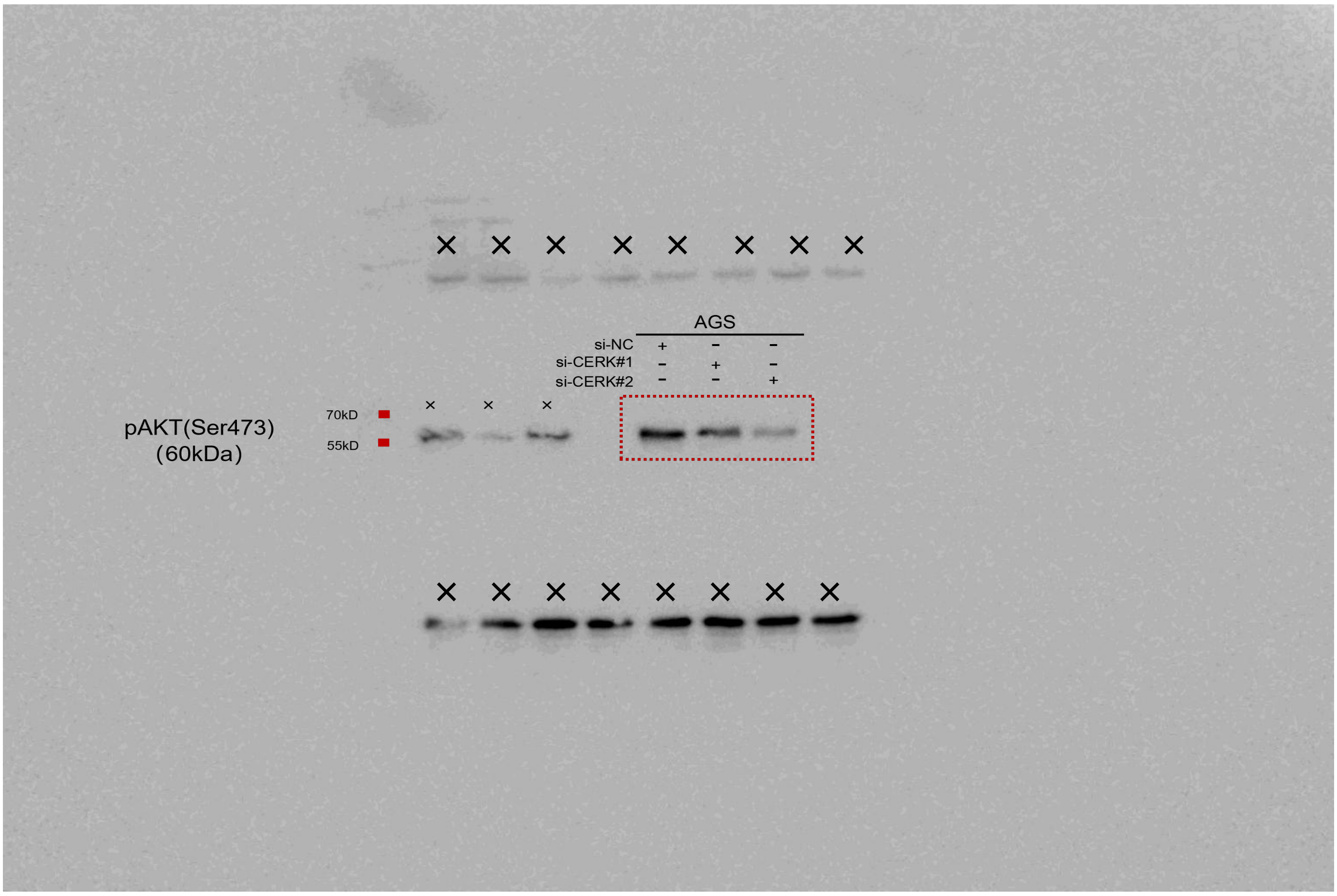

This image corresponds to Figure 6C

Biological Replicate #2

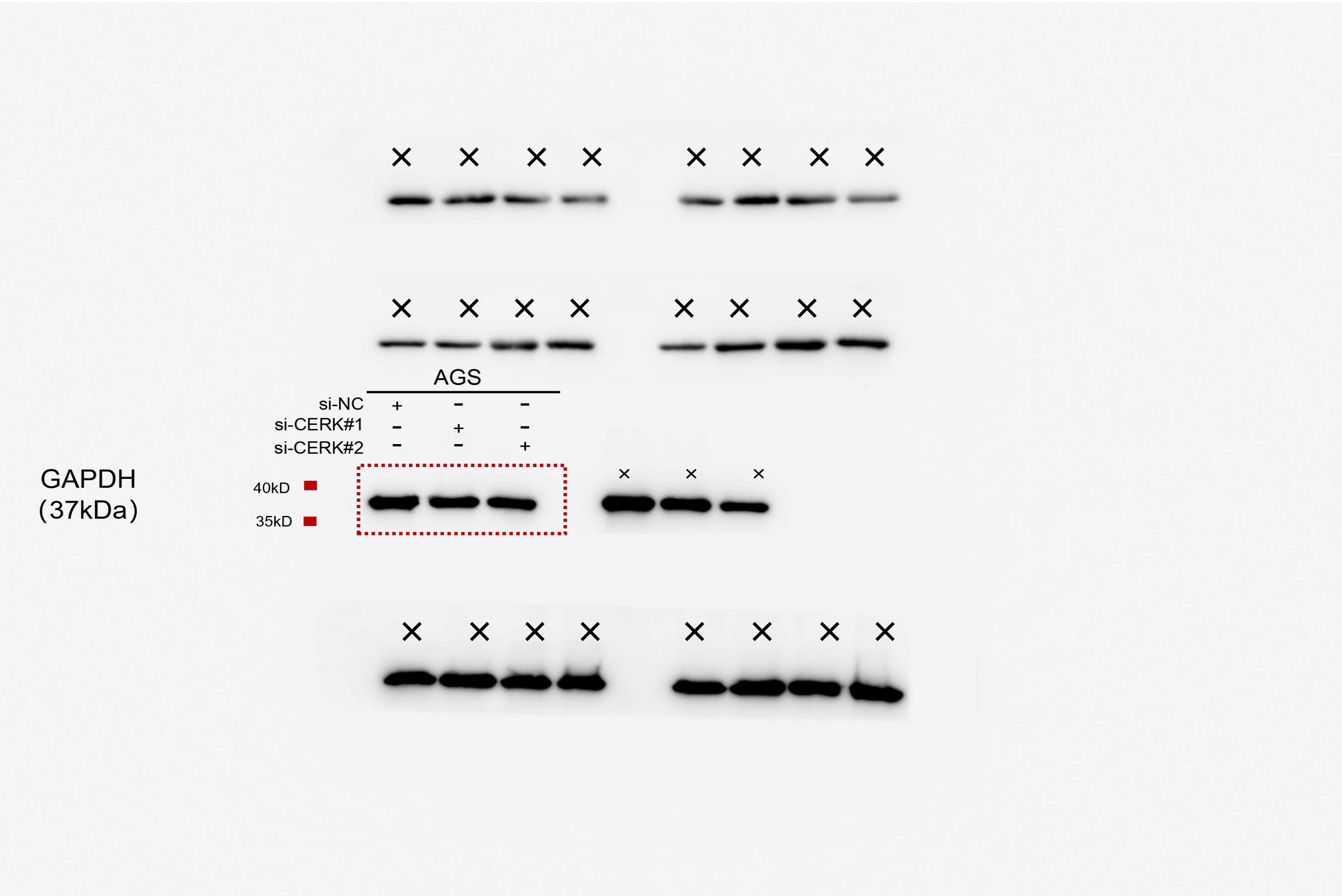

These images correspond to Figure 6C

Biological Replicate #3

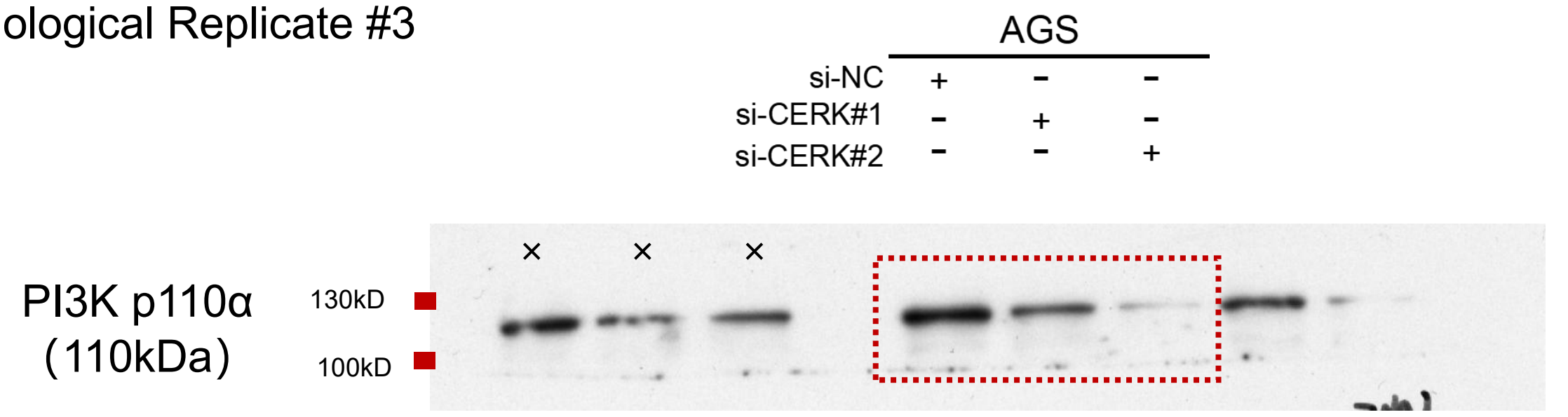

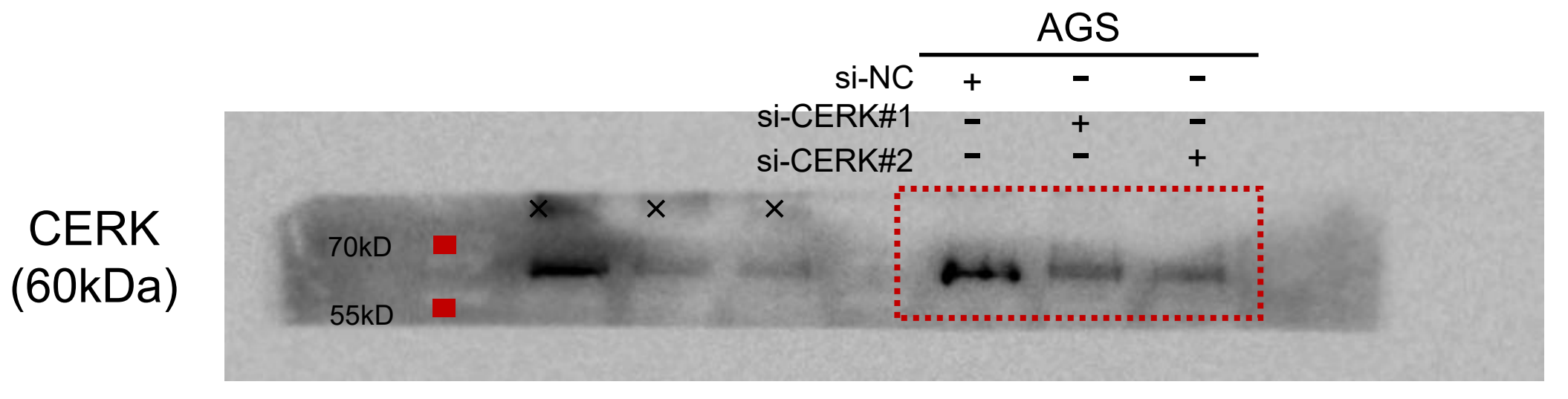

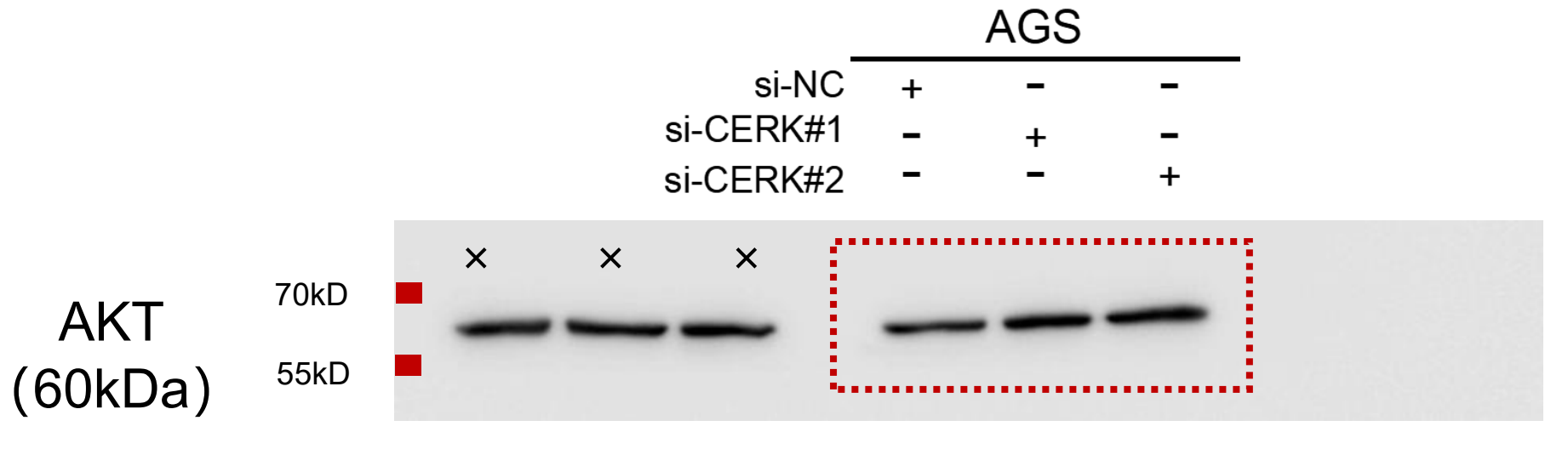

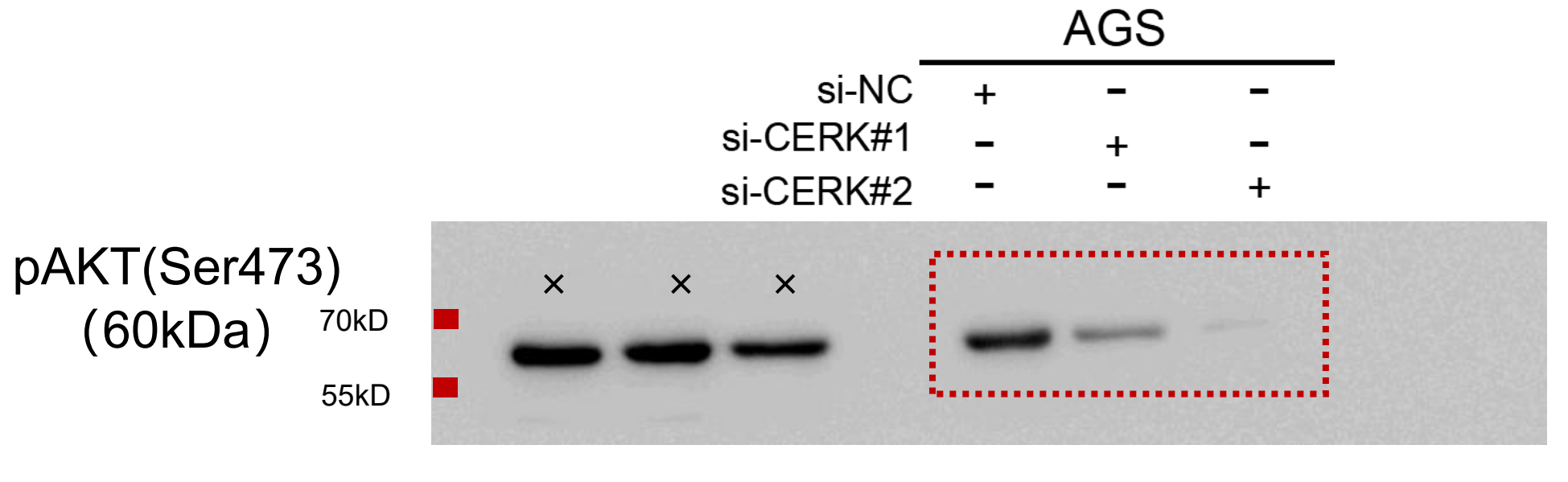

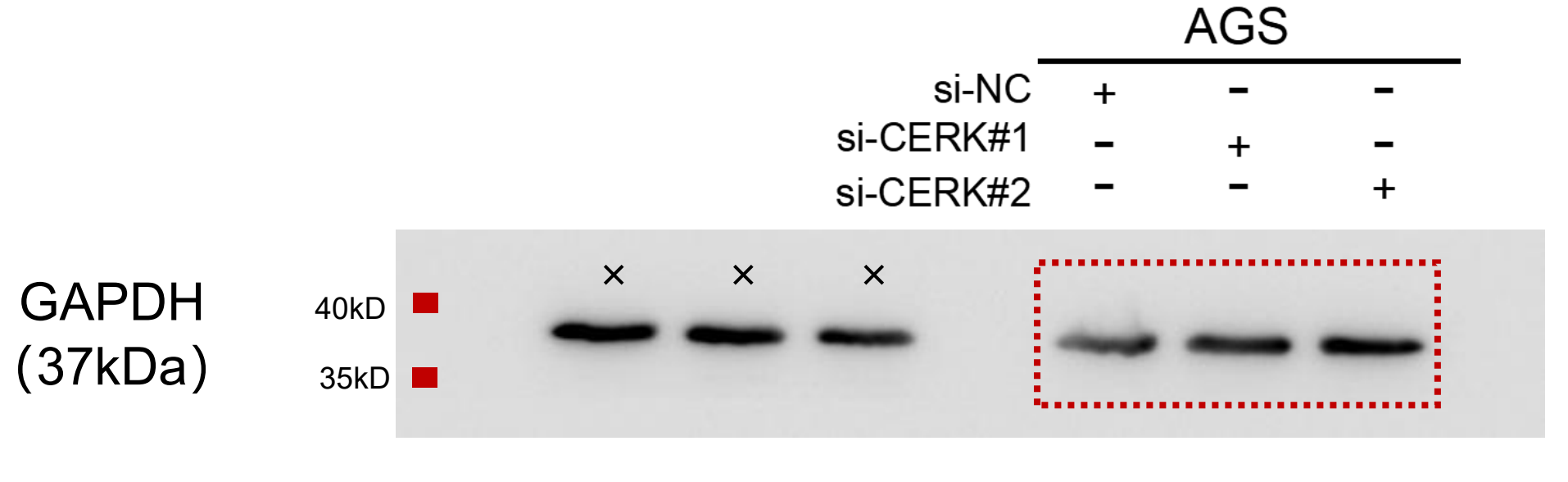

This image corresponds to Figure 6C

Biological Replicate #3

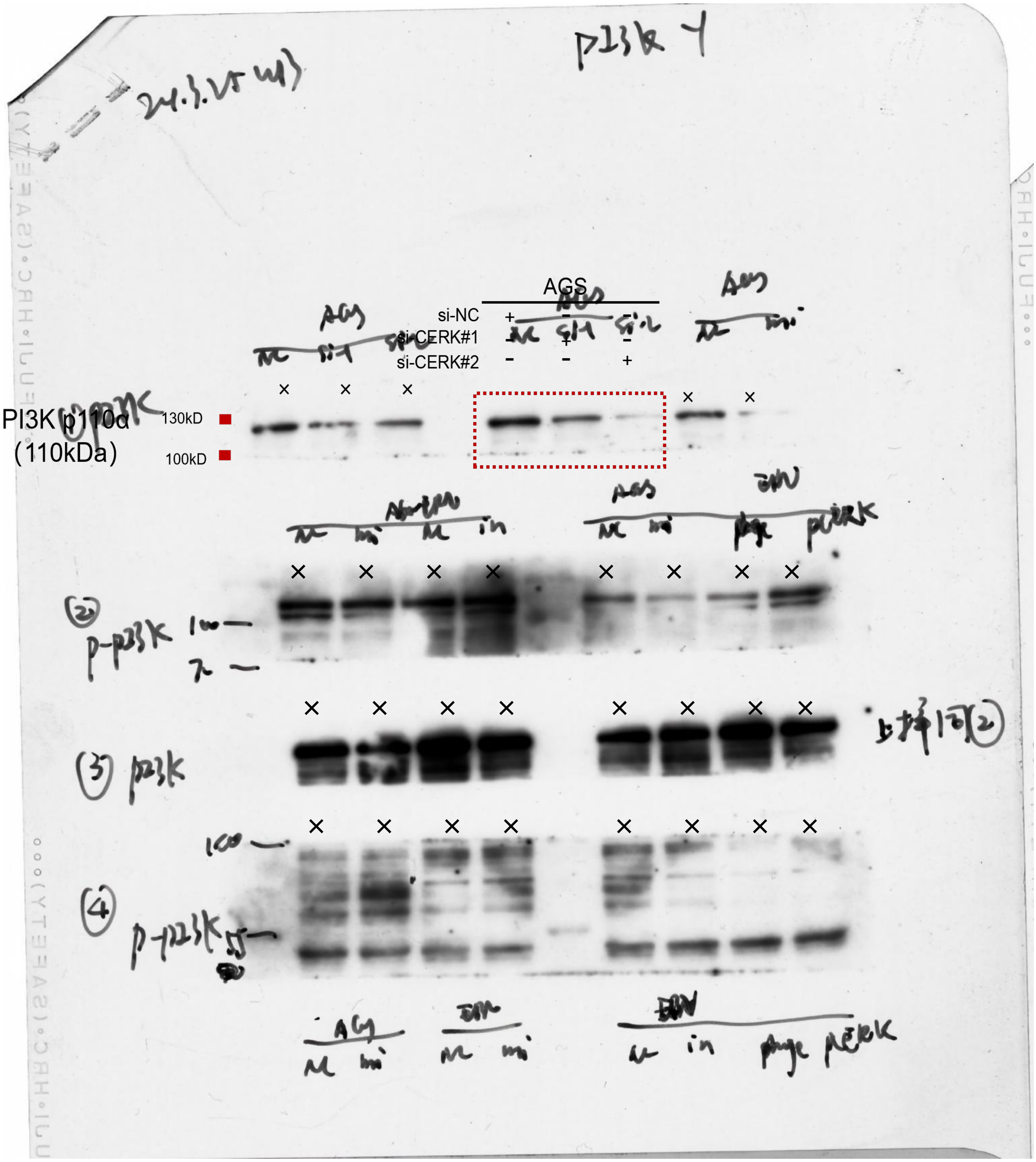

This image corresponds to Figure 6C

Biological Replicate #3

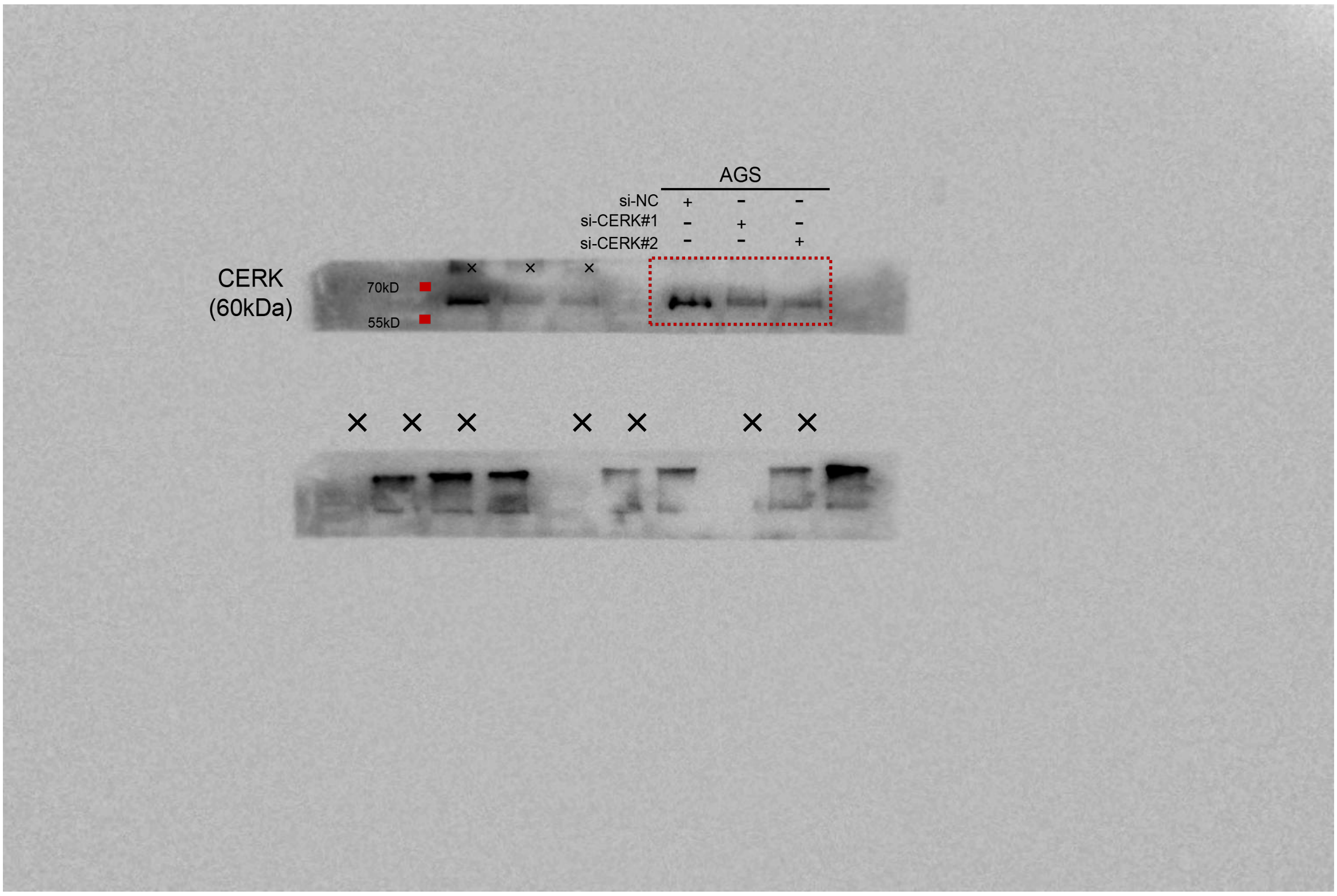

This image corresponds to Figure 6C

Biological Replicate #3

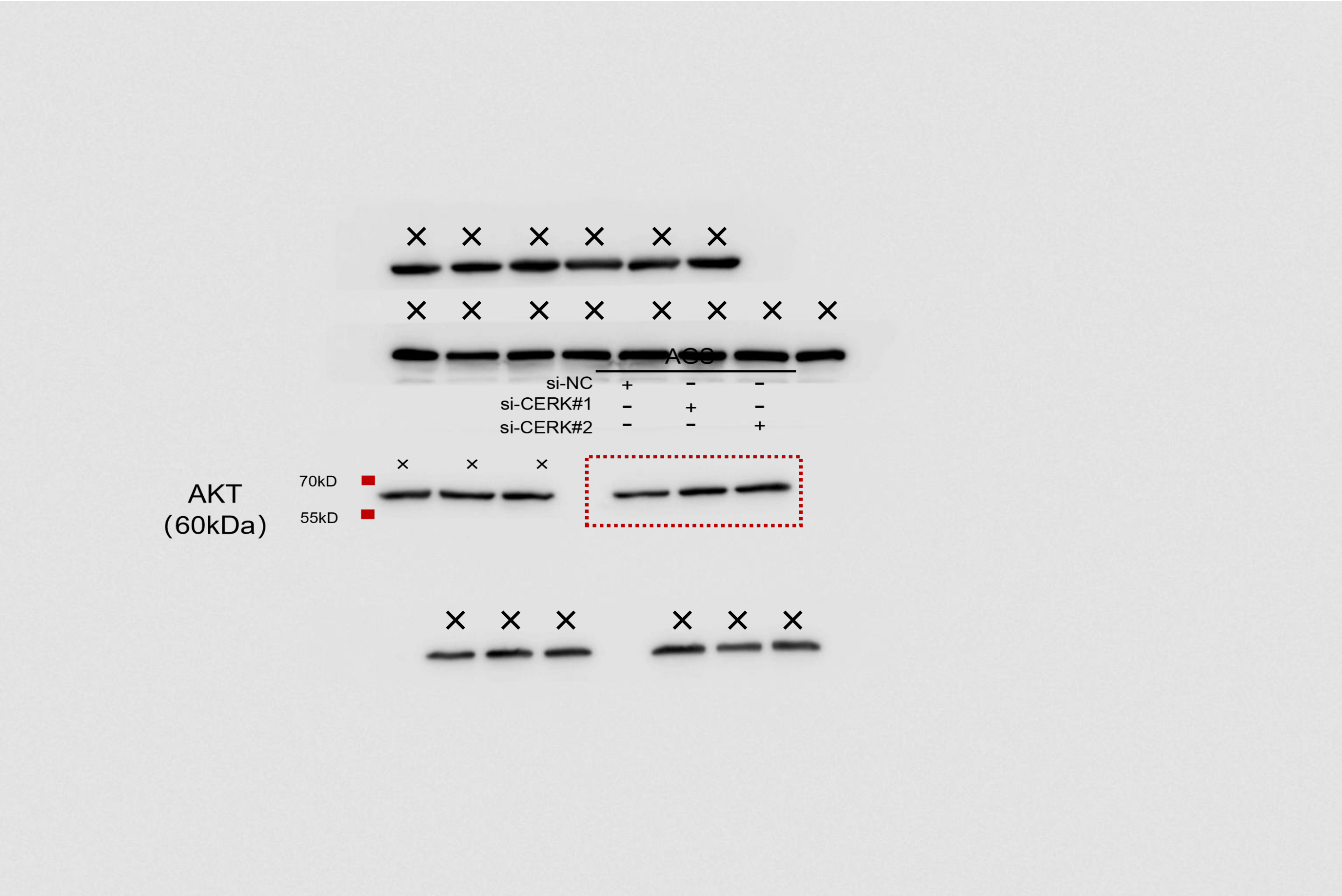

This image corresponds to Figure 6C

Biological Replicate #3

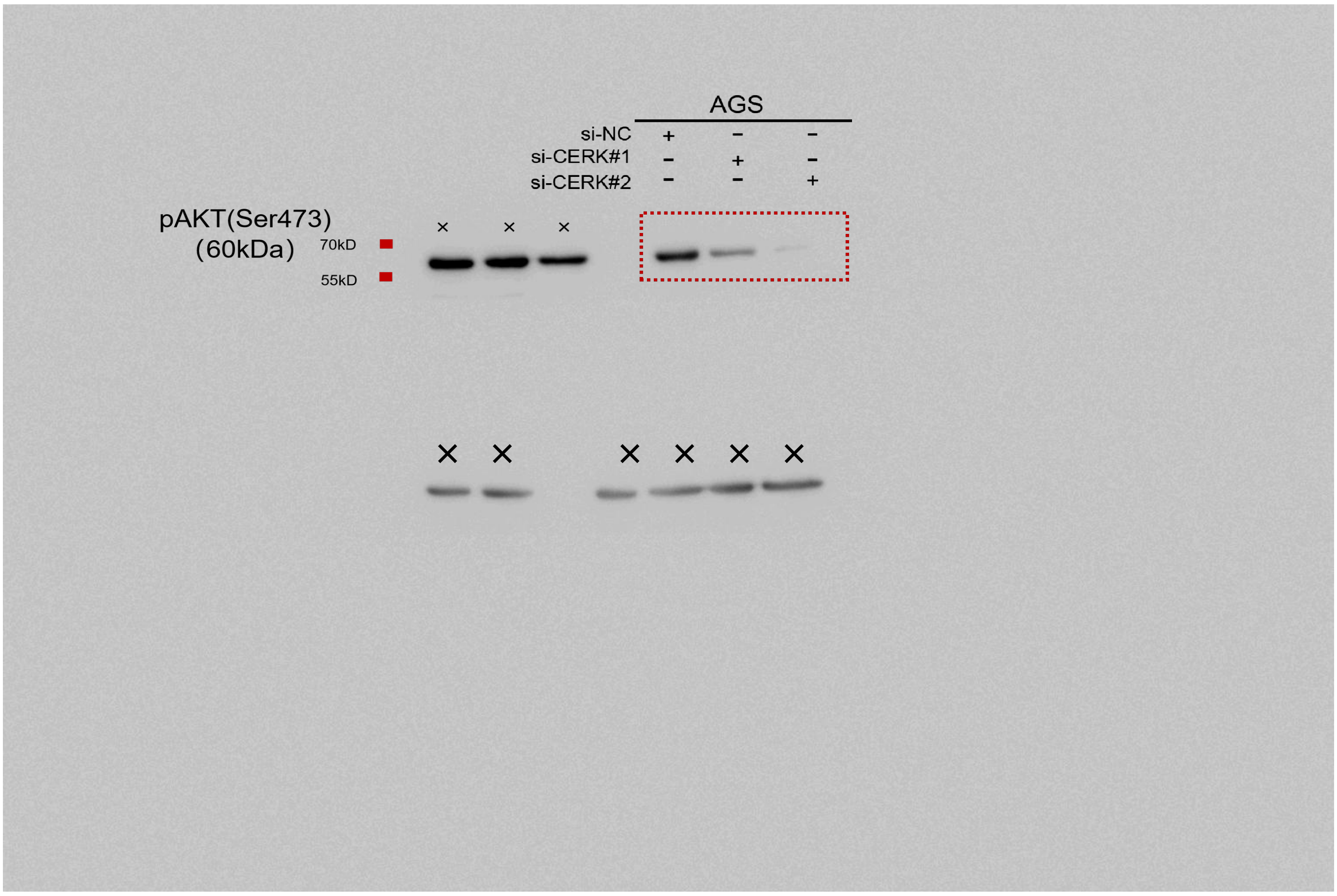

This image corresponds to Figure 6C

Biological Replicate #3

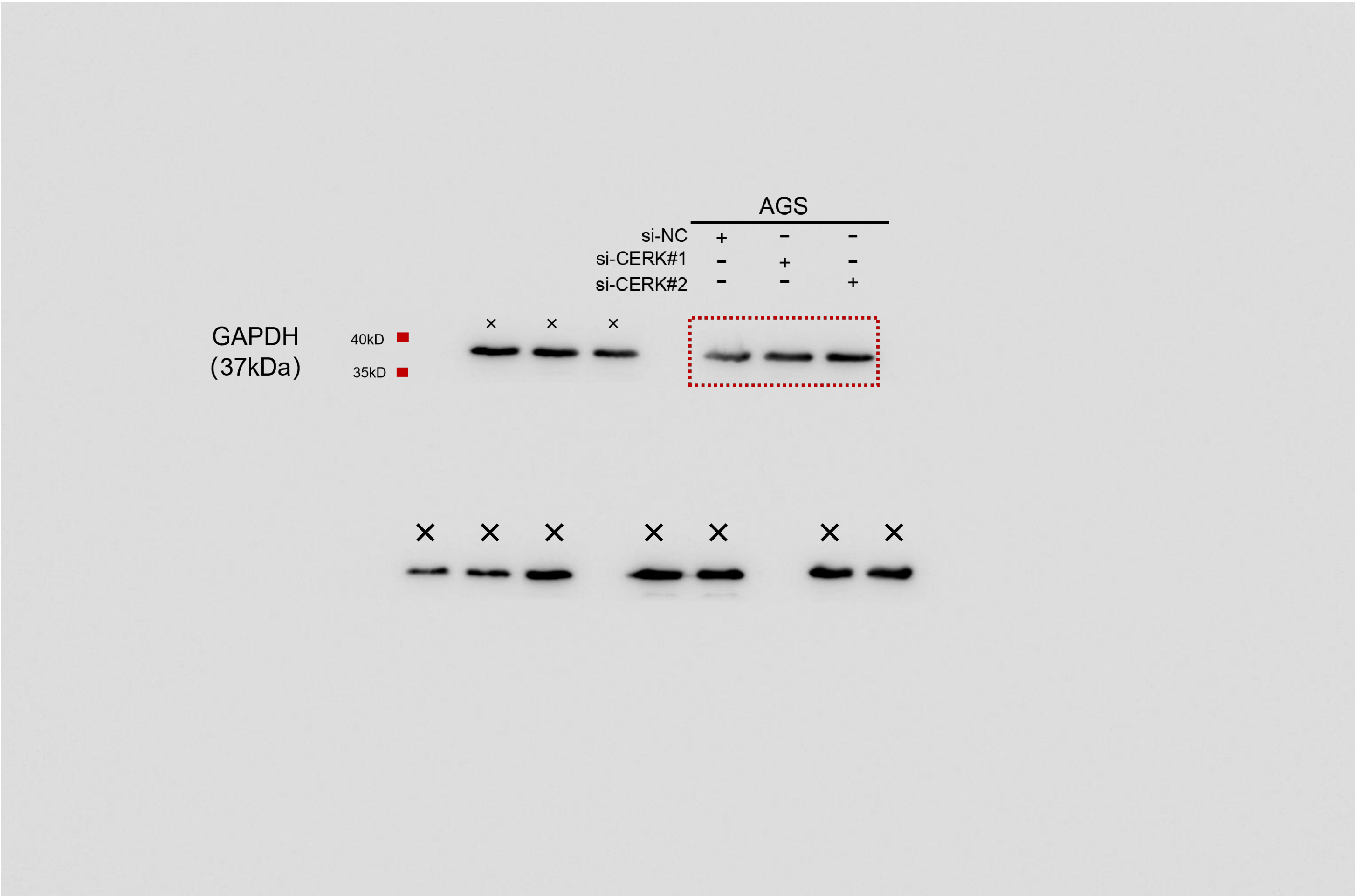

These images correspond to Figure 6E

Biological Replicate #1

PI3K p110α
(110kDa)

AGS-EBV
NC- inhibitor
B17-5p inhibitor

130kD
100kD

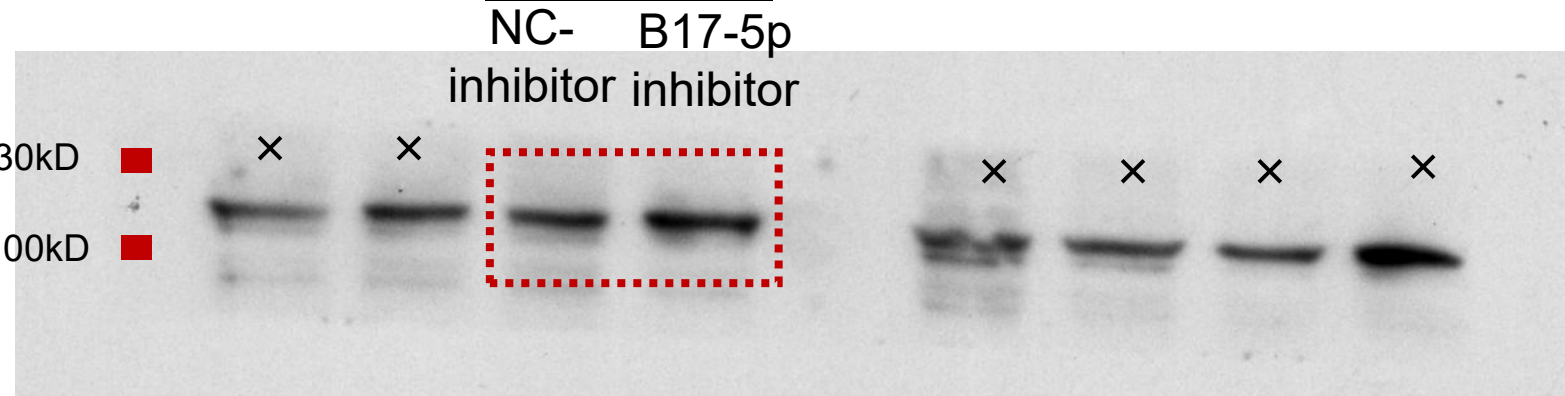

CERK
(60kDa)

AGS-EBV
NC- inhibitor
B17-5p inhibitor

70kD
55kD

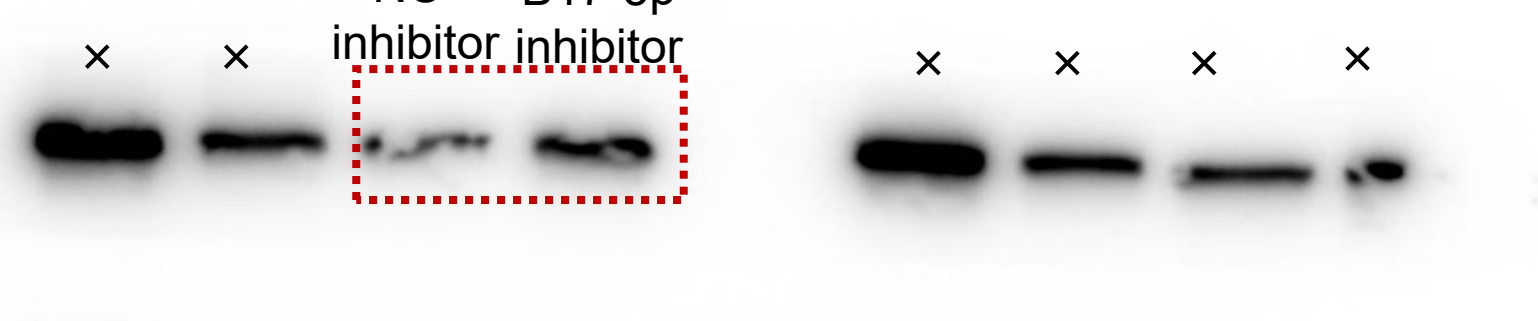

AKT
(60kDa)

AGS-EBV
NC- inhibitor
B17-5p inhibitor

70kD
55kD

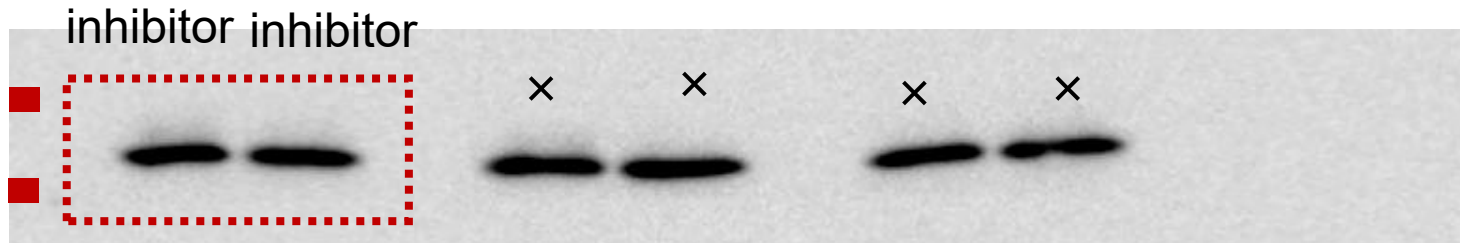

pAKT(Ser473)
(60kDa)

AGS-EBV
NC- inhibitor
B17-5p inhibitor

70kD
55kD

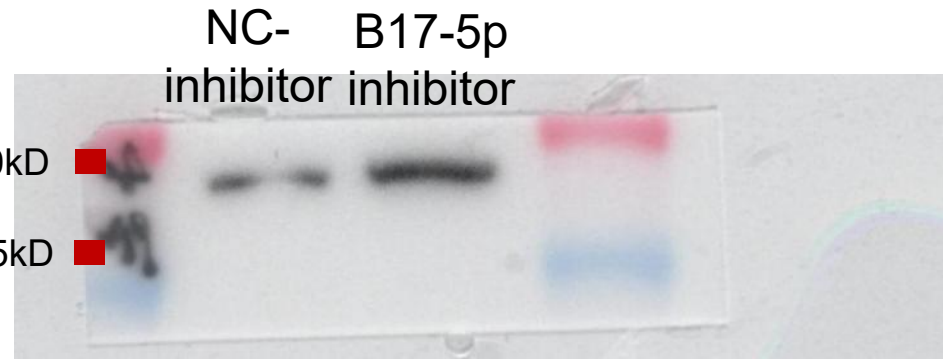

GAPDH
(37kDa)

AGS-EBV
NC- inhibitor
B17-5p inhibitor

40kD
35kD

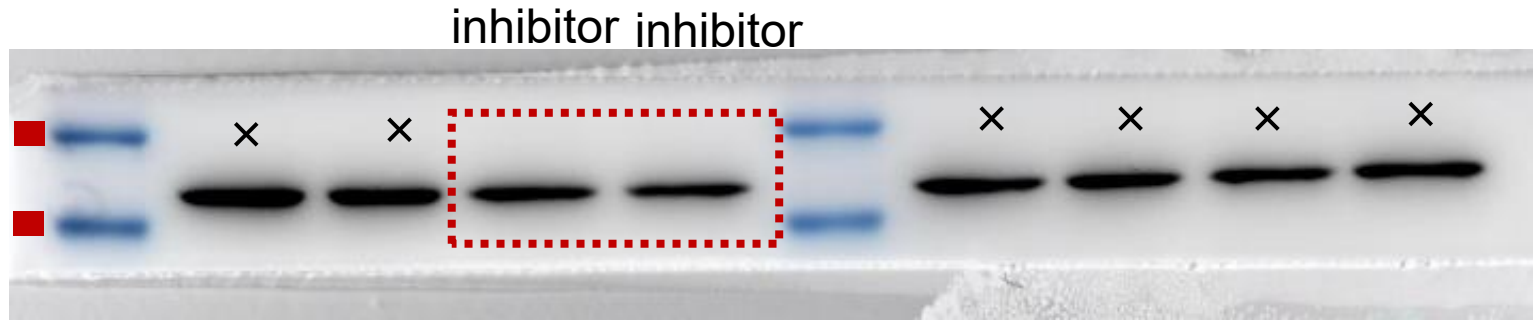

This imagecorresponds to Figure 6E

Biological Replicate #1

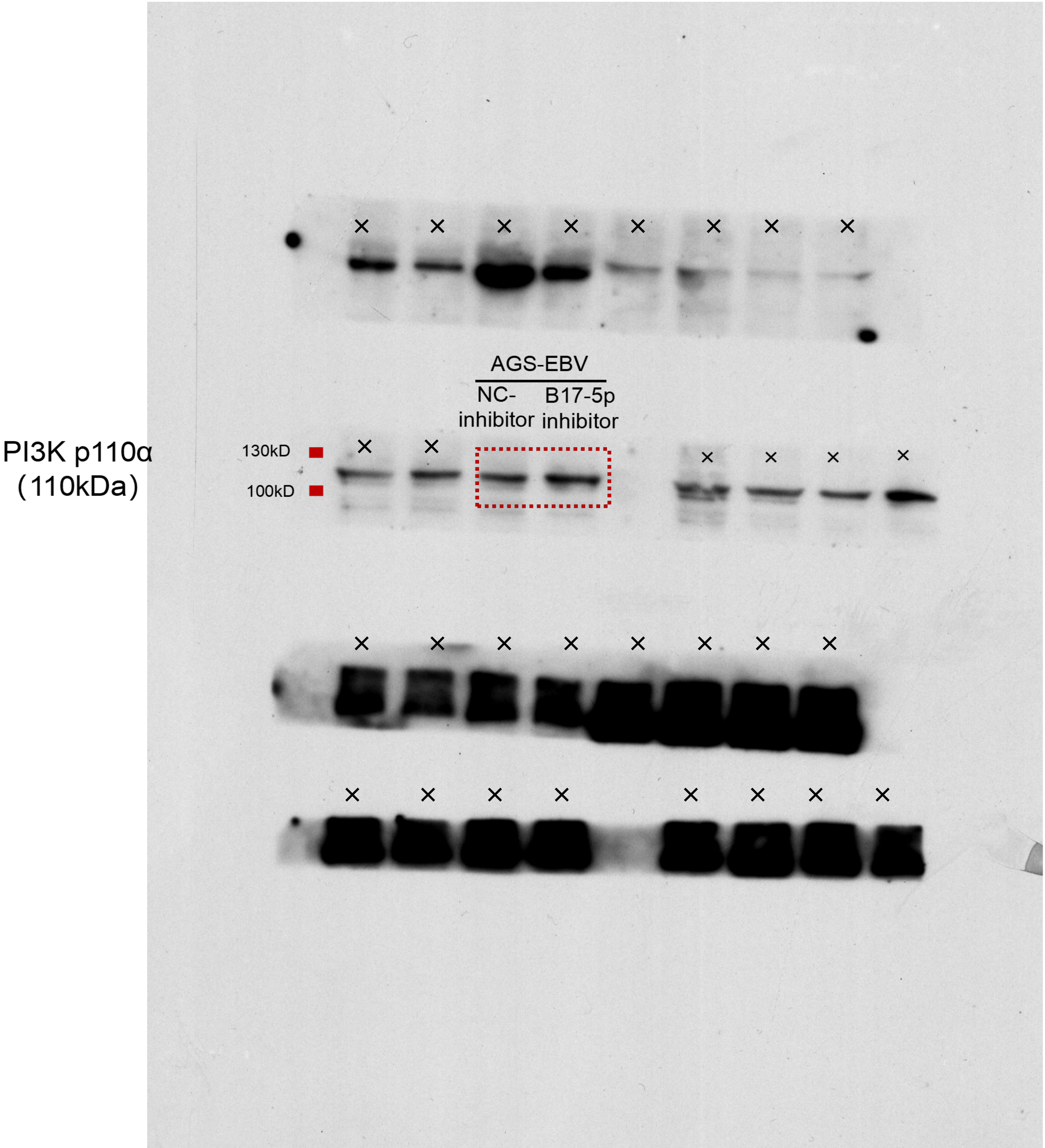

This imagecorresponds to Figure 6E

Biological Replicate #1

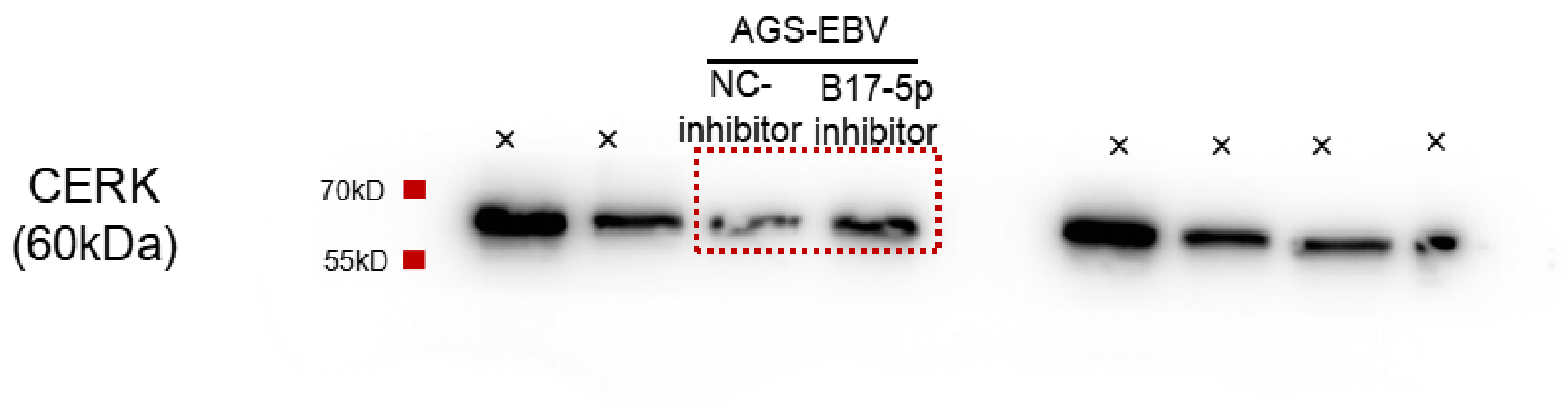

This imagecorresponds to Figure 6E

Biological Replicate #1

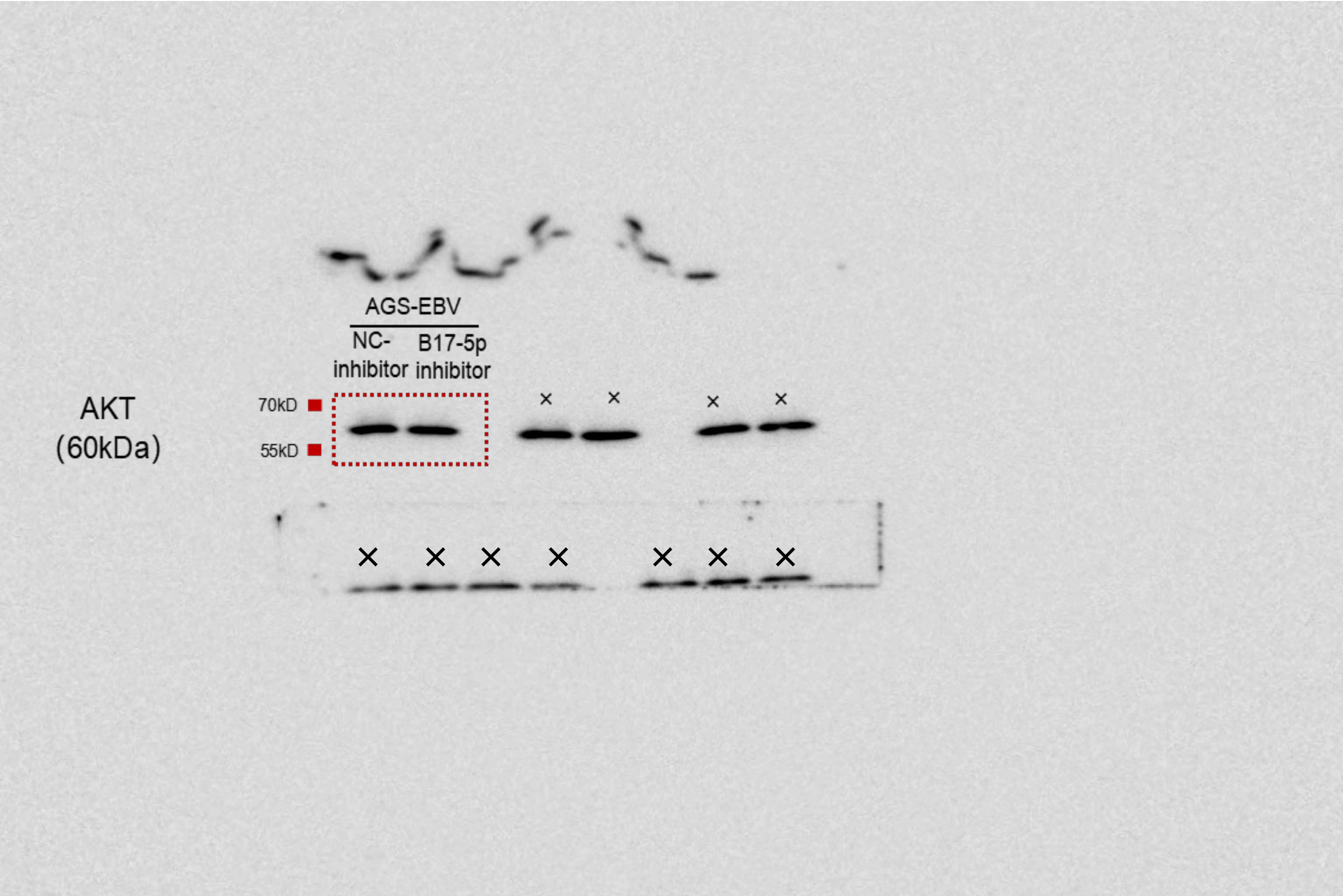

This imagecorresponds to Figure 6E

Biological Replicate #1

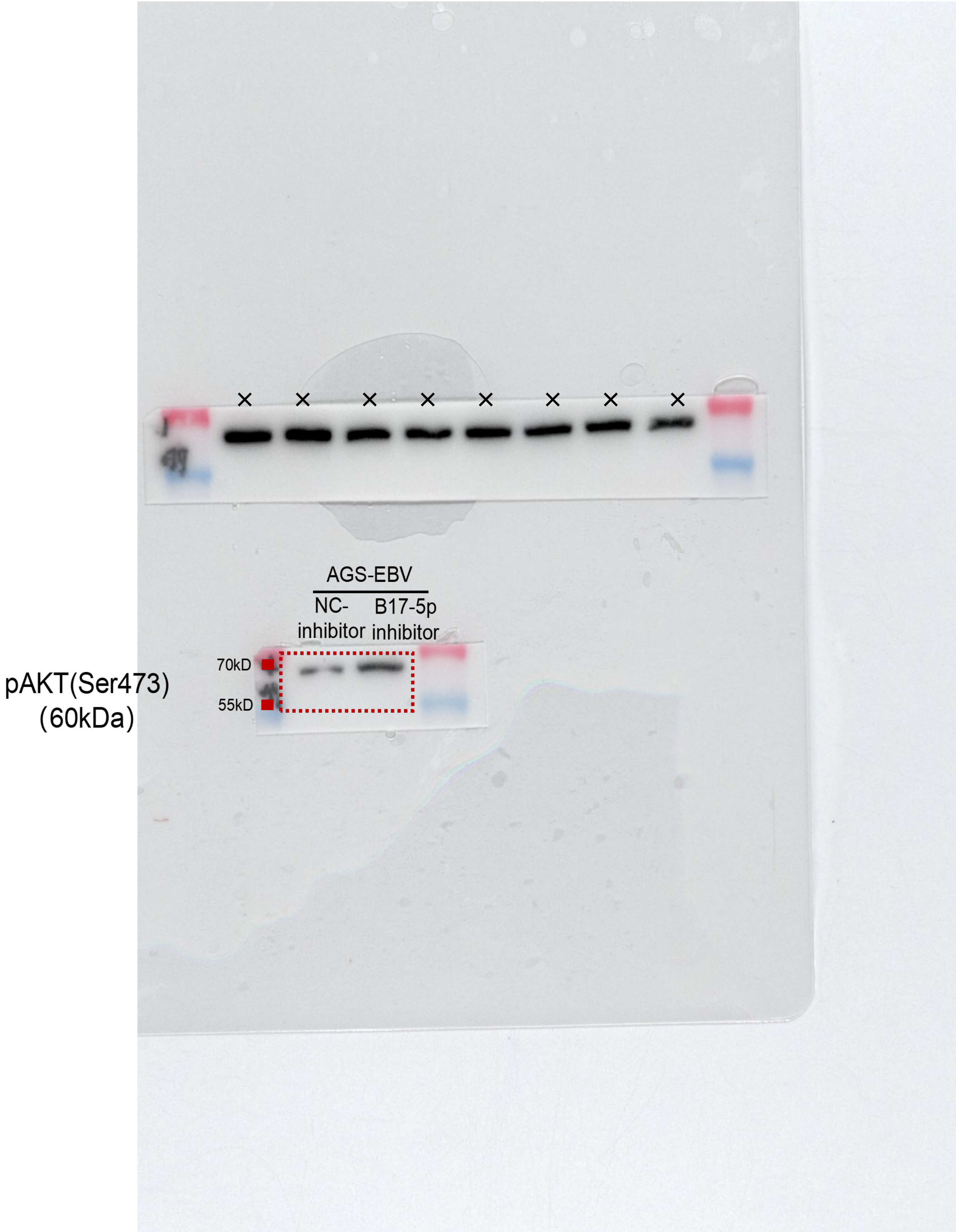

This imagecorresponds to Figure 6E

Biological Replicate #1

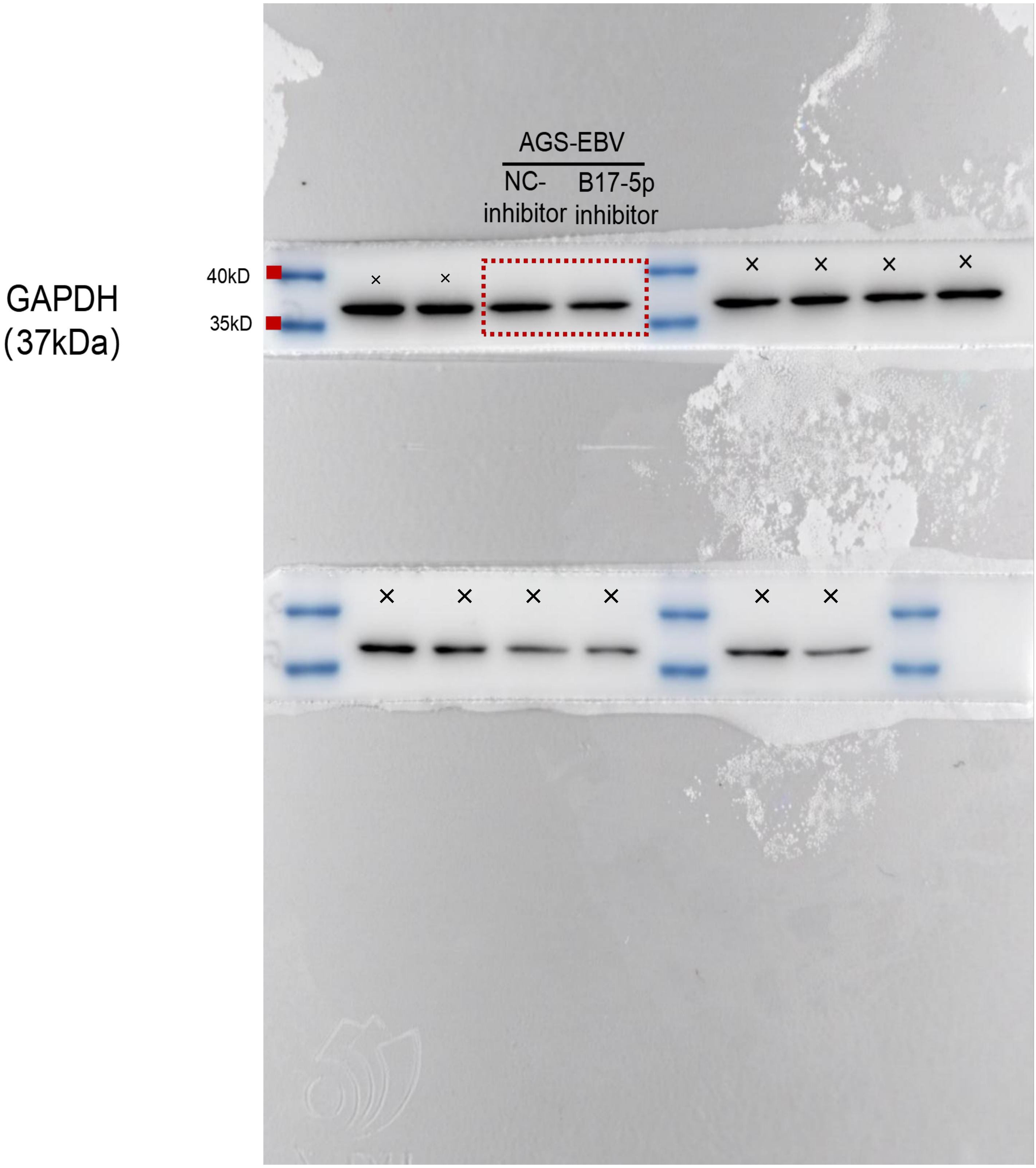

These images correspond to Figure 6E

Biological Replicate #2

▲ These images was selected as the representative result in Figure 6E.

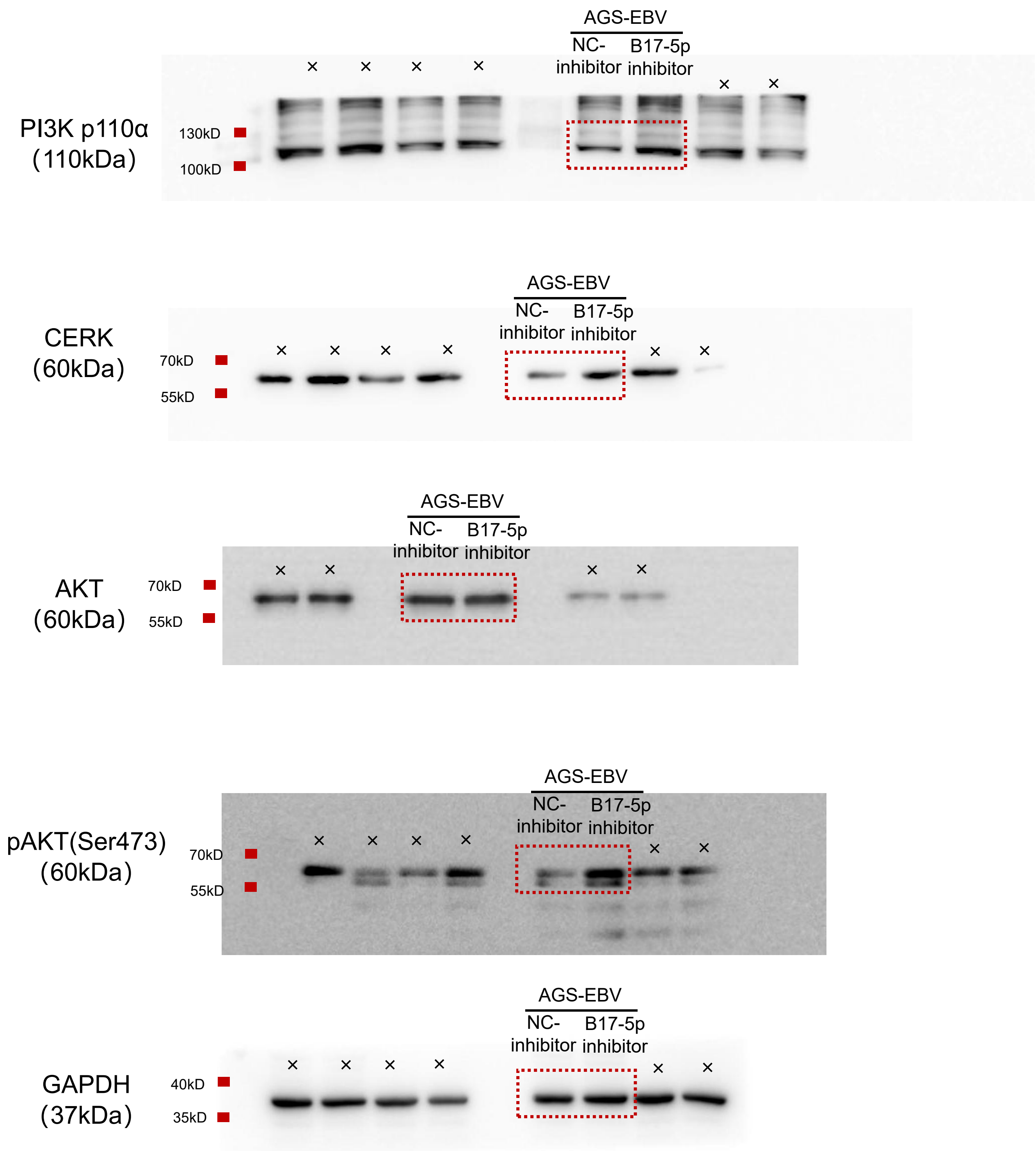

This image corresponds to Figure 6E

Biological Replicate #2

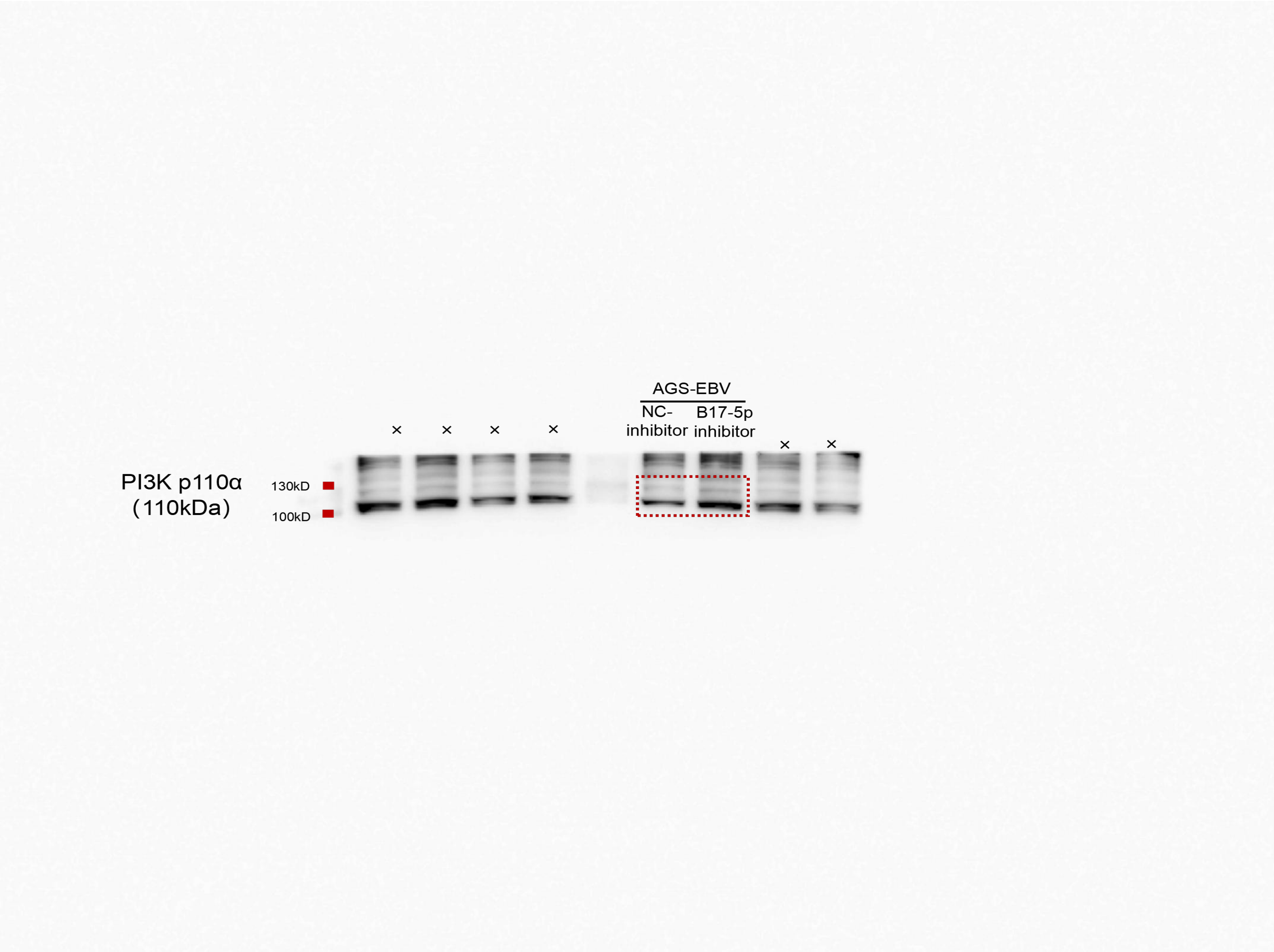

This image corresponds to Figure 6E

Biological Replicate #2

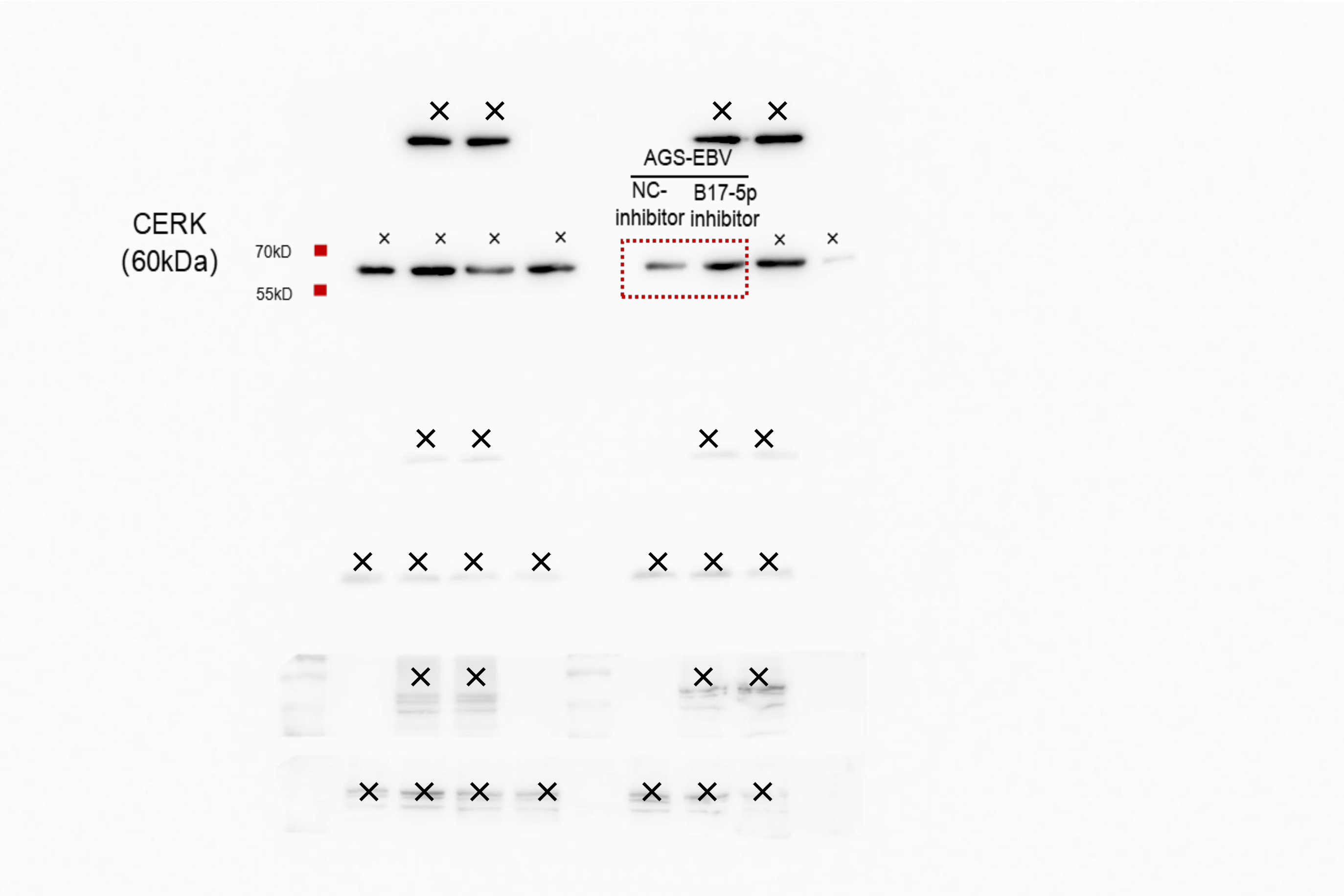

This image corresponds to Figure 6E

Biological Replicate #2

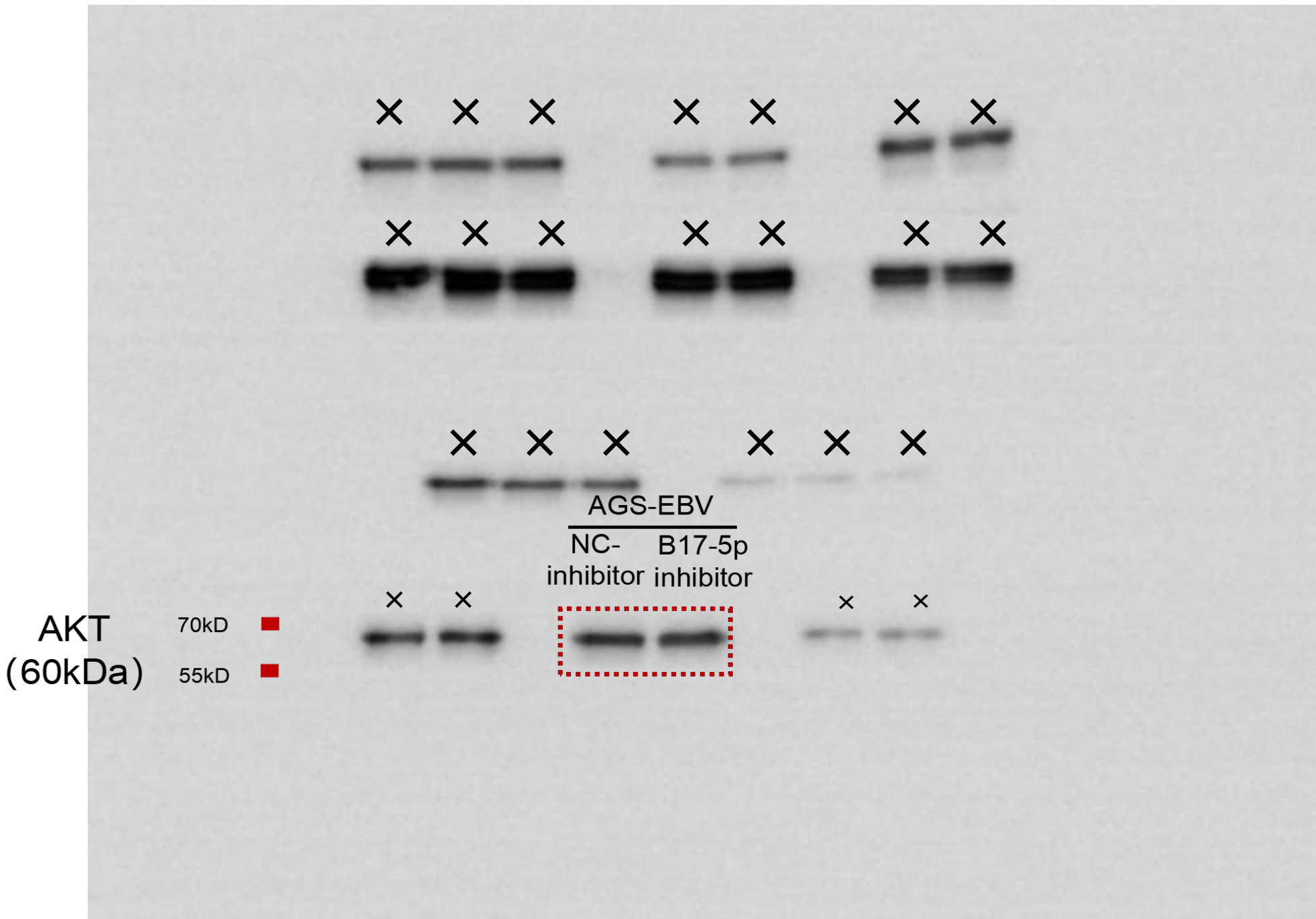

This image corresponds to Figure 6E

Biological Replicate #2

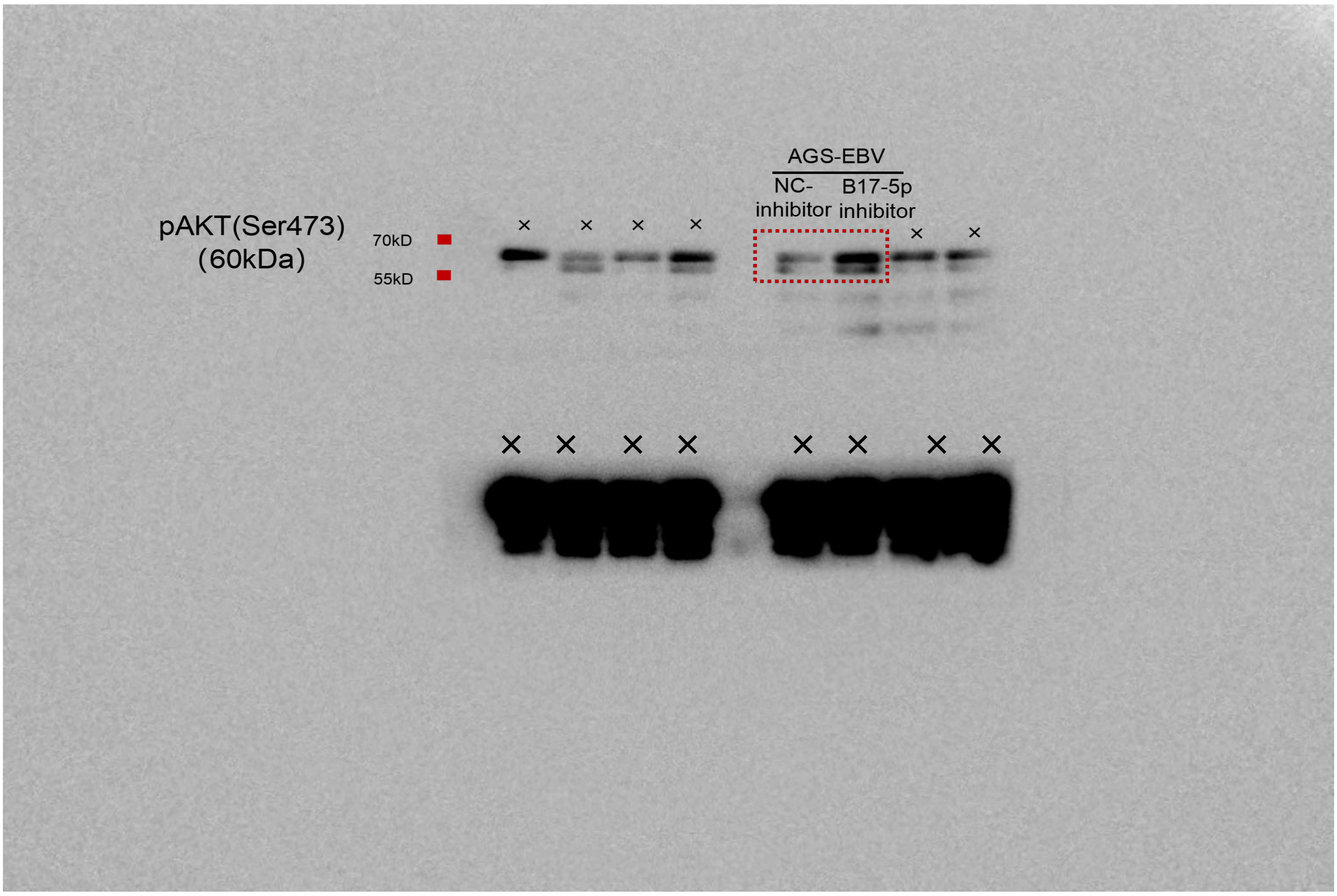

This image corresponds to Figure 6E

Biological Replicate #2

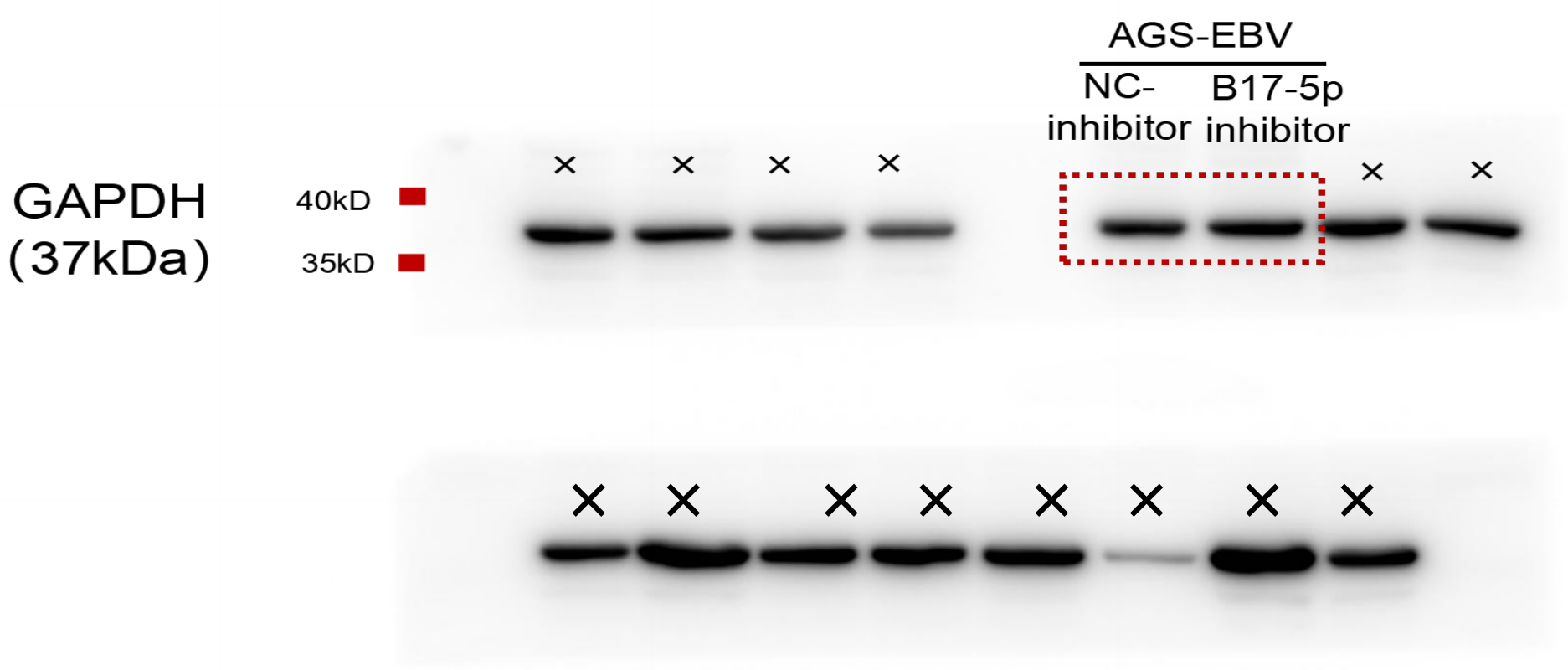

These images correspond to Figure 6E

Biological Replicate #3

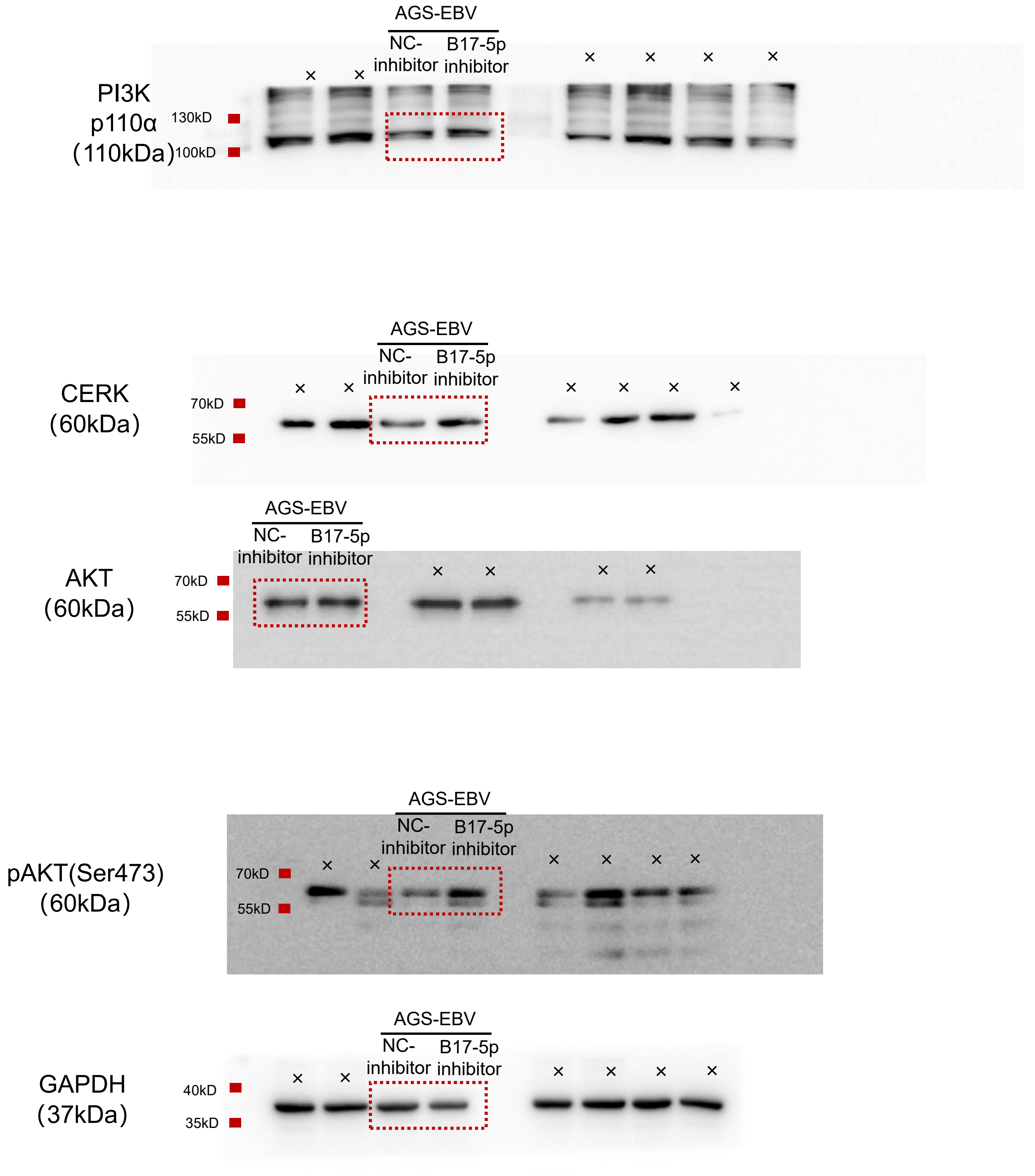

This image corresponds to Figure 6E

Biological Replicate #3

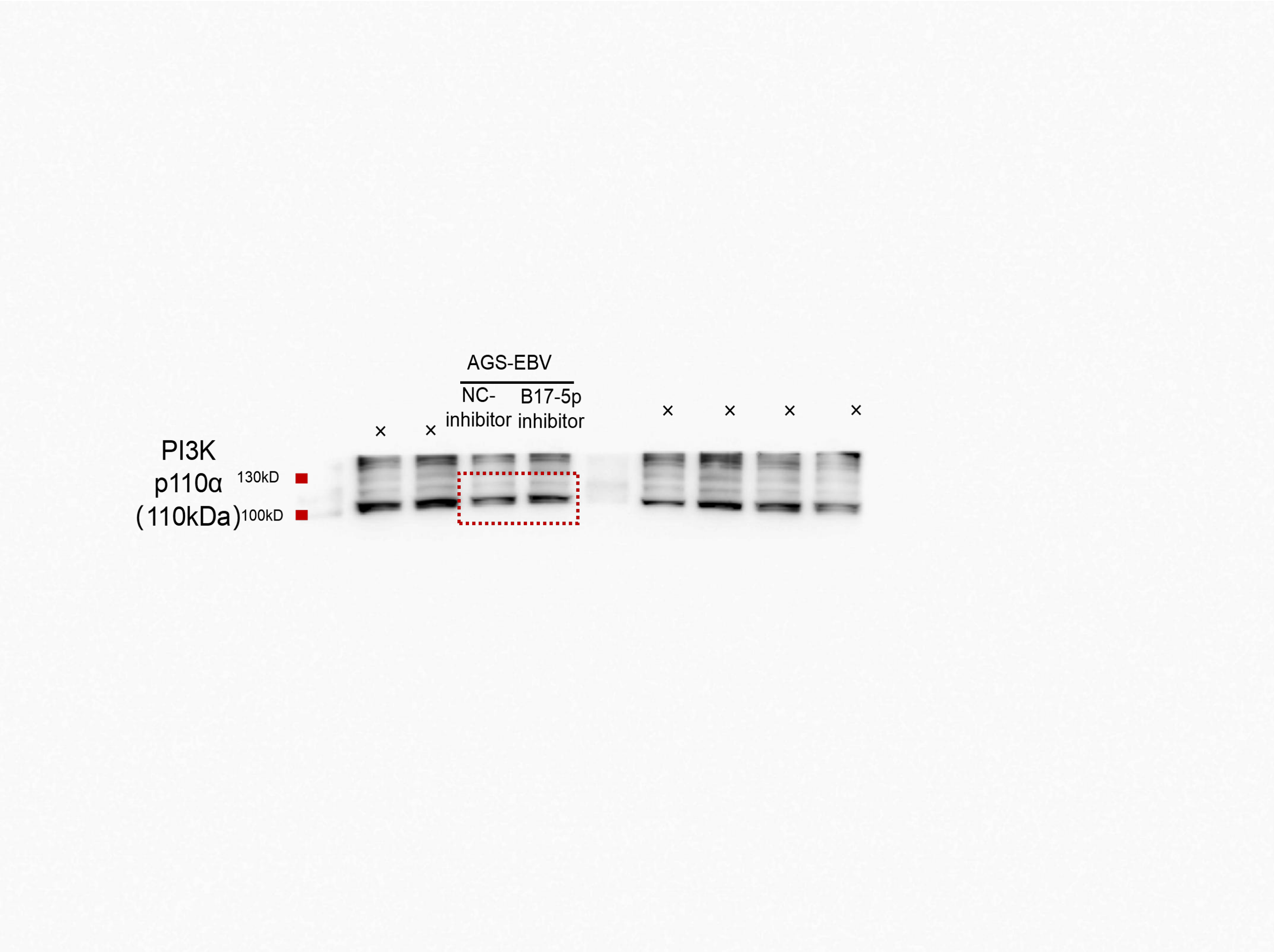

This image corresponds to Figure 6E

Biological Replicate #3

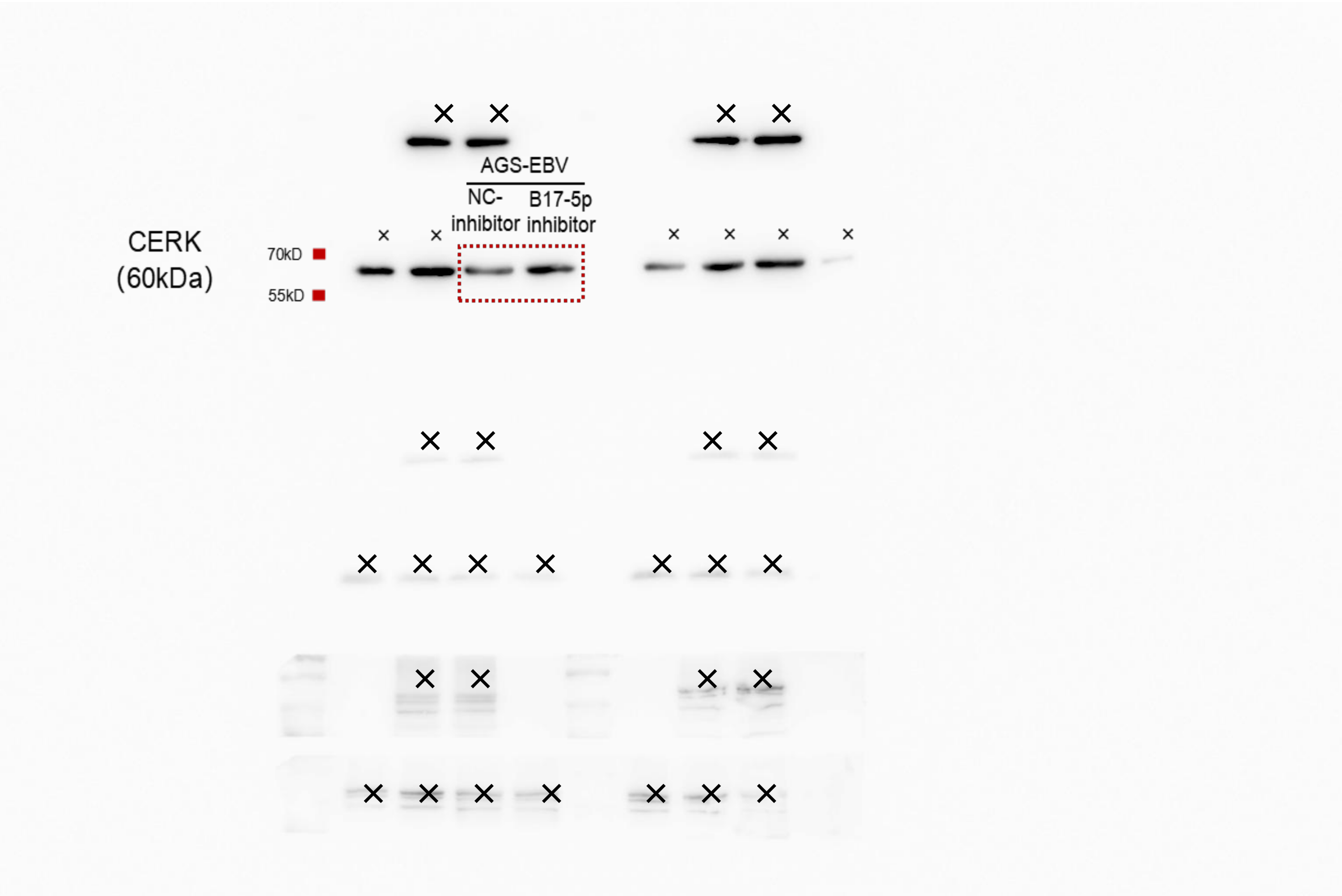

This image corresponds to Figure 6E

Biological Replicate #3

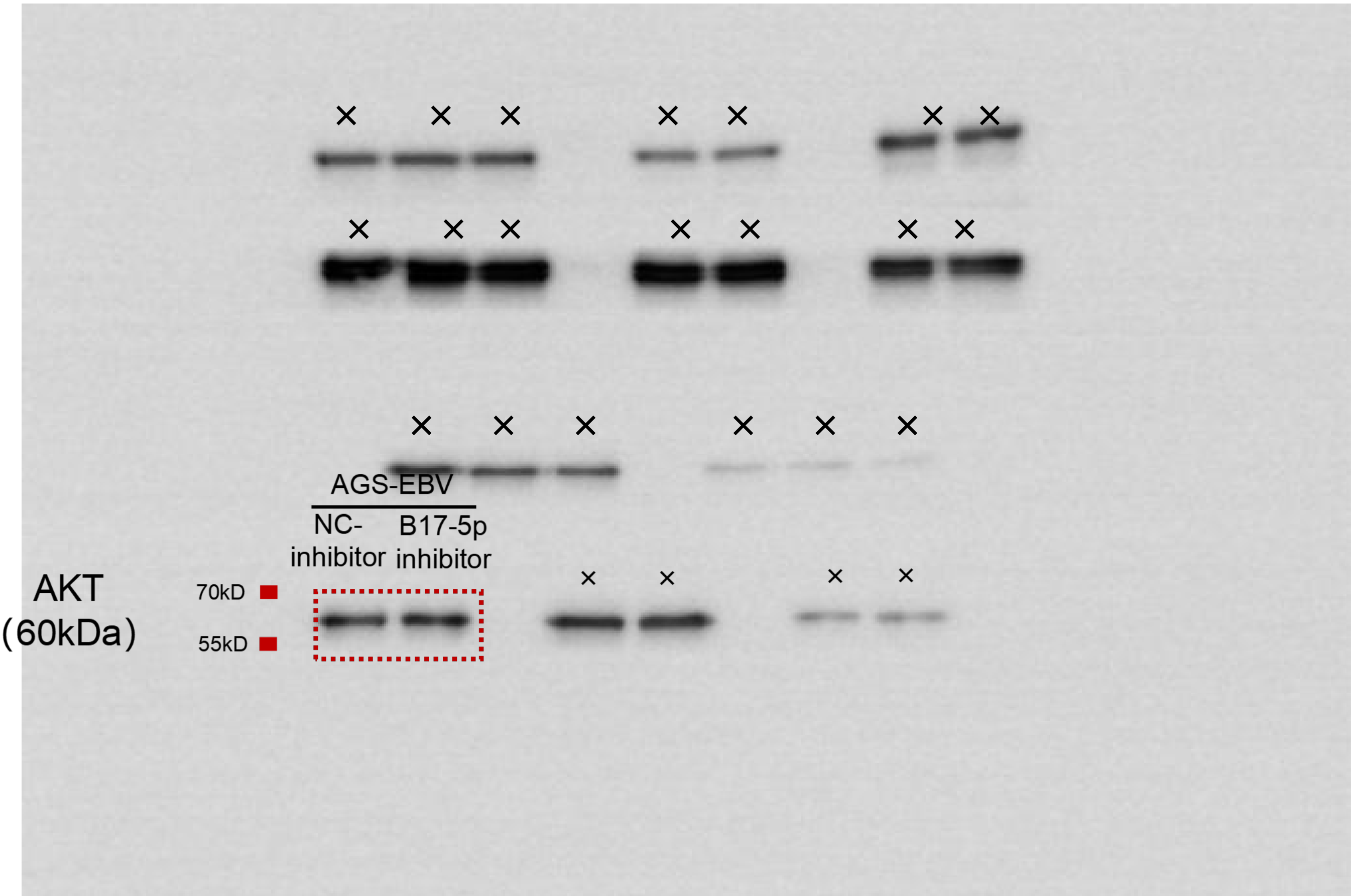

This image corresponds to Figure 6E

Biological Replicate #3

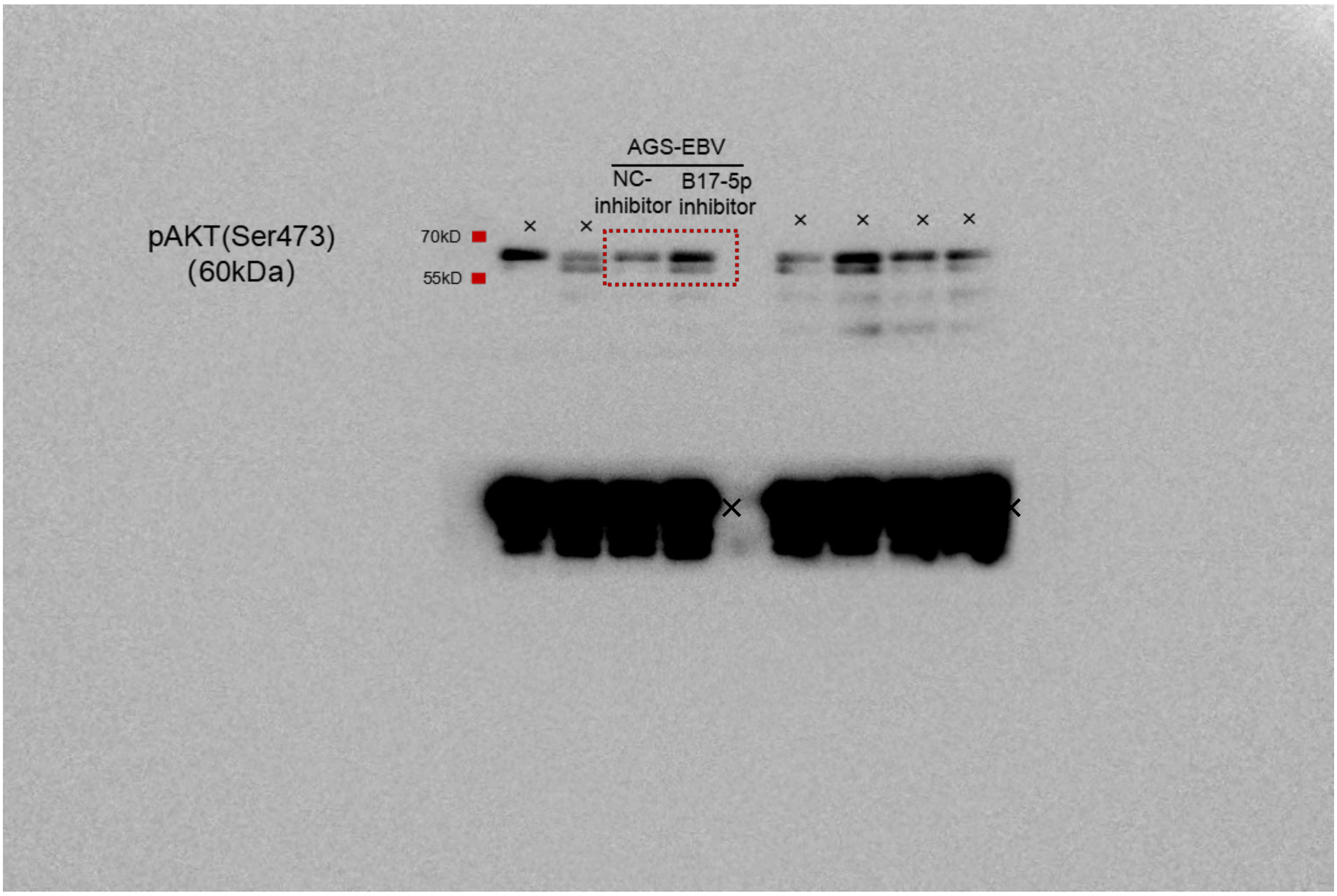

This image corresponds to Figure 6E

Biological Replicate #3

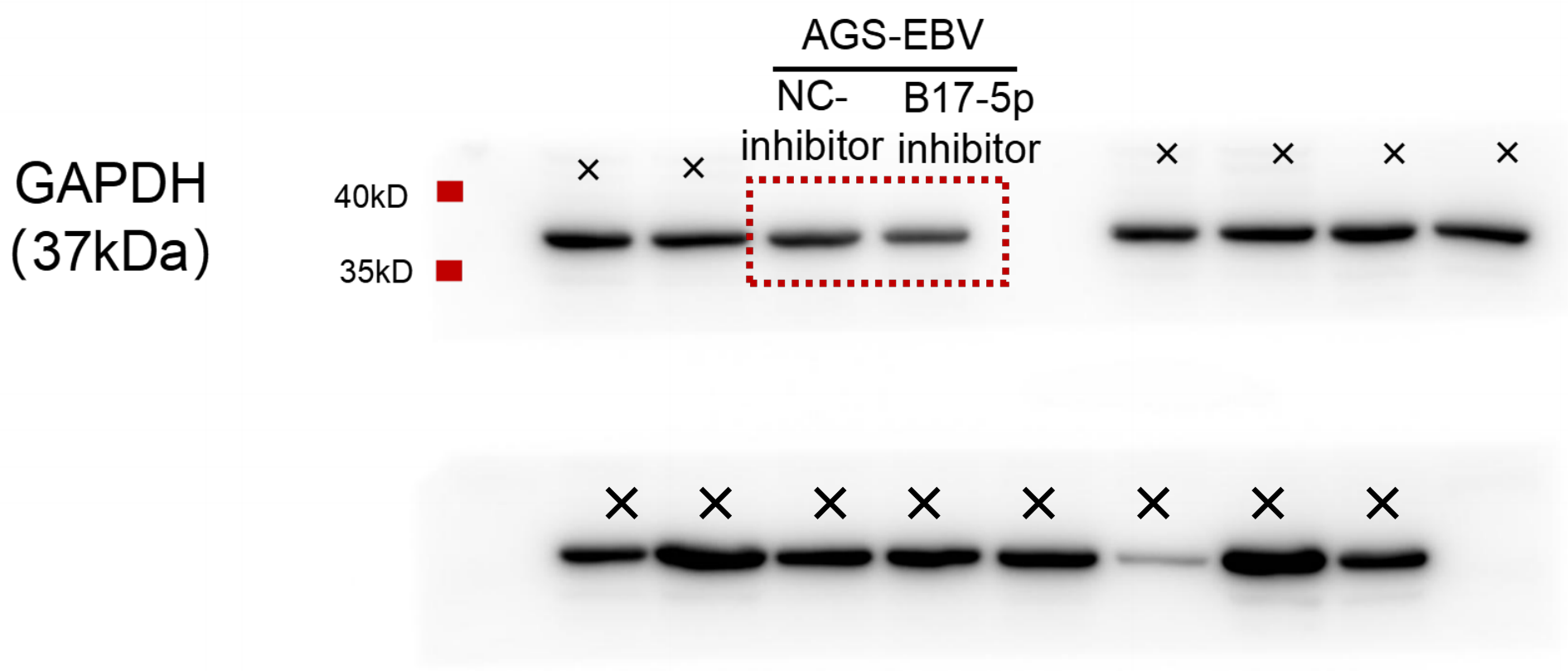

These images correspond to Figure 6G

Biological Replicate #1

▲ These images was selected as the representative result in Figure 6G.

CERK
(60kDa)

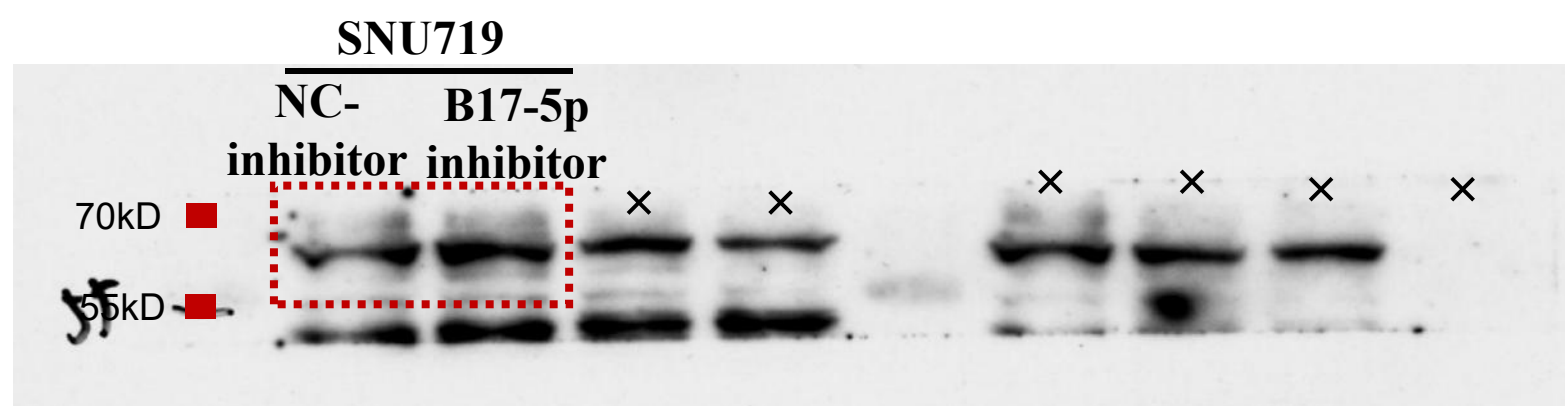

PI3K p110α
(110kDa)

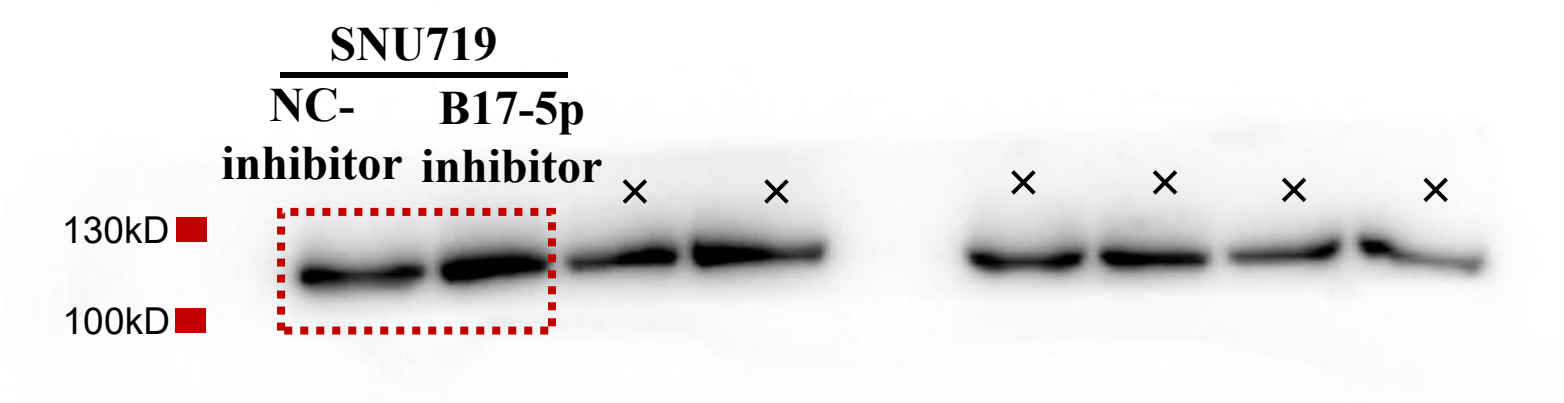

AKT
(60kDa)

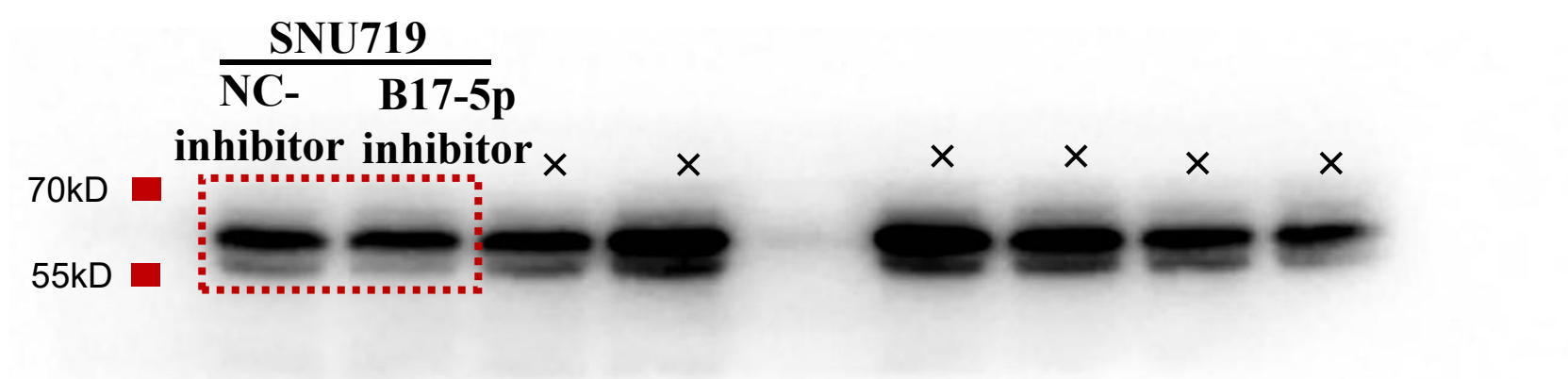

pAKT(Ser473)
(60kDa)

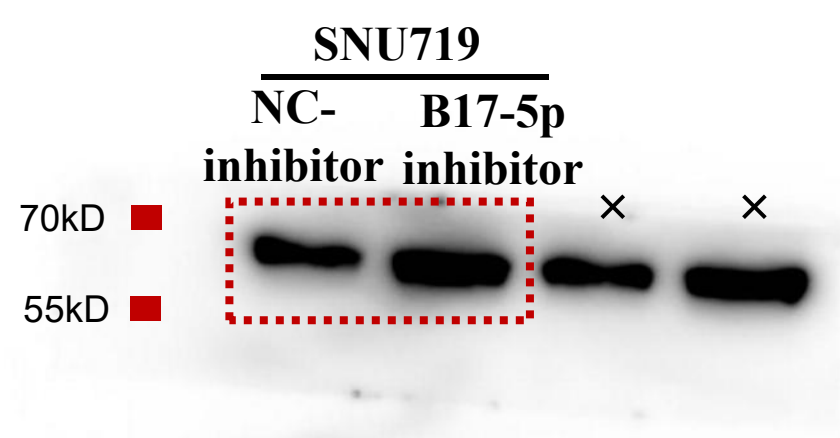

GAPDH
(37kDa)

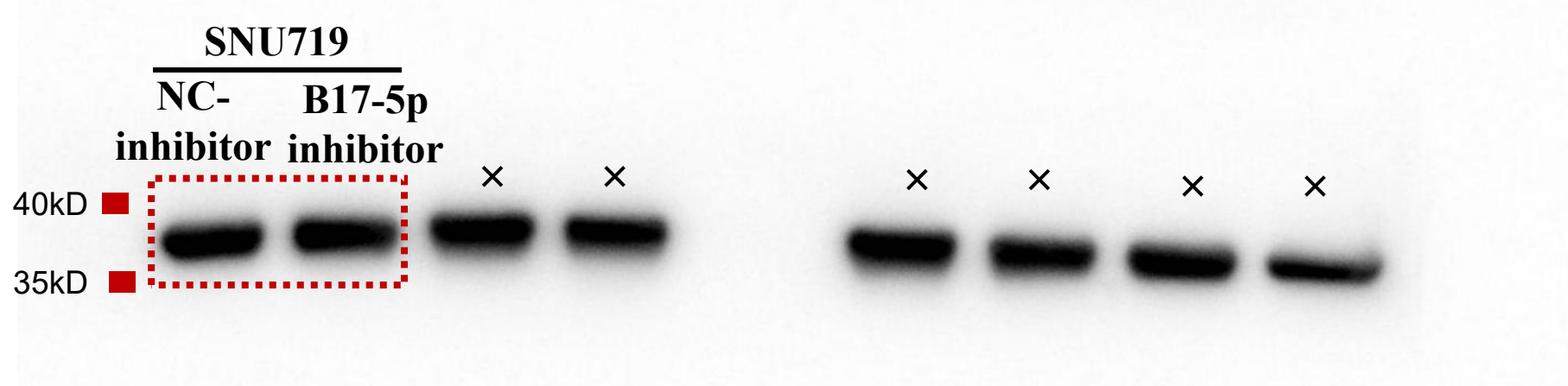

This image corresponds to Figure 6G

Biological Replicate #1

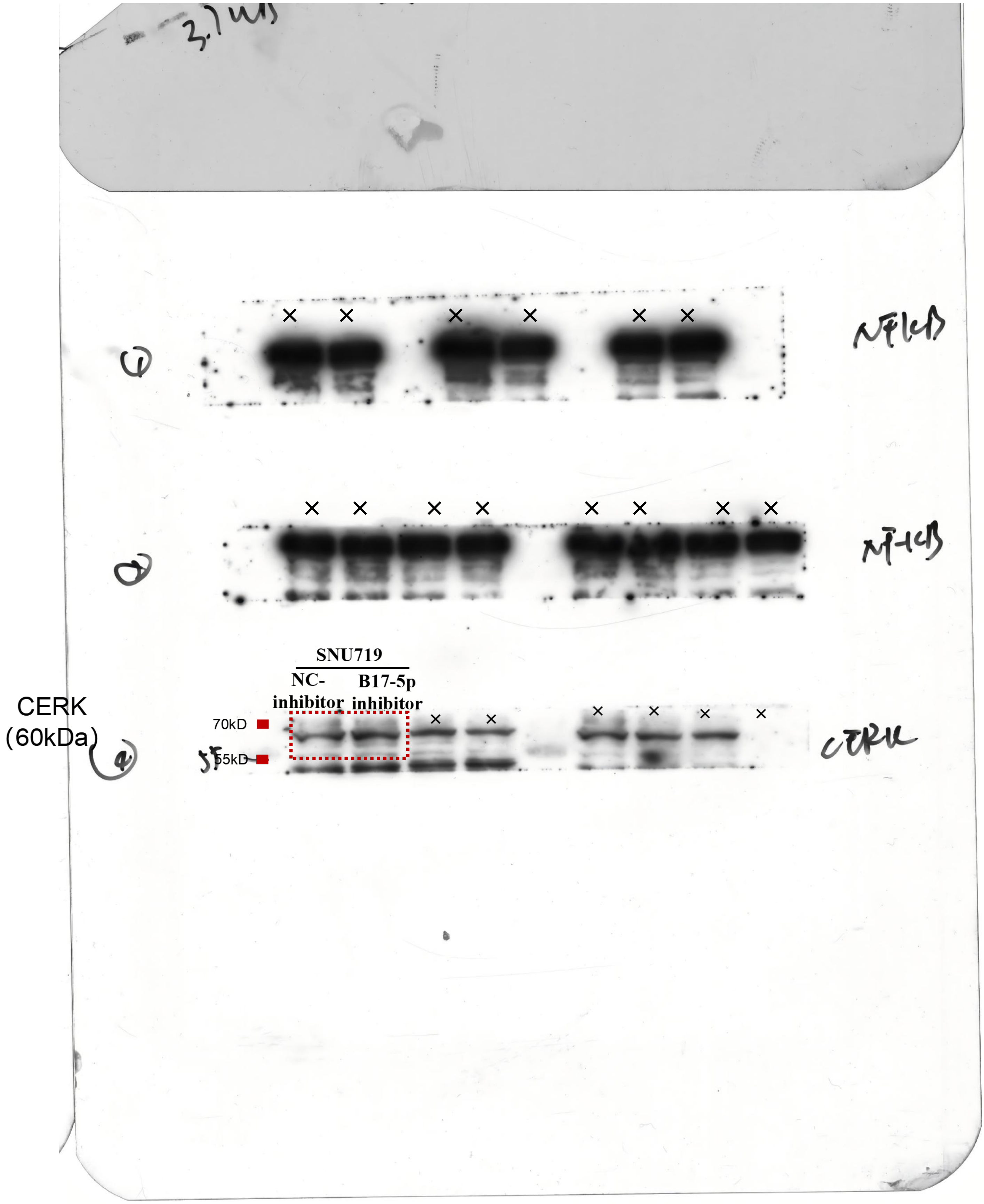

This image corresponds to Figure 6G

Biological Replicate #1

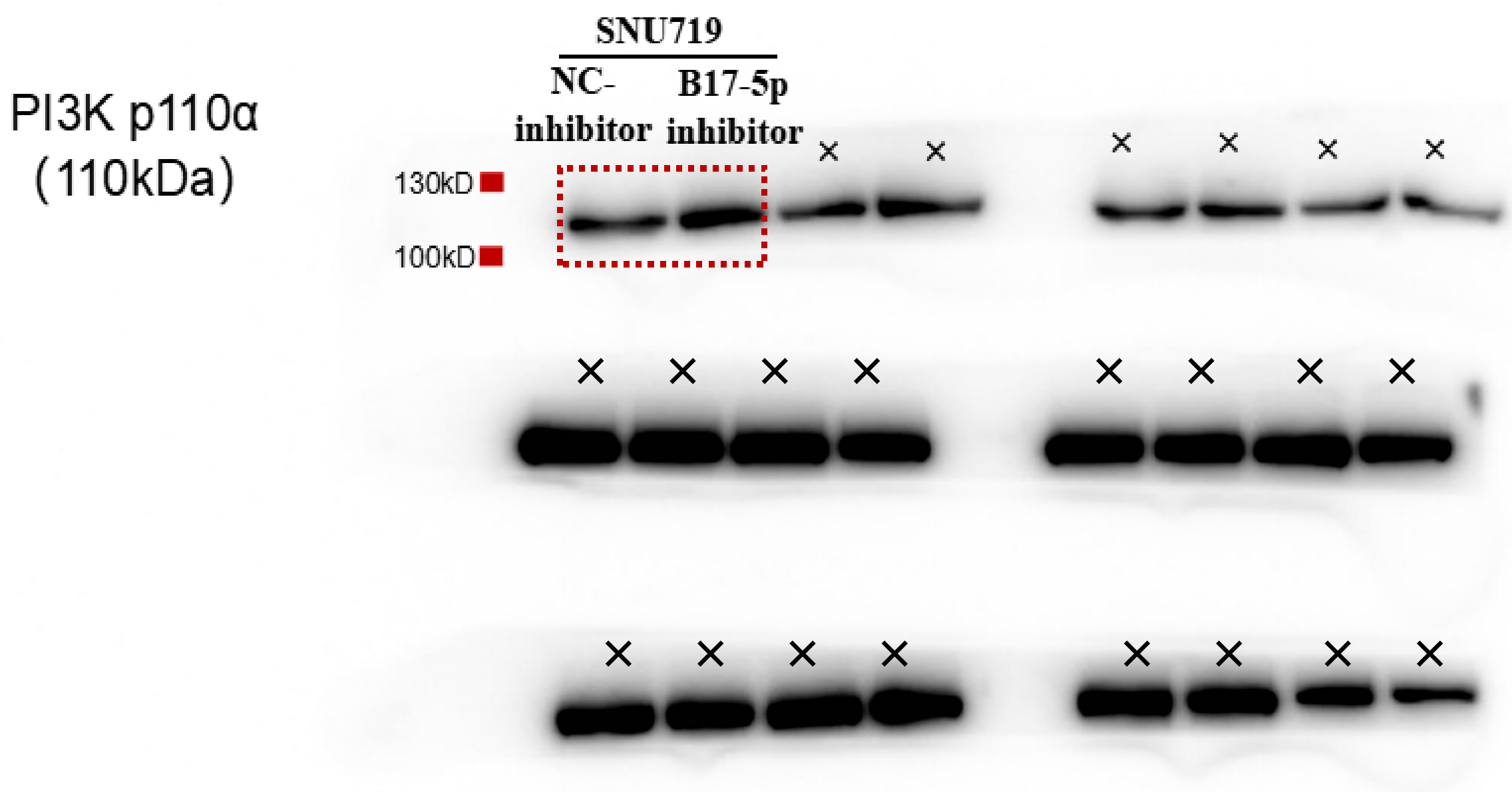

This image corresponds to Figure 6G

Biological Replicate #1

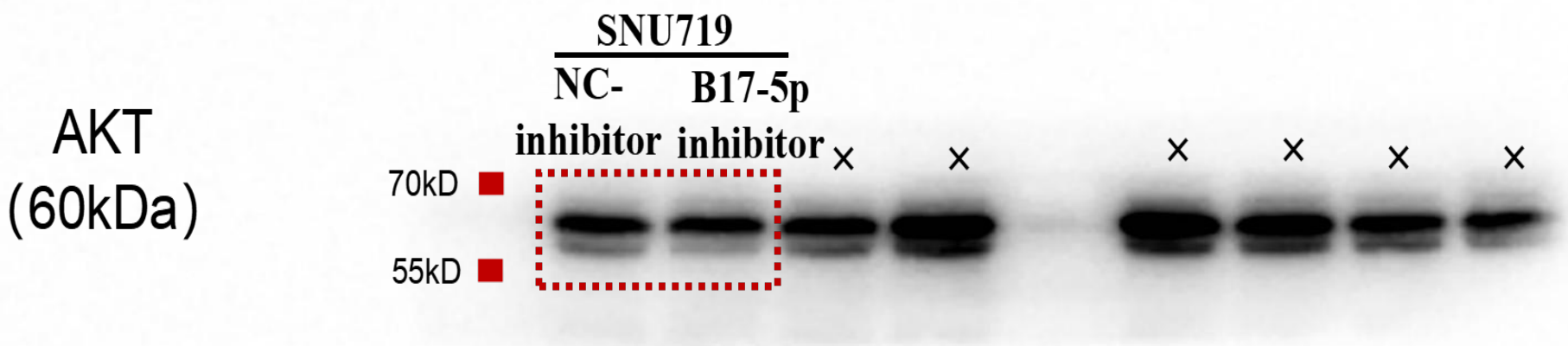

This image corresponds to Figure 6G

Biological Replicate #1

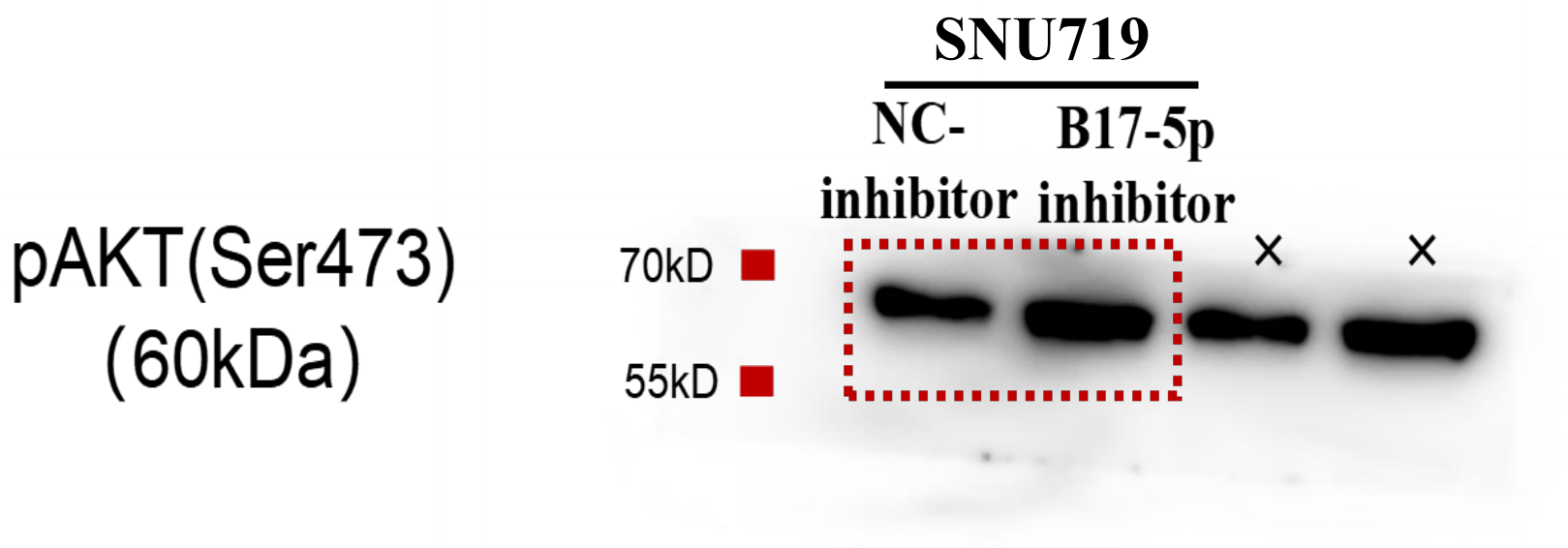

This image corresponds to Figure 6G

Biological Replicate #1

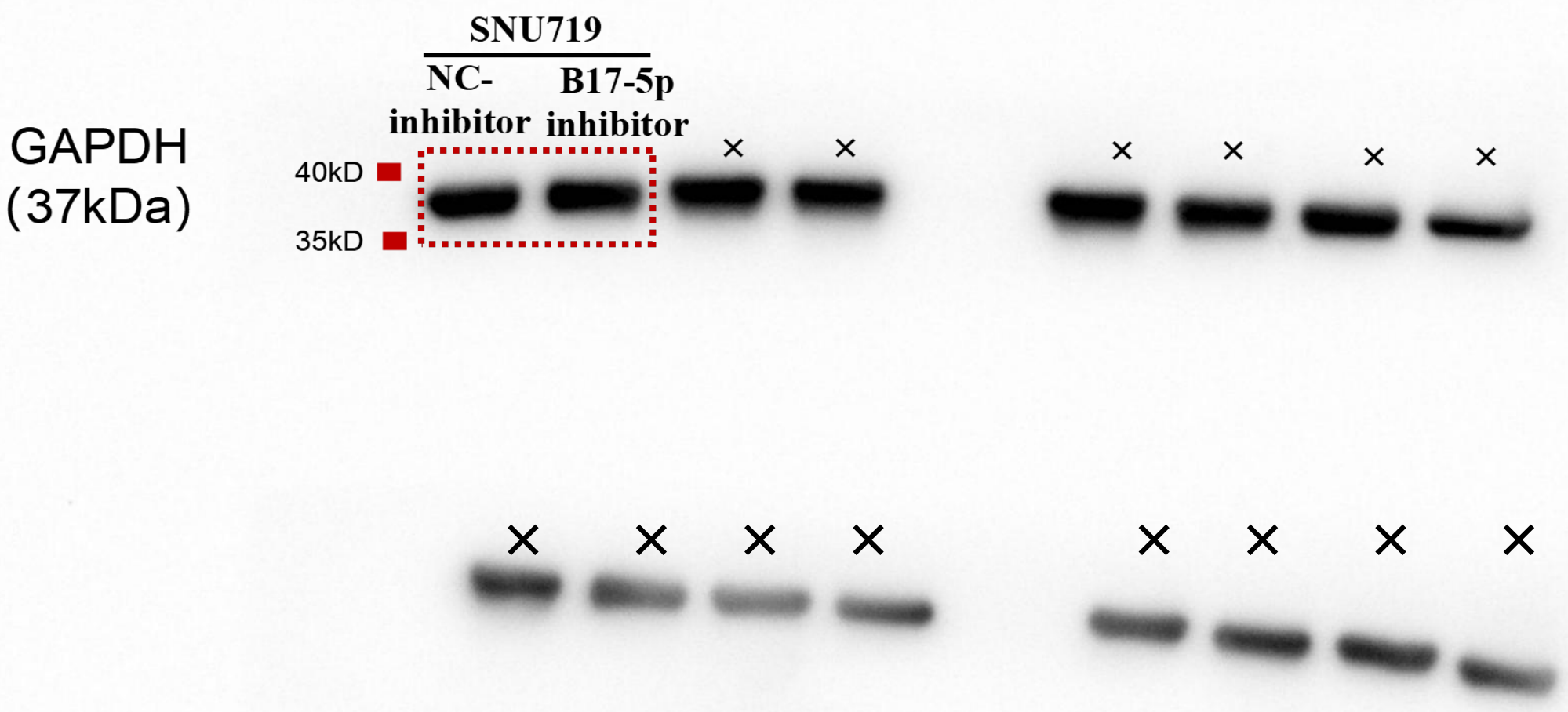

These images correspond to Figure 6G

Biological Replicate #2

CERK
(60kDa)

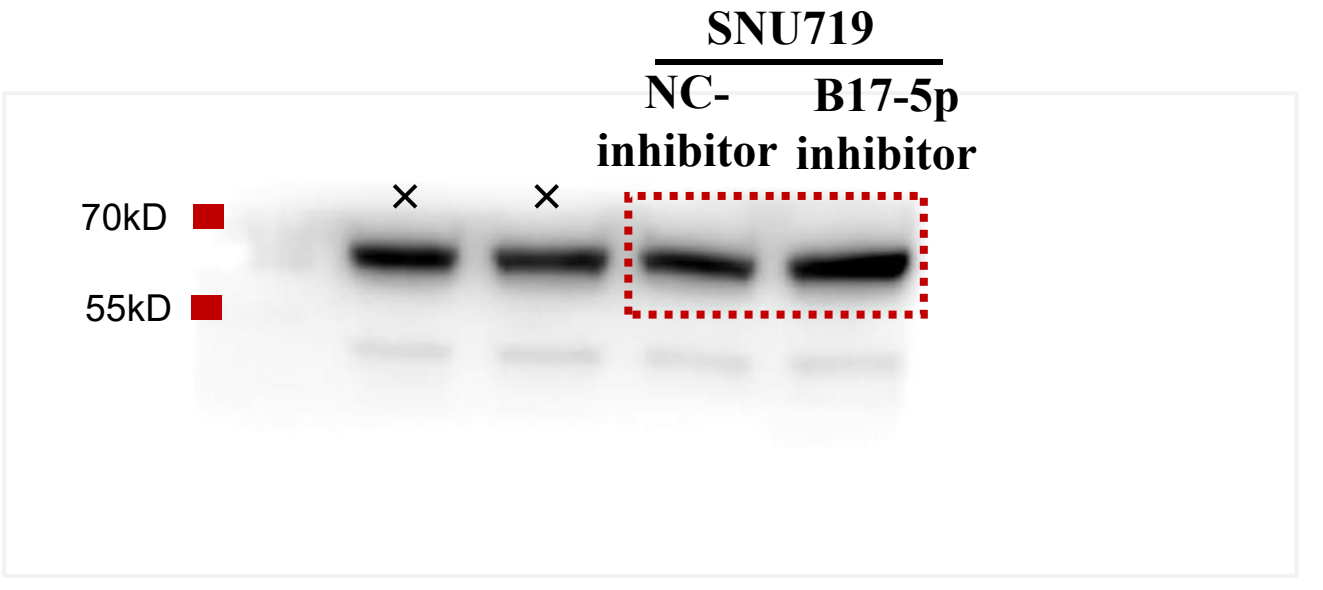

PI3K p110α
(110kDa)

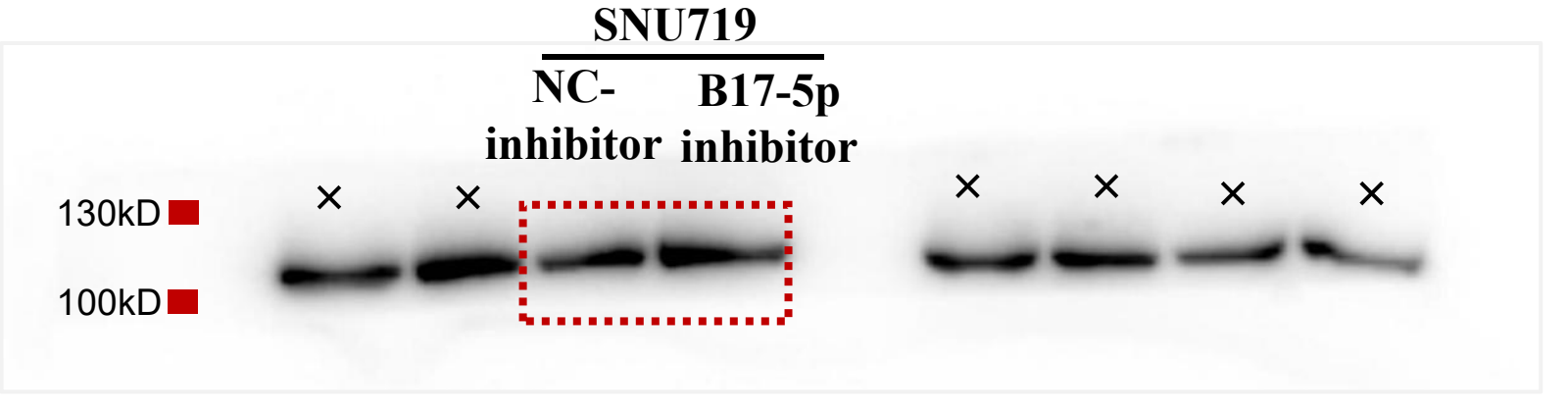

AKT
(60kDa)

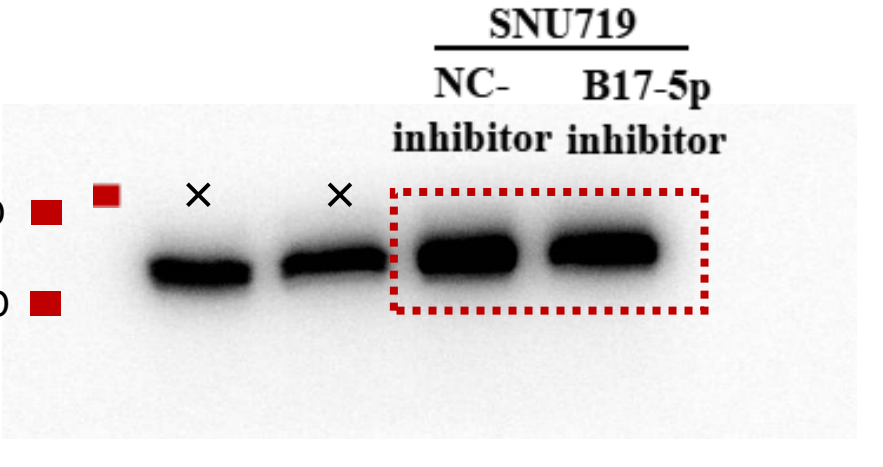

pAKT(Ser473)
(60kDa)

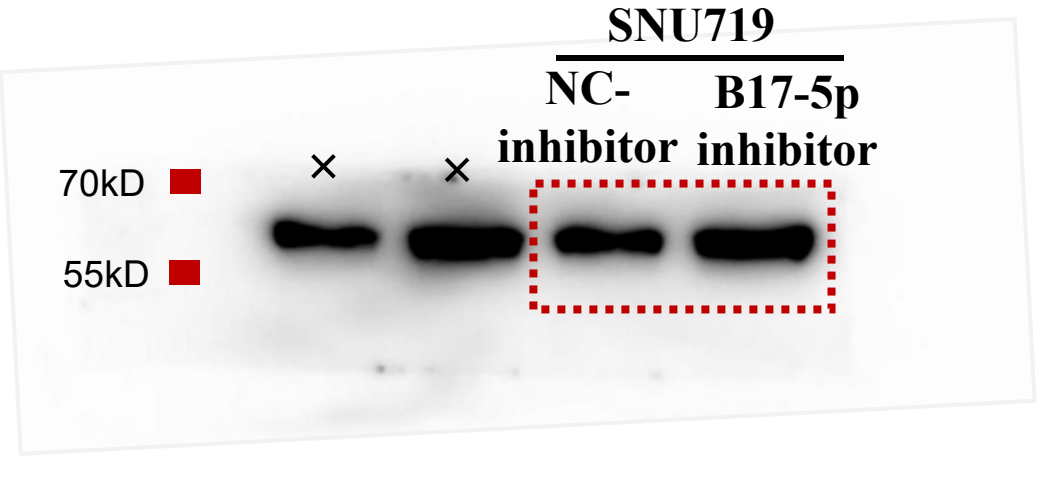

GAPDH
(37kDa)

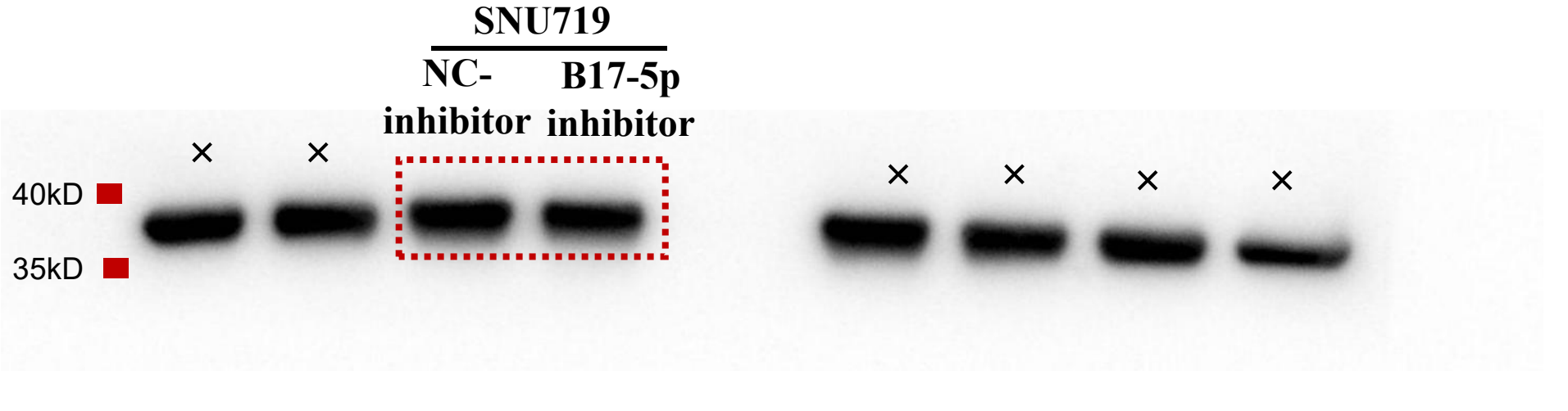

This image corresponds to Figure 6G

Biological Replicate #2

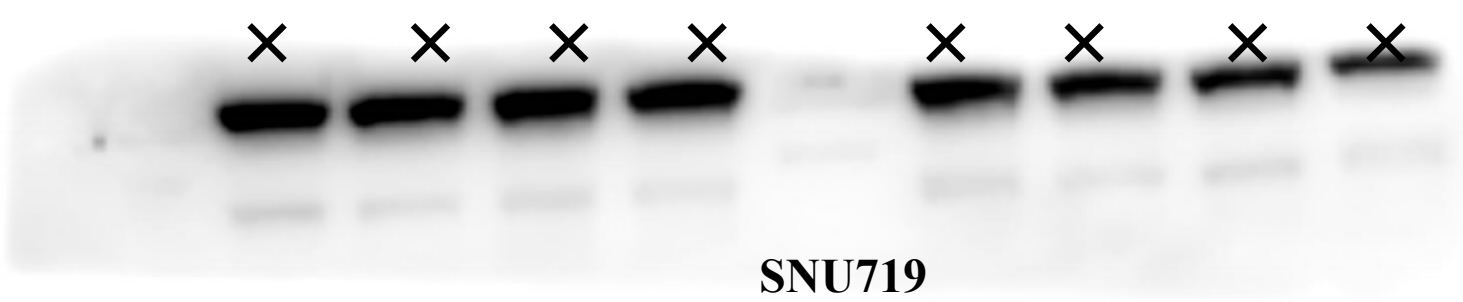

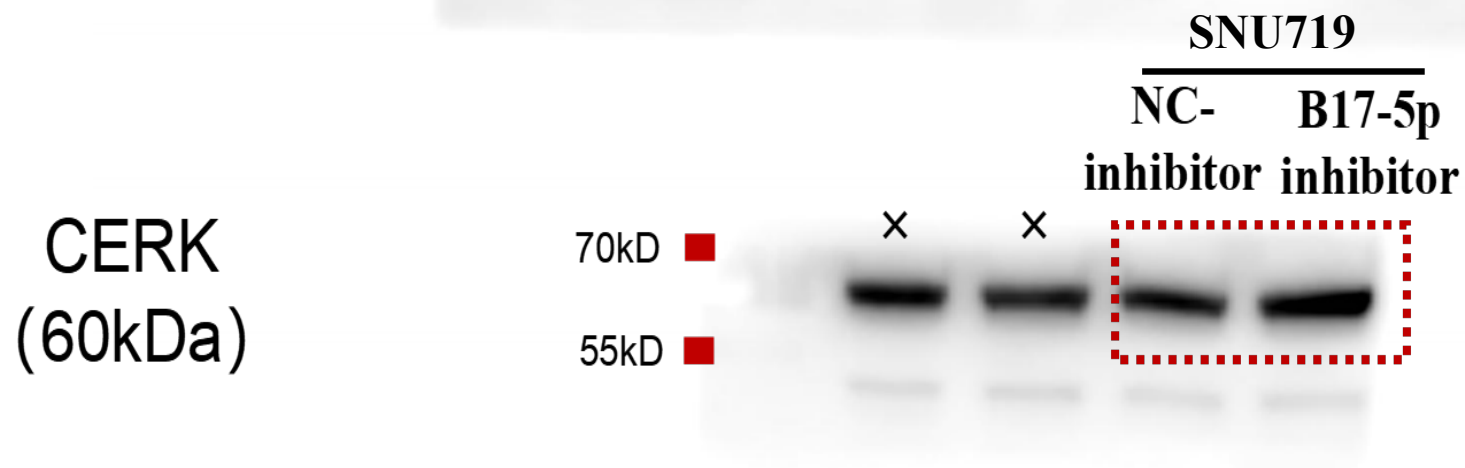

This image corresponds to Figure 6G

Biological Replicate #2

PI3K p110α
(110kDa)

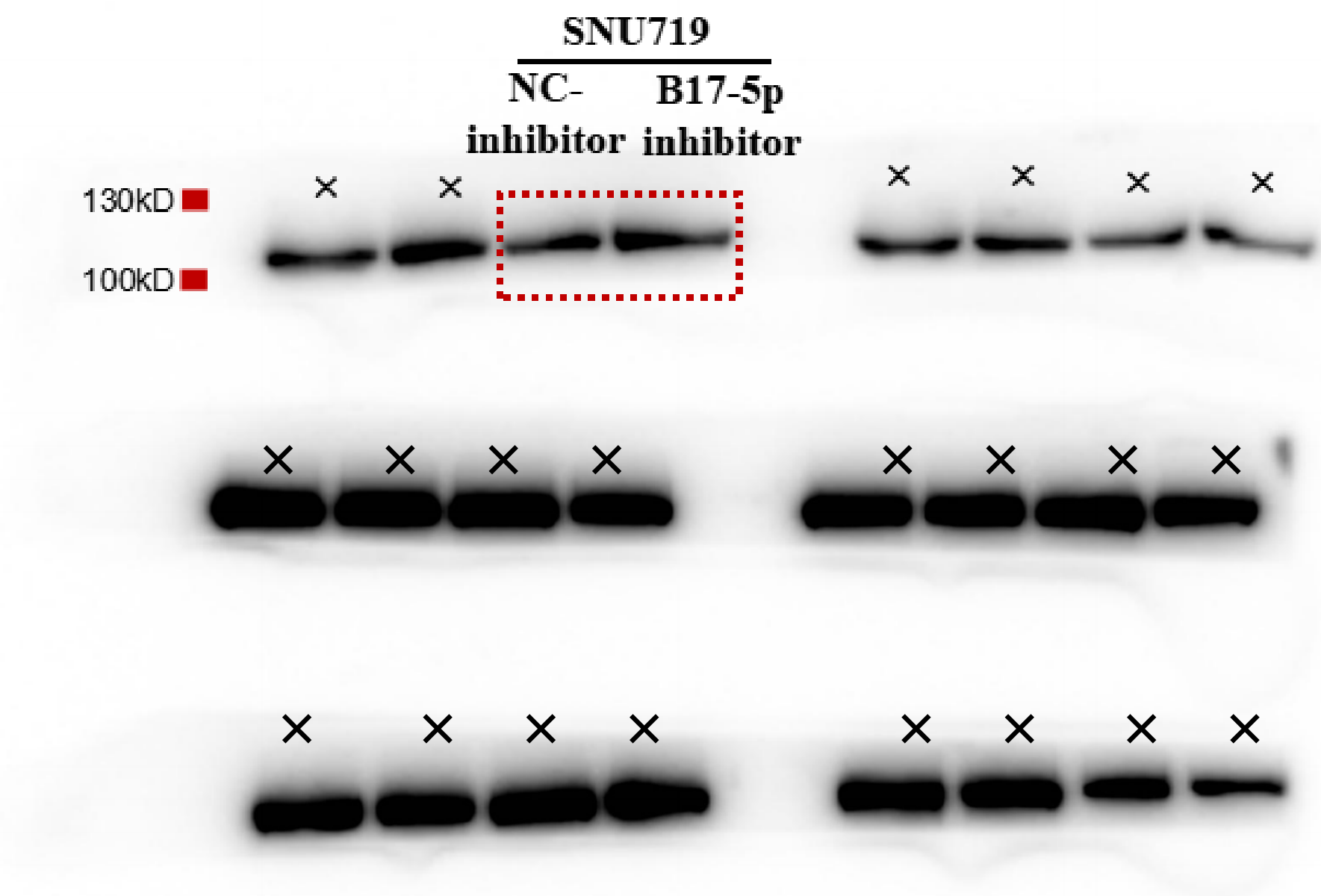

This image corresponds to Figure 6G

Biological Replicate #2

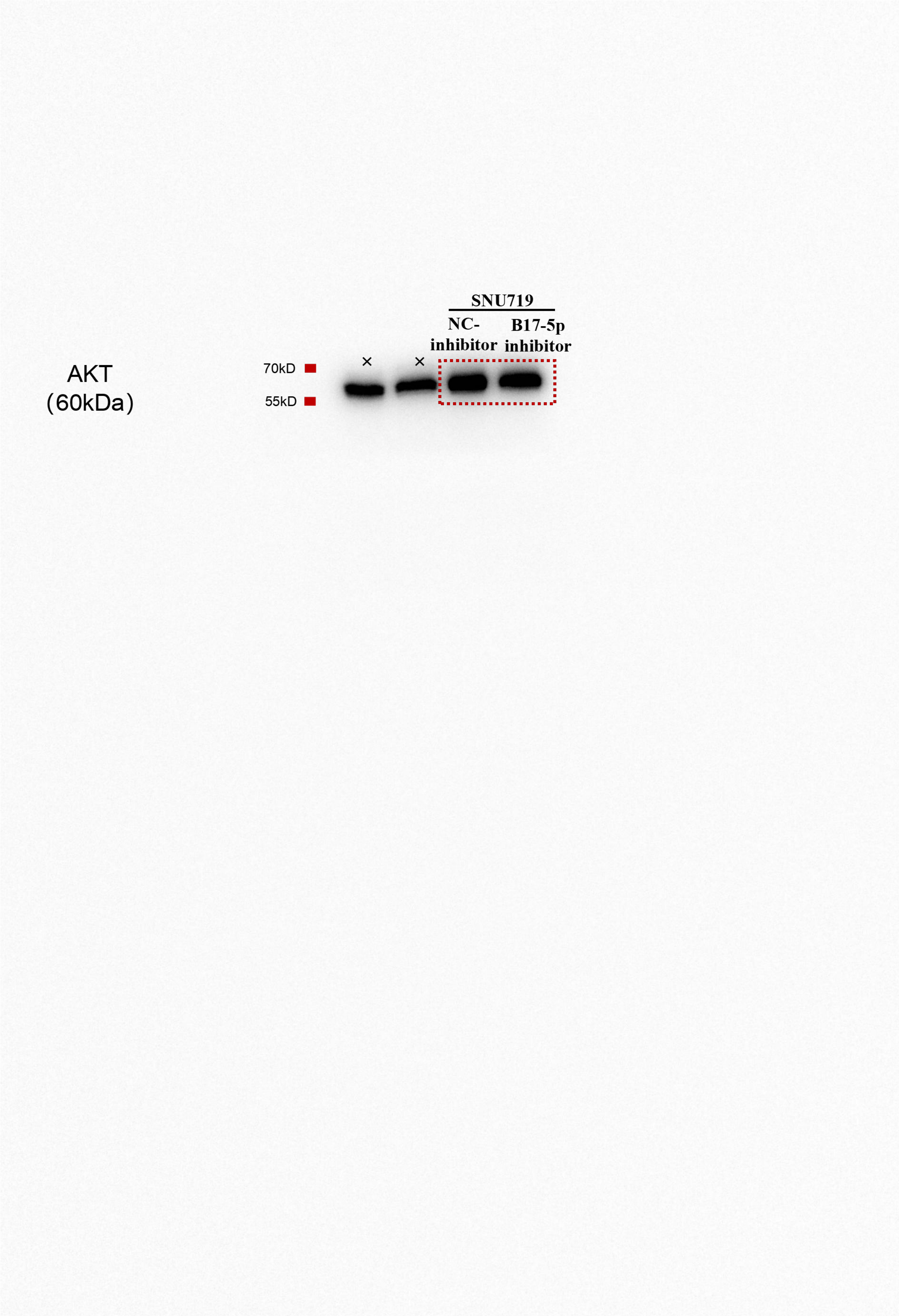

This image corresponds to Figure 6G

Biological Replicate #2

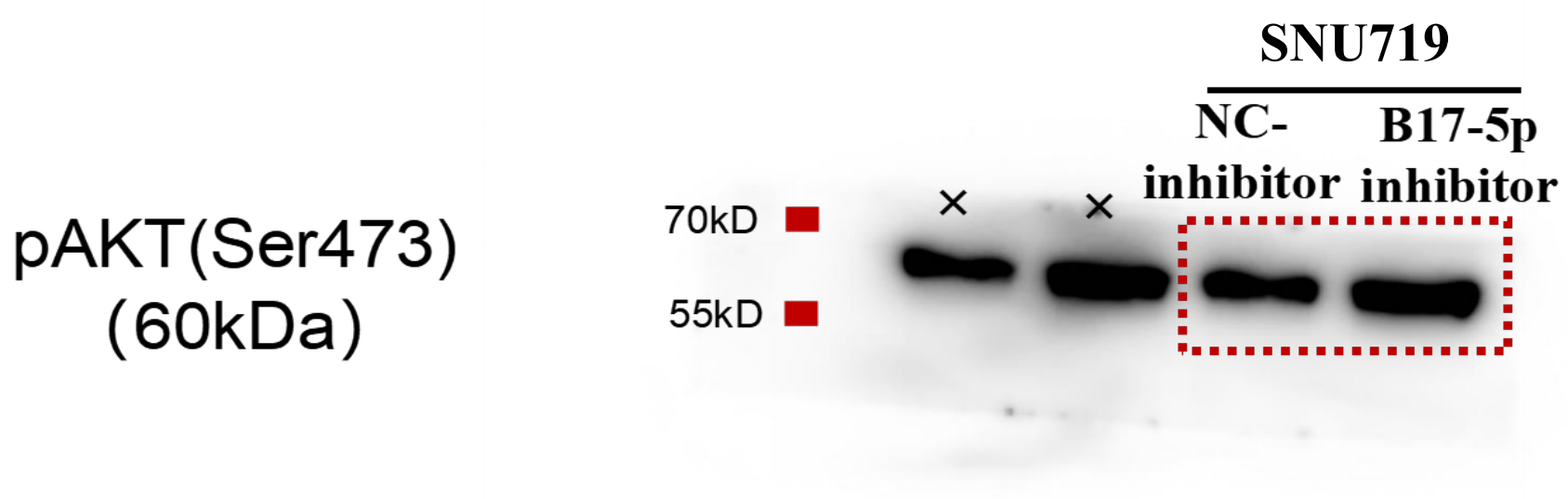

This image corresponds to Figure 6G

Biological Replicate #2

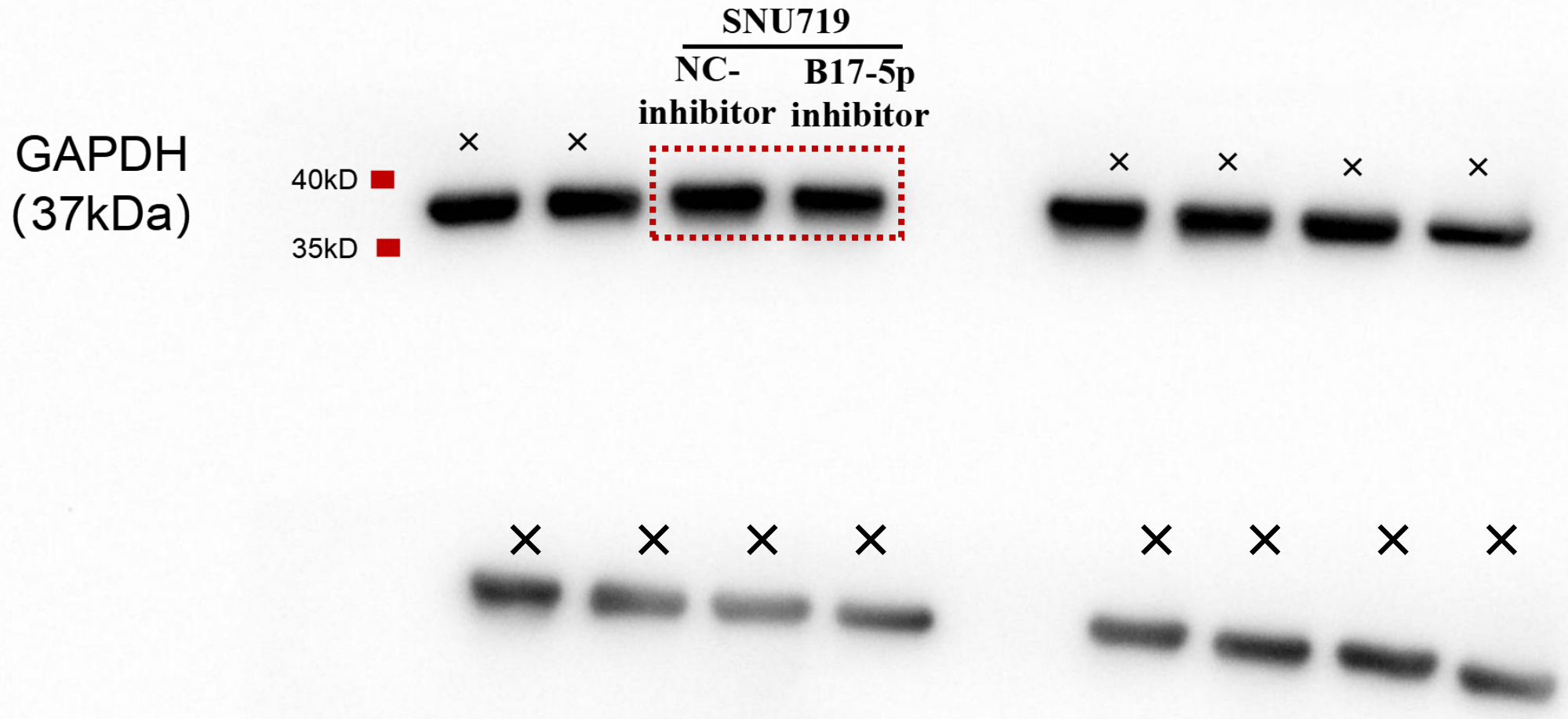

These images correspond to Figure 6G

Biological Replicate #3

CERK
(60kDa)

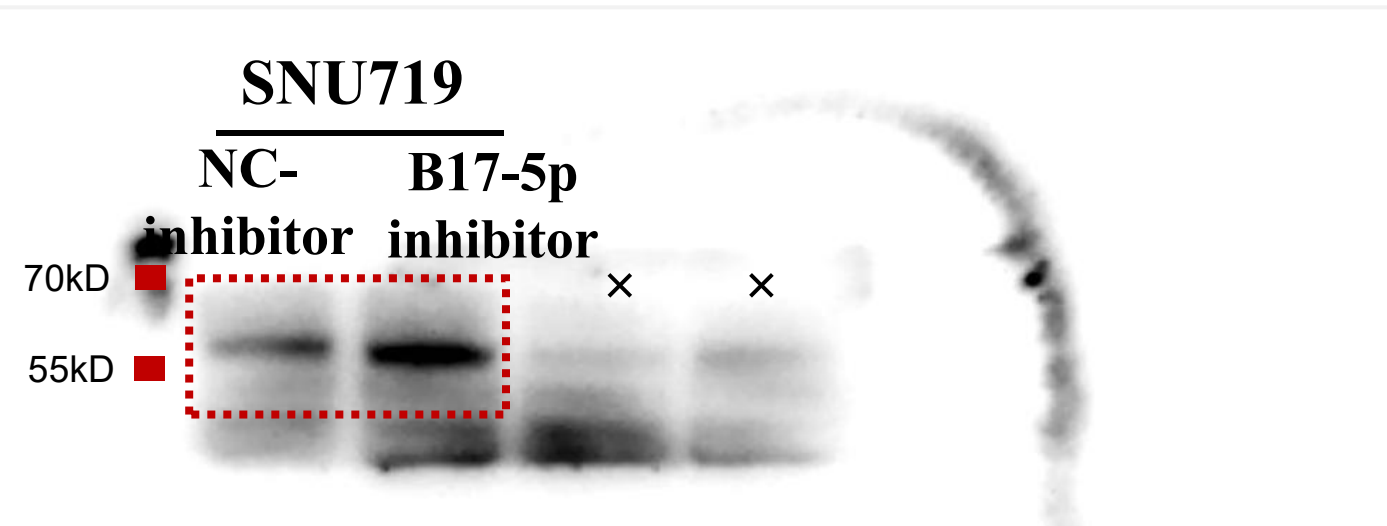

PI3K p110α
(110kDa)

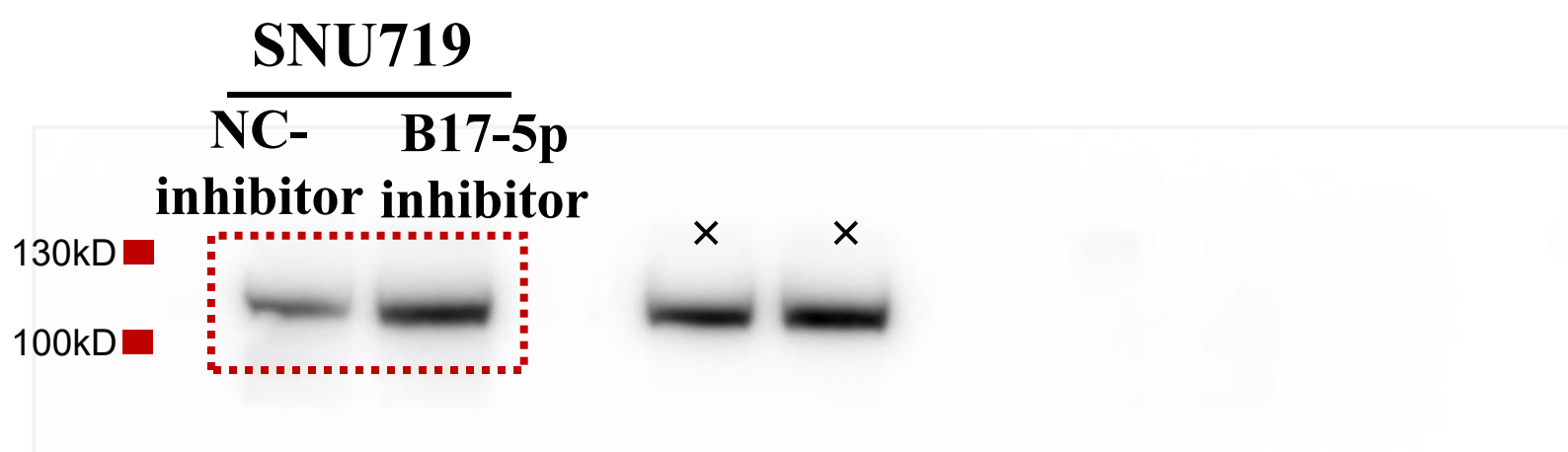

AKT
(60kDa)

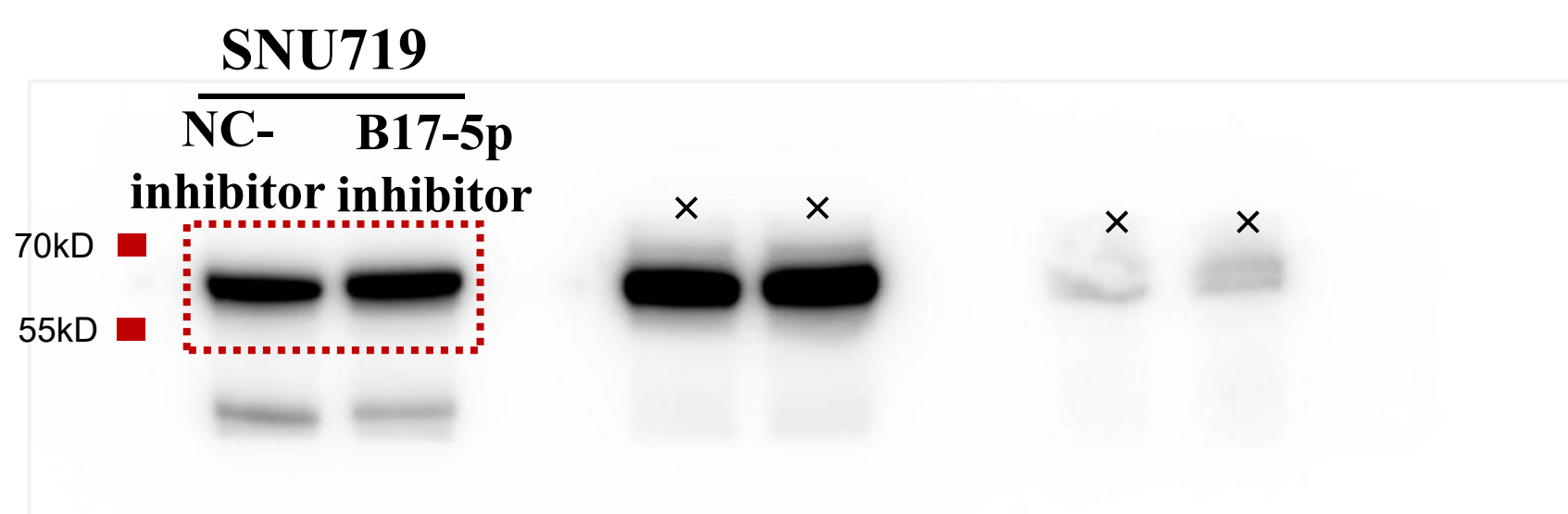

pAKT(Ser473)
(60kDa)

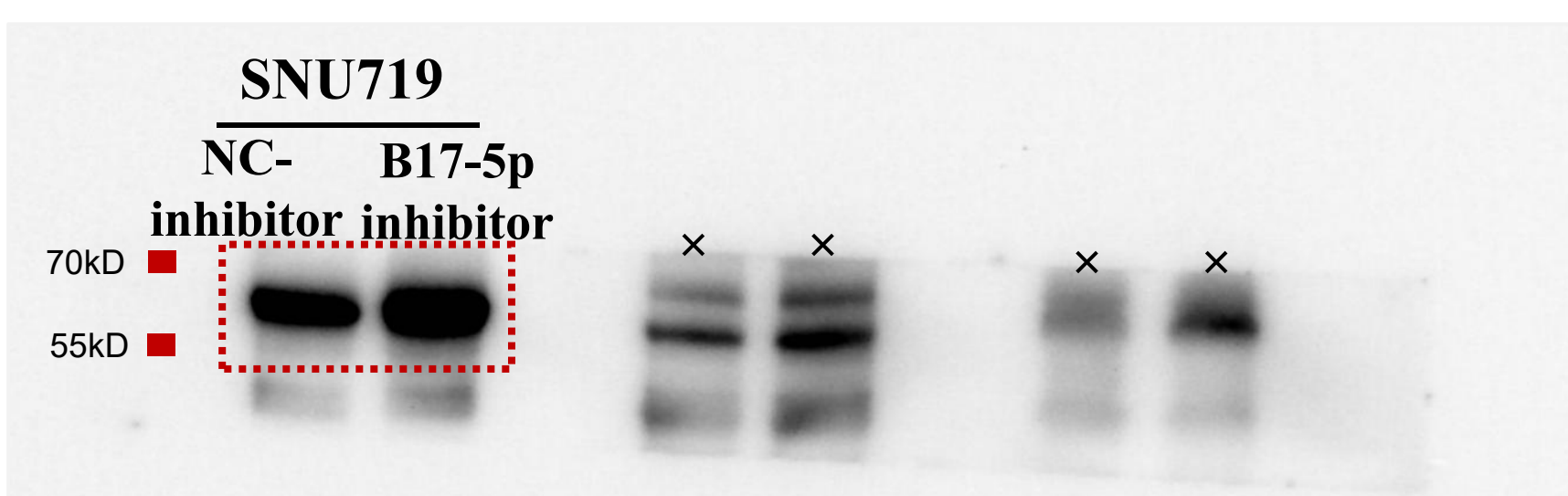

GAPDH
(37kDa)

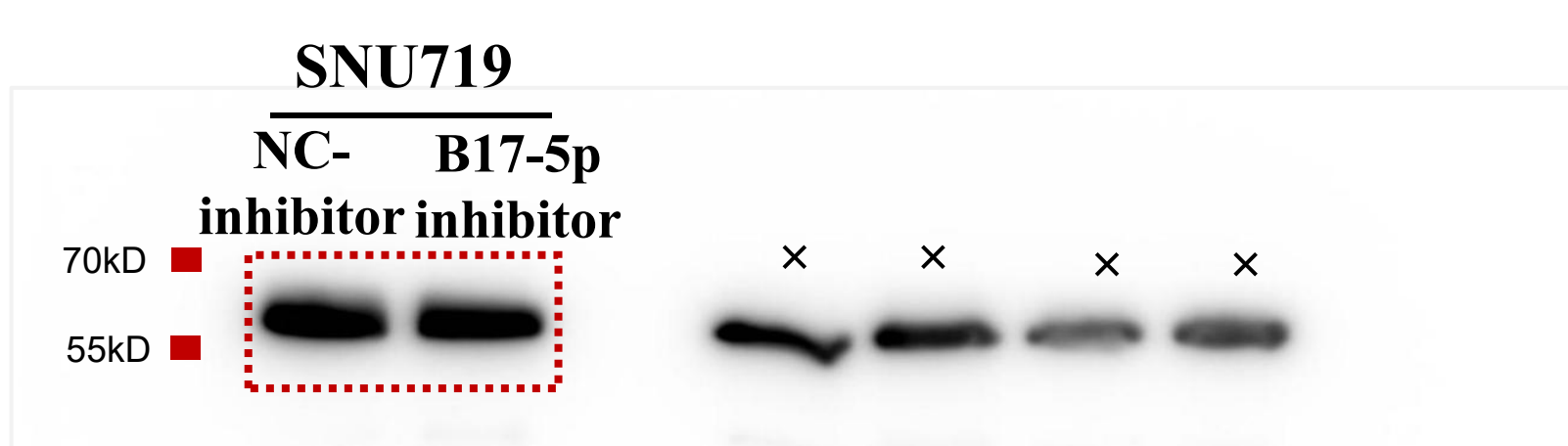

This image corresponds to Figure 6G

Biological Replicate #3

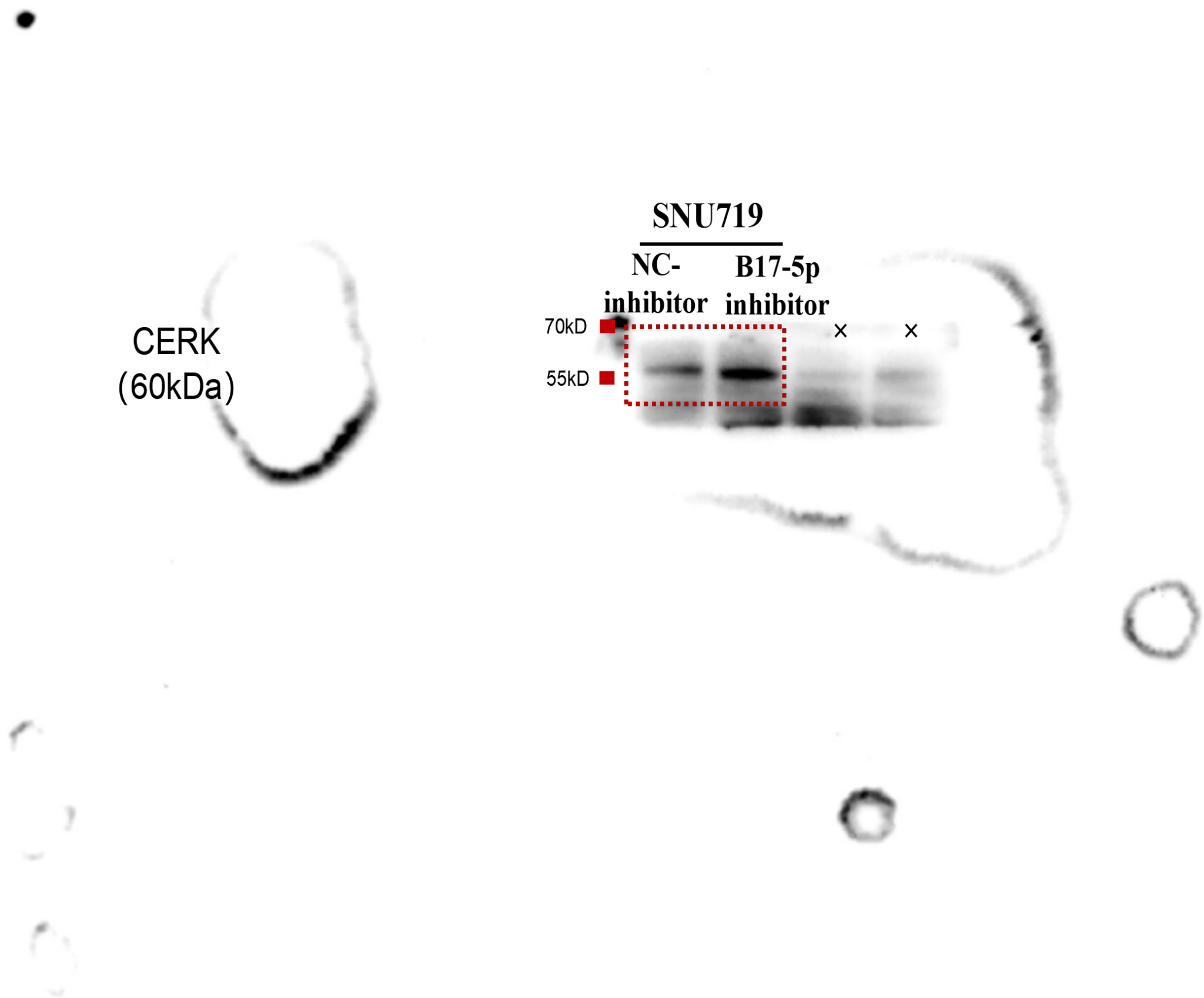

This image corresponds to Figure 6G

Biological Replicate #3

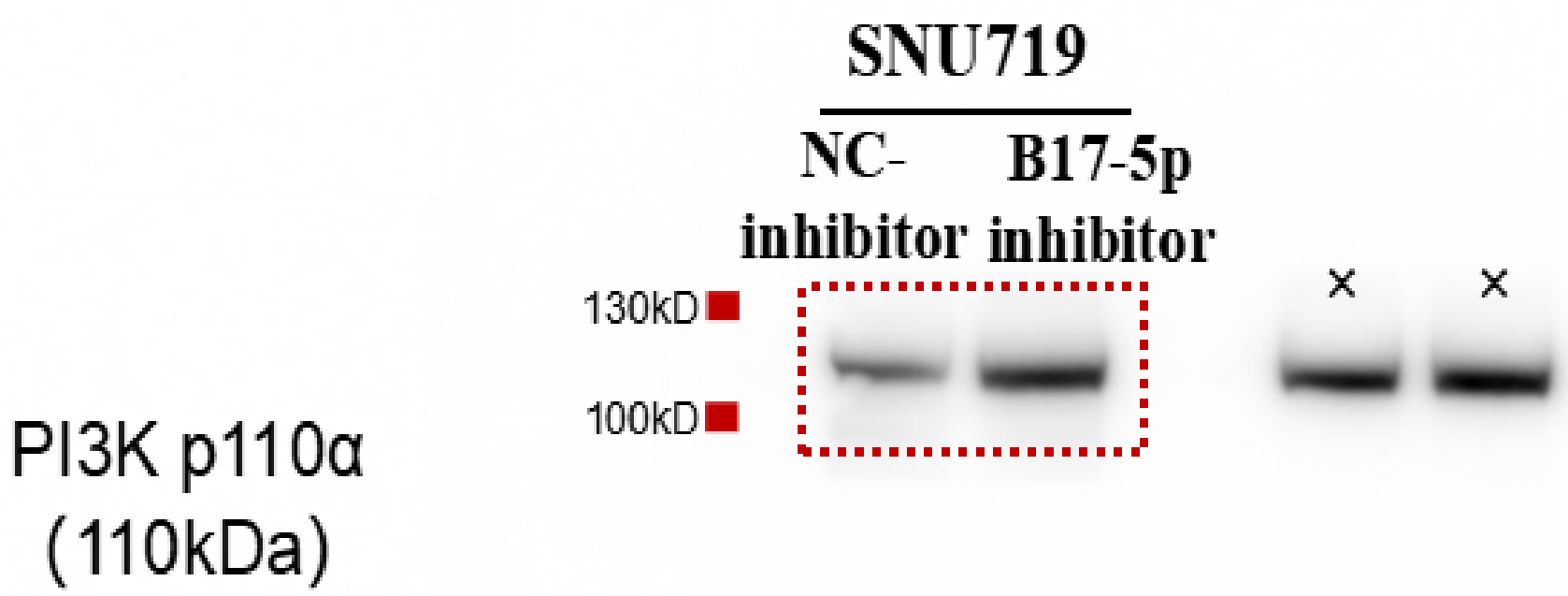

This image corresponds to Figure 6G

Biological Replicate #3

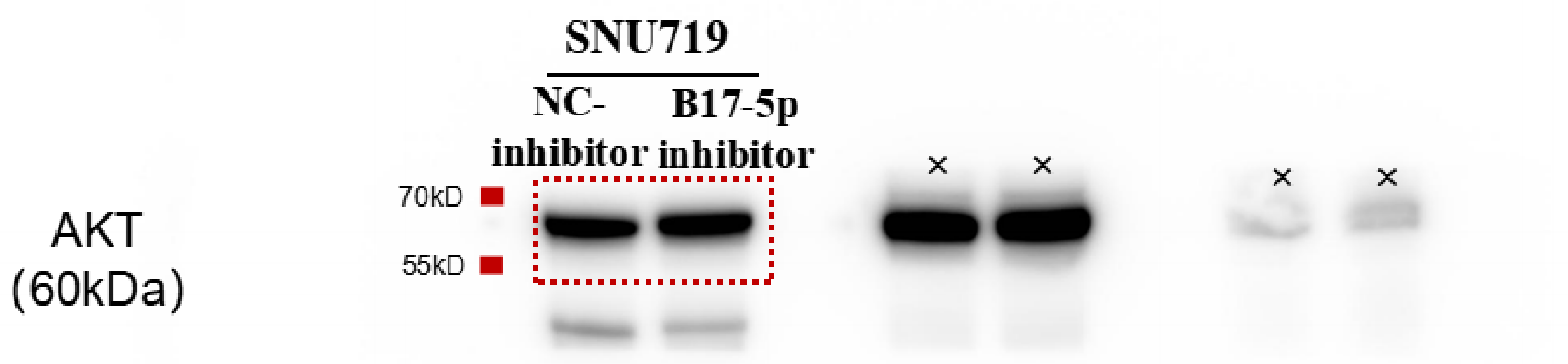

This image corresponds to Figure 6G

Biological Replicate #3

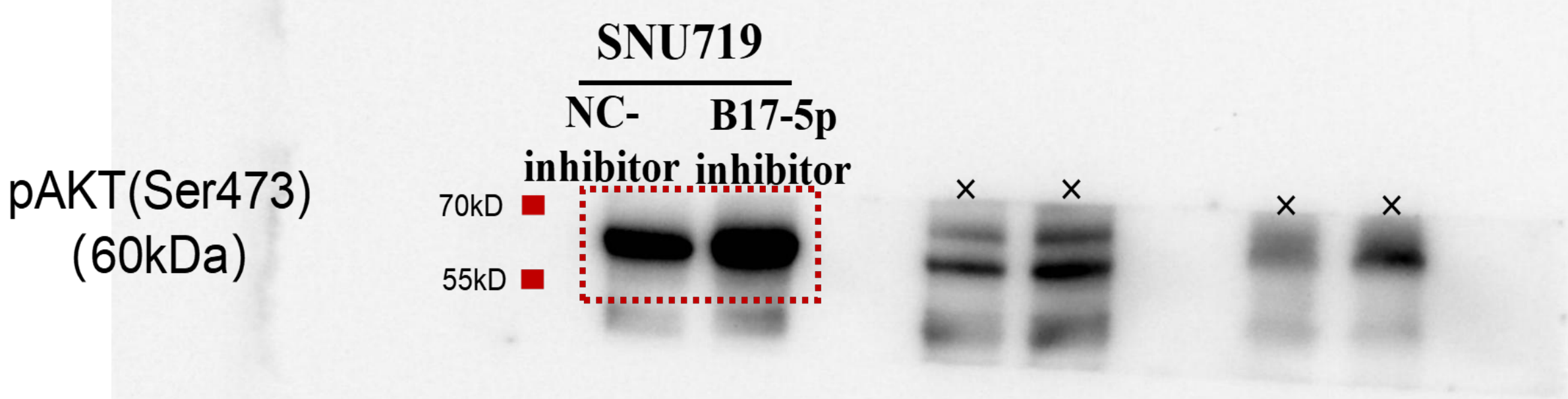

This image corresponds to Figure 6G

Biological Replicate #3

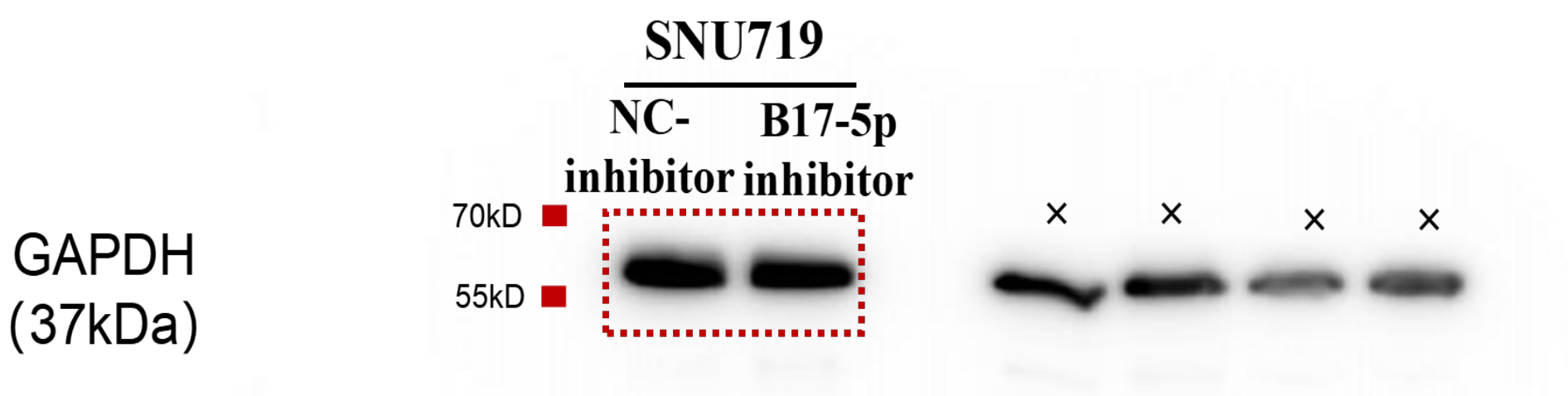

These images correspond to Figure 6I

Biological Replicate #1

▲ These images was selected as the representative result in Figure 6I.

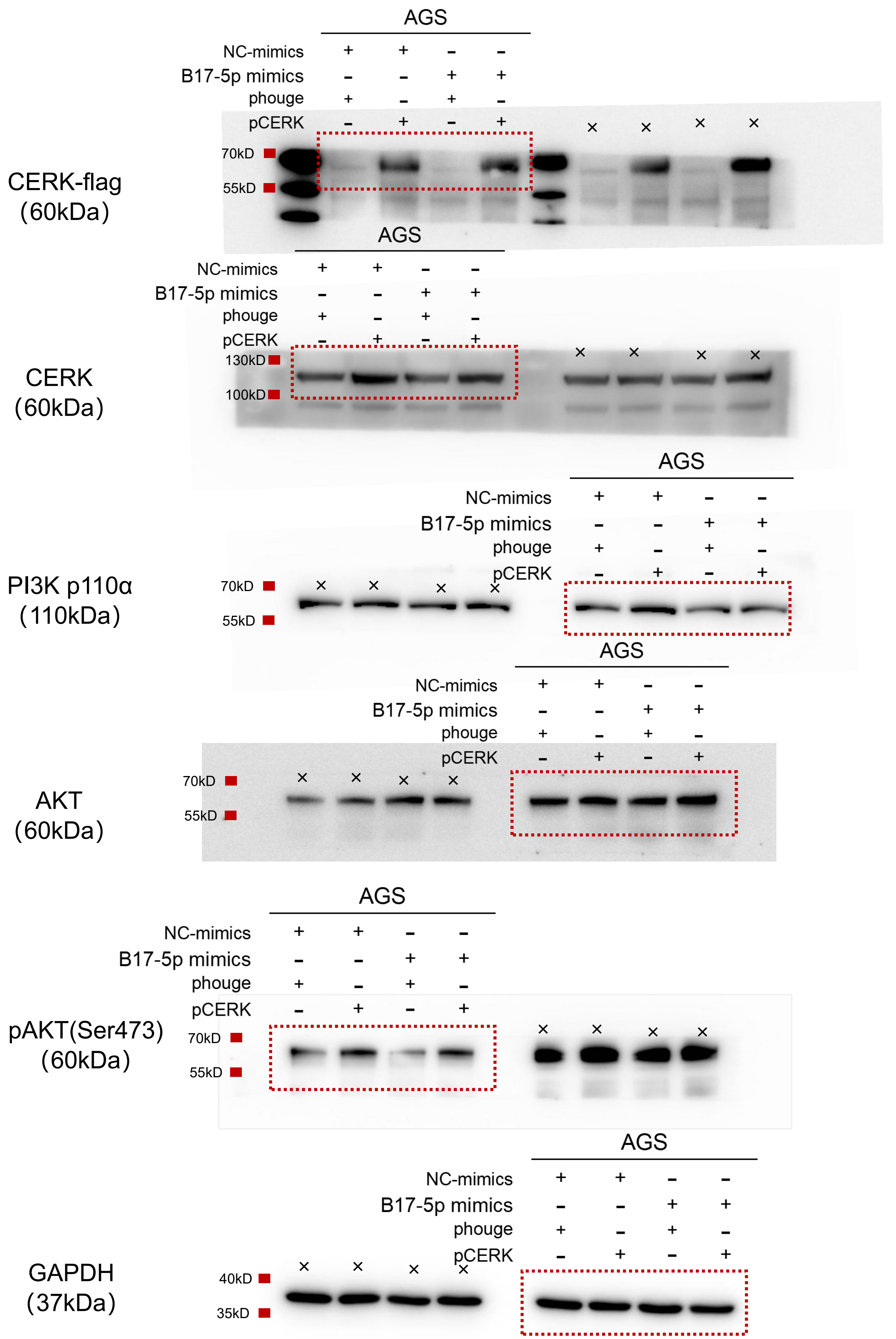

This image corresponds to Figure 6I

Biological Replicate #1

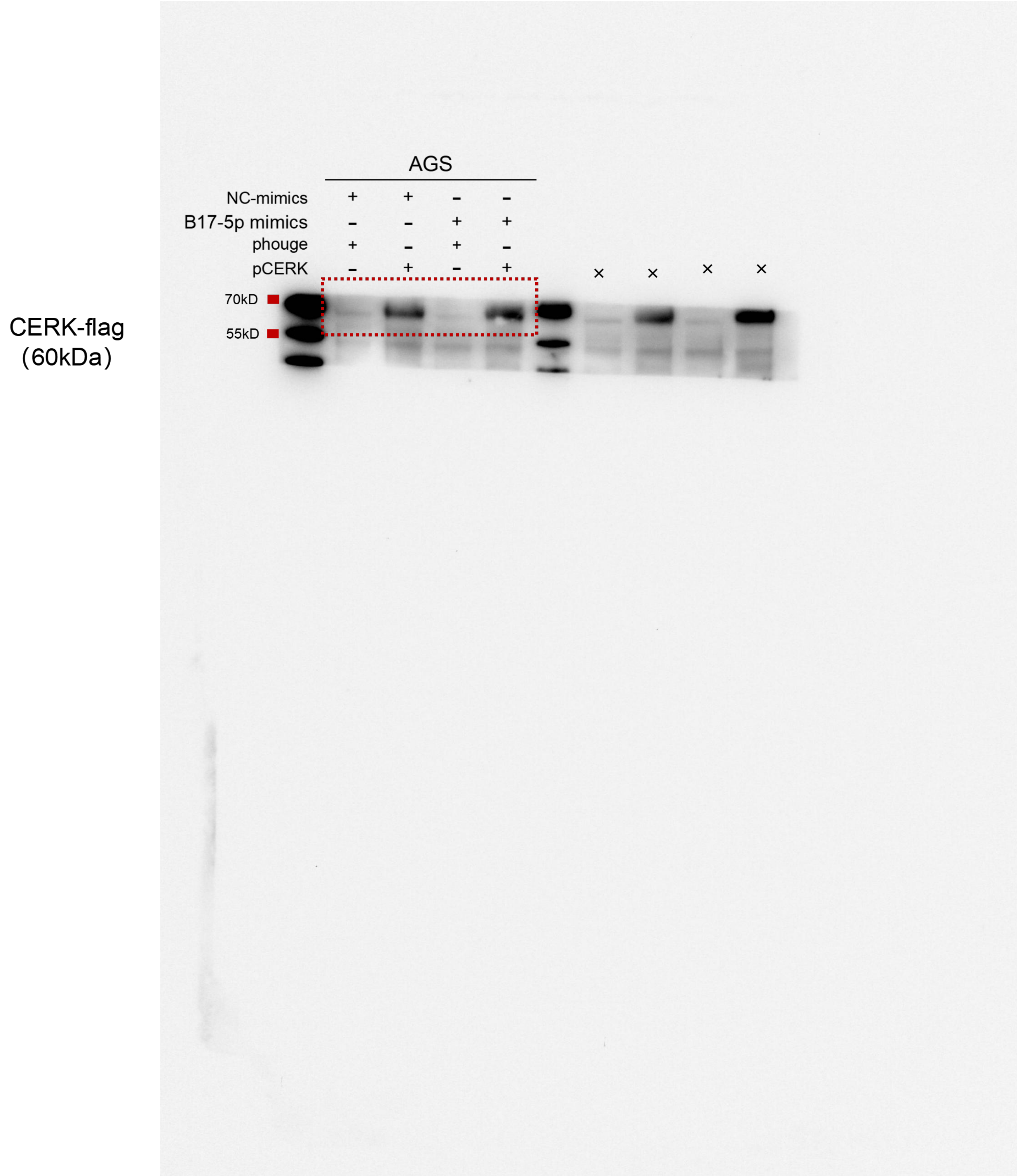

This image corresponds to Figure 6I

Biological Replicate #1

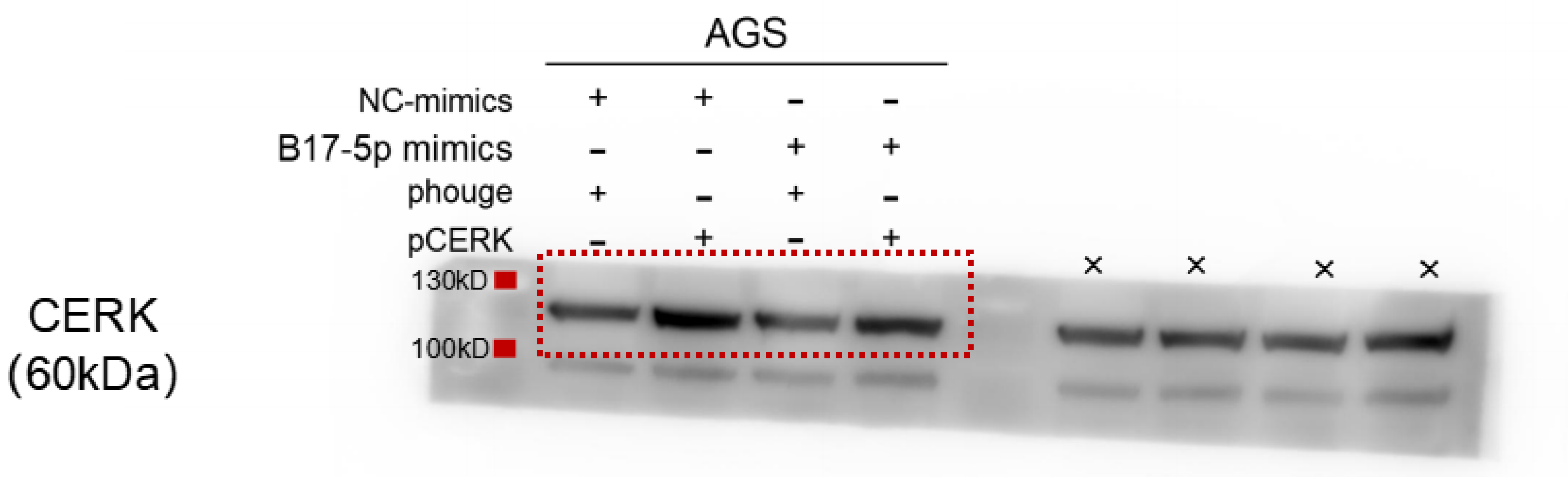

This image corresponds to Figure 6I

Biological Replicate #1

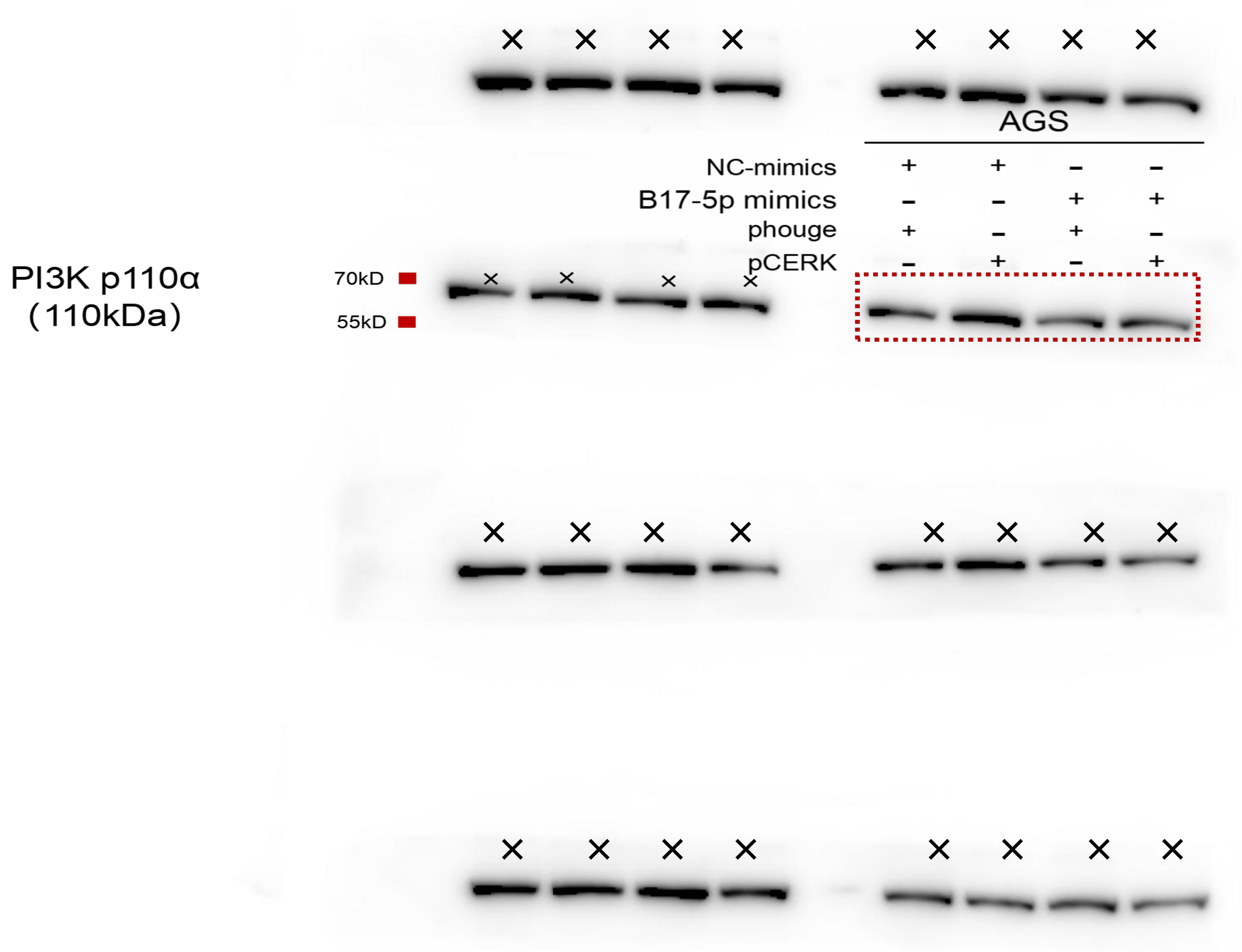

This image corresponds to Figure 6I

Biological Replicate #1

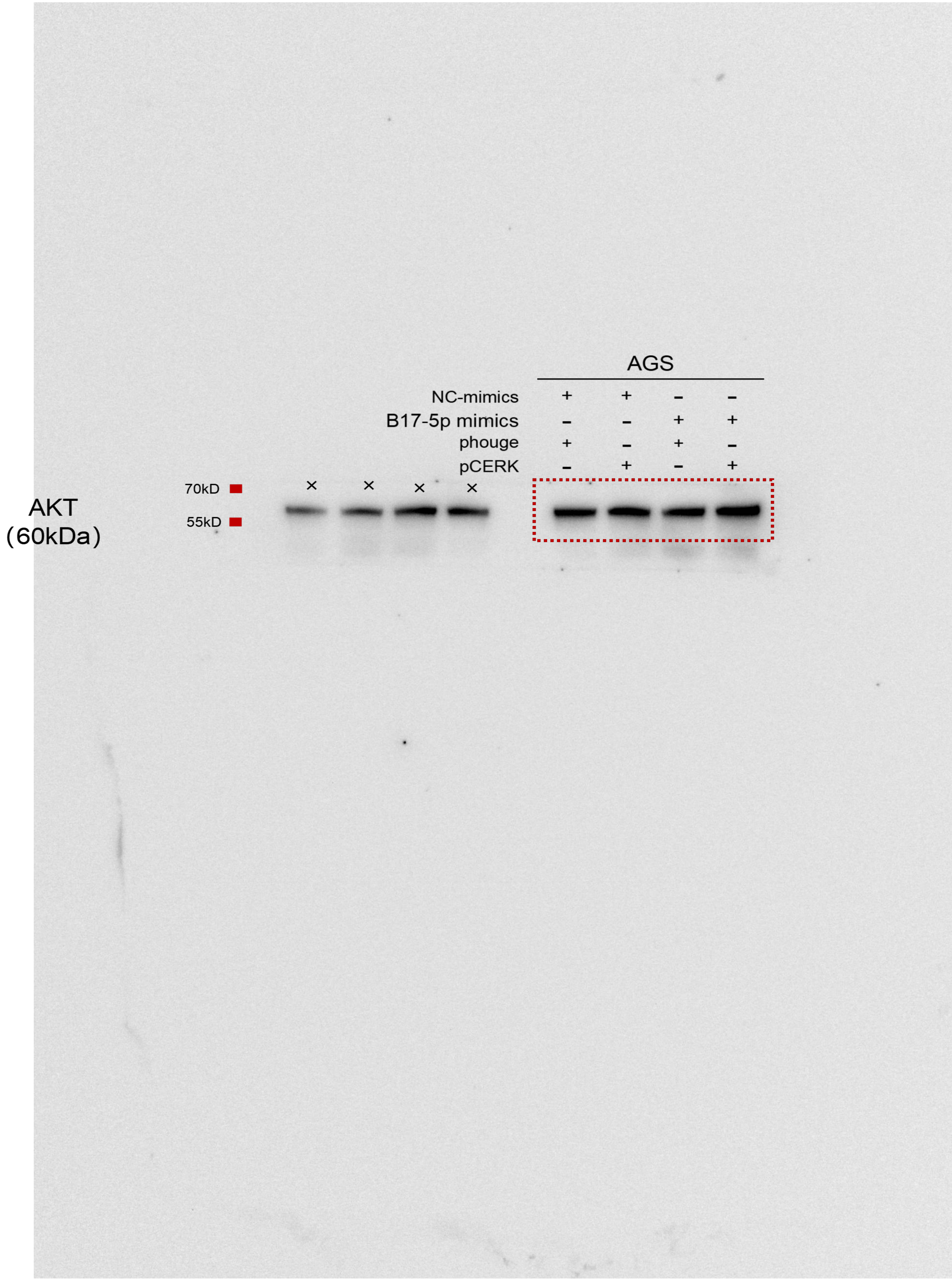

This image corresponds to Figure 6I

Biological Replicate #1

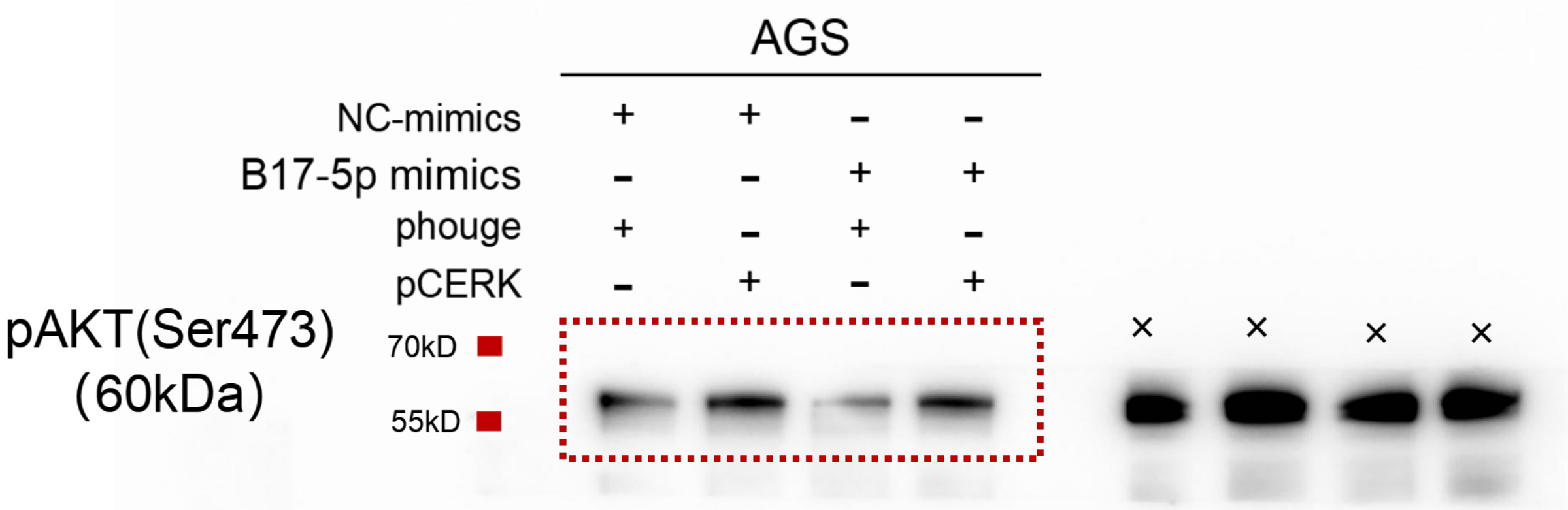

This image corresponds to Figure 6I

Biological Replicate #1

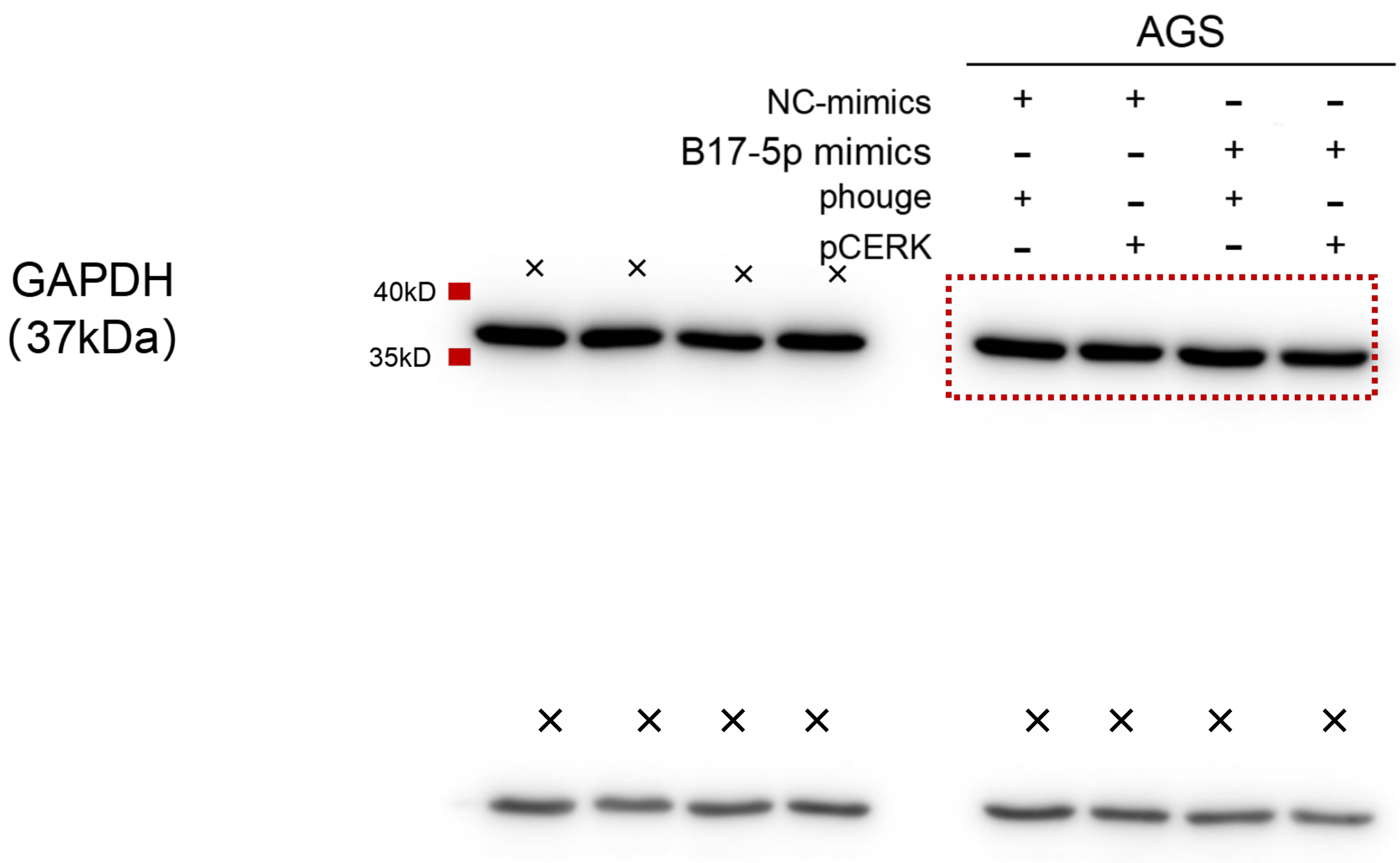

These images correspond to Figure 6I

Biological Replicate #2

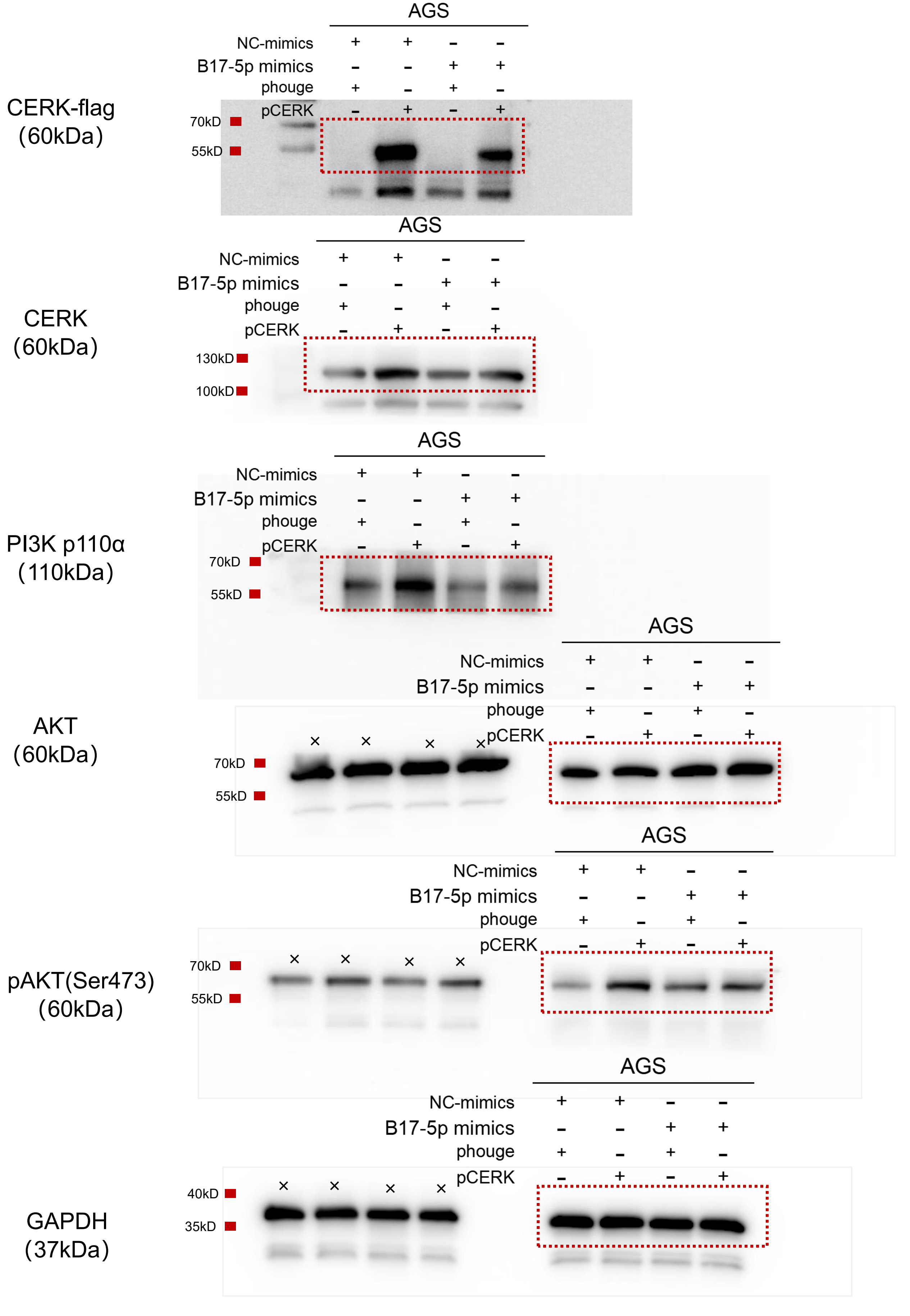

This image corresponds to Figure 6I

Biological Replicate #2

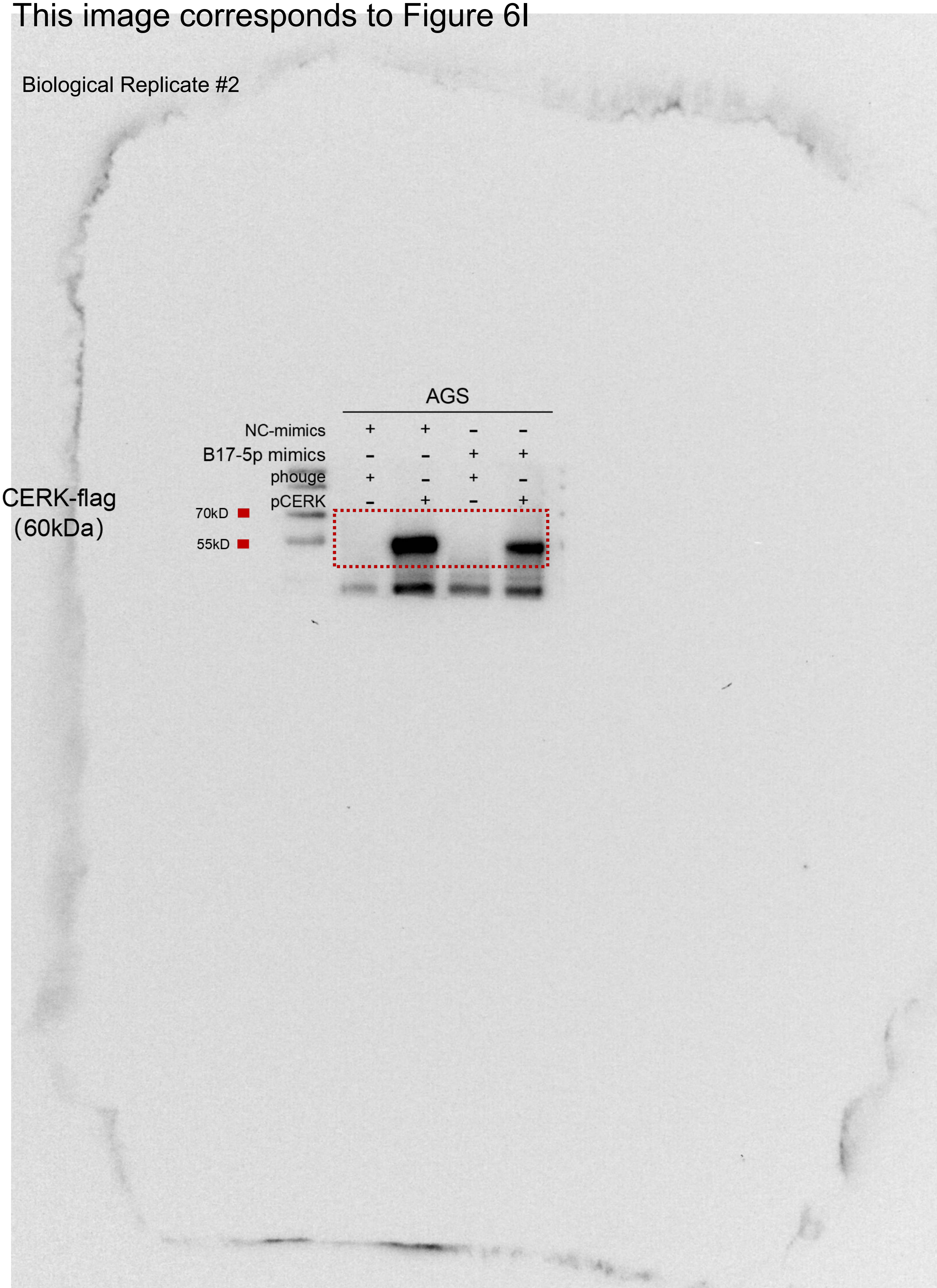

This image corresponds to Figure 6I

Biological Replicate #2

CERK
(60kDa)

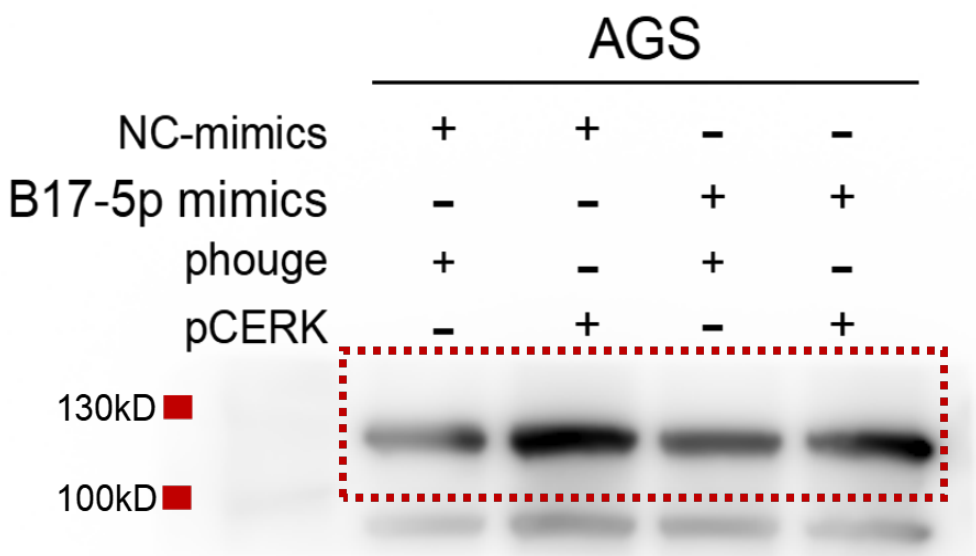

This image corresponds to Figure 6I

Biological Replicate #2

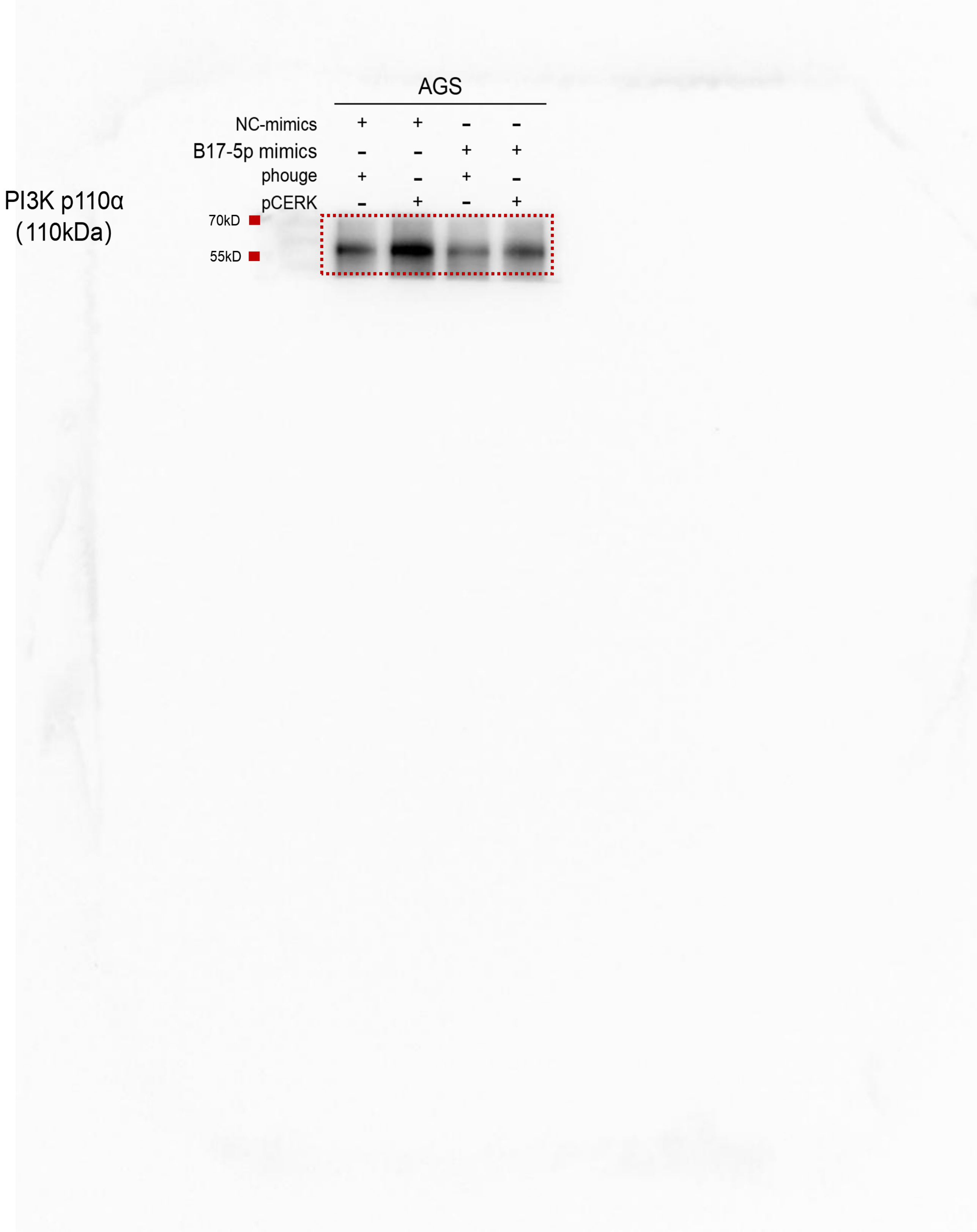

This image corresponds to Figure 6I

Biological Replicate #2

AKT
(60kDa)

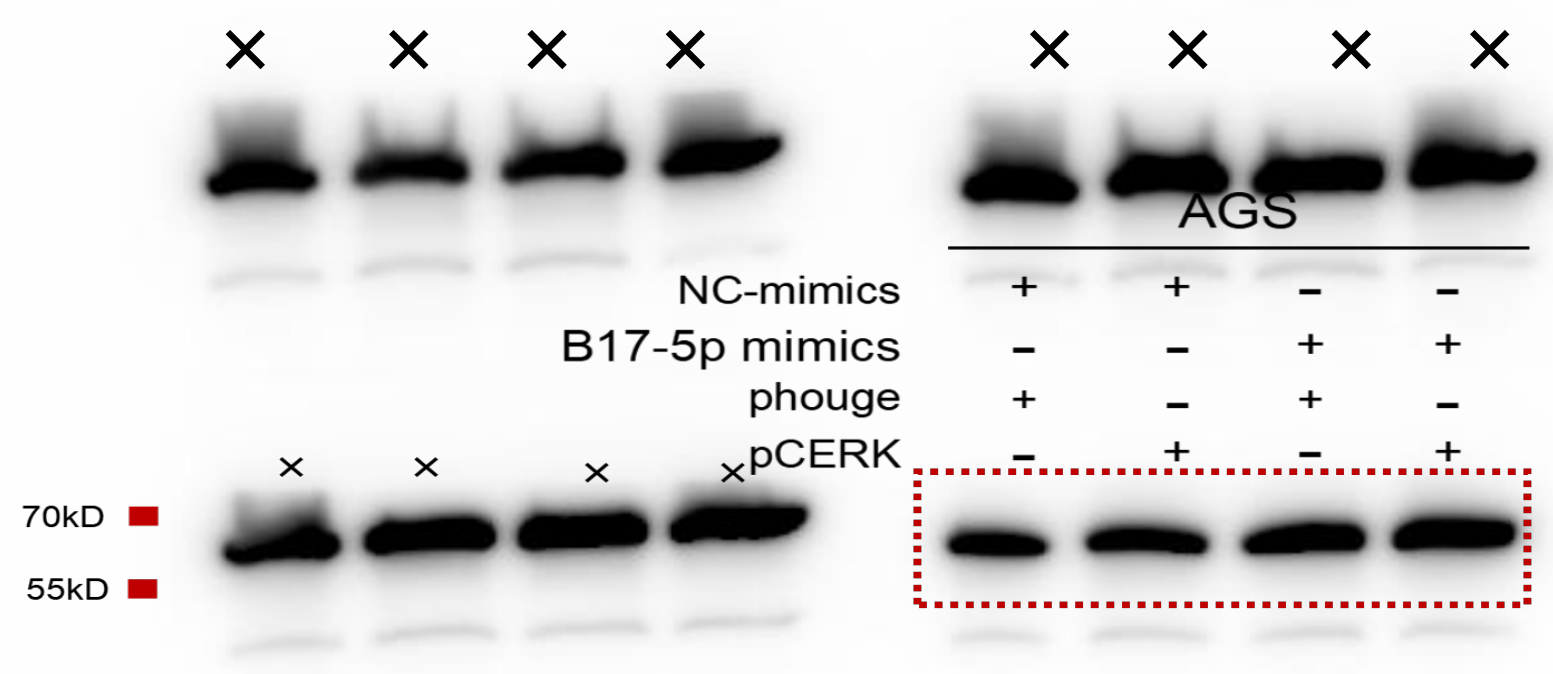

This image corresponds to Figure 6I

Biological Replicate #2

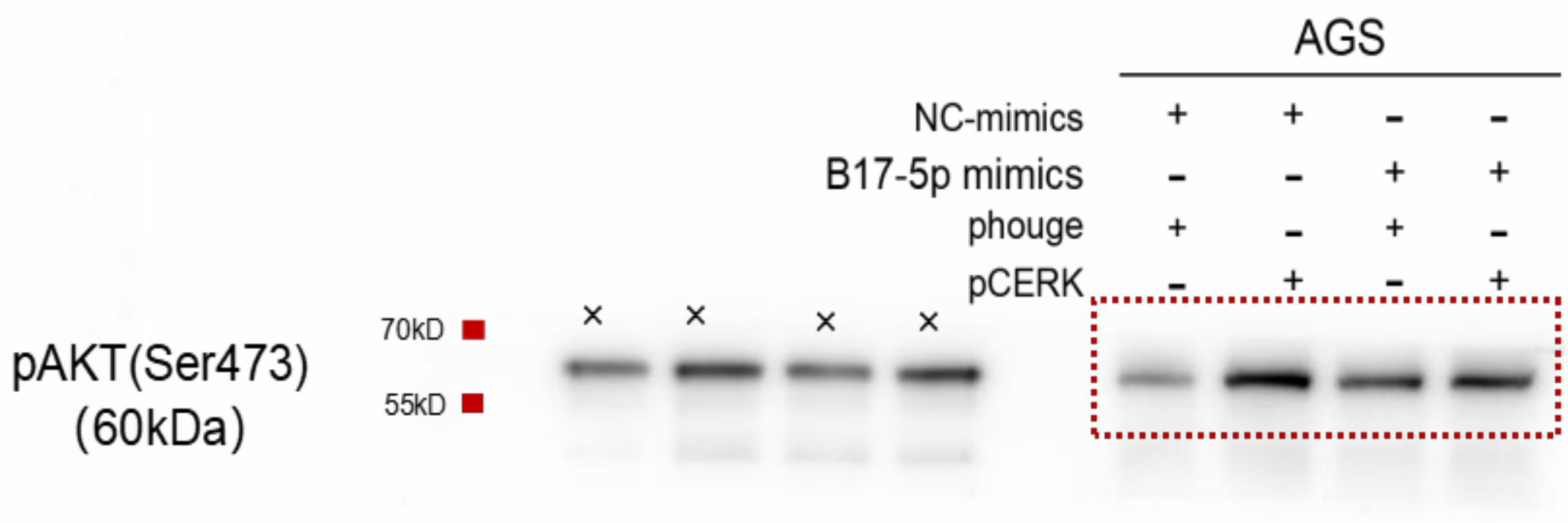

This image corresponds to Figure 6I

Biological Replicate #2

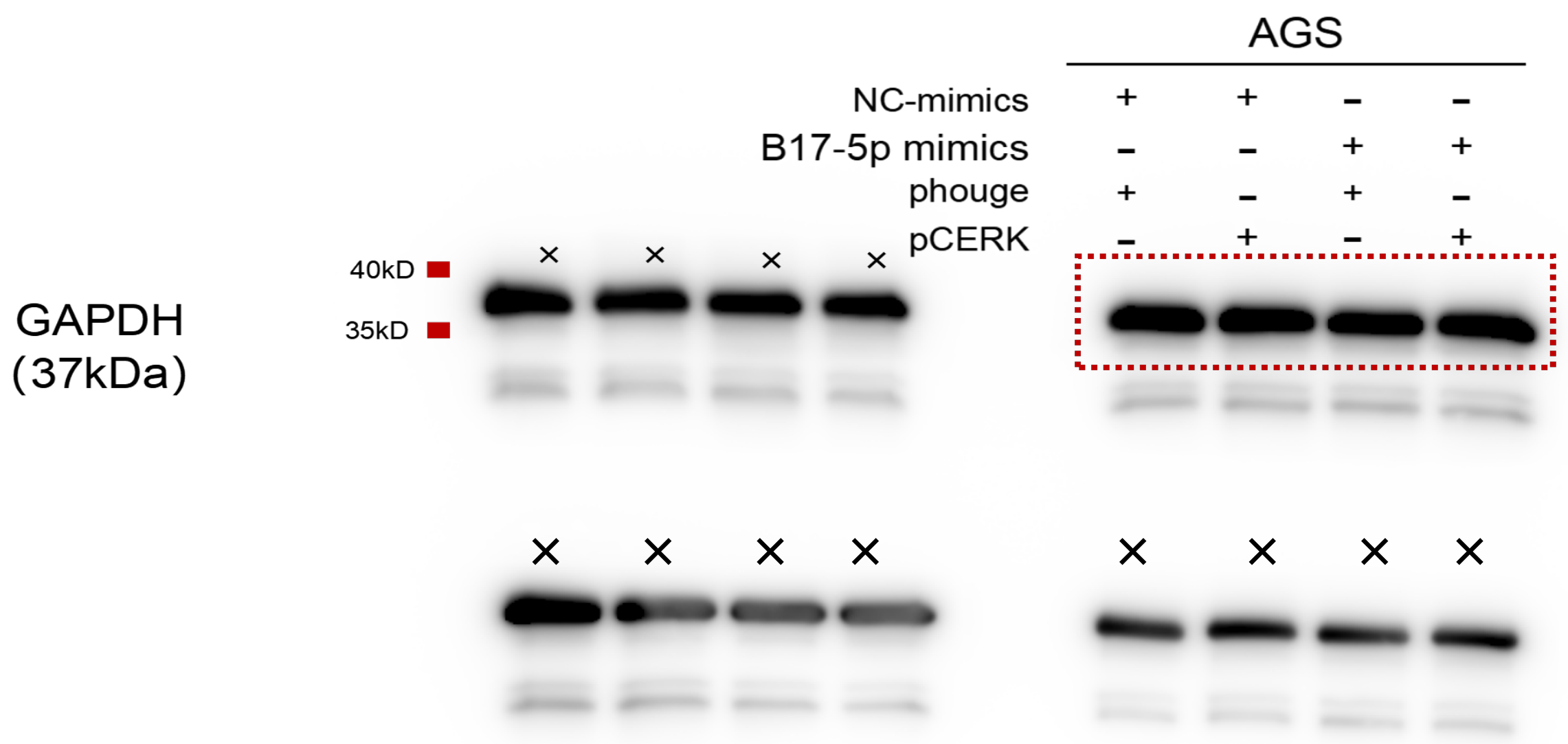

These images correspond to Figure 6I

Biological Replicate #3

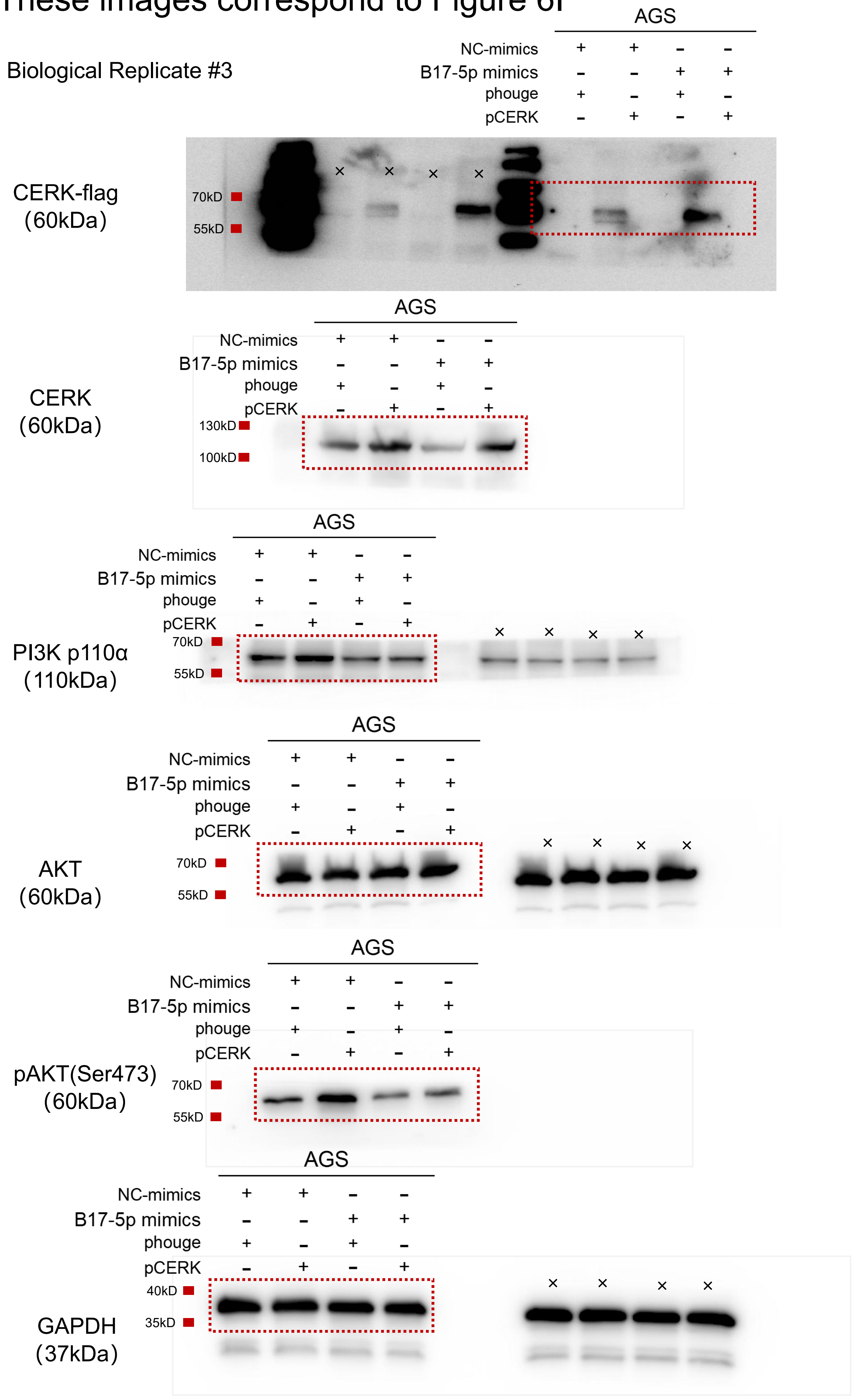

This image corresponds to Figure 6I

Biological Replicate #3

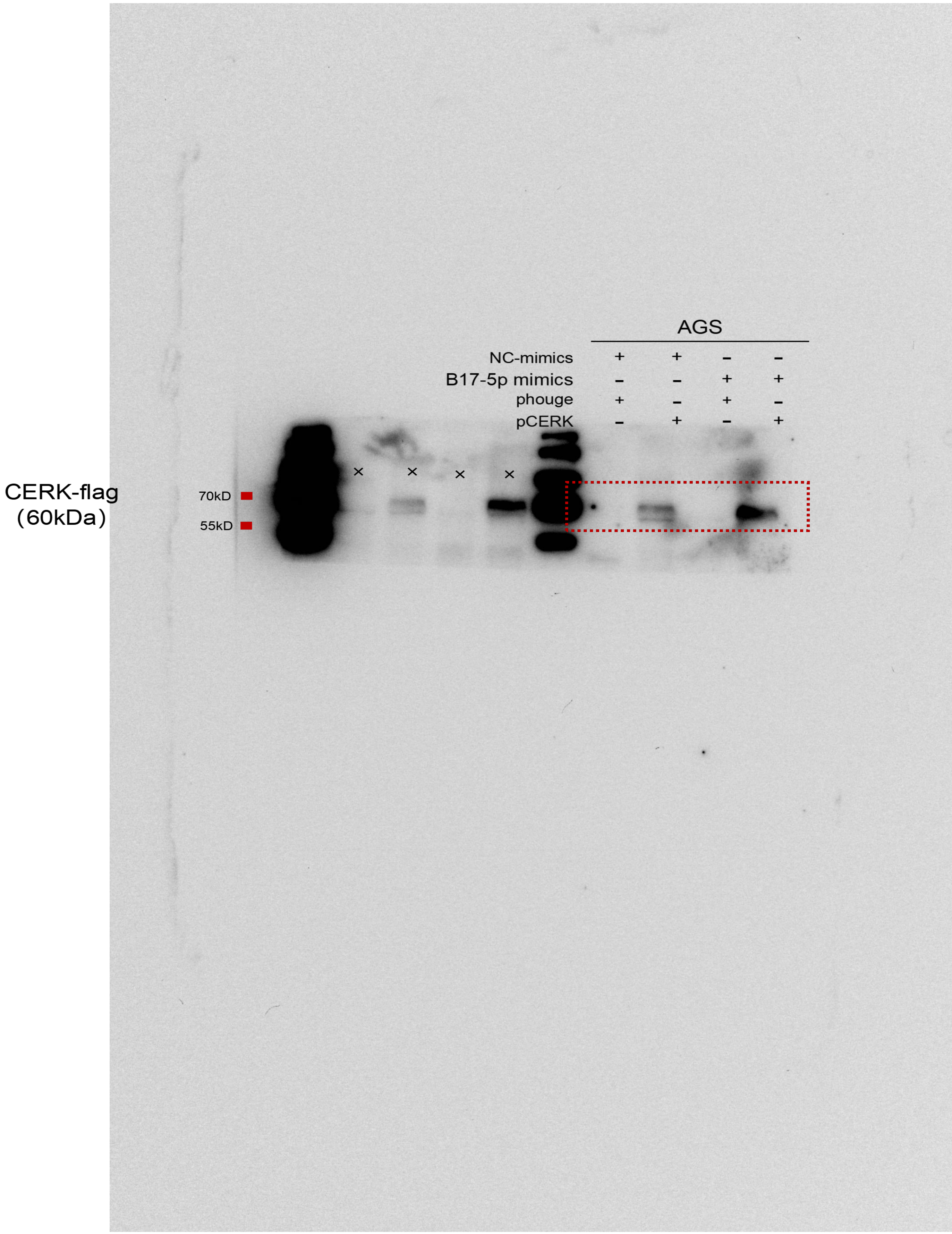

This image corresponds to Figure 6I

Biological Replicate #3

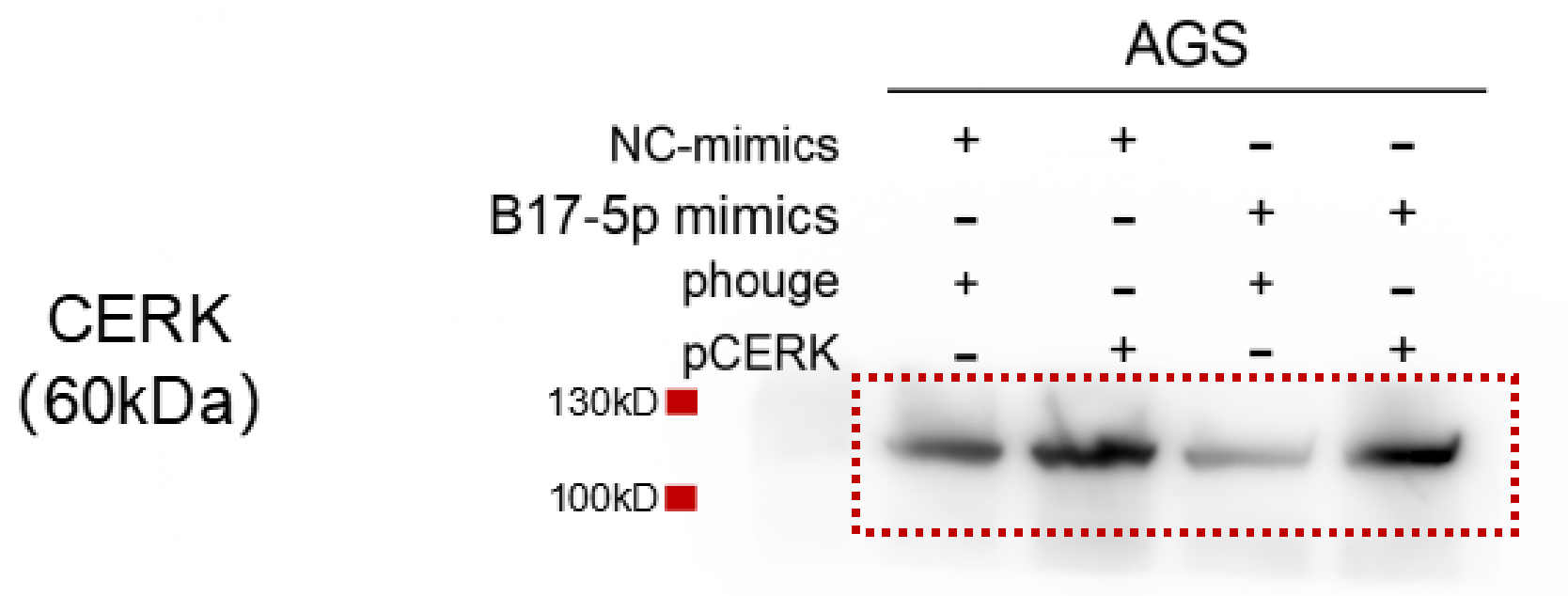

This image corresponds to Figure 6I

Biological Replicate #3

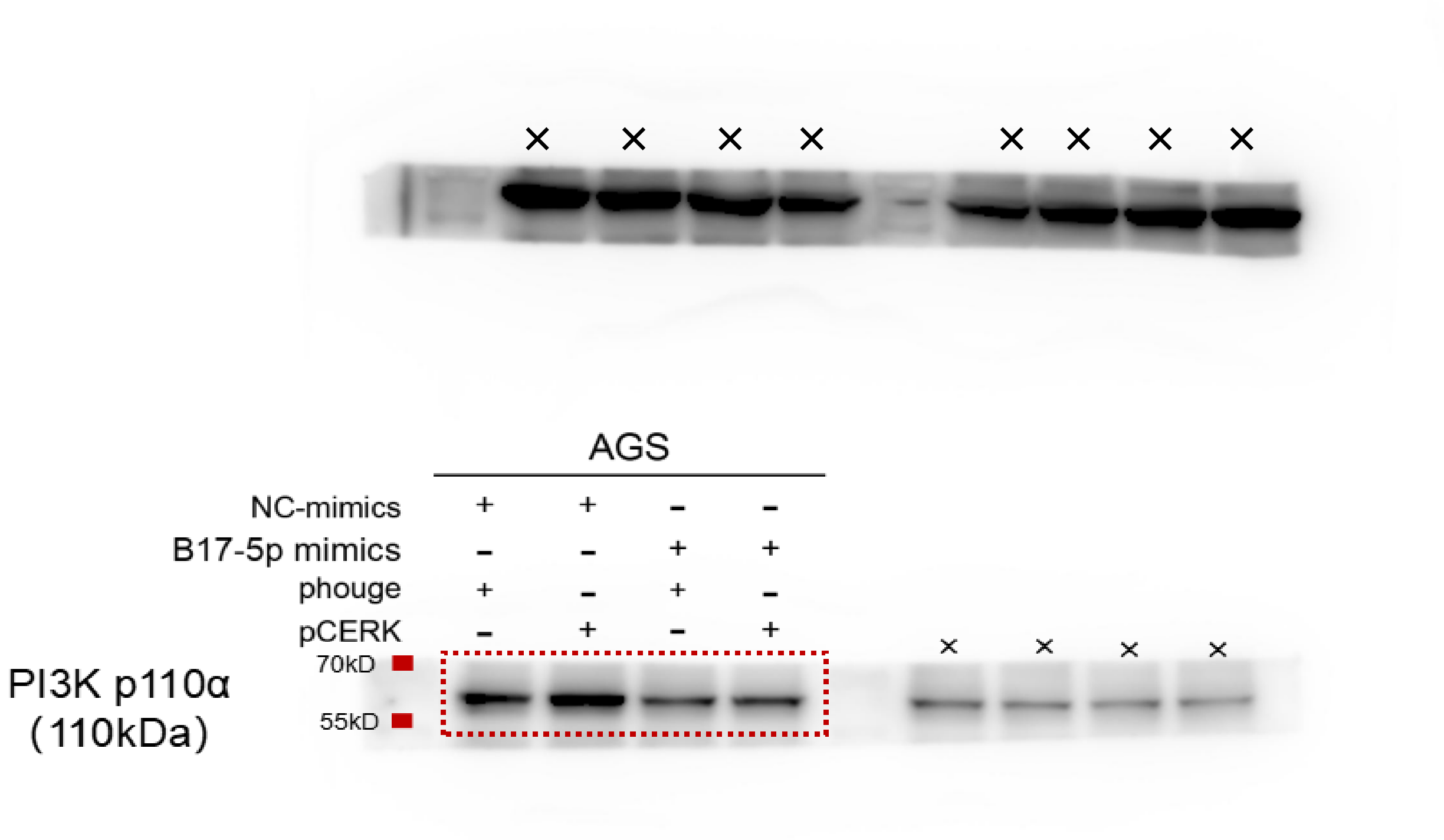

This image corresponds to Figure 6I

Biological Replicate #3

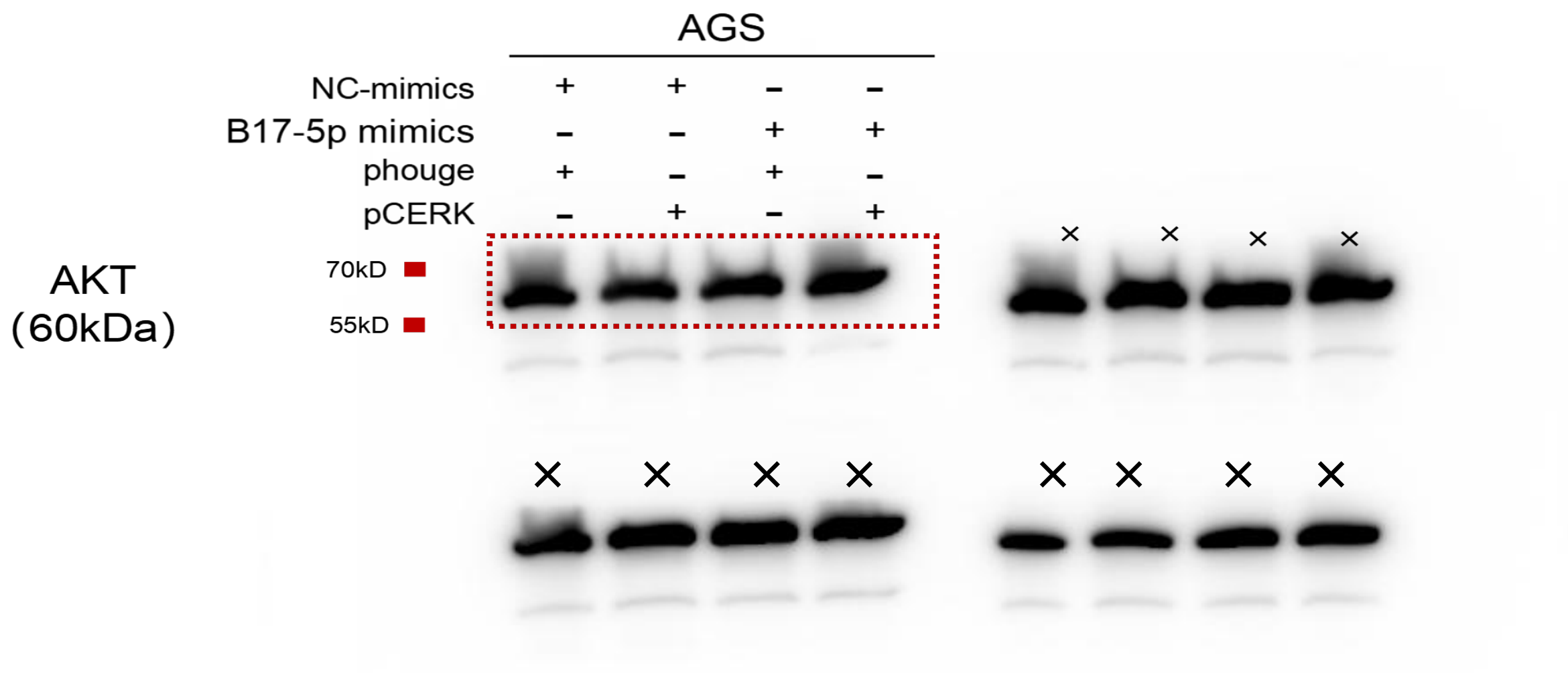

This image corresponds to Figure 6I

Biological Replicate #3

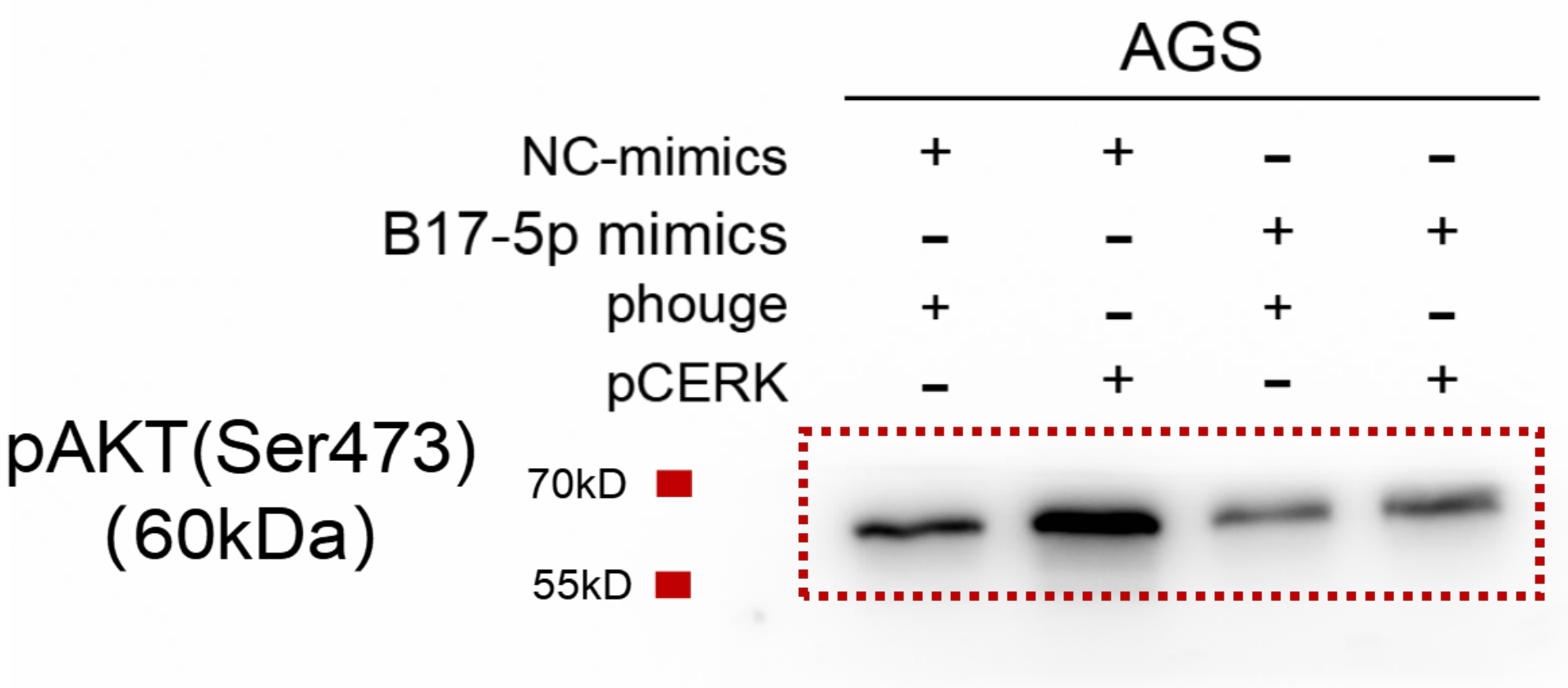

This image corresponds to Figure 6I

Biological Replicate #3

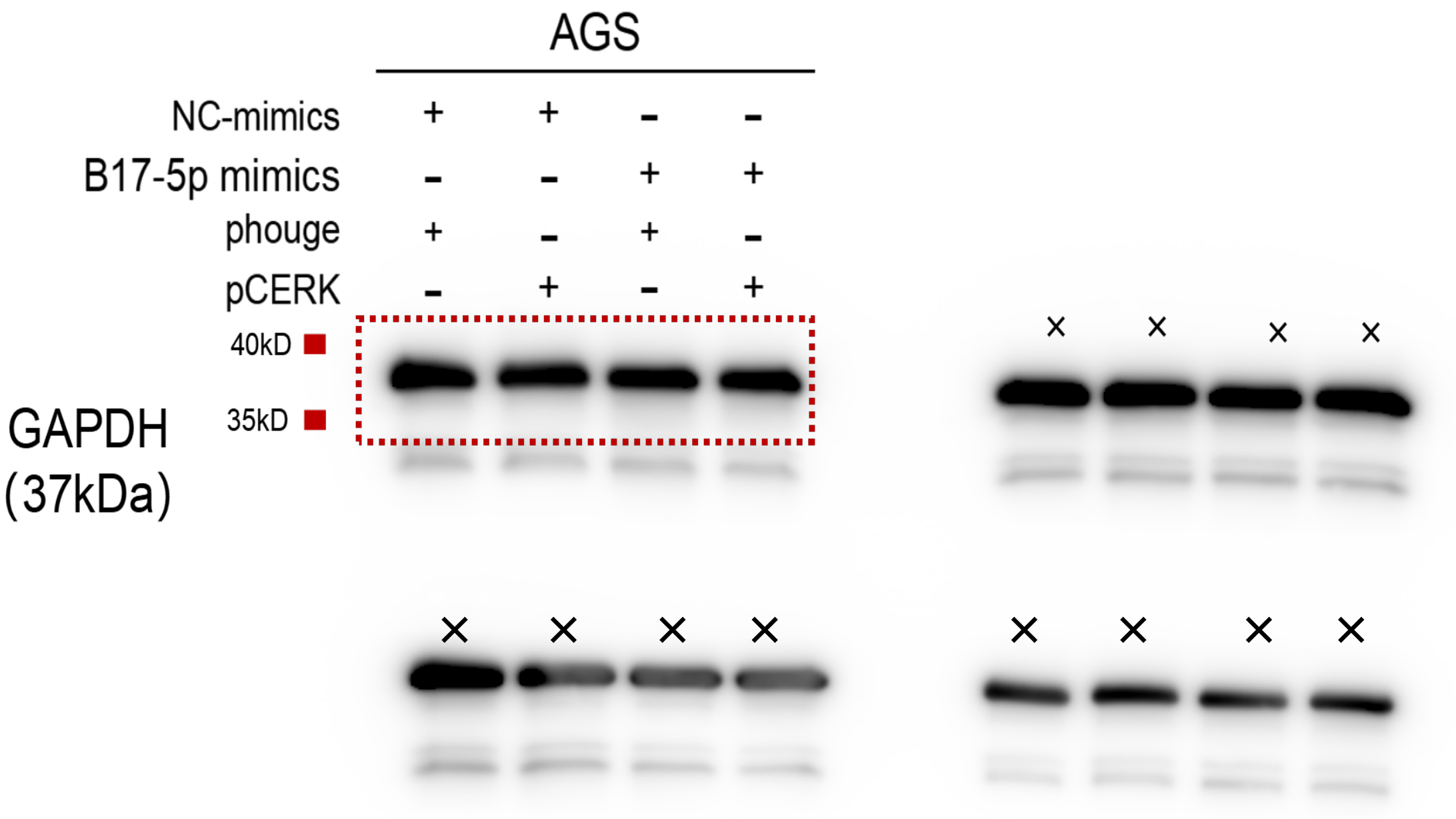

These images correspond to Figure 7A

Biological Replicate #1

▲ These images was selected as the representative result in Figure 6I.

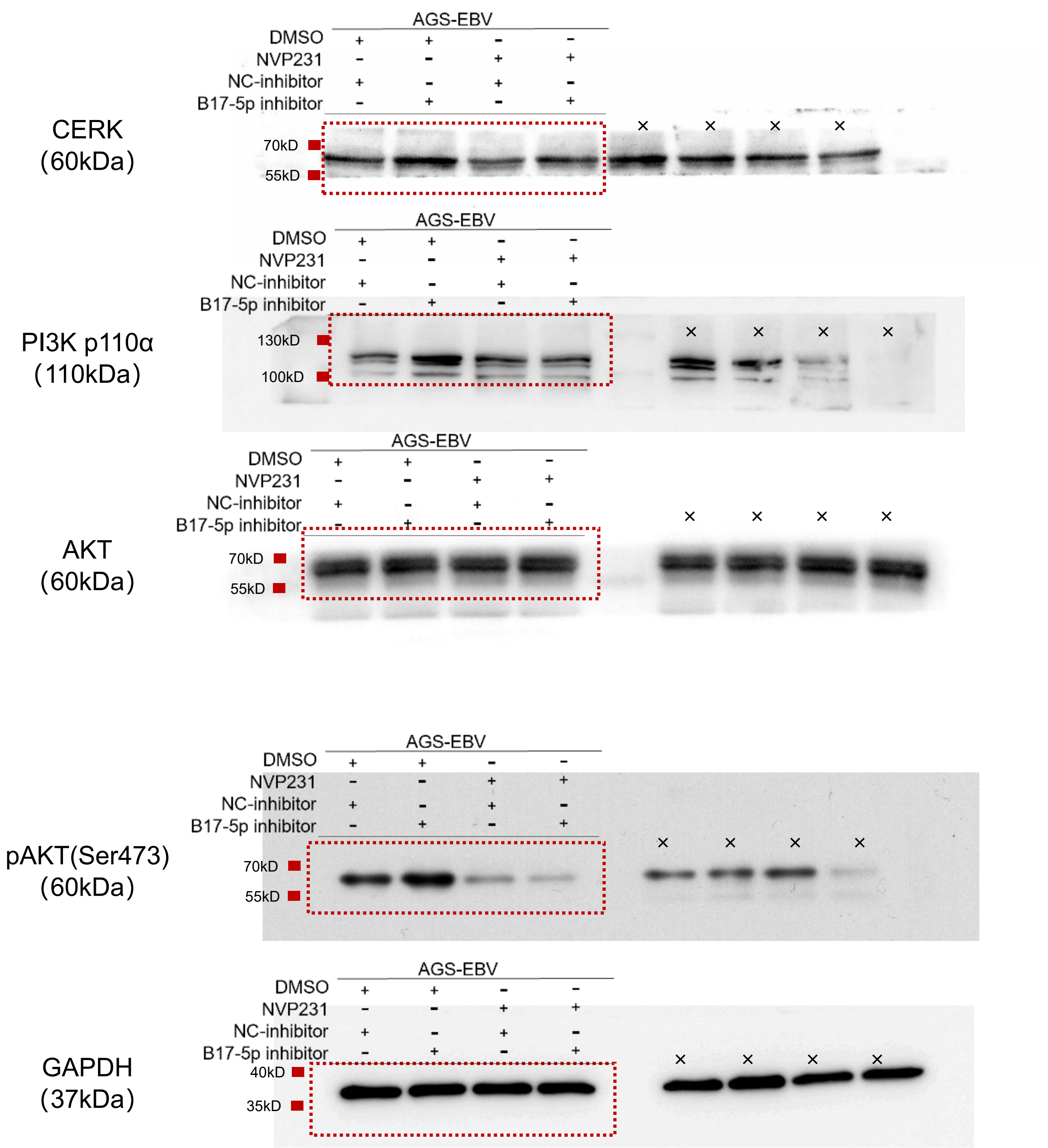

This image corresponds to Figure 7A

Biological Replicate #1

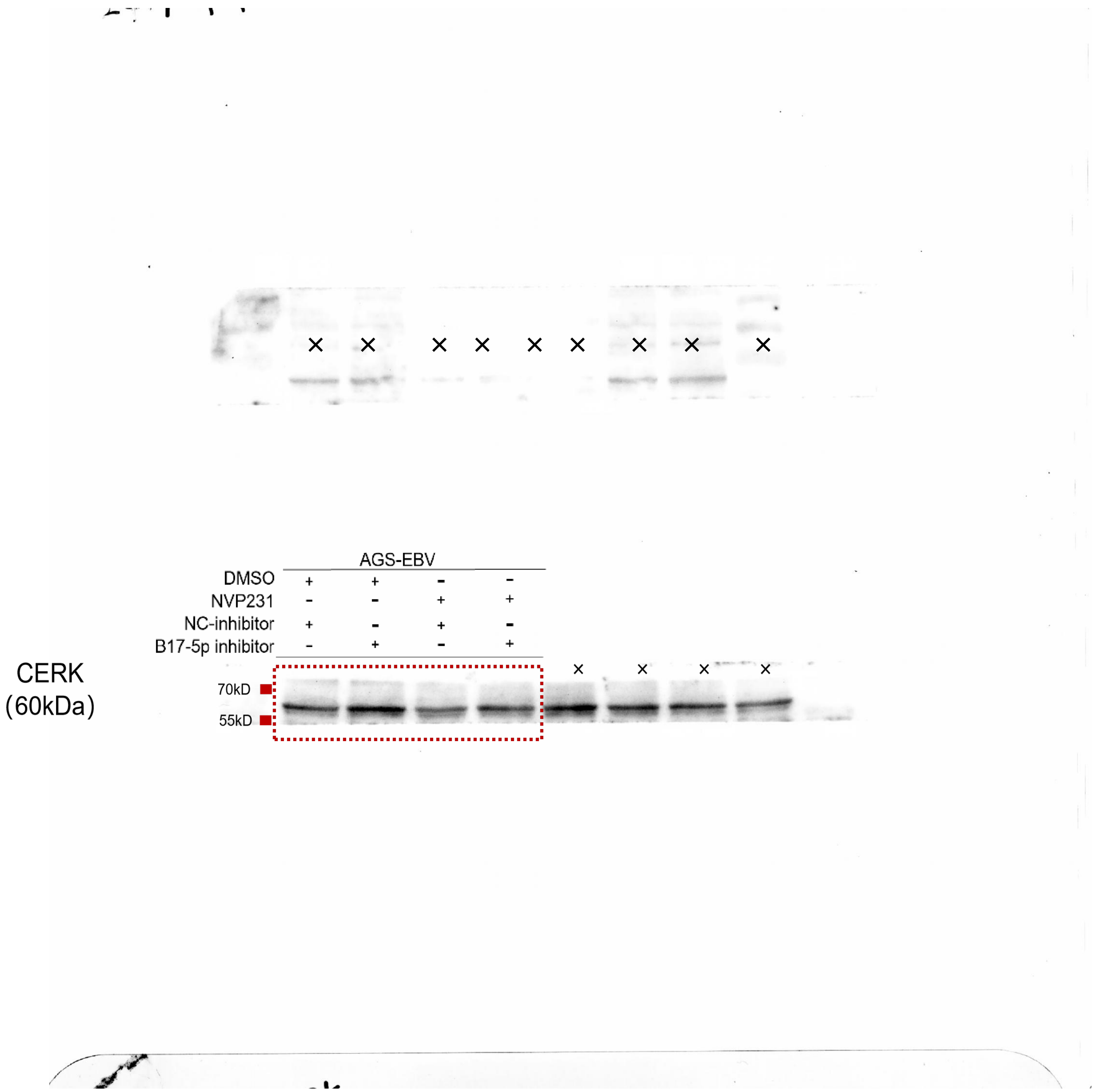

This image corresponds to Figure 7A

Biological Replicate #1

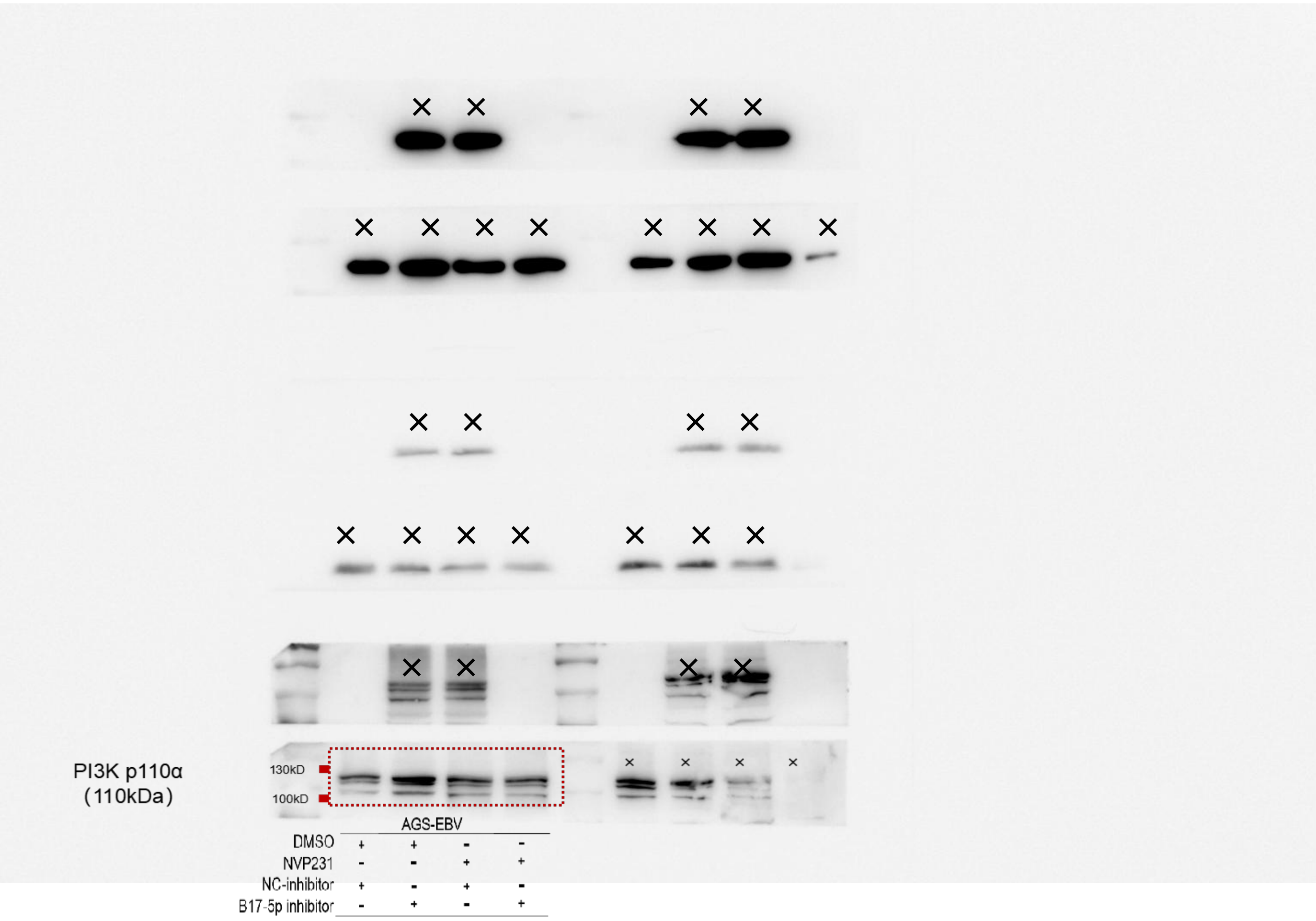

This image corresponds to Figure 7A

Biological Replicate #1

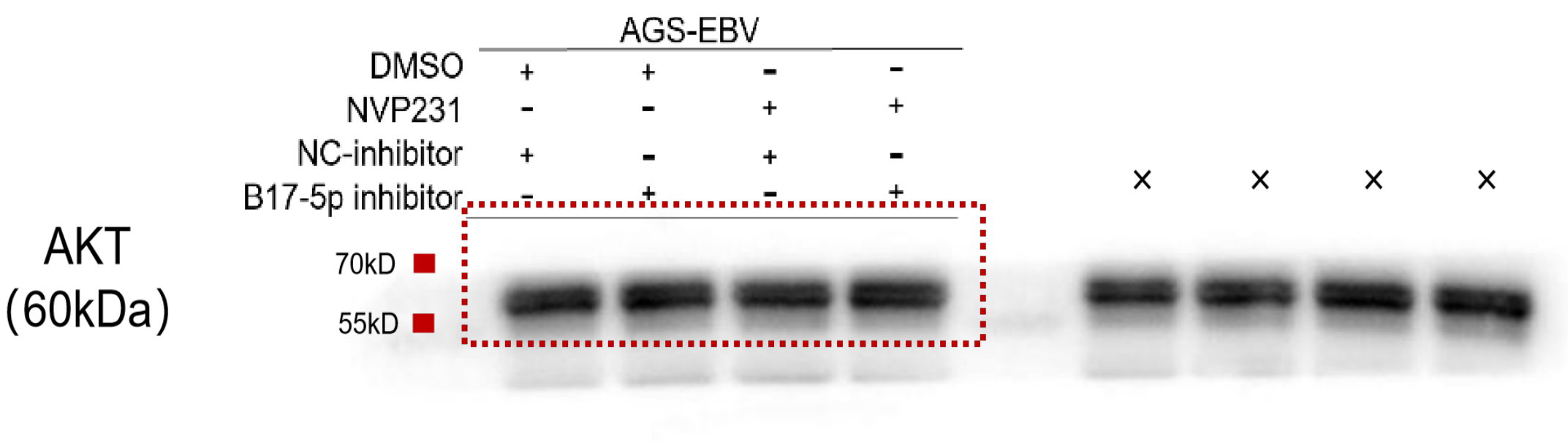

This image corresponds to Figure 7A

Biological Replicate #1

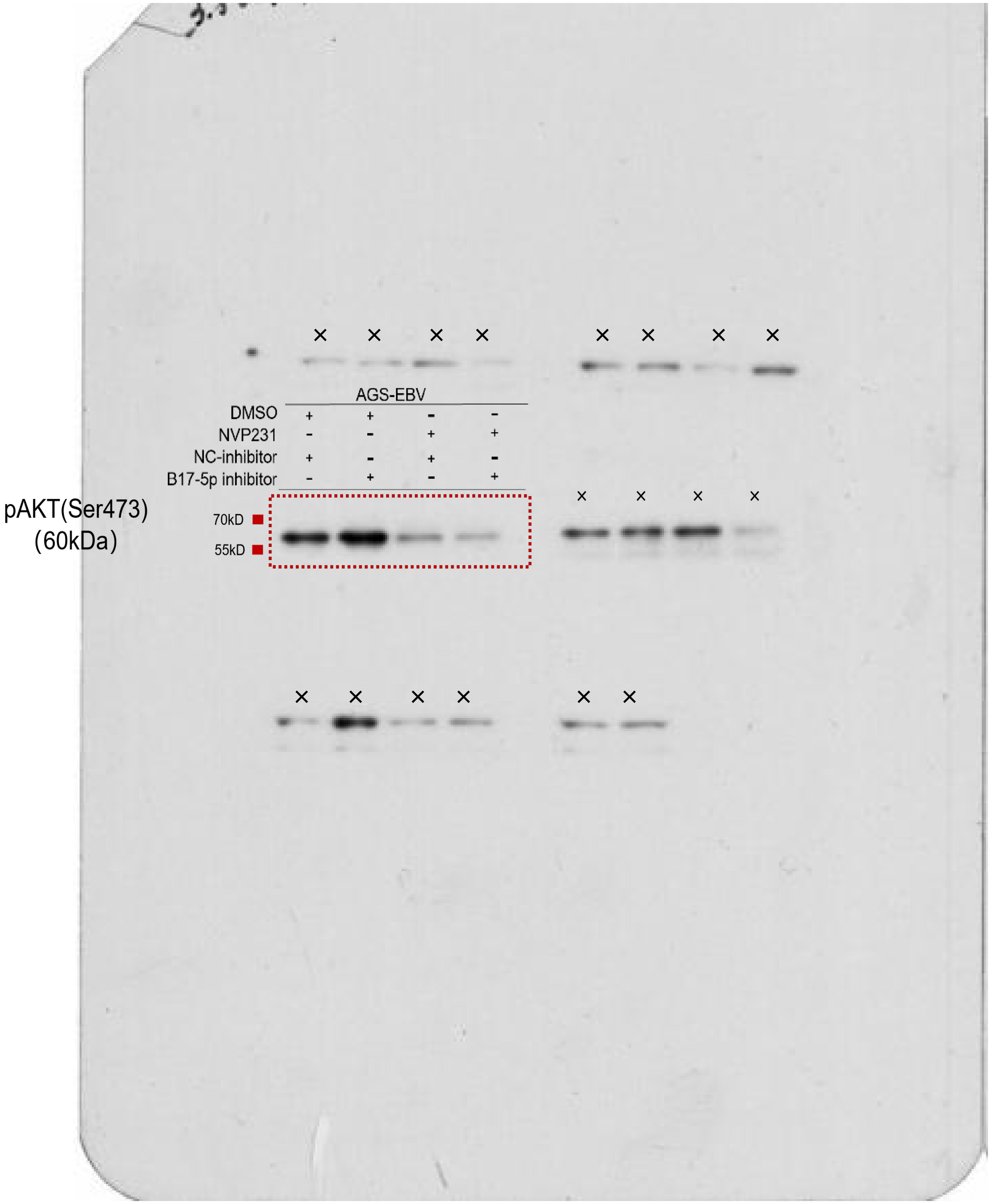

This image corresponds to Figure 7A

Biological Replicate #1

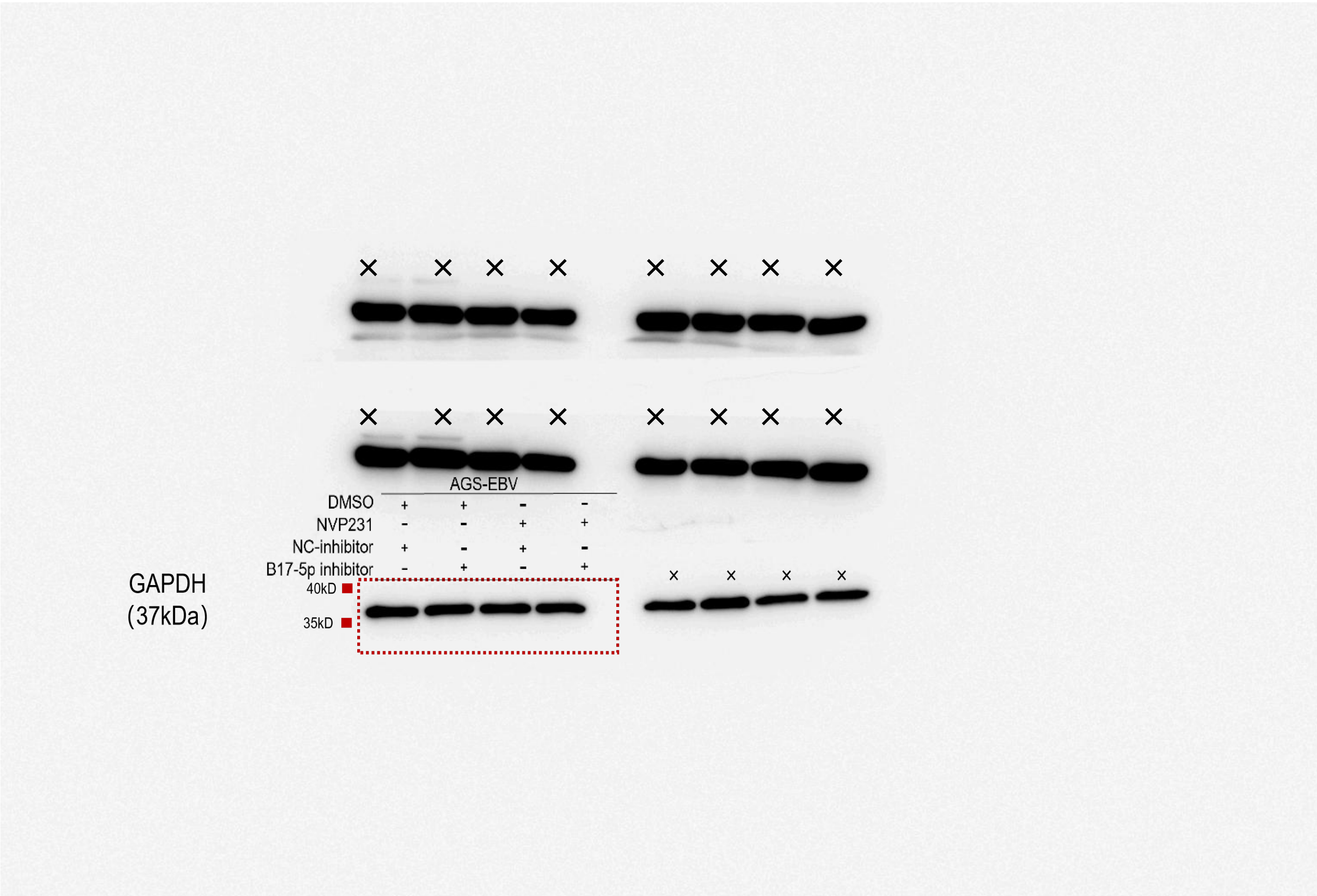

These images correspond to Figure 7A

Biological Replicate #2

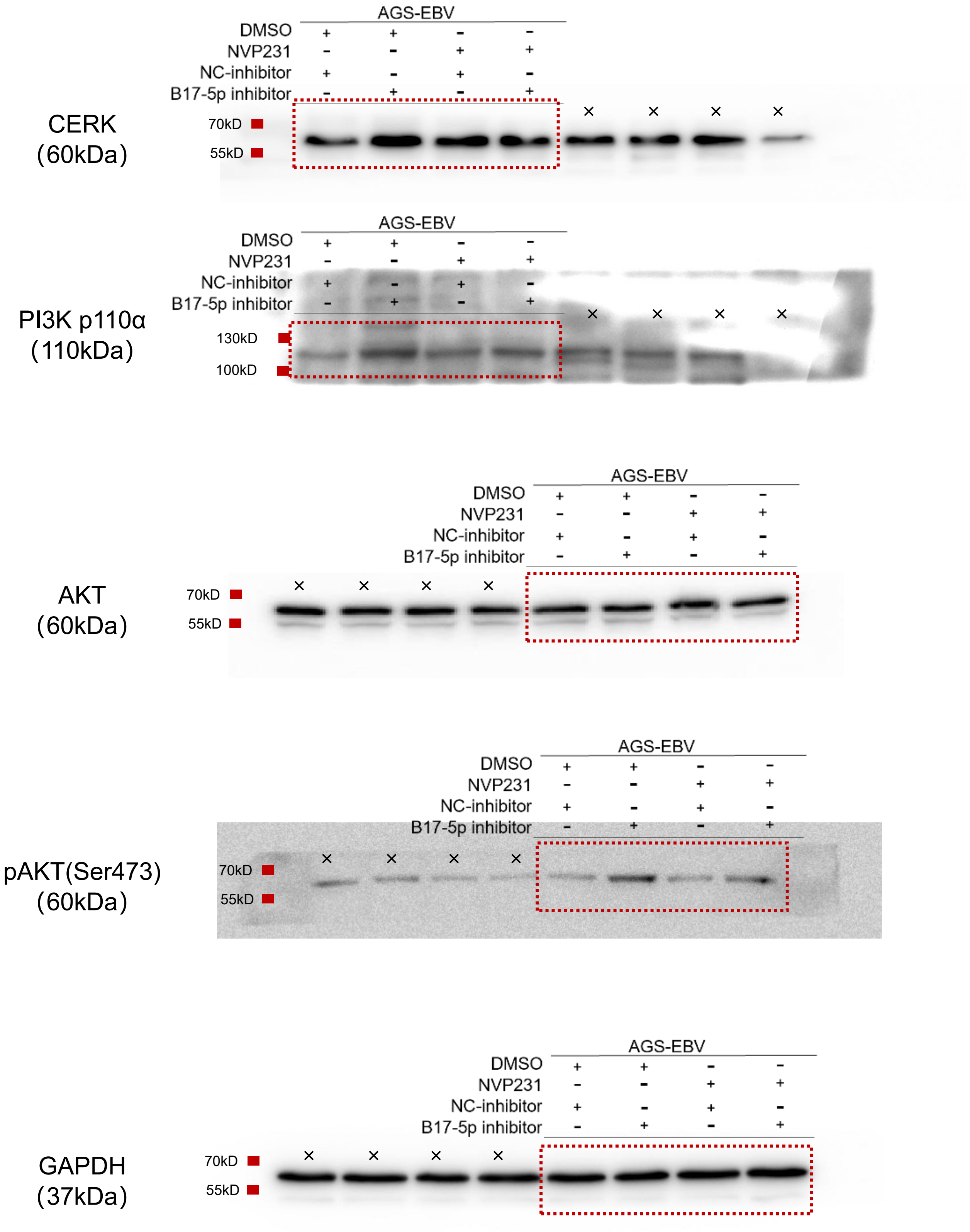

This image corresponds to Figure 7A

Biological Replicate #2

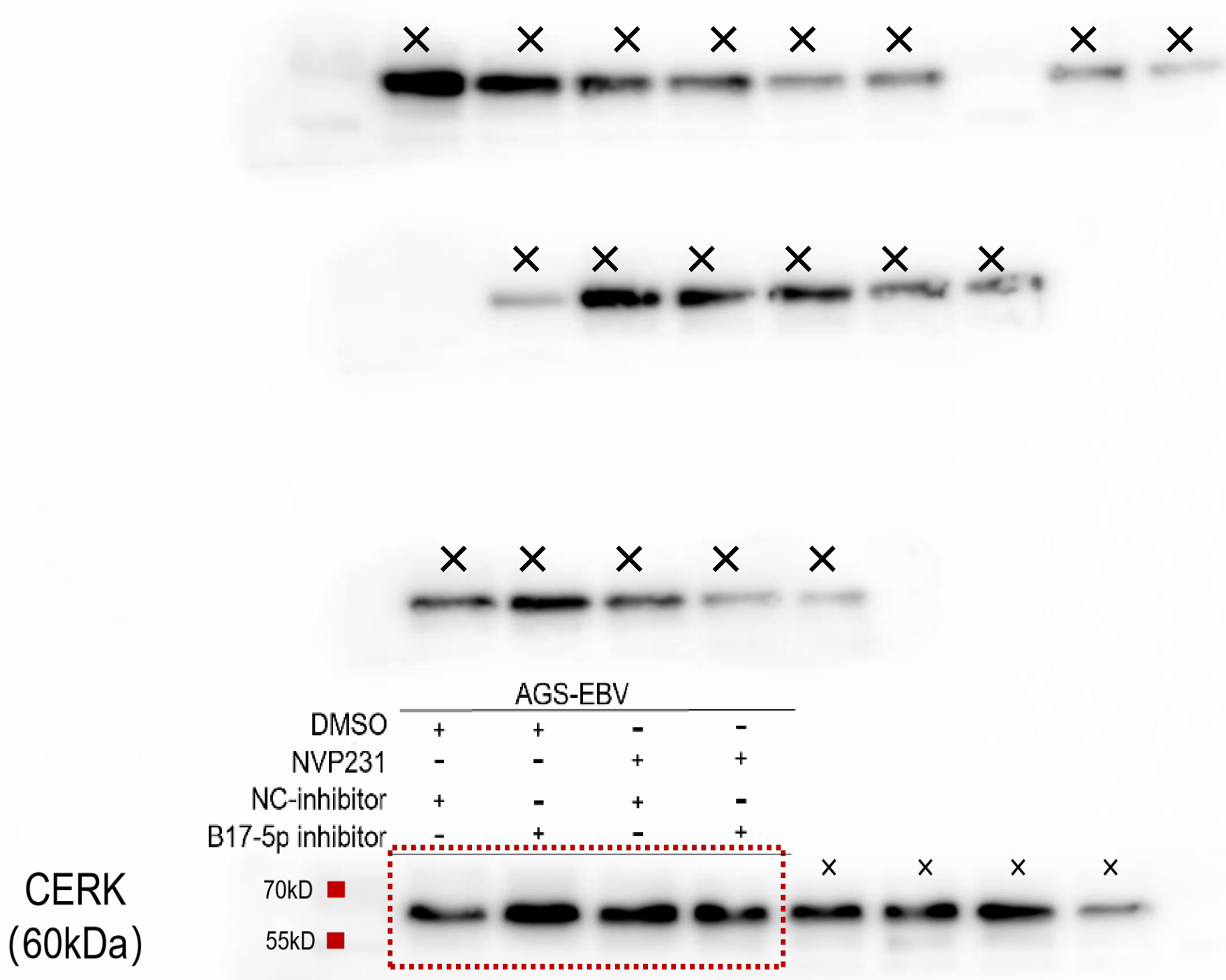

This image corresponds to Figure 7A

Biological Replicate #2

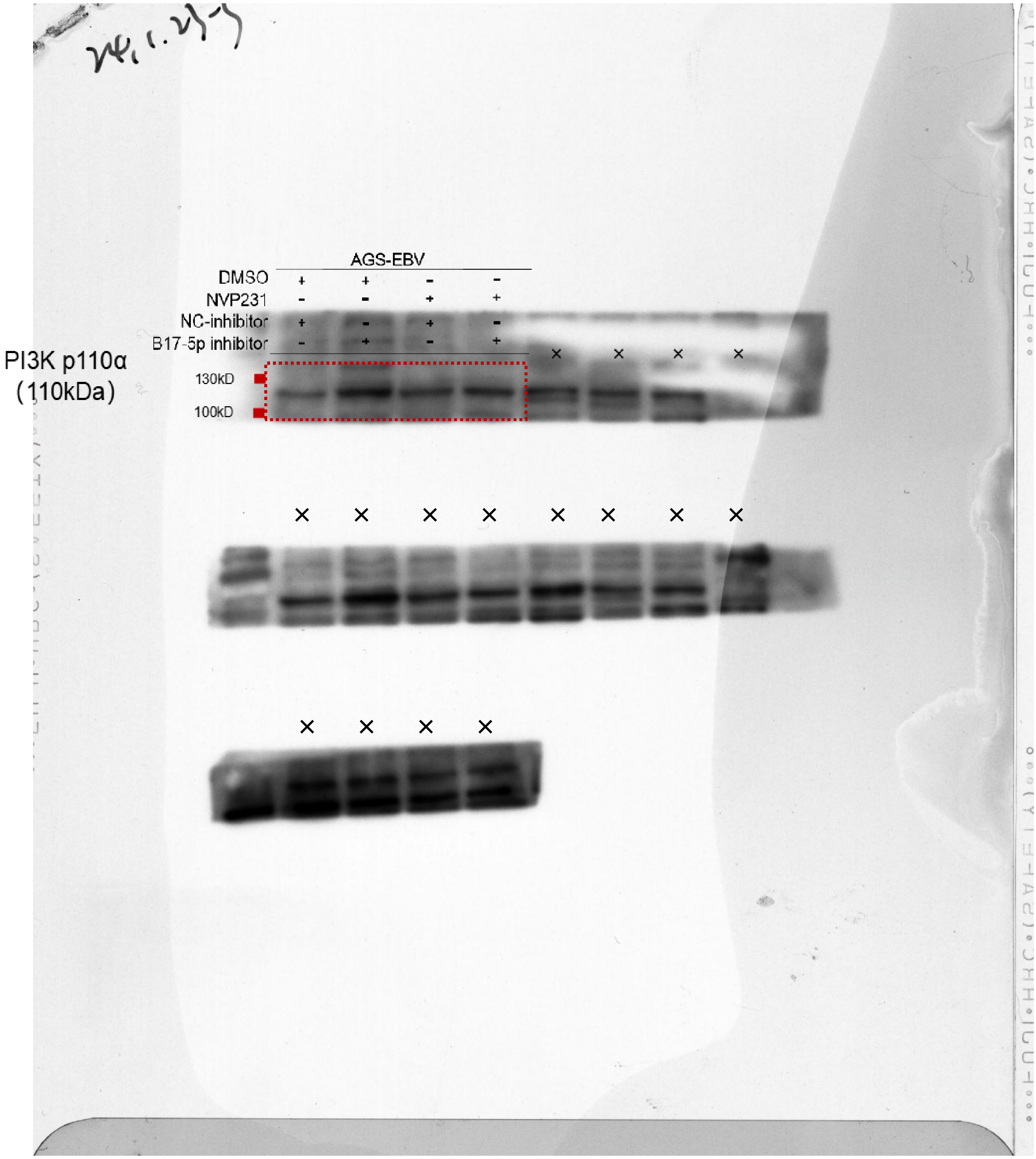

This image corresponds to Figure 7A

Biological Replicate #2

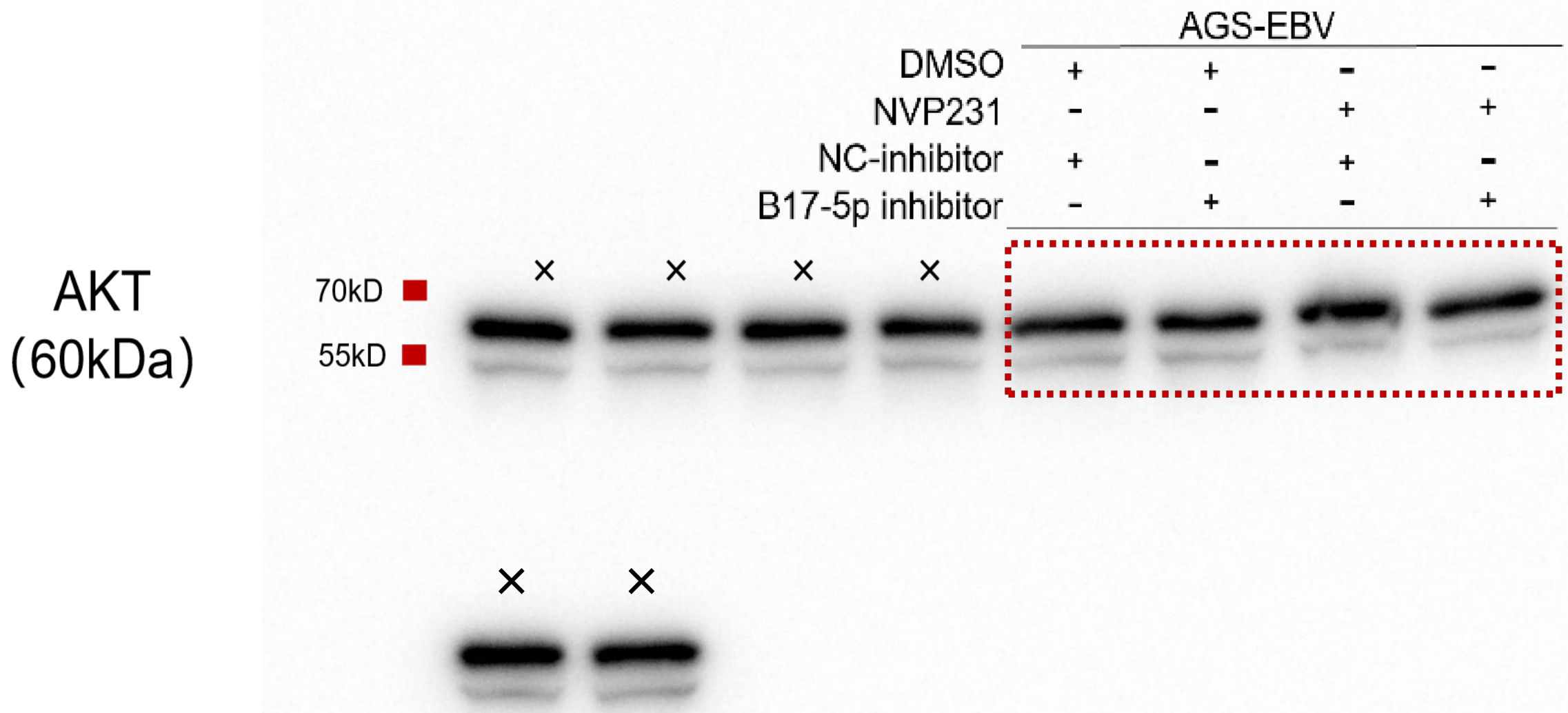

This image corresponds to Figure 7A

Biological Replicate #2

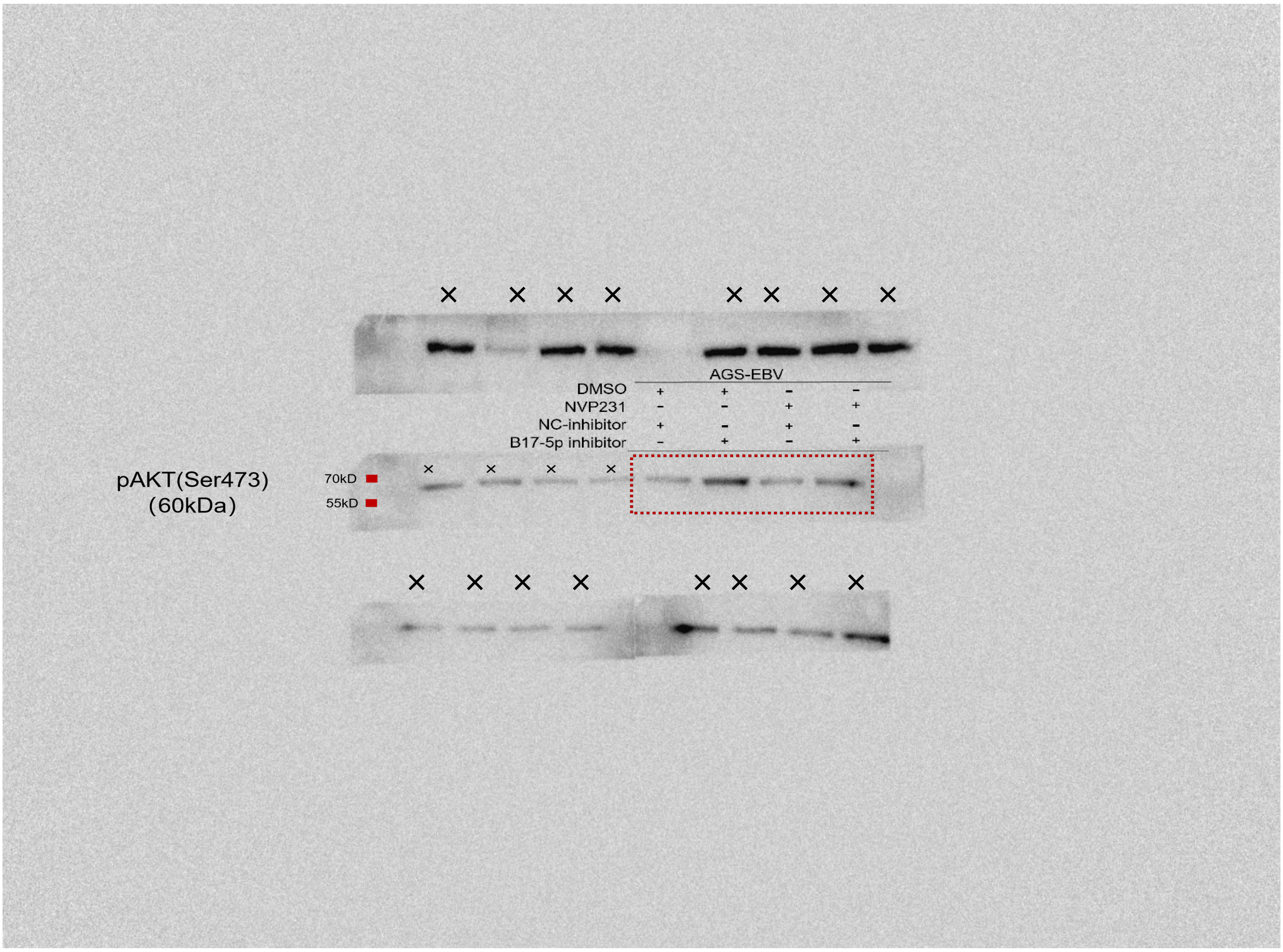

This image corresponds to Figure 7A

Biological Replicate #2

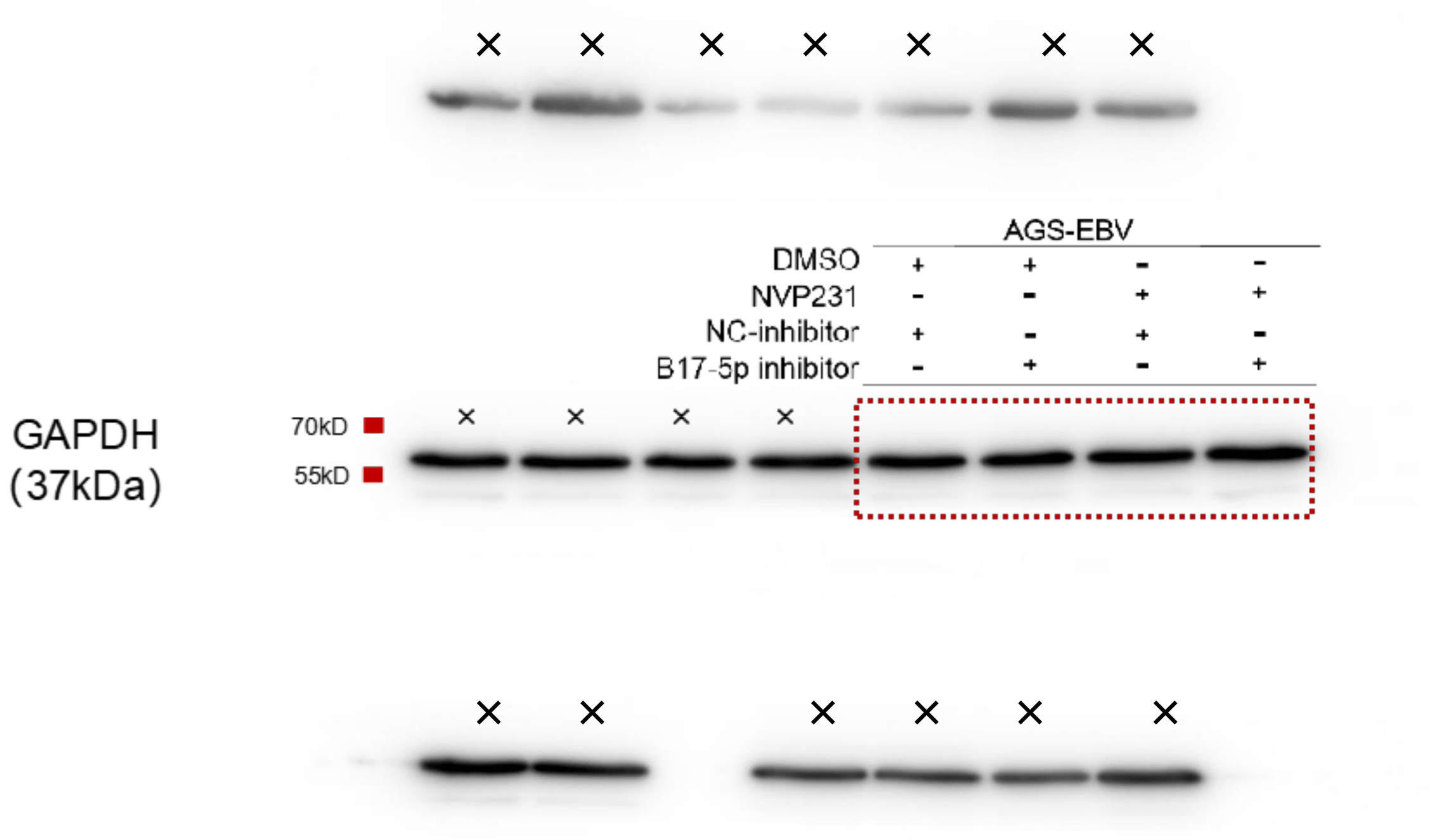

These images correspond to Figure 7A

Biological Replicate #3

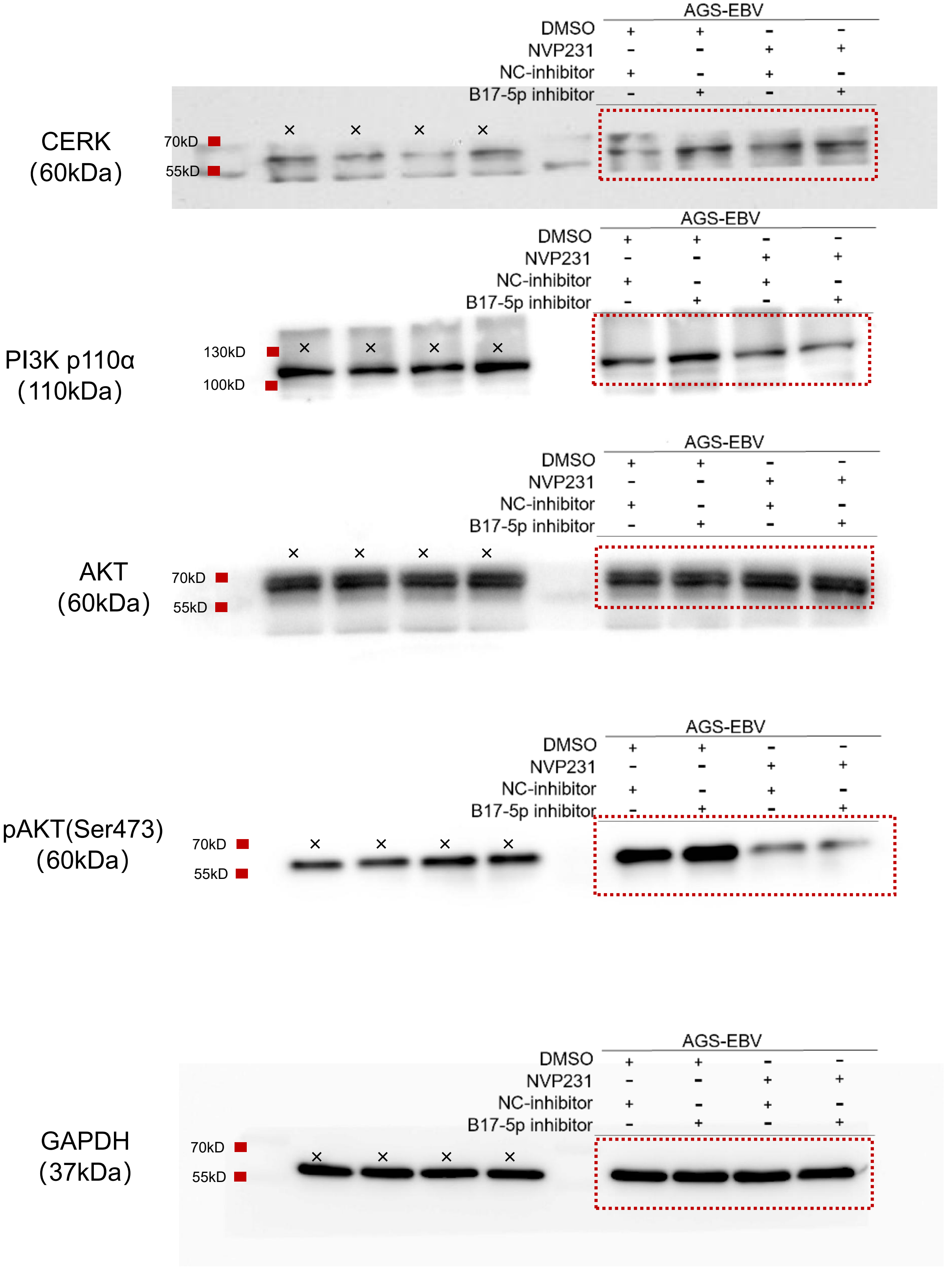

This image corresponds to Figure 7A

Biological Replicate #3

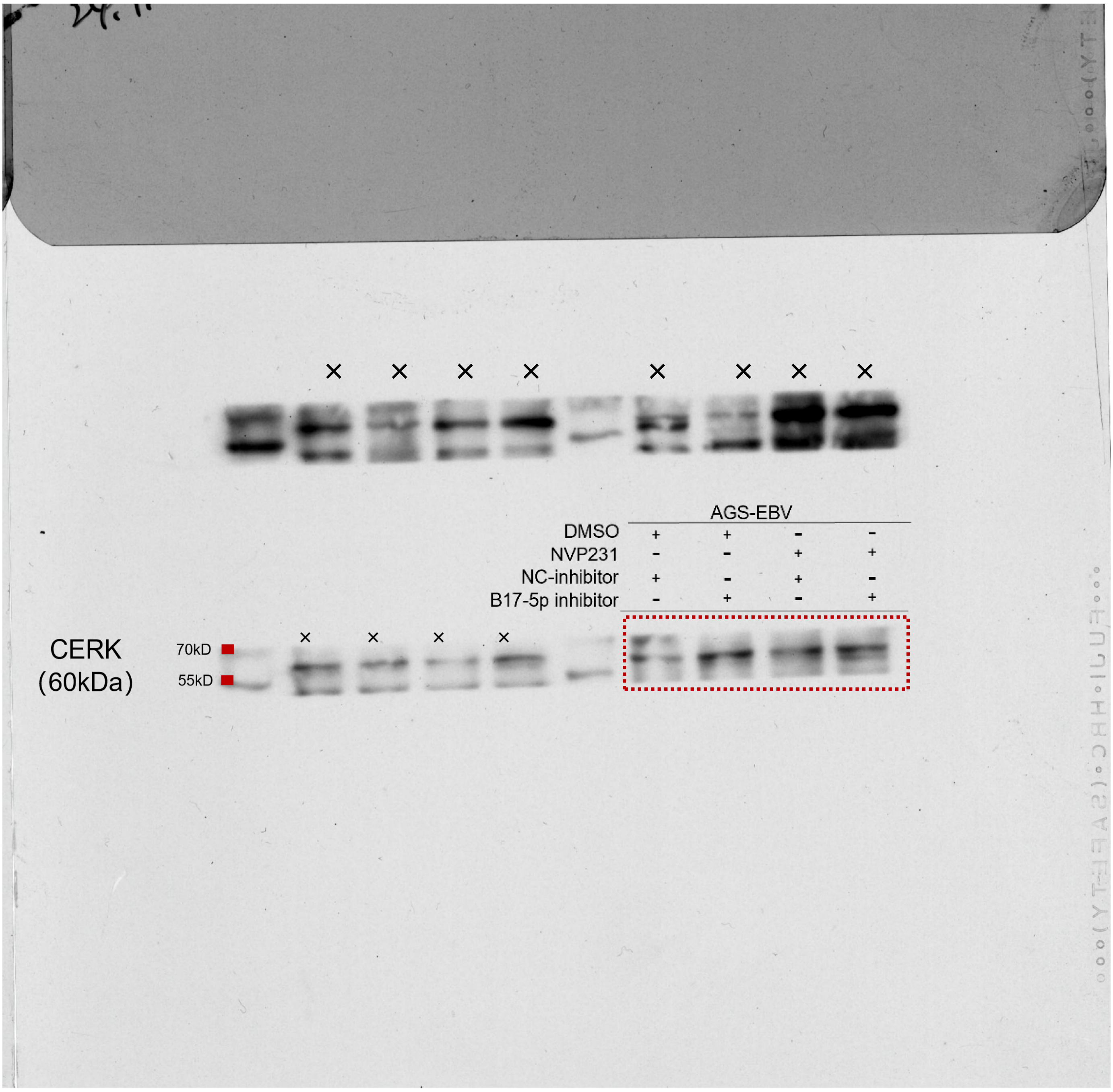

This image corresponds to Figure 7A

Biological Replicate #3

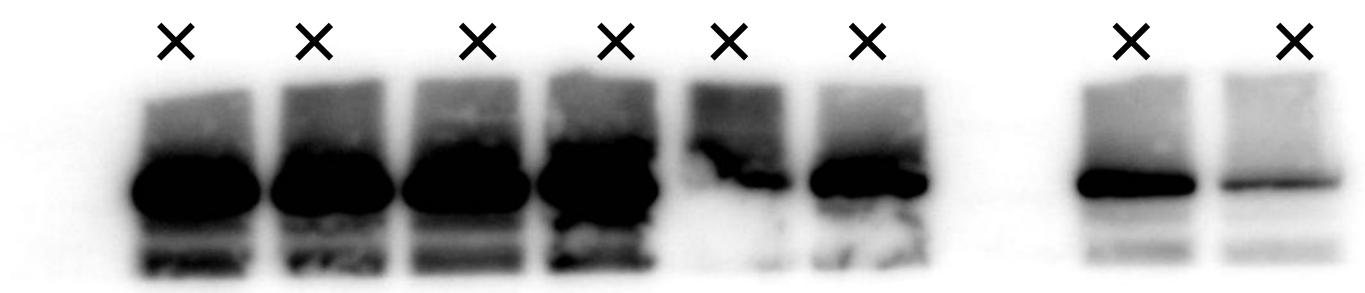

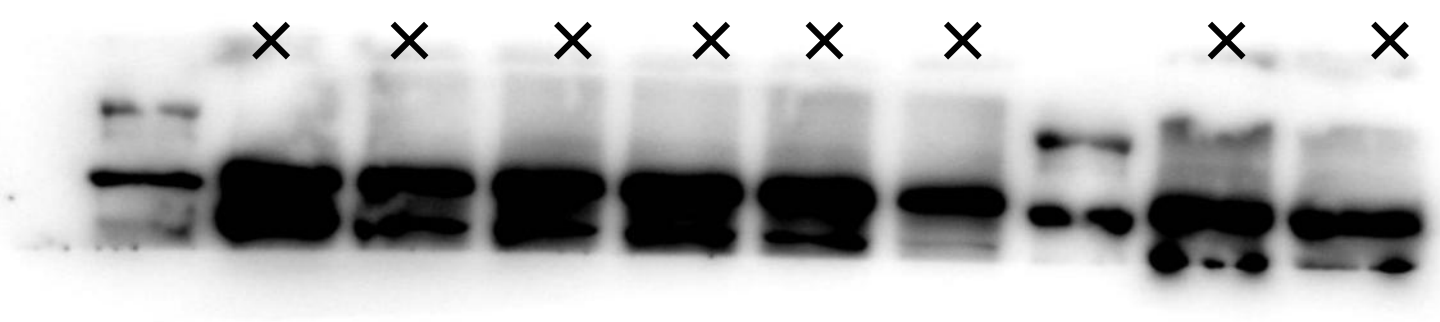

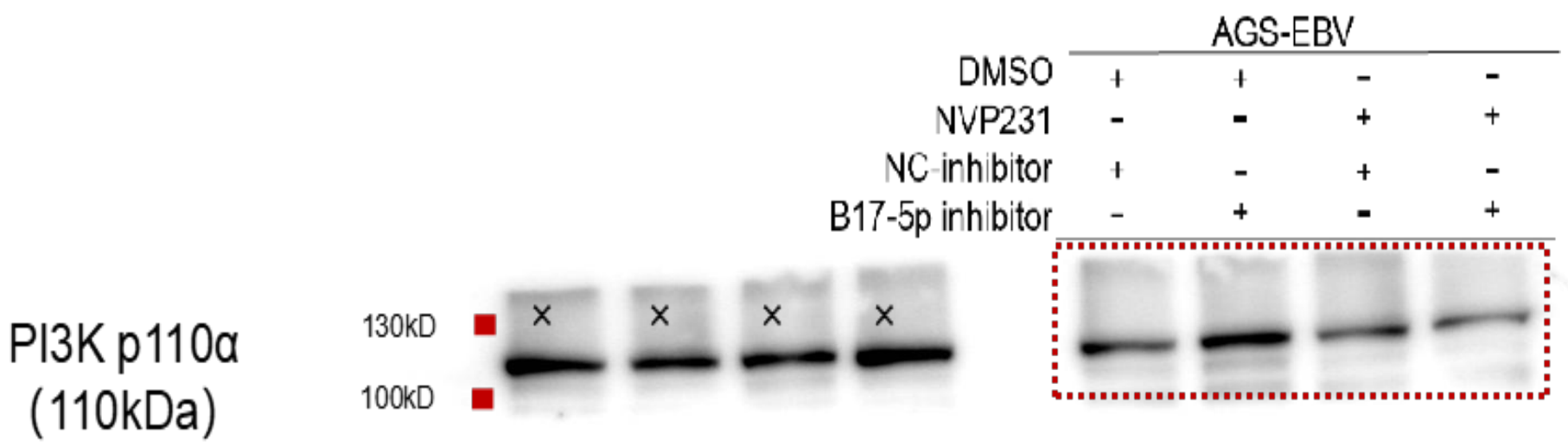

This image corresponds to Figure 7A

Biological Replicate #3

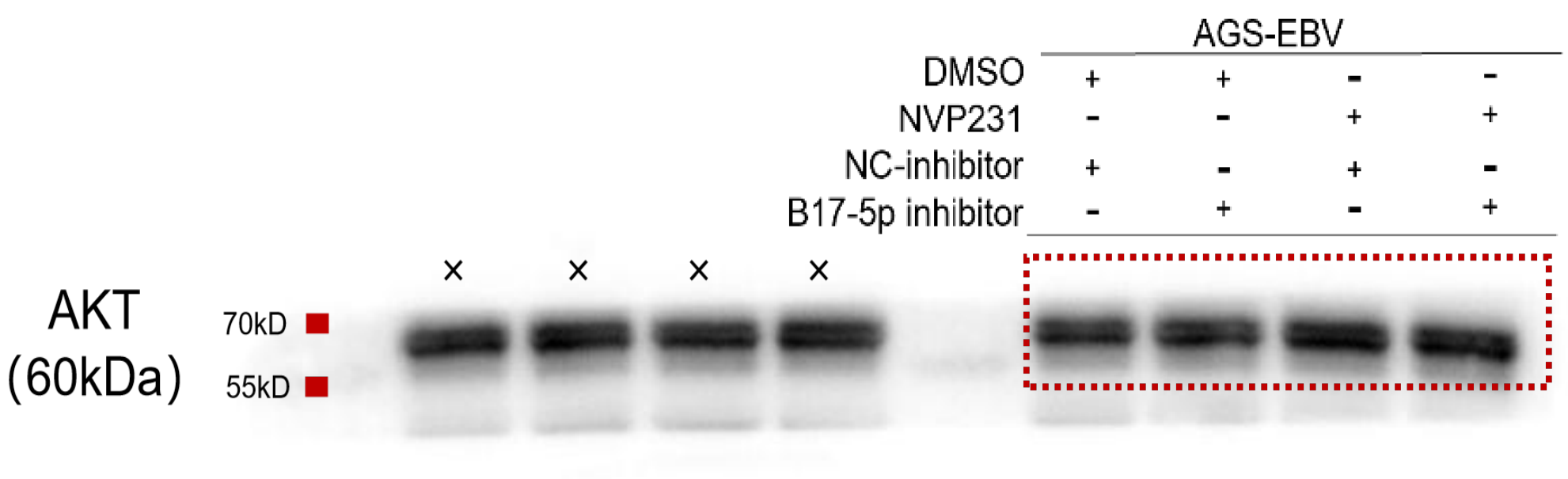

This image corresponds to Figure 7A

Biological Replicate #3

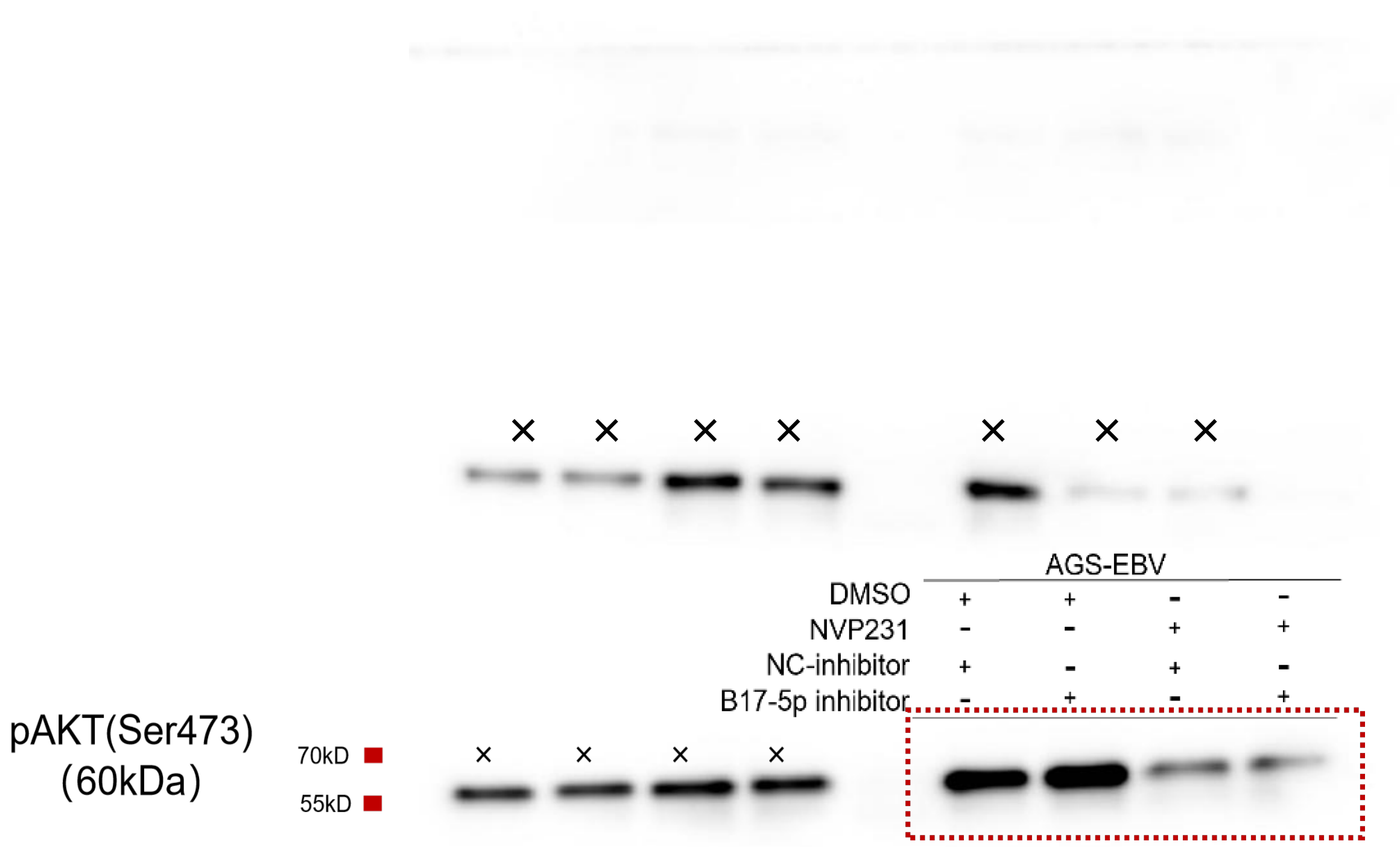

This image corresponds to Figure 7A

Biological Replicate #3

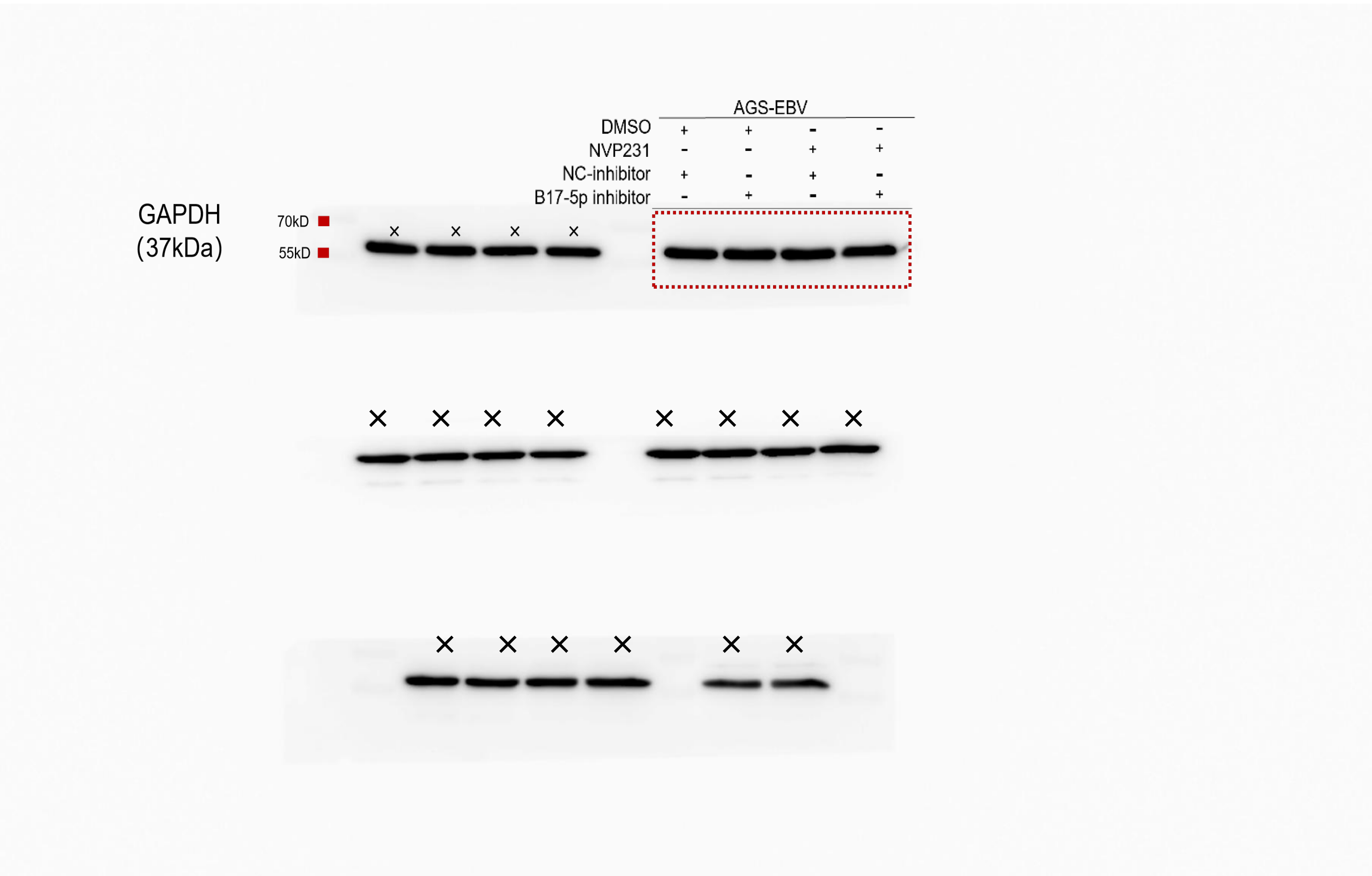

These images correspond to Figure 7C

Biological Replicate #1

▲ These images was selected as the representative result in Figure 6I.

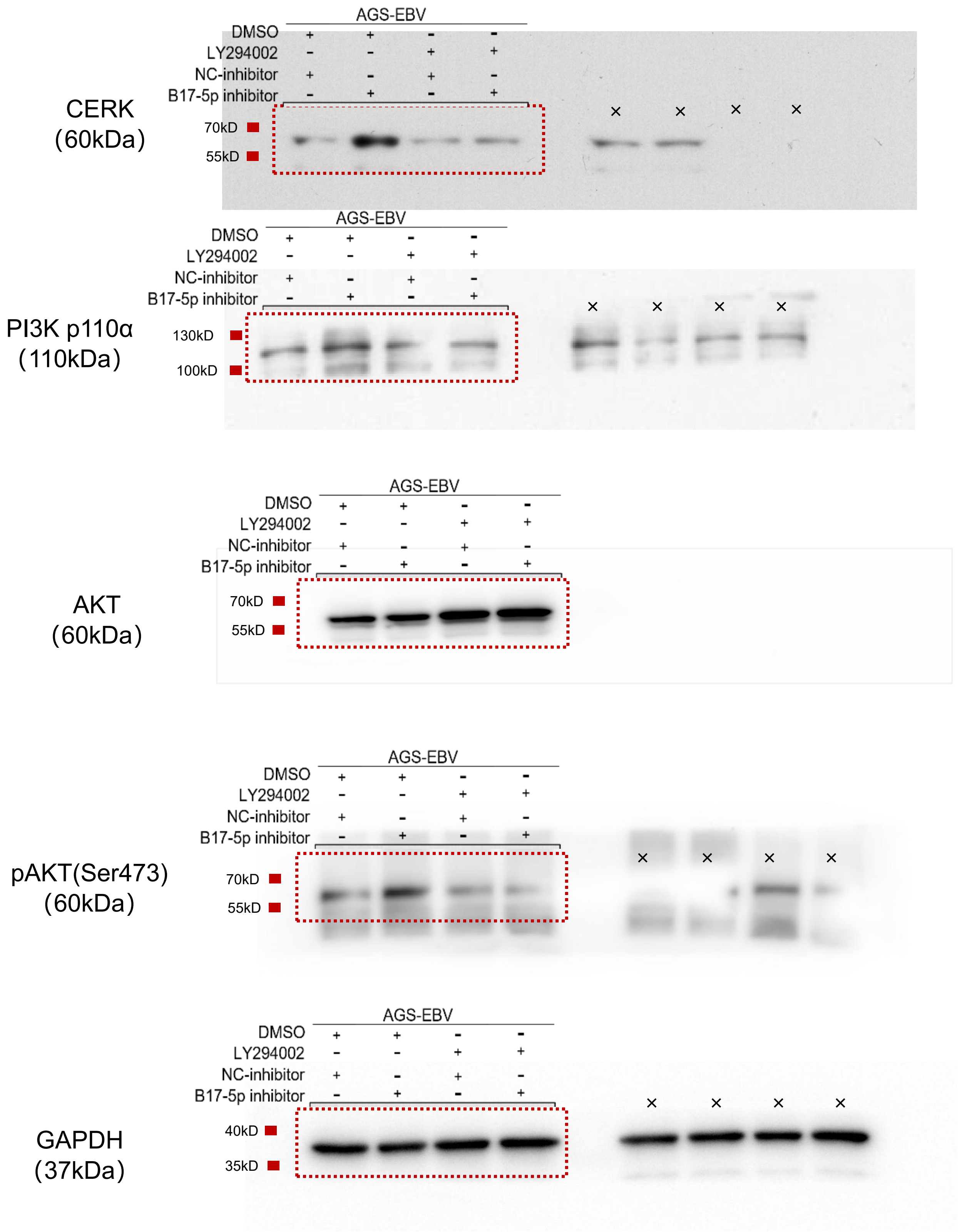

This image corresponds to Figure 7C

Biological Replicate #1

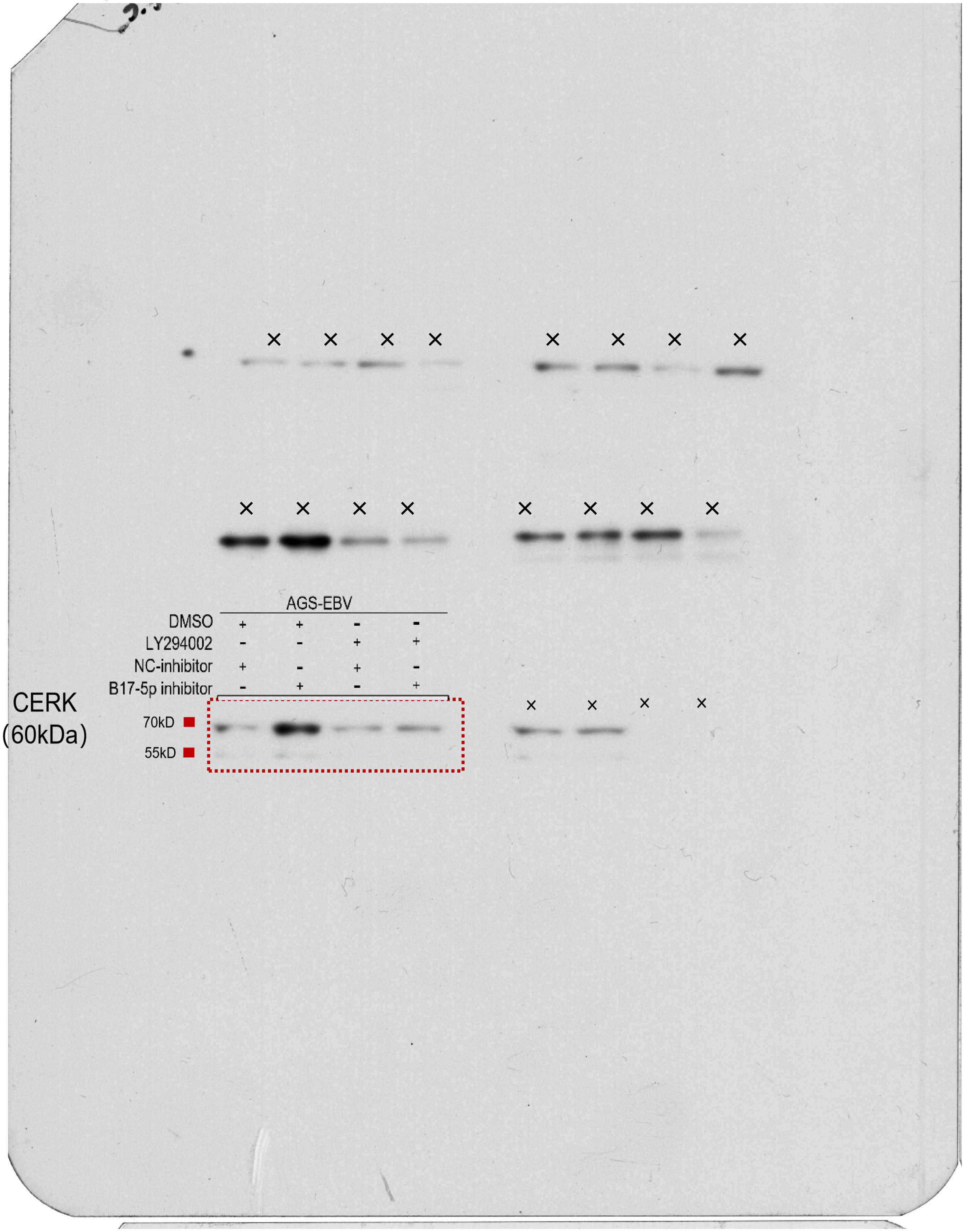

This image corresponds to Figure 7C

Biological Replicate #1

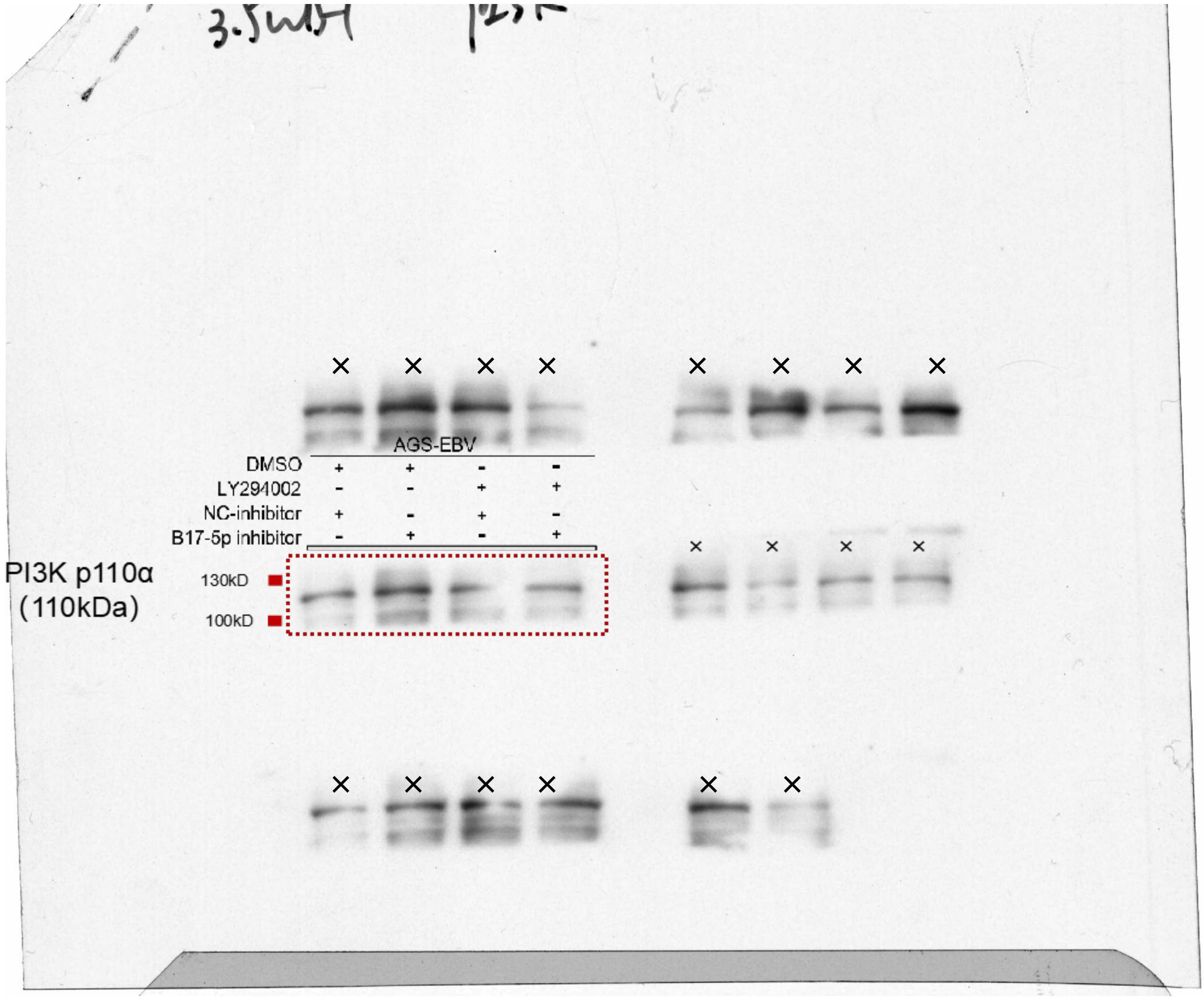

This image corresponds to Figure 7C

Biological Replicate #1

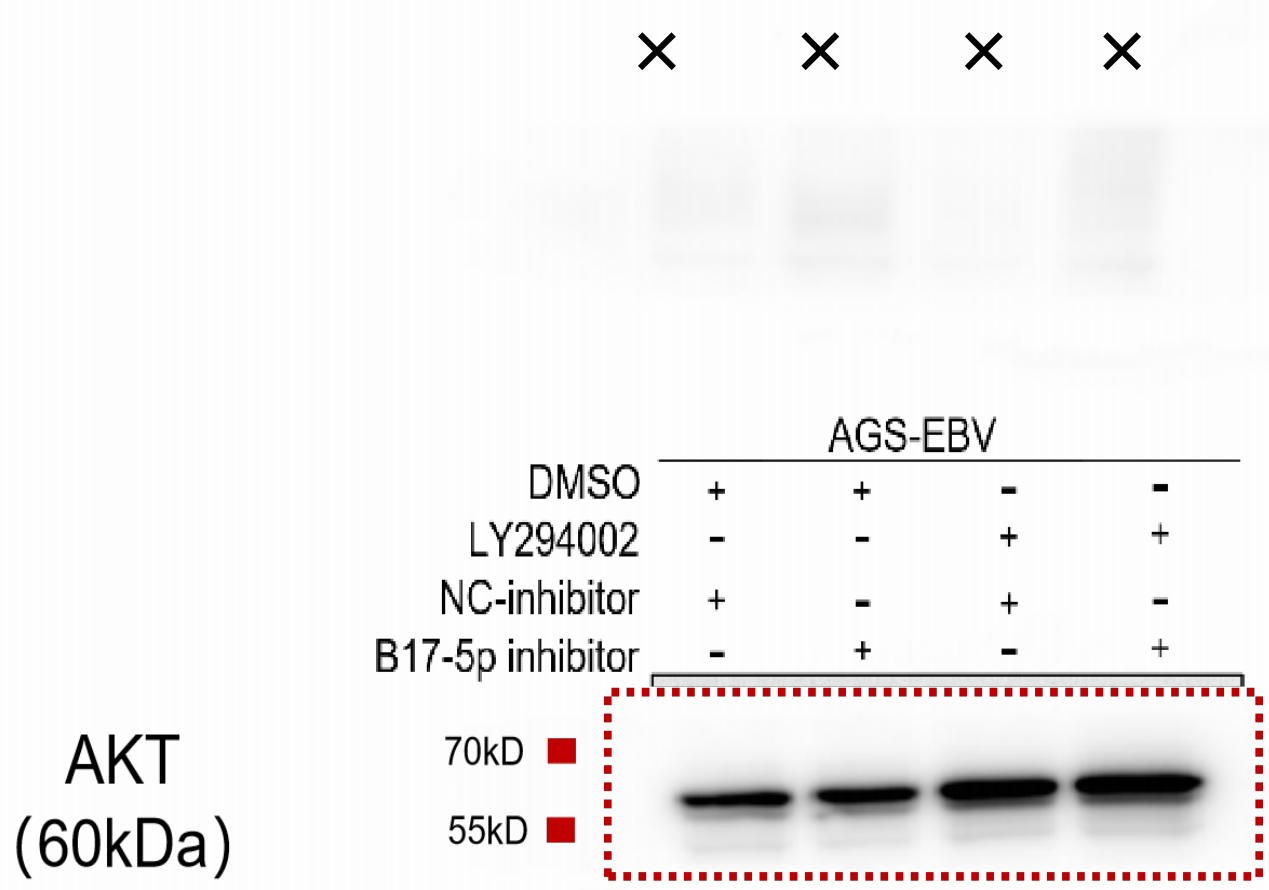

This image corresponds to Figure 7C

Biological Replicate #1

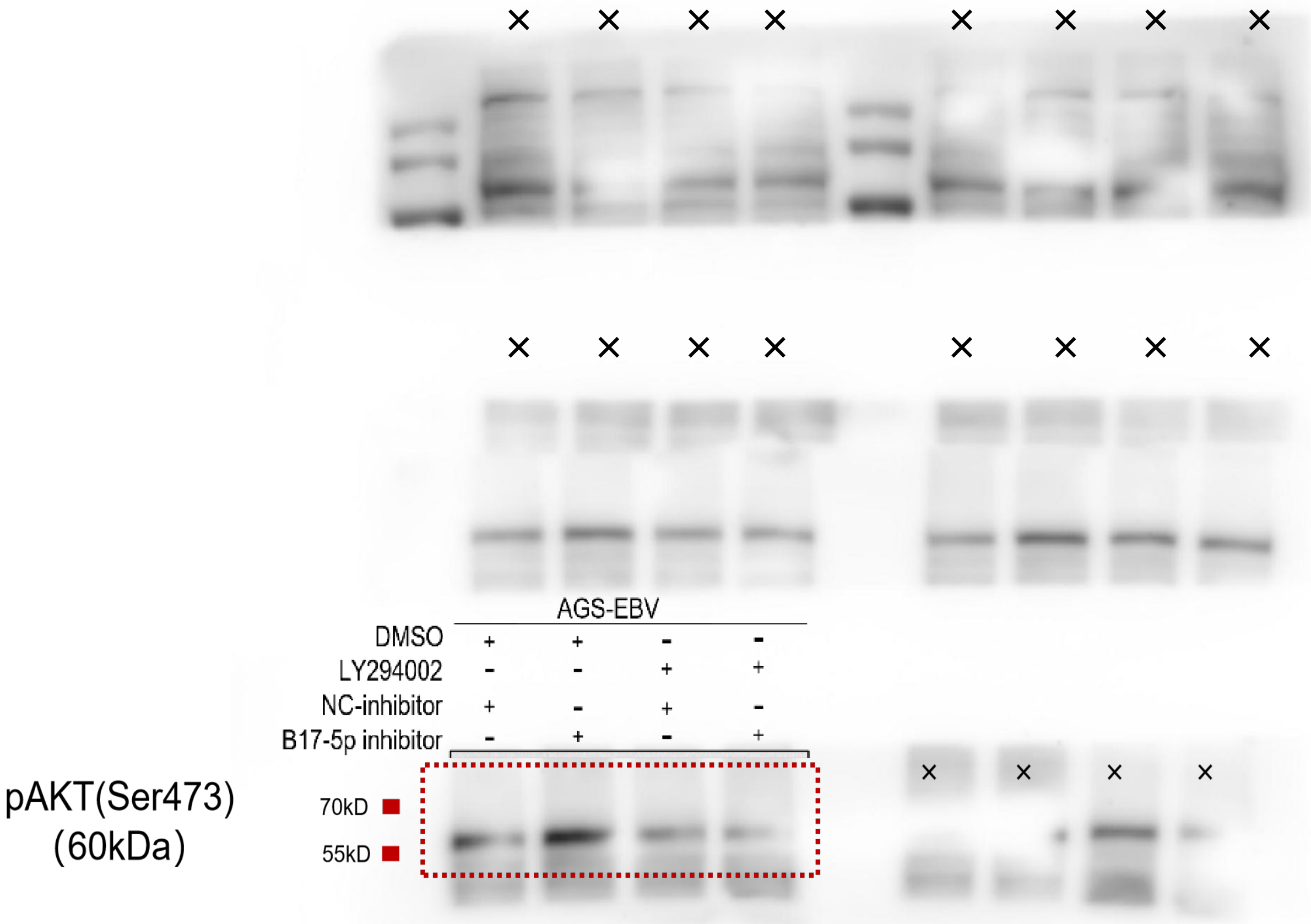

This image corresponds to Figure 7C

Biological Replicate #1

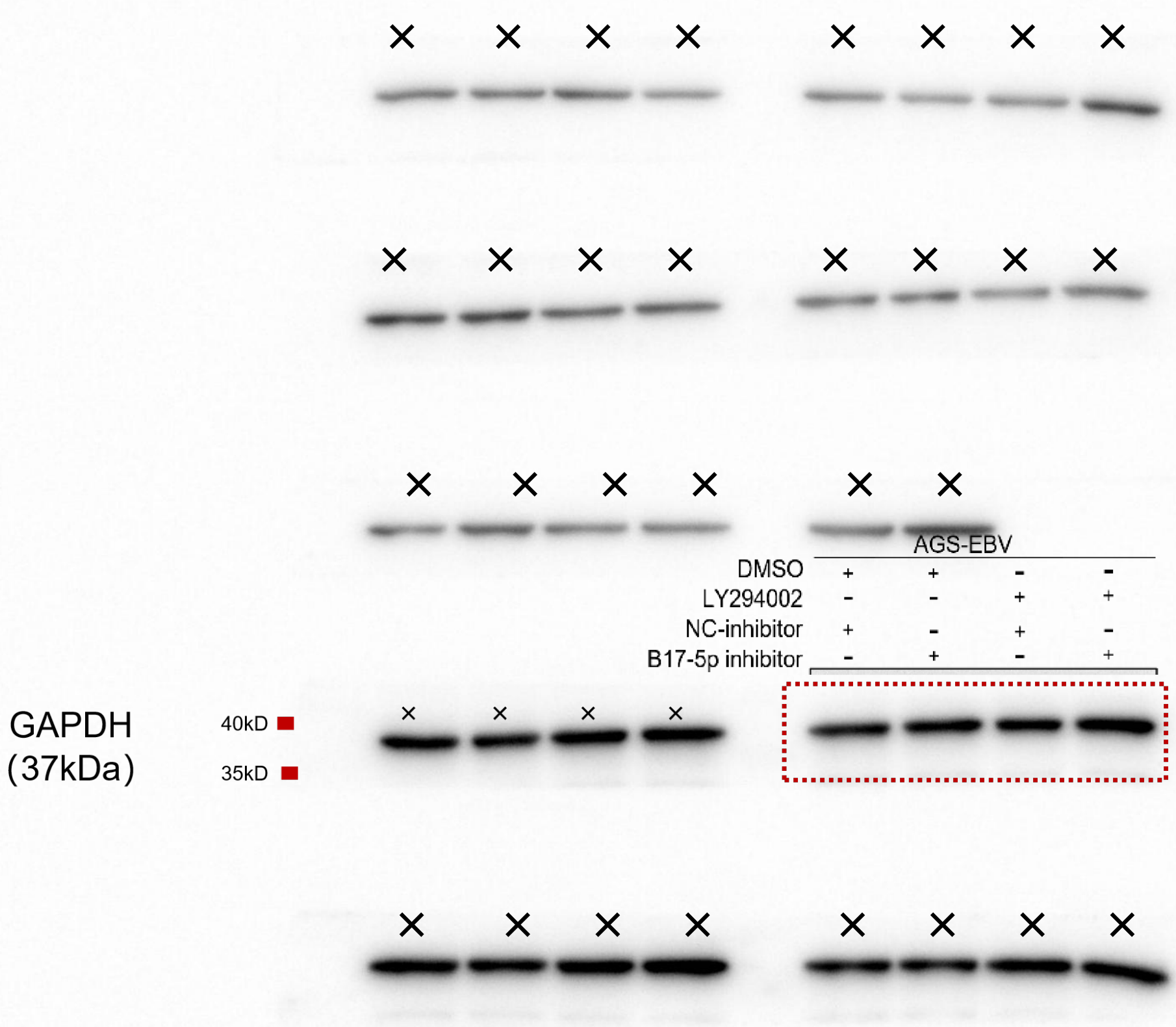

These images correspond to Figure 7C

Biological Replicate #2

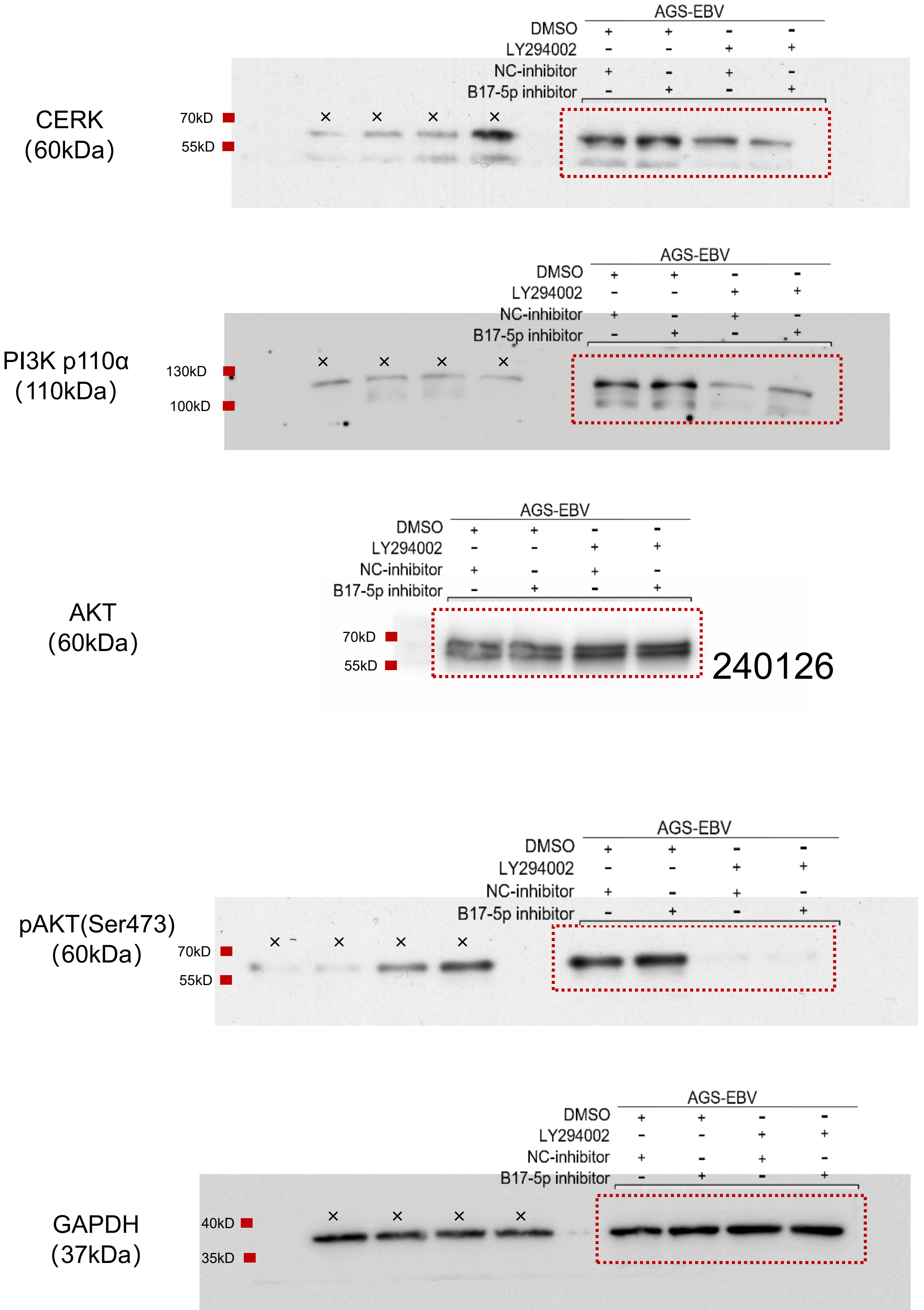

This image corresponds to Figure 7C

Biological Replicate #2

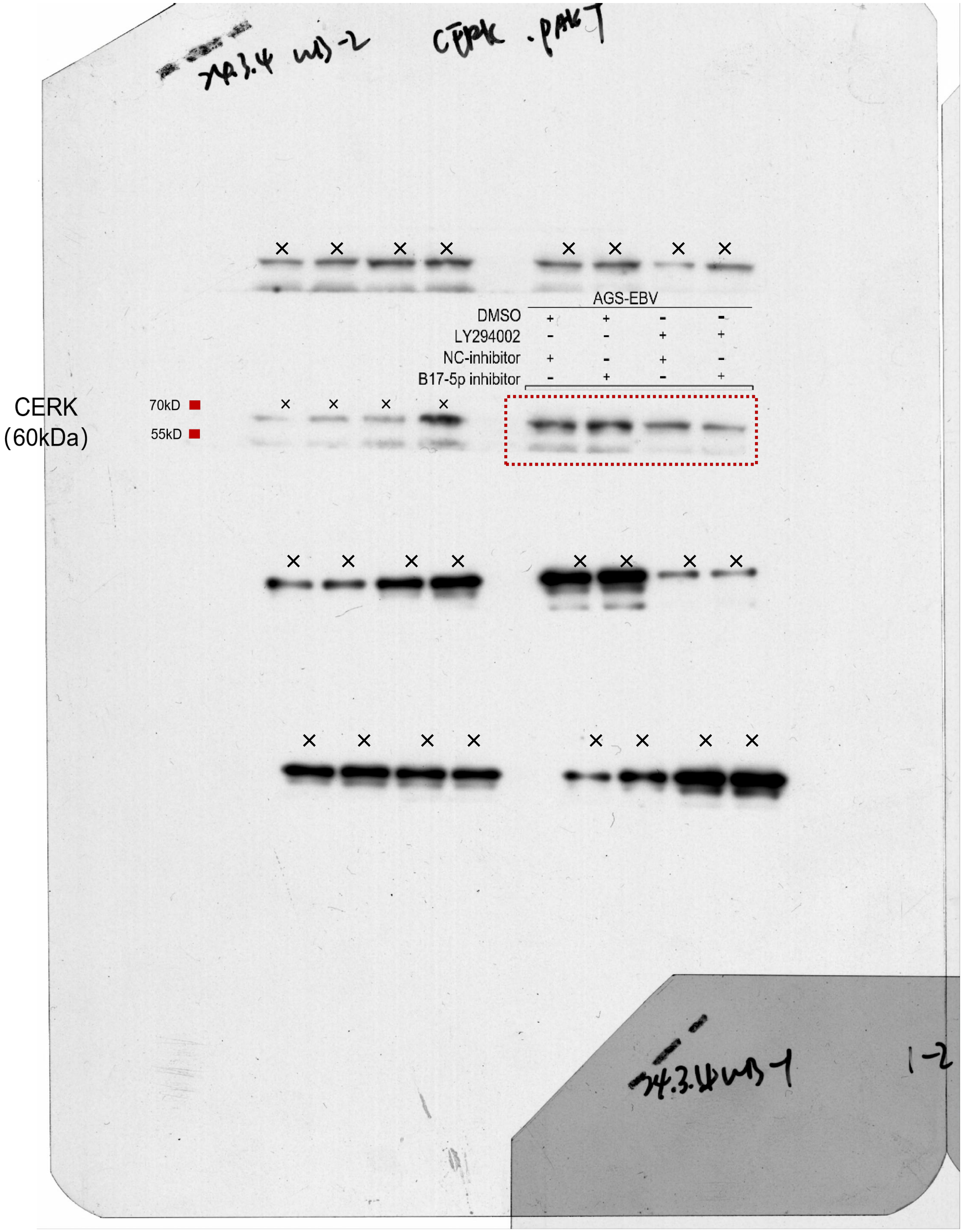

This image corresponds to Figure 7C

Biological Replicate #2

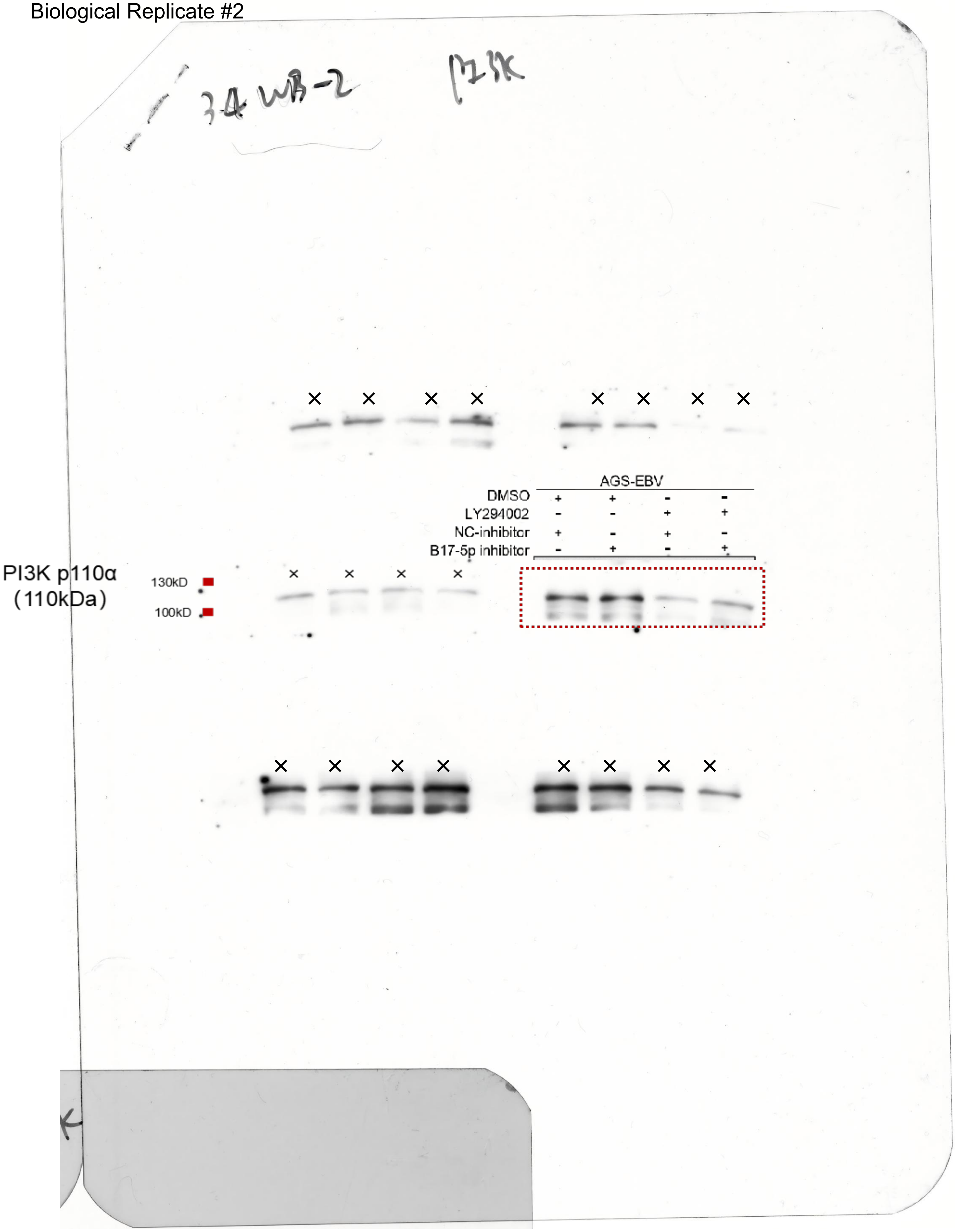

This image corresponds to Figure 7C

Biological Replicate #2

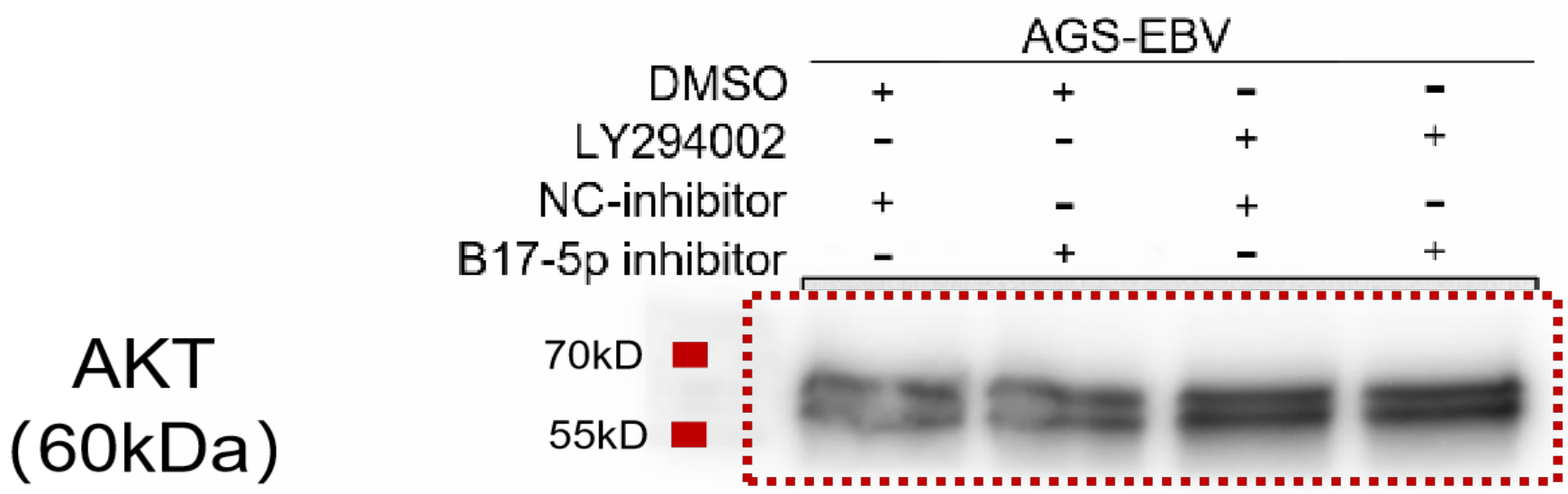

This image corresponds to Figure 7C

Biological Replicate #2

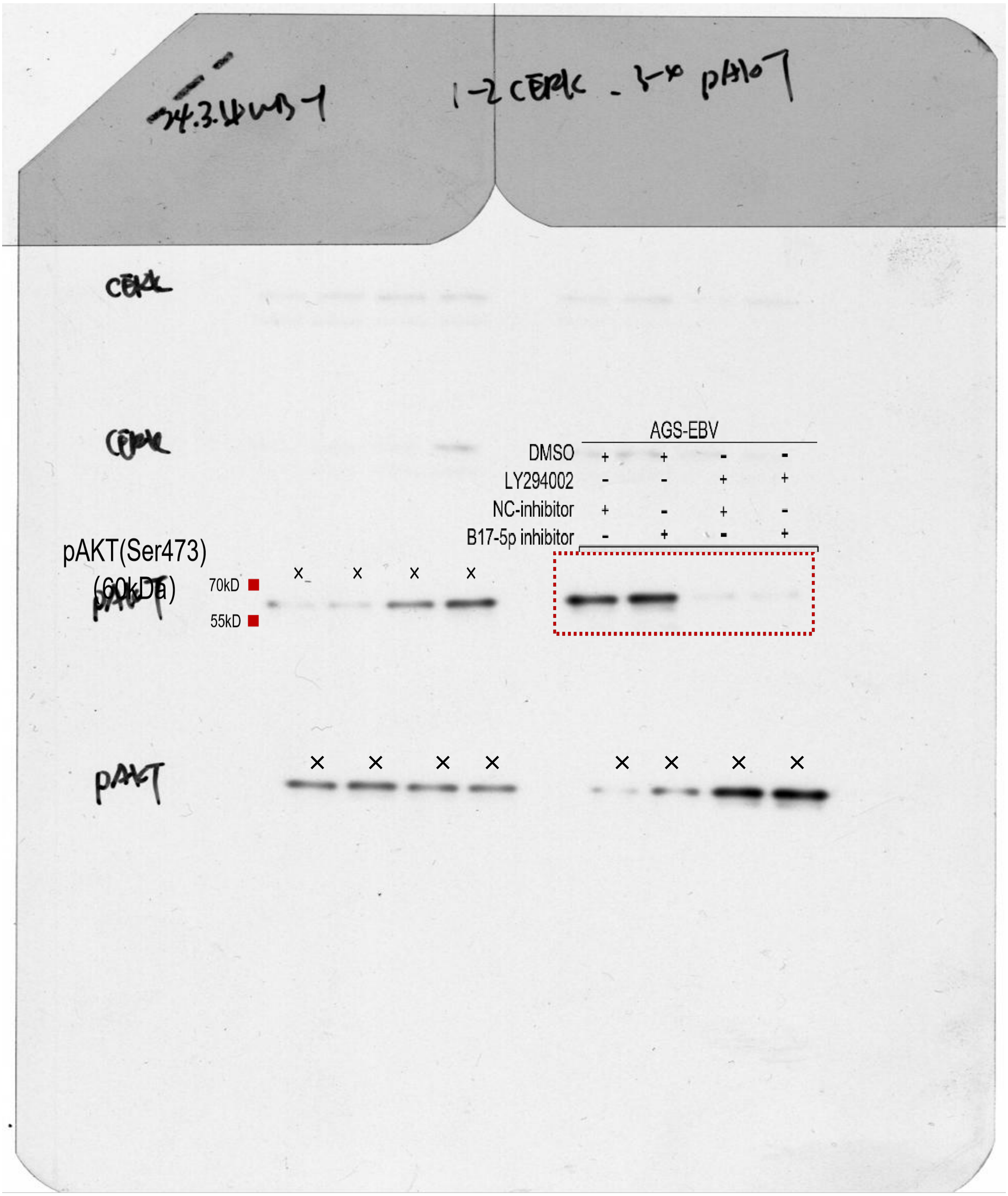

This image corresponds to Figure 7C

Biological Replicate #2

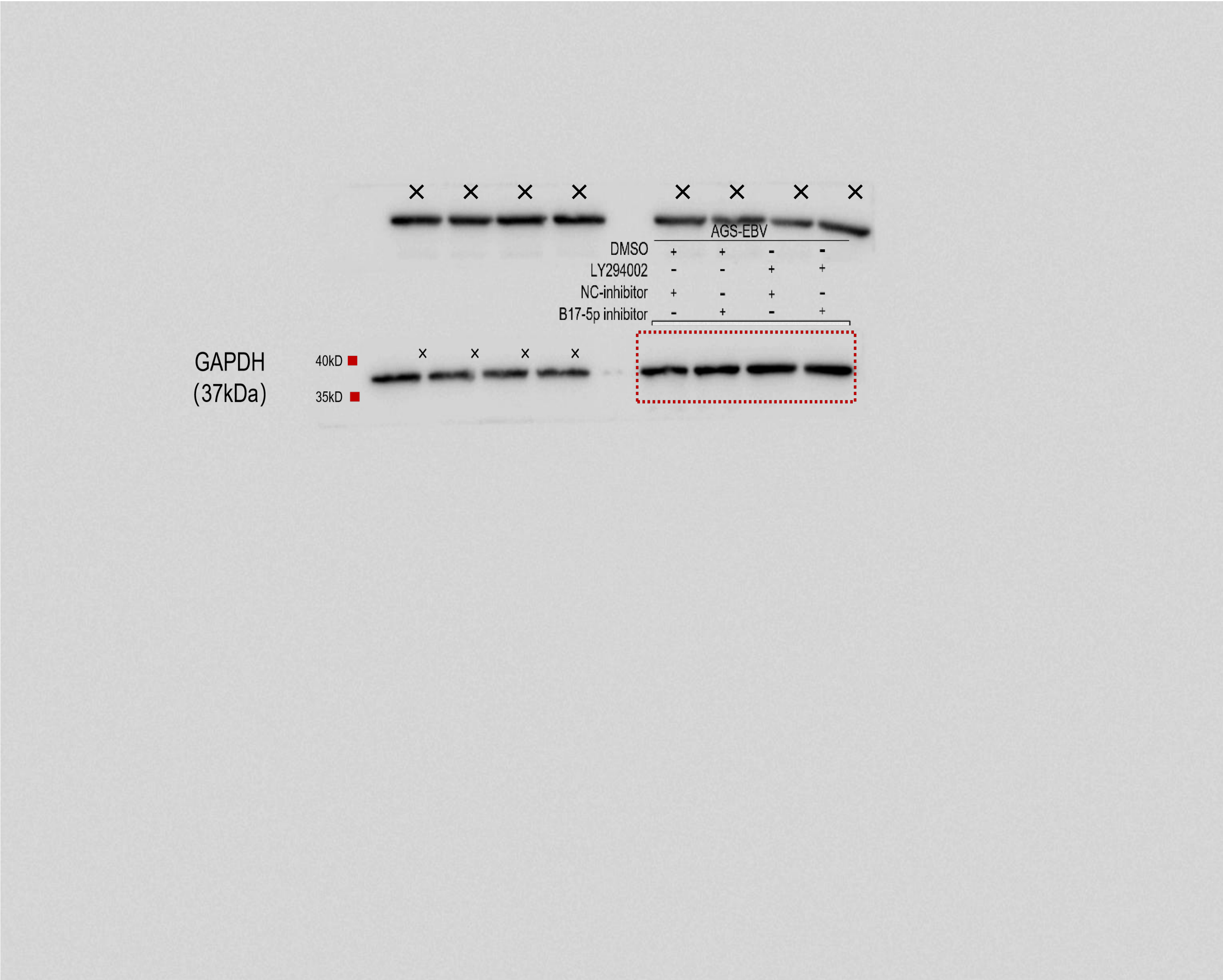

These images correspond to Figure 7C

Biological Replicate #3

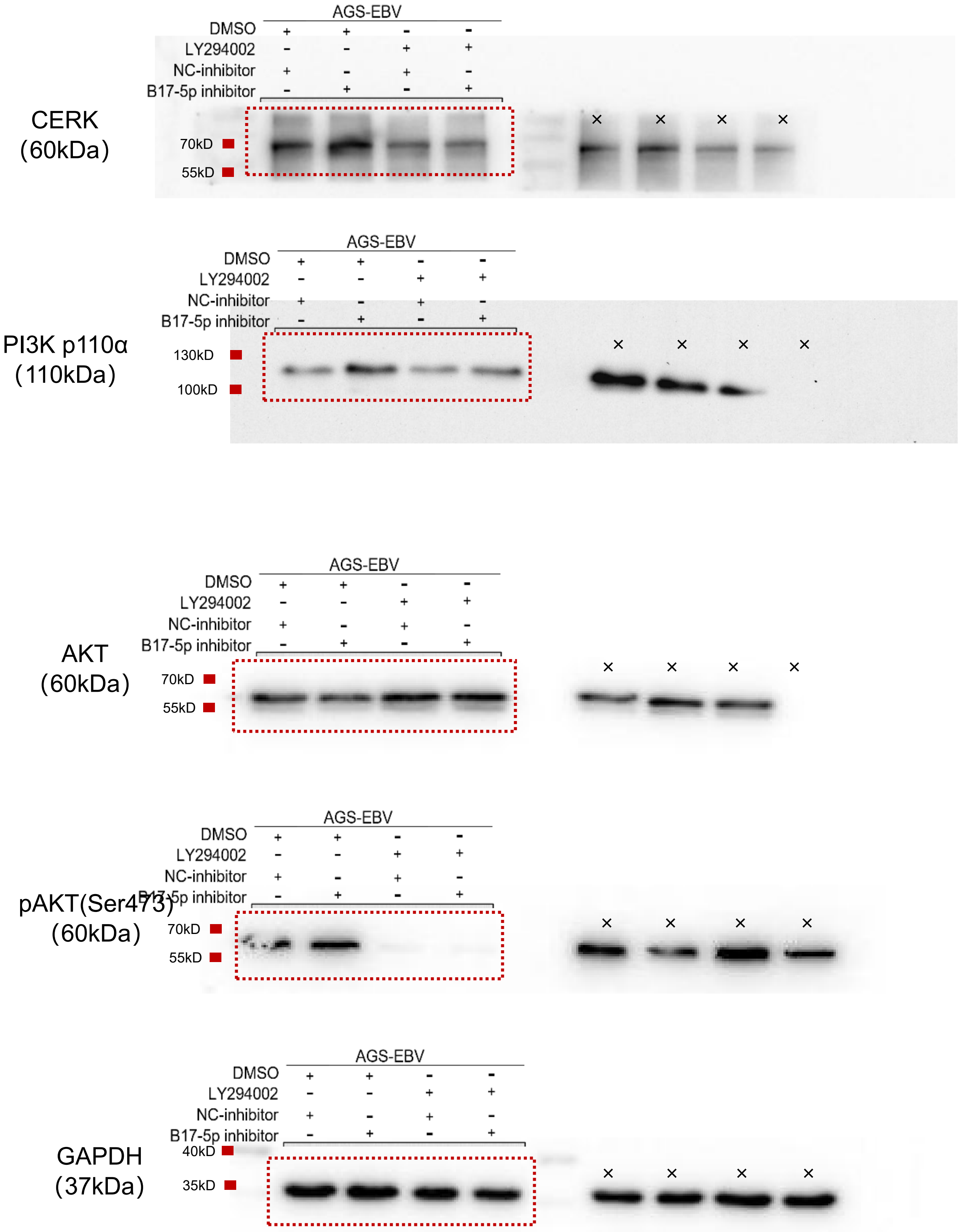

These images correspond to Figure 7C

Biological Replicate #3

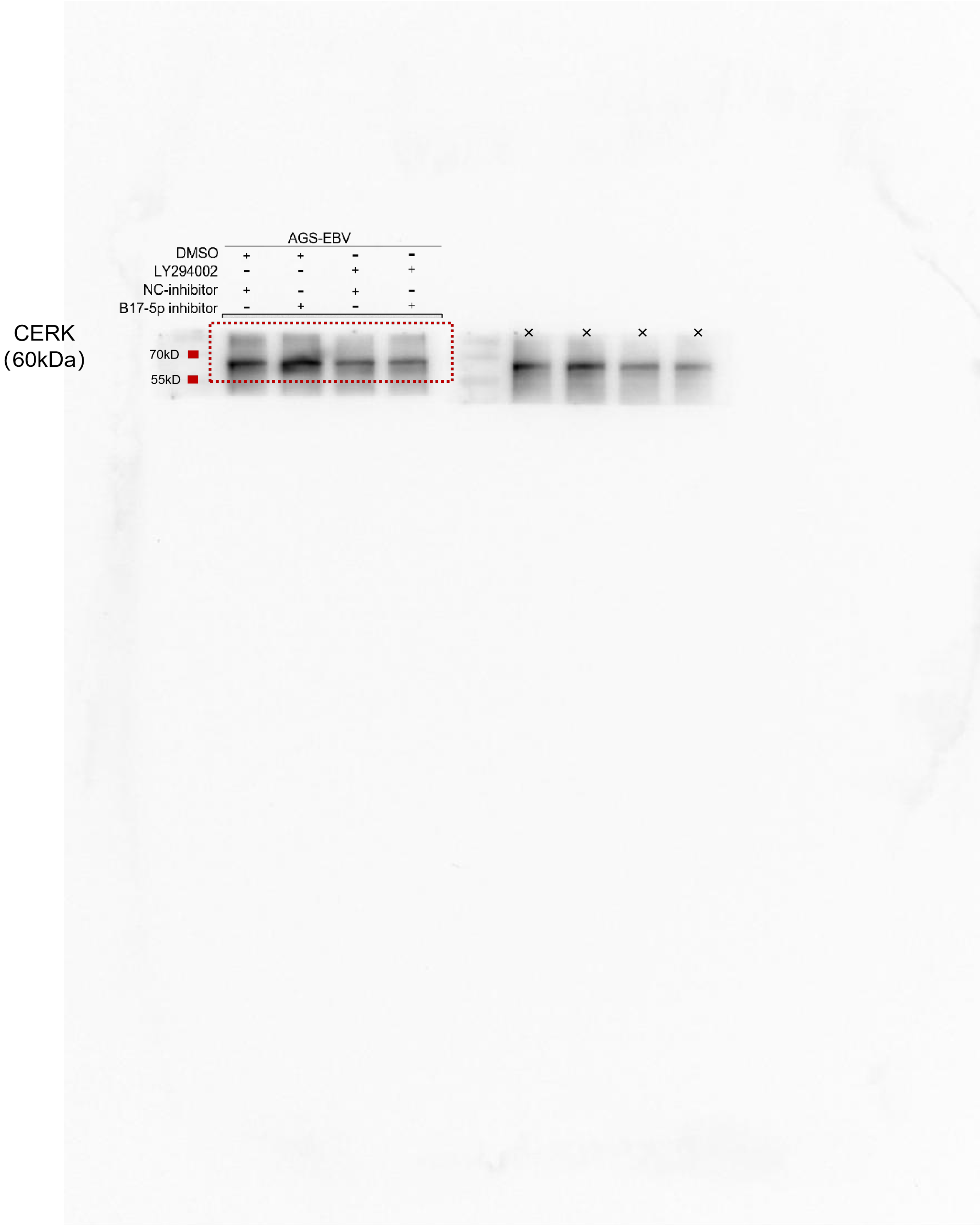

These images correspond to Figure 7C

Biological Replicate #3

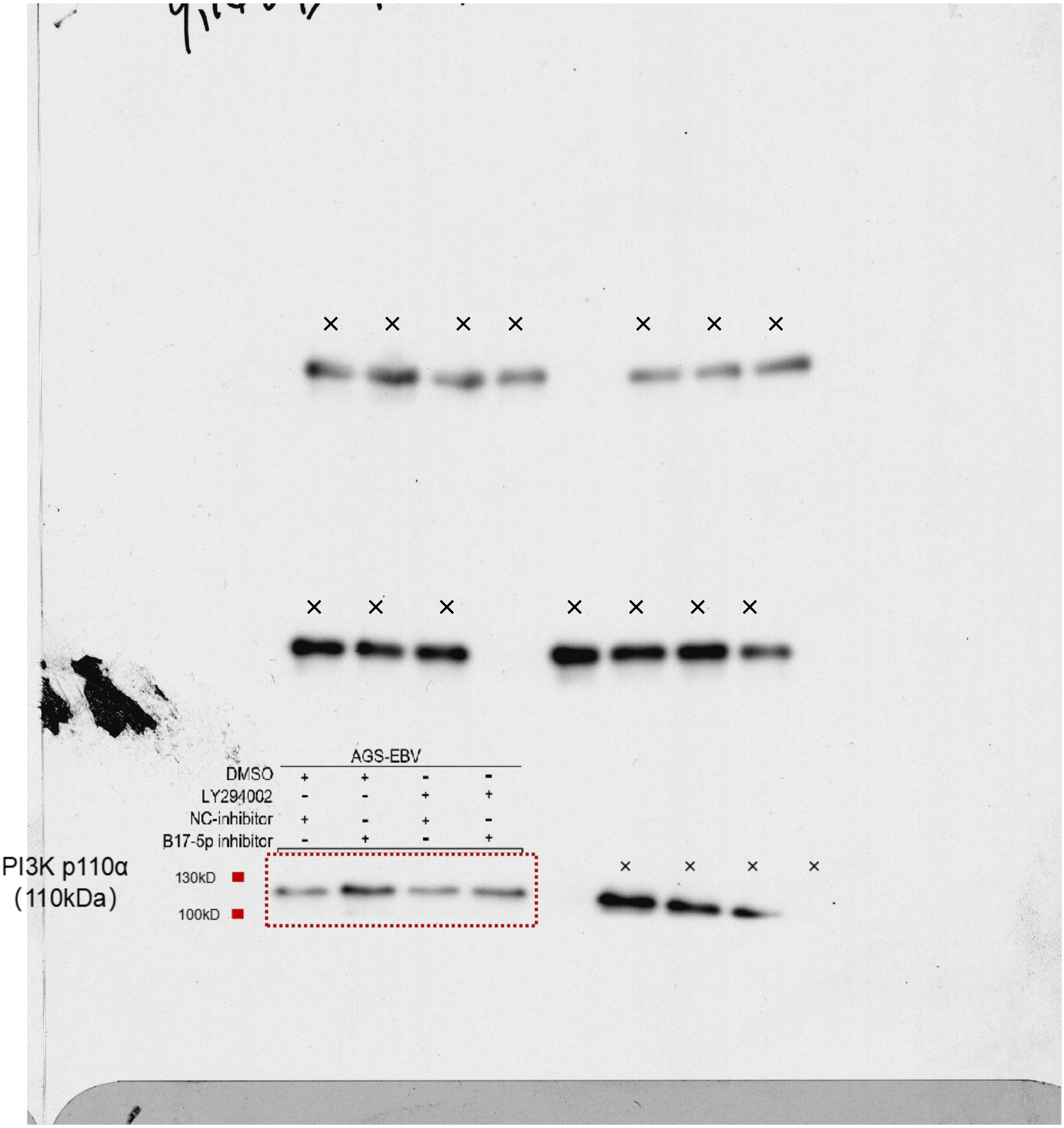

These images correspond to Figure 7C

Biological Replicate #3

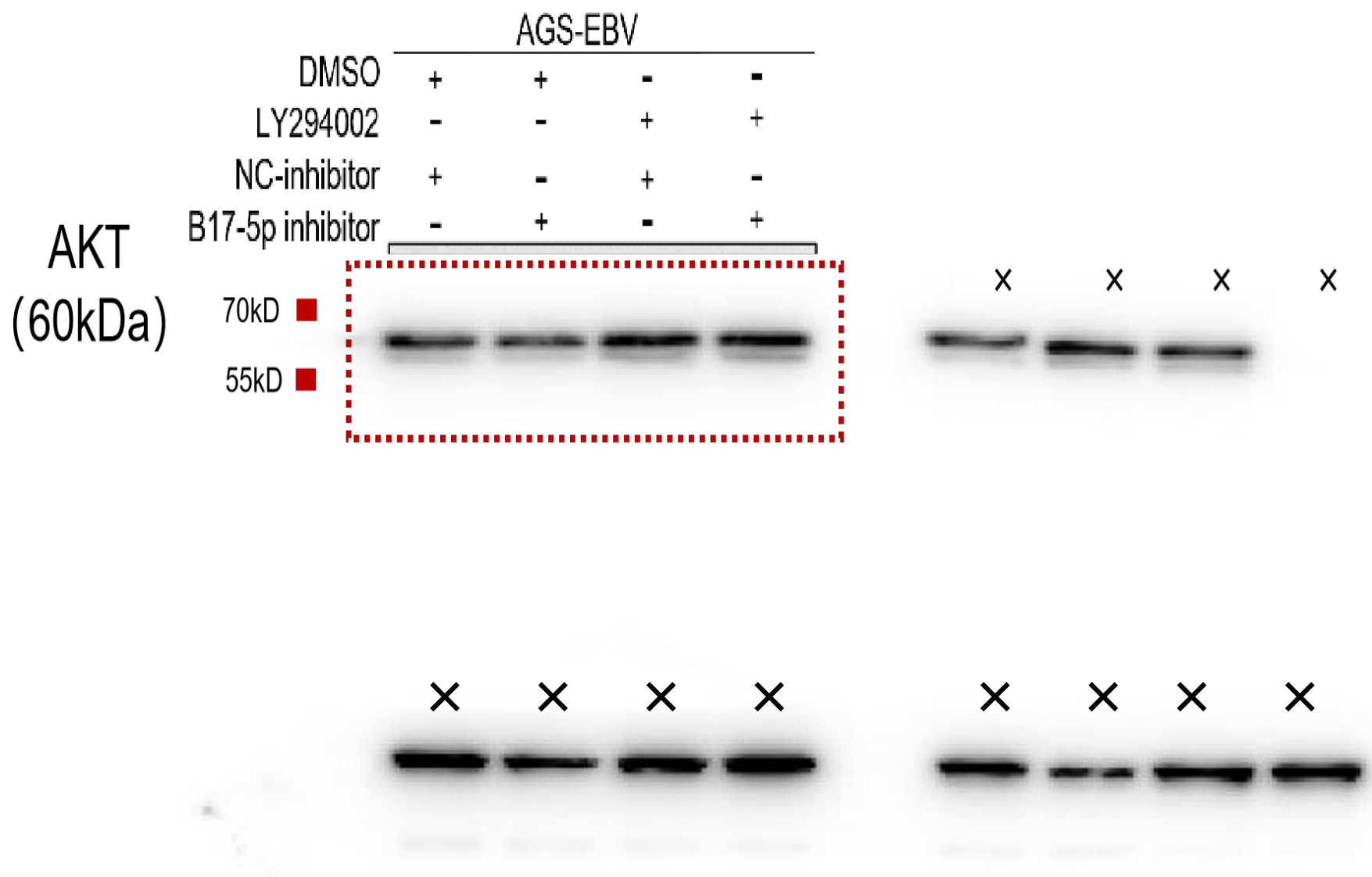

These images correspond to Figure 7C

Biological Replicate #3

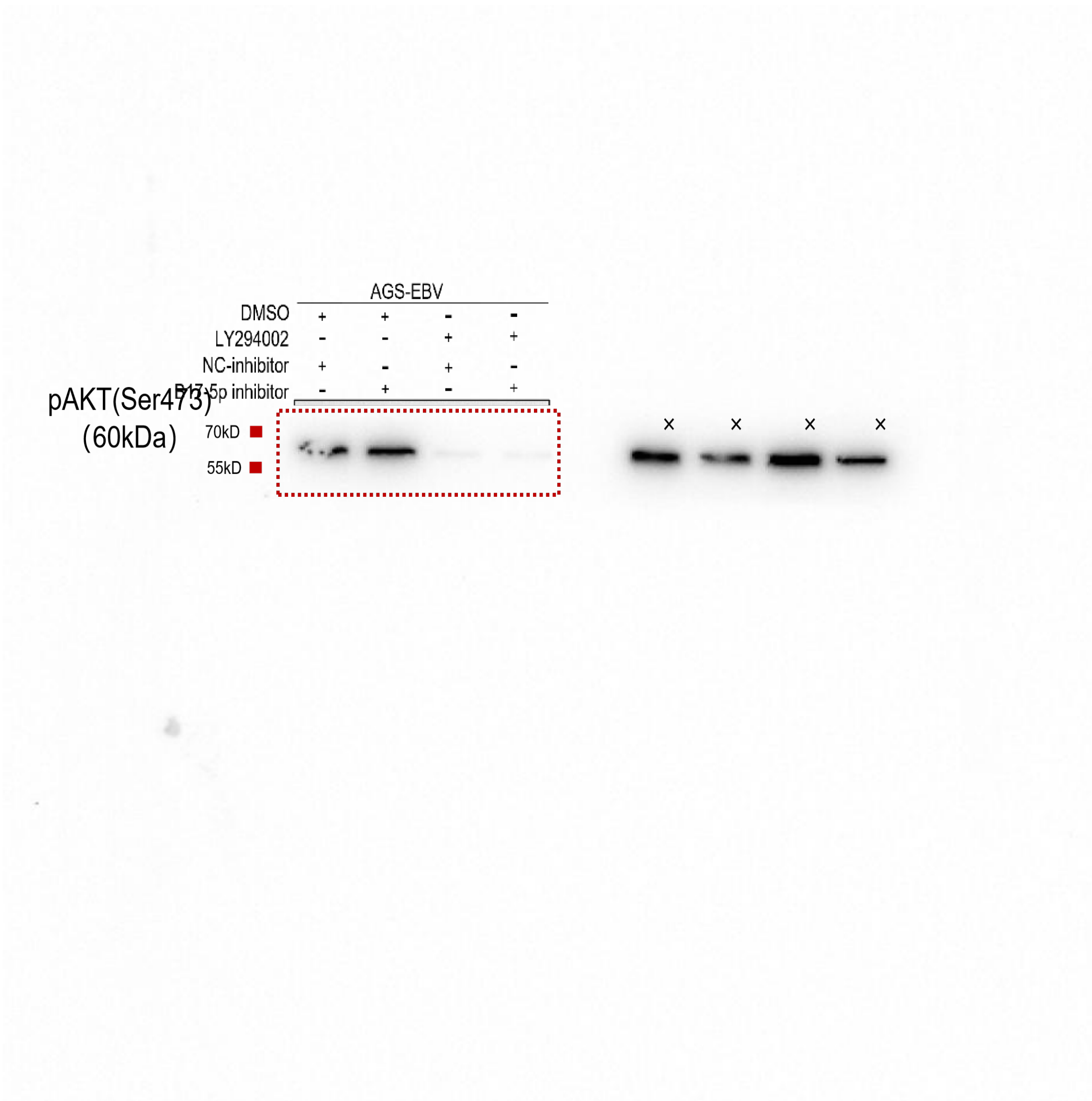

These images correspond to Figure 7C

Biological Replicate #3

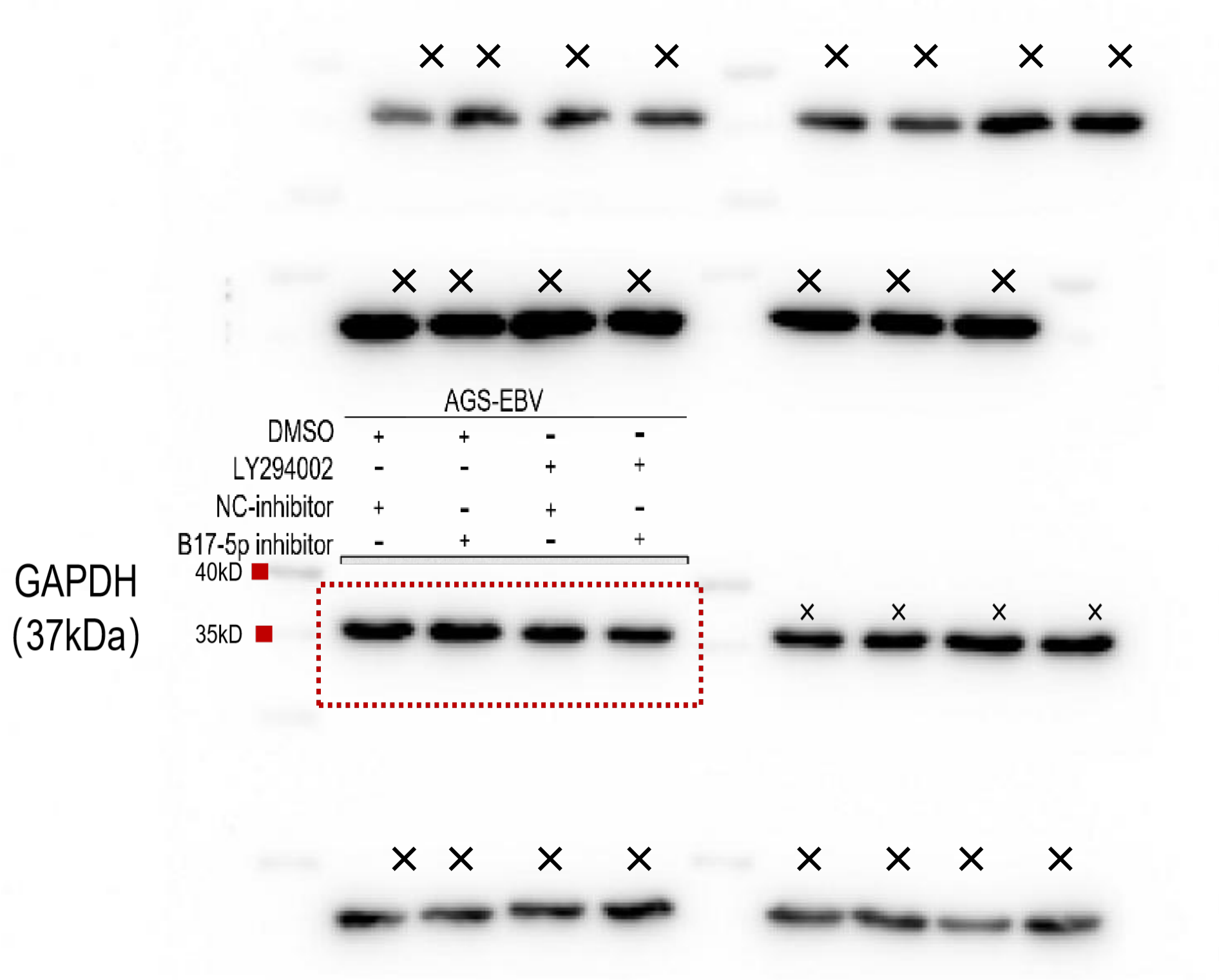

These images correspond to Figure 8J

pAKT(Ser473)
(60kDa)

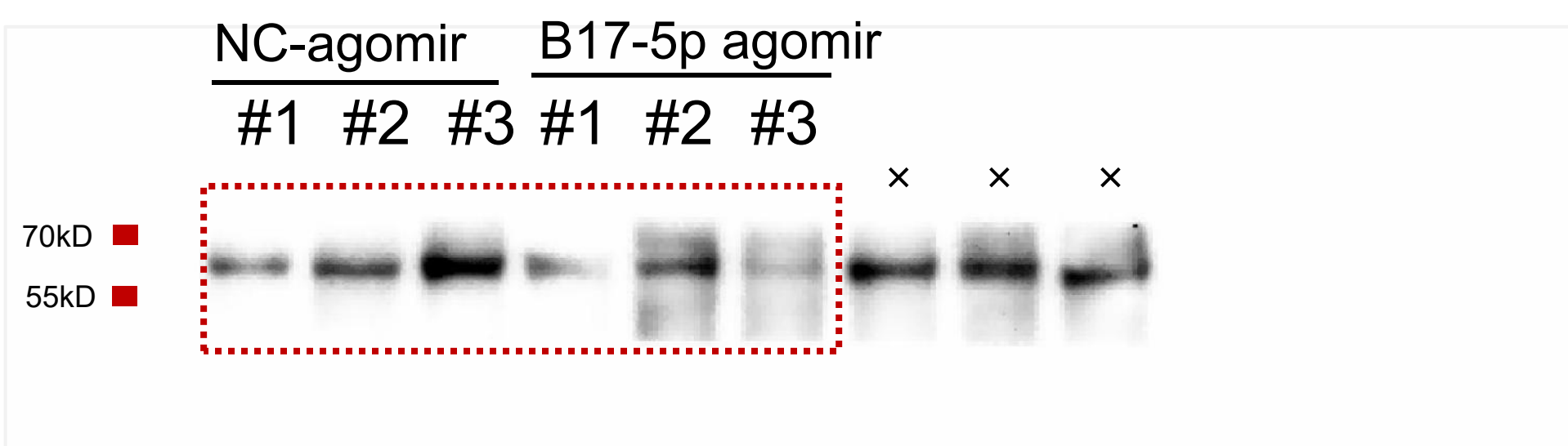

AKT
(60kDa)

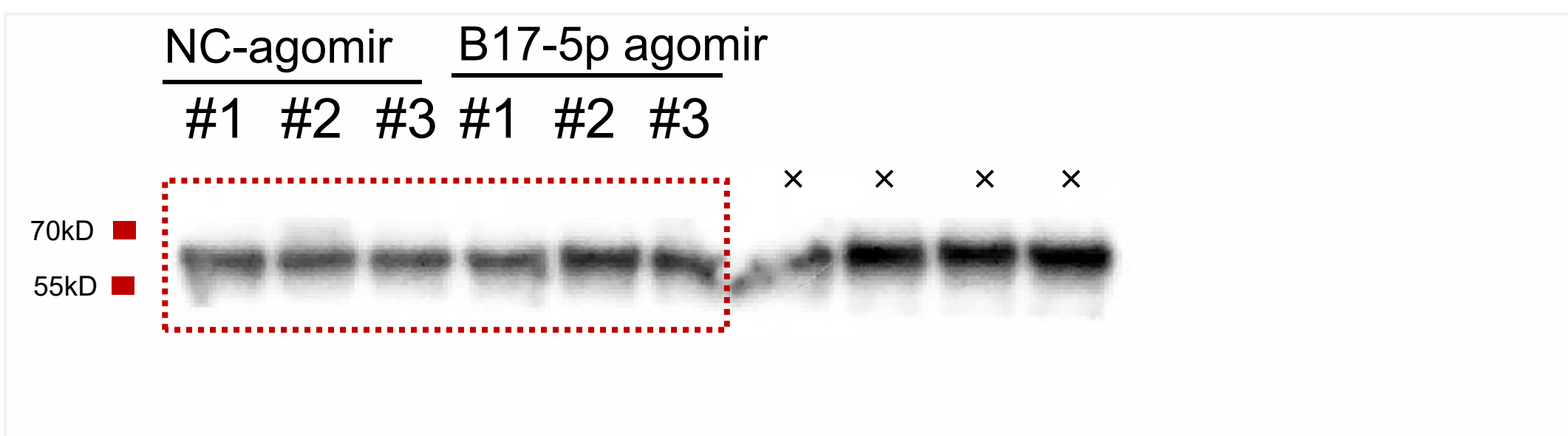

CERK
(60kDa)

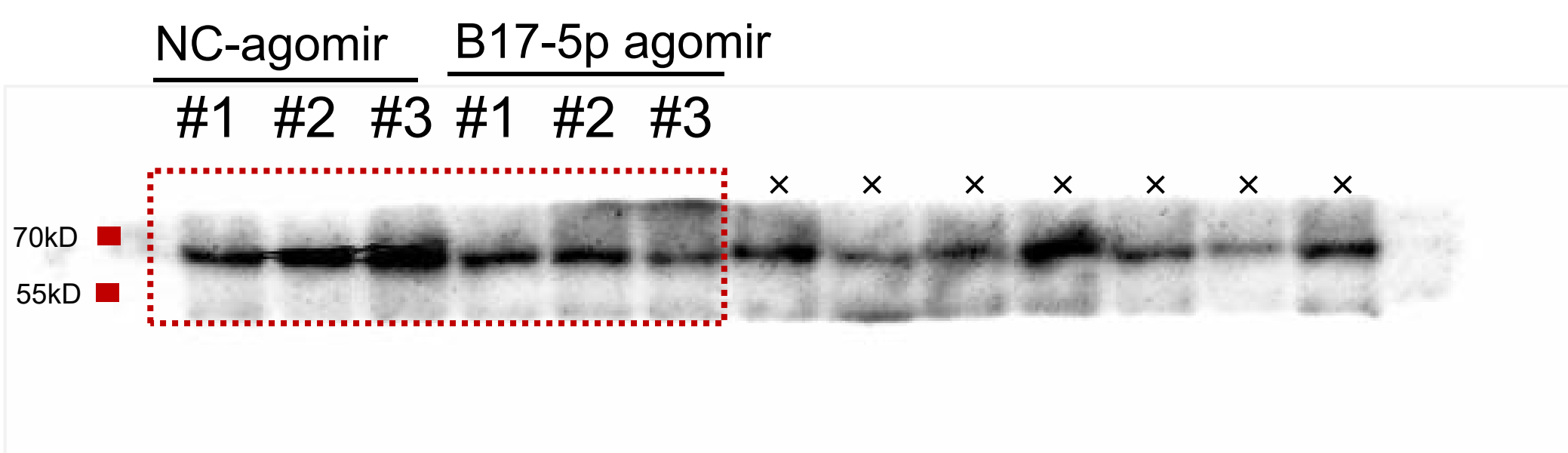

GAPDH
(37kDa)

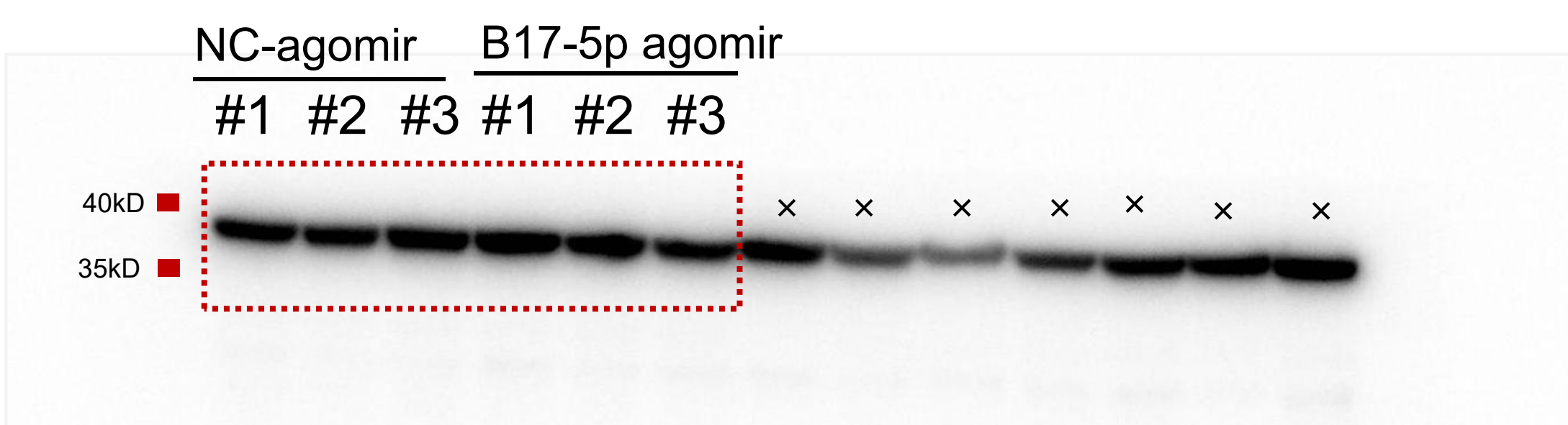

This image corresponds to Figure 8J

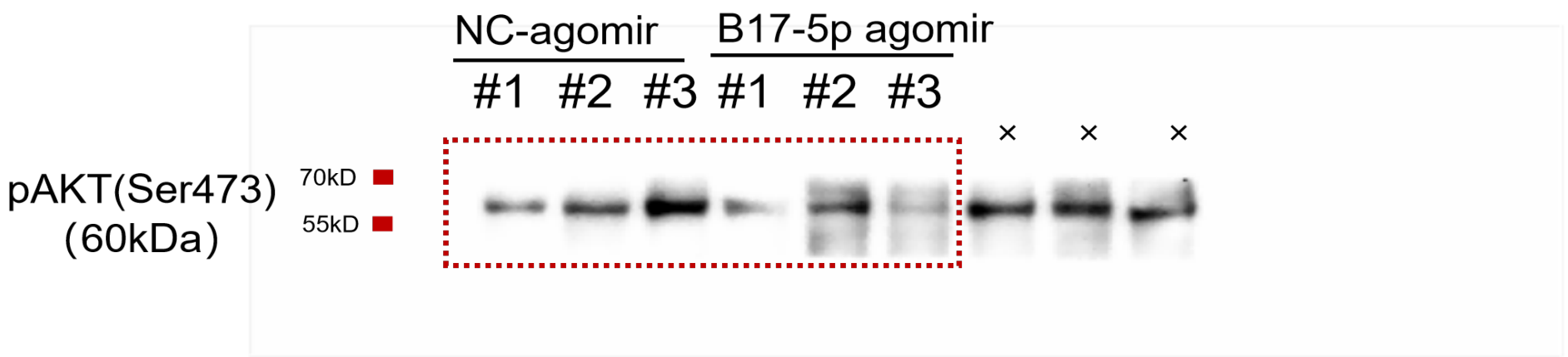

This image corresponds to Figure 8J

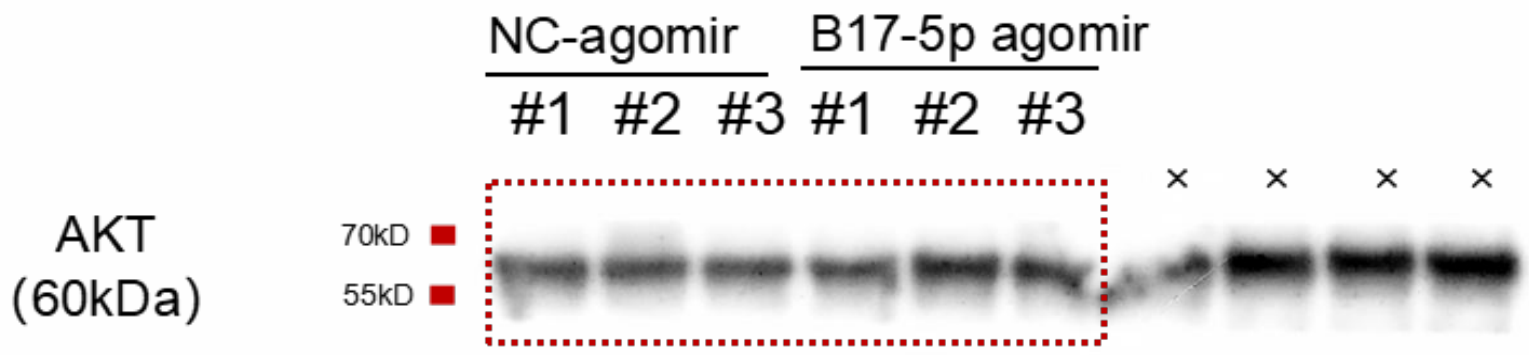

This image corresponds to Figure 8J

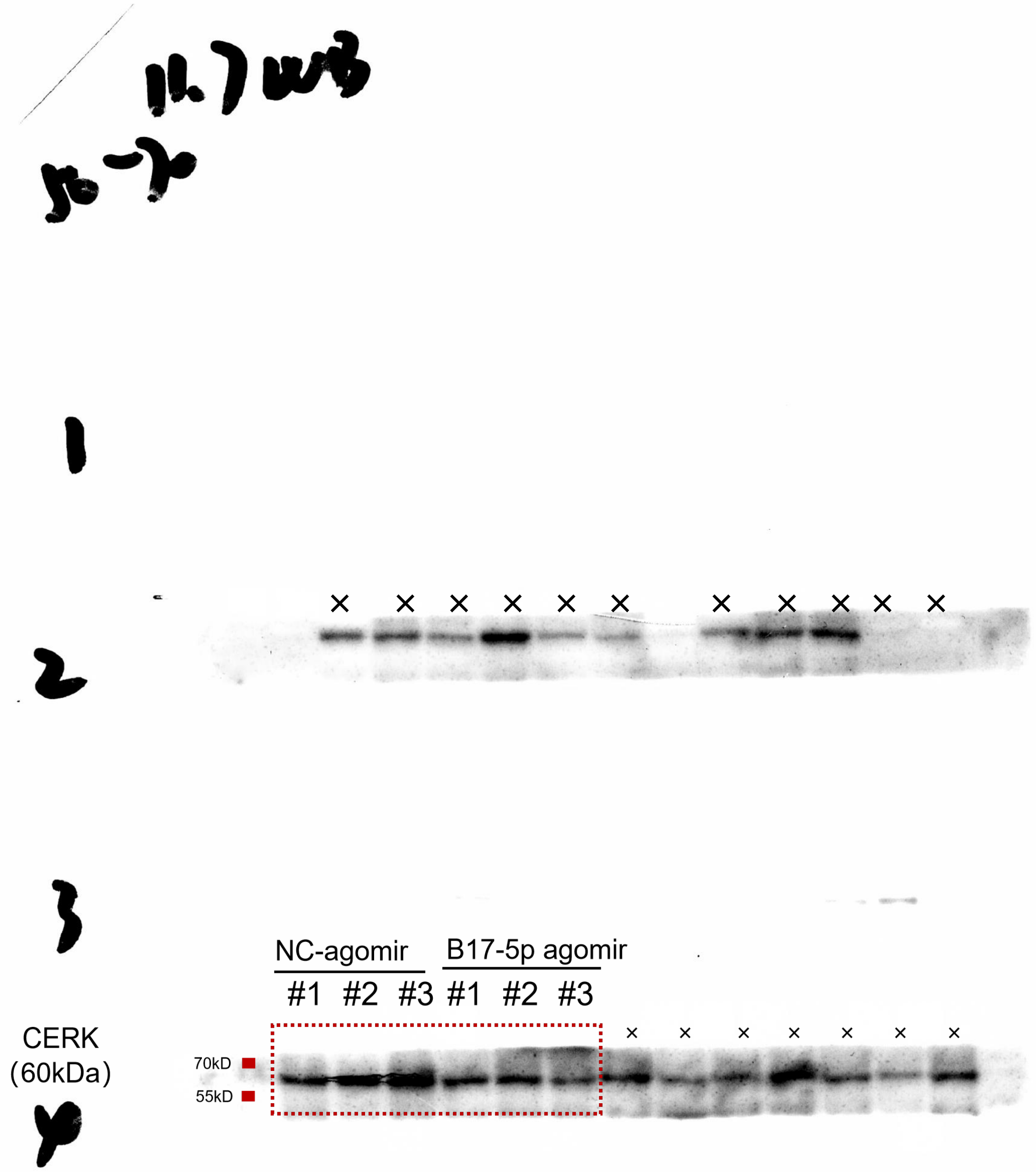

This image corresponds to Figure 8J

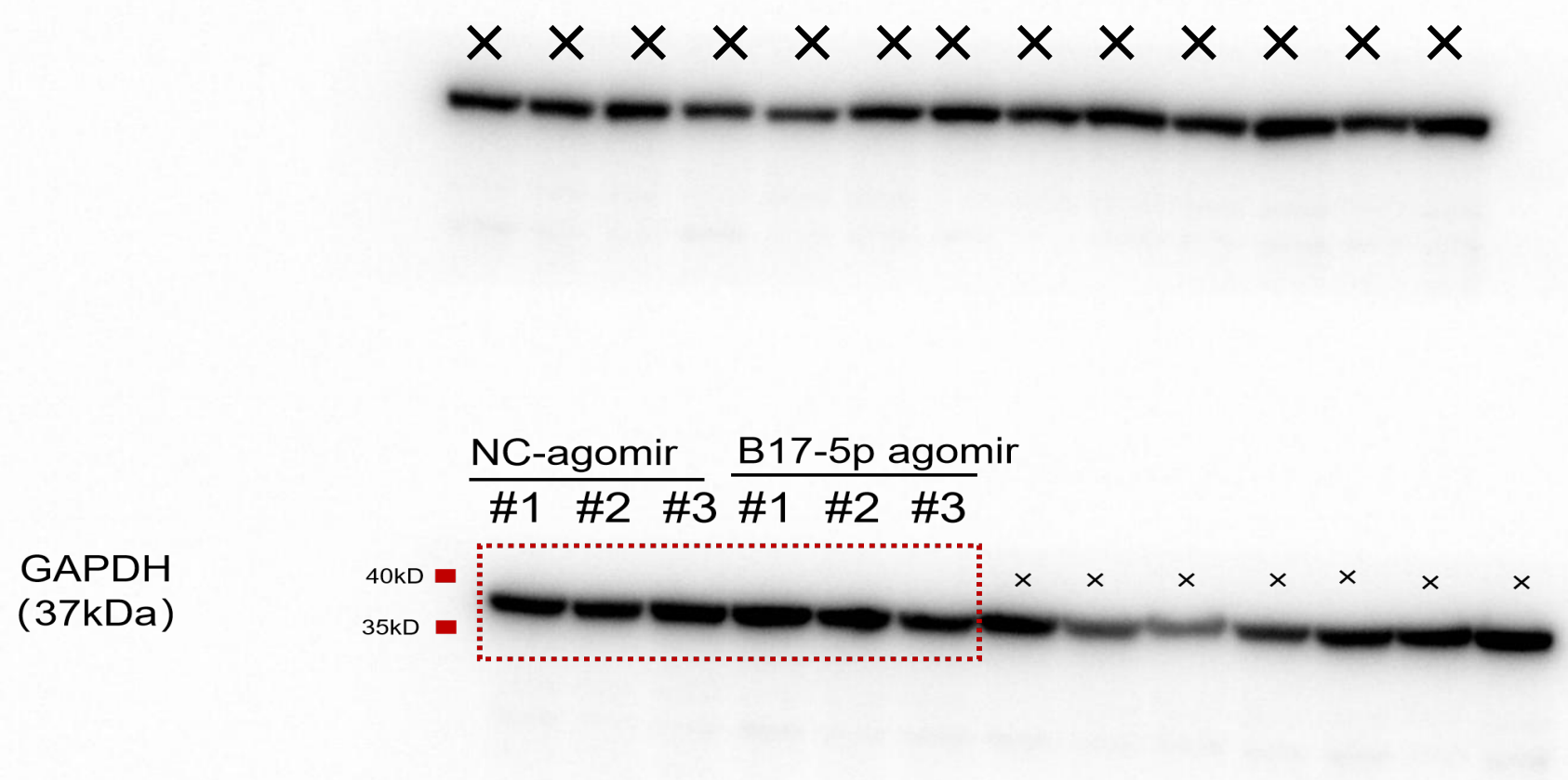

These images correspond to Figure 8L

pAKT(Ser473)
(60kDa)

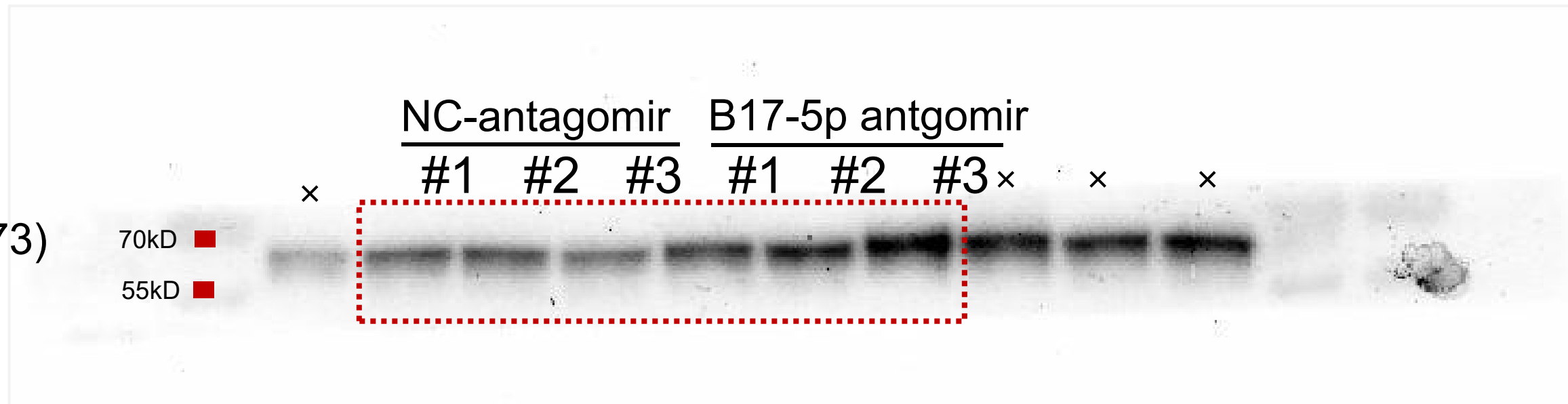

AKT
(60kDa)

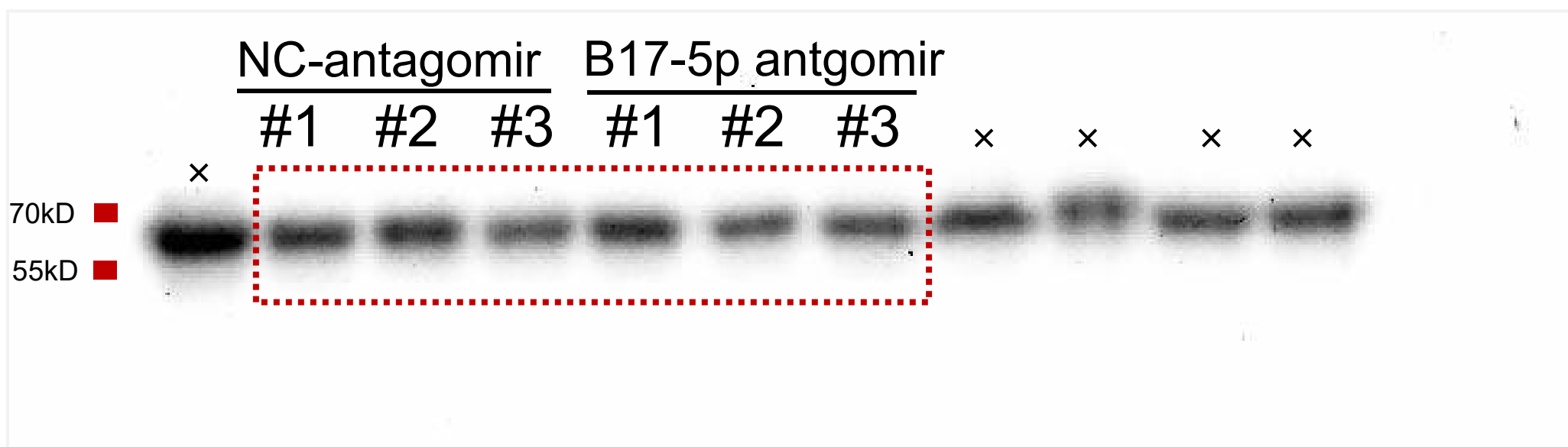

CERK
(60kDa)

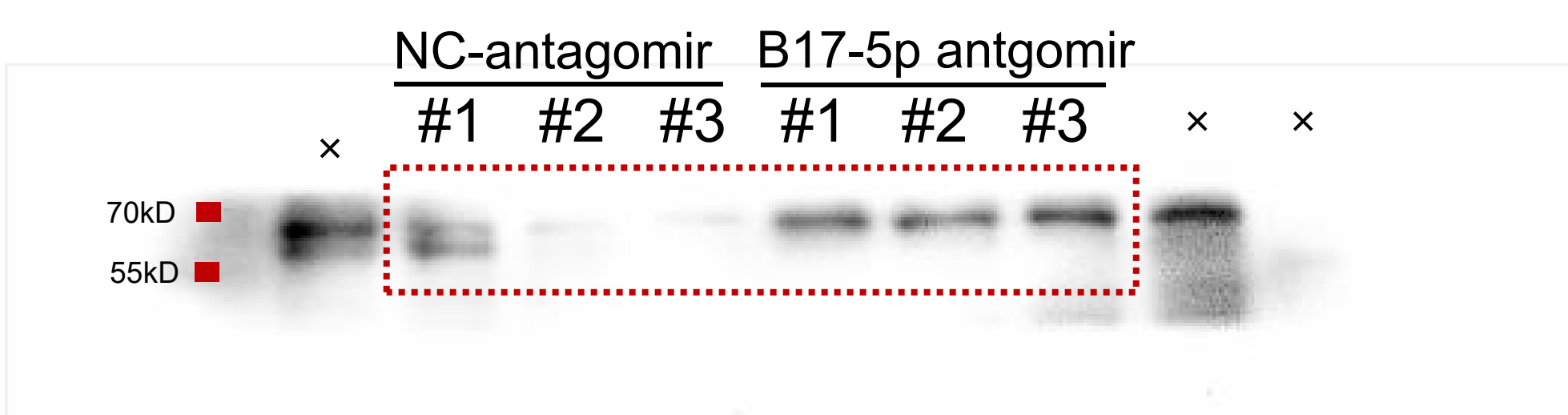

GAPDH
(37kDa)

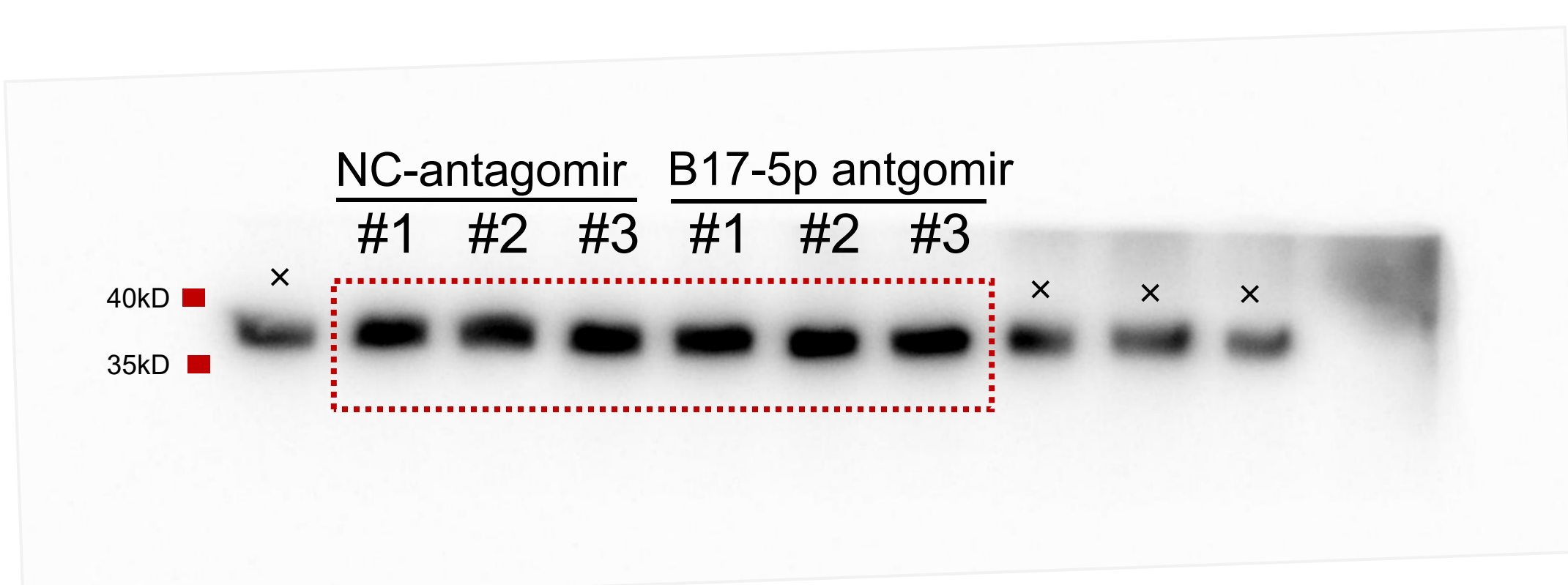

This image corresponds to Figure 8L

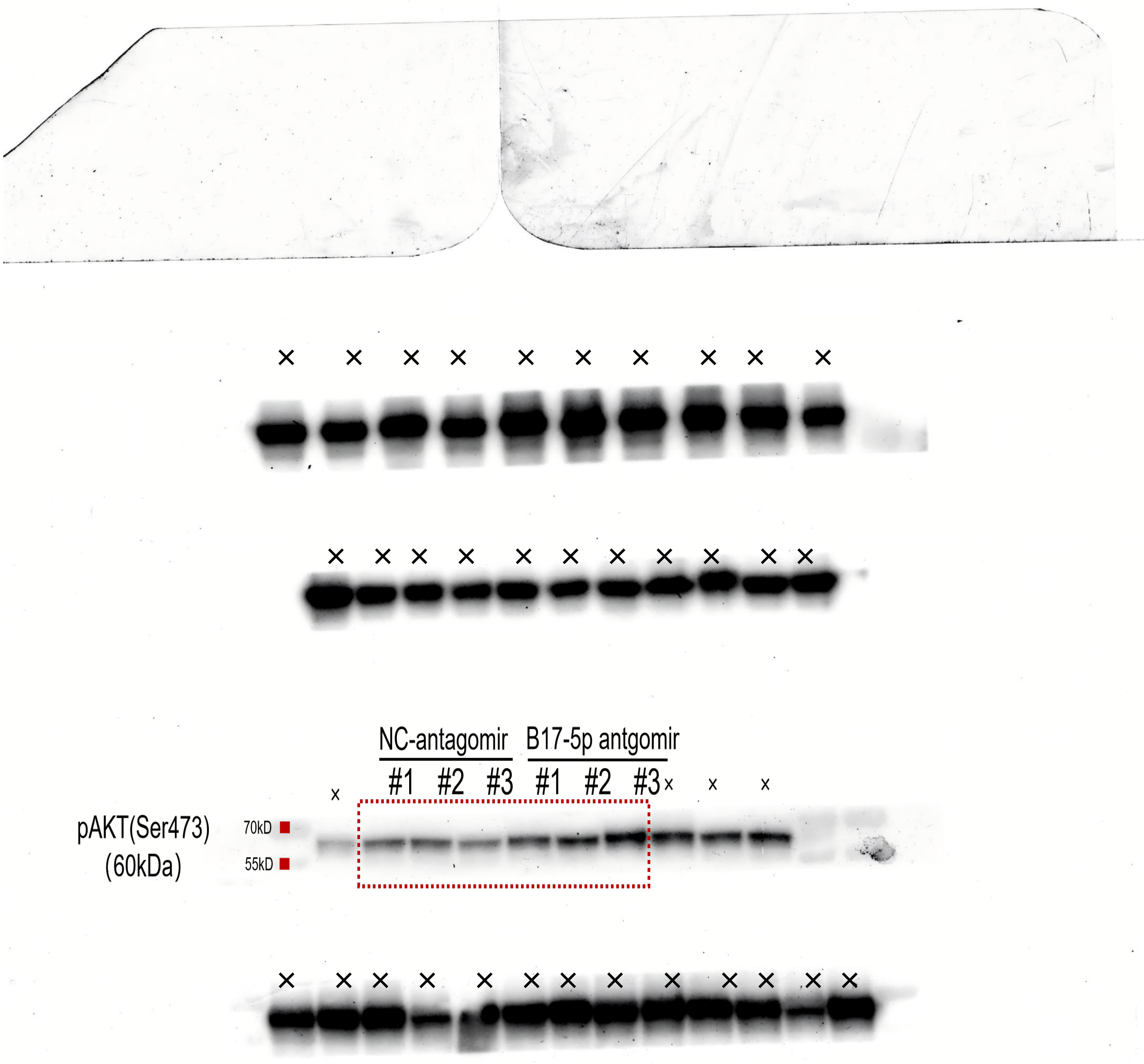

This image corresponds to Figure 8L

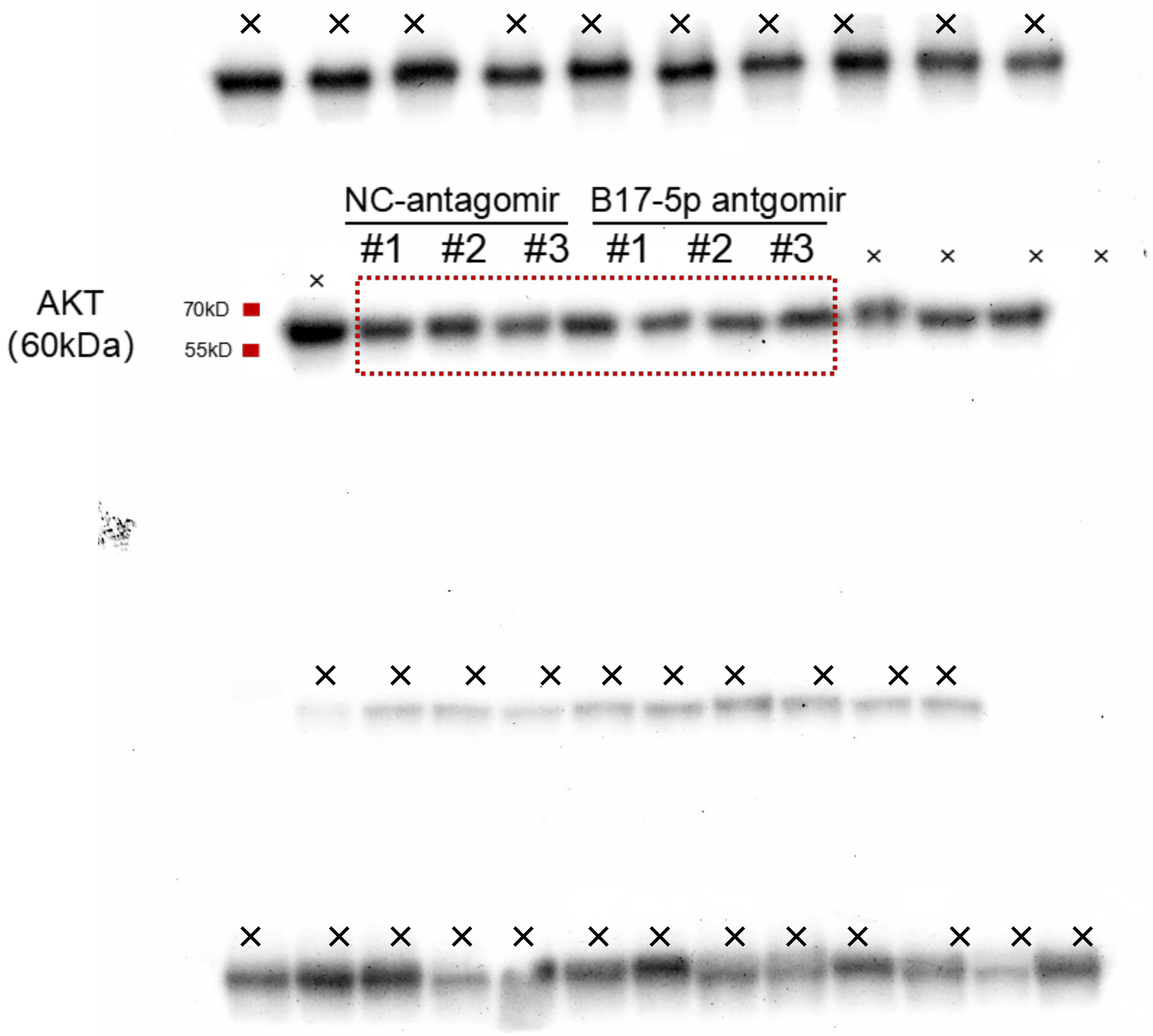

This image corresponds to Figure 8L

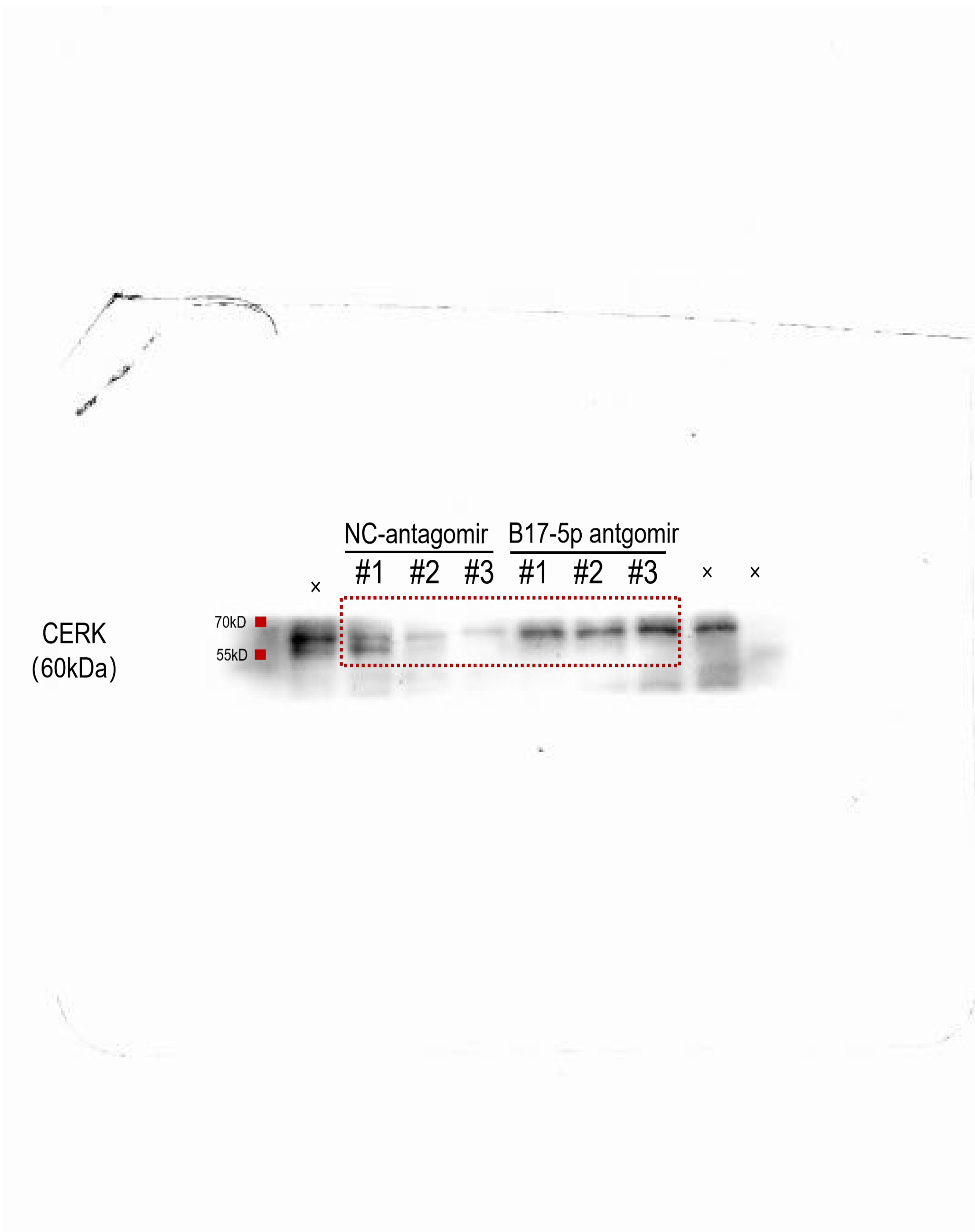

This image corresponds to Figure 8L

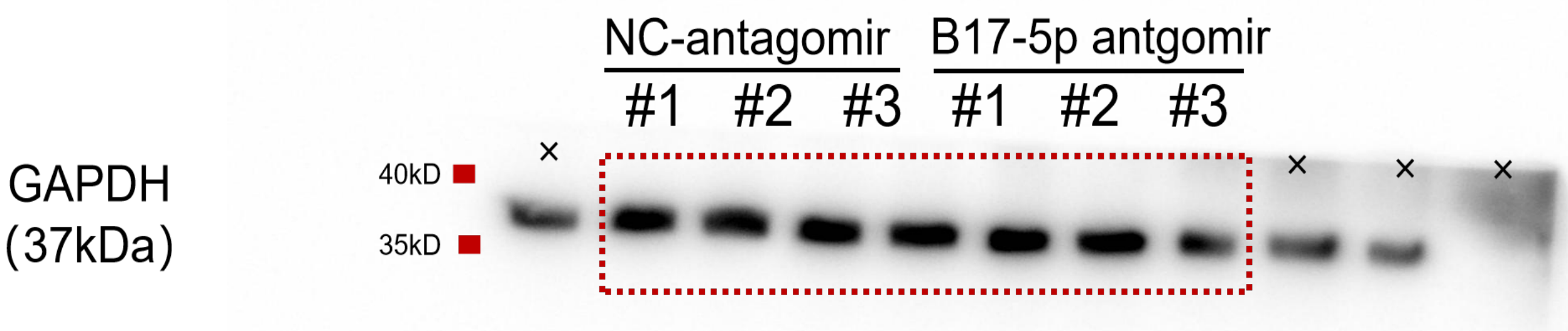

Supplement: Supplementary file 1 [file ppat.1014519.s005.pdf]
